# The 2022 Europe report of the *Lancet* Countdown on health and climate change: towards a climate resilient future

**DOI:** 10.1016/S2468-2667(22)00197-9

**Published:** 2022-10-26

**Authors:** Kim R van Daalen, Marina Romanello, Joacim Rocklöv, Jan C Semenza, Cathryn Tonne, Anil Markandya, Niheer Dasandi, Slava Jankin, Hicham Achebak, Joan Ballester, Hannah Bechara, Max W Callaghan, Jonathan Chambers, Shouro Dasgupta, Paul Drummond, Zia Farooq, Olga Gasparyan, Nube Gonzalez-Reviriego, Ian Hamilton, Risto Hänninen, Aleksandra Kazmierczak, Vladimir Kendrovski, Harry Kennard, Gregor Kiesewetter, Simon J Lloyd, Martin Lotto Batista, Jaime Martinez-Urtaza, Carles Milà, Jan C Minx, Mark Nieuwenhuijsen, Julia Palamarchuk, Marcos Quijal-Zamorano, Elizabeth J Z Robinson, Daniel Scamman, Oliver Schmoll, Maquins Odhiambo Sewe, Henrik Sjödin, Mikhail Sofiev, Balakrishnan Solaraju-Murali, Marco Springmann, Joaquin Triñanes, Josep M Anto, Maria Nilsson, Rachel Lowe

**Affiliations:** a Institute for Global Health, University College London, London, UK; b Institute for Sustainable Resources, University College London, London, UK; c Energy Institute, University College London, London, UK; d Cardiovascular Epidemiology Unit, Department of Public Health and Primary Care, Cambridge University, Cambridge, UK; e Heidelberg Institute of Global Health, University of Heidelberg, Heidelberg, Germany; f Department of Public Health and Clinical Medicine, Umeå University, Umeå, Sweden; g Department of Epidemiology and Global Health, Umeå University, Umeå, Sweden; h Barcelona Institute for Global Health (ISGlobal), Barcelona, Spain; i Universitat Pompeu Fabra (UPF), Barcelona, Spain; j CIBER Epidemiología y Salud Pública (CIBERESP), Barcelona, Spain; k BC3 Basque Centre for Climate Change, Bilbao, Spain; l School of Government, University of Birmingham, Birmingham, UK; m Data Science Lab, Hertie School, Berlin, Germany; n Priestley International Centre for Climate, University of Leeds, Leeds, UK; o Mercator Research Institute on Global Commons and Climate Change, Berlin, Germany; p Energy Efficiency Group, Institute for Environmental Sciences (ISE), University of Geneva, Switzerland; q Centro Euro-Mediterraneo sui Cambiamenti Climatici (CMCC), Venice, Italy; r Grantham Research Institute on Climate Change and the Environment, London School of Economics and Political Sciences (LSE), UK; s Barcelona Supercomputing Center (BSC), Barcelona, Spain; t Finnish Meteorological Institute (FMI), Helsinki, Finland; u European Environment Agency, Copenhagen, Denmark; v European Centre for Environment and Health, WHO Regional Office for Europe, Bonn, Germany; w Air Quality and Greenhouse Gases Programme, International Institute for Applied Systems Analysis, Laxenburg, Austria; x Helmholtz Centre for Infection Research, Department of Epidemiology, Brunswick, Germany; y Department of Genetics and Microbiology, Universitat Autònoma de Barcelona, Barcelona, Spain; z Oxford Martin Programme on the Future of Food and Nuffield Department of Population Health, University of Oxford, Oxford, UK; aa Department of Electronics and Computer Science, Universidade de Santiago de Compostela, Santiago, Spain; ab Centre for Climate Change and Planetary Health, London School of Hygiene and Tropical Medicine (LSHTM), London, UK; ac Catalan Institution for Research and Advanced Studies (ICREA), Barcelona, Spain

## Executive summary

In the past few decades, major public health advances have happened in Europe, with drastic decreases in premature mortality and a life expectancy increase of almost 9 years since 1980. European countries have some of the best health-care systems in the world. However, Europe is challenged with unprecedented and overlapping crises that are detrimental to human health and livelihoods and threaten adaptive capacity, including the COVID-19 pandemic, the Russian invasion of Ukraine, the fastest-growing migrant crisis since World War 2, population displacement, environmental degradation, and deepening inequalities. Compared with pre-industrial times, the mean average European surface air temperature increase has been almost 1°C higher than the average global temperature increase, and 2022 was the hottest European summer on record. As the world's third largest economy and a major contributor to global cumulative greenhouse gas emissions, Europe is a key stakeholder in the world's response to climate change and has a global responsibility and opportunity to lead the transition to becoming a low-carbon economy and a healthier, more resilient society.

The *Lancet* Countdown in Europe is a collaboration of 44 leading researchers, established to monitor the links between health and climate change in Europe and to support a robust, evidence-informed response to protect human health**.** Mirroring the Global *Lancet* Countdown, this report monitors the health effects of climate change and the health co-benefits of climate action in Europe. Indicators will be updated on an annual basis and new indicators will be incorporated to provide a broad overview to help guide policies to create a more climate-resilient future.

### The health costs of delayed decarbonisation

The 2022 Intergovernmental Panel on Climate Change report exposed how dangerously close the world is to reaching climate-driven points of no return. Alarming increases in health-related hazards, vulnerabilities, exposures, and impacts from climate change across Europe show the urgent need for ambitious mitigation targets that restrict the global temperature rise to less than 1·5°C above pre-industrial levels and effective adaptation strategies to build resilience to the increasing health threats of climate change.

Population exposure to heatwaves increased by 57% on average in 2010–19 compared with 2000–09, and by more than 250% in some regions, putting older people, young children, people with underlying chronic health conditions, and people who do not have adequate access to health care at high risk of heat-related morbidity and mortality (indicator 1.1.2). Global warming observed between 2000 and 2020 has been associated with an estimated temperature-related mortality increase in most regions monitored, with an average of 15·1 additional deaths per million inhabitants per decade (95% CI –1·51 to 31·6; indicator 1.1.4). Besides the direct health impacts, heat exposure also undermines people's livelihoods and the social determinants of health by reducing labour capacity. Labour supply in highly exposed sectors (eg, agriculture) was lower in 2016–19 compared with 1965–94 because of increased heat exposure (indicator 4.1.2). Climate change is also driving increasingly intense and frequent climate-related extreme events in Europe, with both direct and indirect health impacts, loss of infrastructure, and economic costs. Between 2011 and 2020, 55% of the European regions have had extreme-to-exceptional summer drought (indicator 1.2.2), and climate-related extreme events were associated with record economic losses in 2021, totalling almost €48 billion (indicator 4.1.1). The changing environmental conditions are also shifting the environmental suitability for the transmission of various infectious diseases. An increasing percentage of coastal waters in Europe are showing suitable conditions for the transmission of pathogenic non-*cholerae Vibrio* (indicator 1.3.1), the climatic suitability for the transmission of dengue increased by 30% in the past decade compared with the 1950s (indicator 1.3.3), and the environmental risk of West Nile virus outbreaks increased by 149% in southern Europe and 163% in central and eastern Europe in 1986–2020 compared with 1951–85 (indicator 1.3.2). Warmer temperatures are also shifting flowering seasons of several allergenic tree species, with birch, olive, and alder seasons beginning 10–20 days earlier than 41 years ago, affecting the health of around 40% of the population in Europe who have pollen allergies (indicator 1.4.1).

These overlapping and interconnecting health impacts, which are evolving against a backdrop of a pandemic and a devastating war in Ukraine, reveal the urgent need for interventions that build resilience in the health sector and protect people from increasing health hazards. Some progress has been made in Europe's health adaptation. In 2021, 15 (68%) of 22 European countries reported having national health and climate change strategies or plans (indicator 2.1.2), and 10 (45%) reported conducting a climate change and health vulnerability and adaptation assessment (indicator 2.1.1). 150 European cities (76%) reported performing city-level climate assessments, with 118 (59·9%) reporting that climate change threatens their public health or health services (indicator 2.1.3). Population-weighted greenness increased from 2000 to 2020 in most European countries, with the largest percentage increase in southern Europe and the smallest increase in western Europe (indicator 2.2.2). Climate adaptation often needs to compete for scarce financial resources, and the enactment of adaptation plans alone is not sufficient to advance adaptive capacity. With the impacts of climate change on the rise, adaptation efforts must rapidly accelerate and be carefully implemented alongside mitigation strategies.

In a world 1·2°C warmer than pre-industrial times, the magnitude of the overlapping and interconnected health impacts of climate change is a warning of the consequences of exceeding the 1·5°C target of the Paris Agreement. Europe should reach net-zero greenhouse gas emission by 2050 to meet the Paris Agreement commitments. However, Europe's current emissions are excessively high at 5·6 tonnes (t)CO_2_ per person just from the combustion of fossil fuels for energy production (indicator 3.1.1). The region's delayed response could be costing millions of lives each year, not only by exacerbating the health impacts of climate change, but also given the missed direct and indirect health co-benefits that more ambitious climate action could deliver. The continued burning of fossil fuels led to 117 000 deaths in 2020 from exposure to particulate matter of less than 2·5 μm in diameter (PM_2·5_) air pollution, with the transport sector being the main contributor (indicator 3.2). Importantly, coal contributed to 12% of the total energy supply in Europe in 2020, an inefficient fuel source that substantially contributes to air pollution (indicator 3.1.2). The excessive consumption of high-carbon, meat-rich diets contributed to an estimated 2·2 million deaths in 2019 (indicator 3.4.1), and European food demand was estimated to be responsible for 2·5 tCO_2_ equivalent (eq) emitted per person, accounting for 37% of the carbon footprint of the average person in EU27 (ie, the 27 countries in the EU after the UK left; indicator 3.4.2). However, despite the clear health impacts of climate change and the substantial health opportunities of climate action, 23 (43%) of 53 European countries analysed are allocating public funds to deliver overall fossil fuel subsidies, financially constraining decarbonisation targets (indicator 4.2.1).

The delayed implementation of locally generated, low-carbon energy sources has made Europe susceptible to volatile energy prices, which reached record high values in 2022. The Russian invasion of Ukraine has shown Europe's over-reliance on fossil fuels, exacerbating the energy crisis. While the world is trying to recover from the COVID-19 pandemic and responding to multiple coinciding disasters, recovery is hindered by the negative climate change impacts on health and its determinants, emphasising the urgent need for action.

### A transformational change for health

To avoid a catastrophic increase in global temperatures, the Intergovernmental Panel on Climate Change makes it clear that Europe must fully decarbonise its power sector by 2035, with all coal-fired power plants globally closing by 2040. Despite the scarce climate action in Europe to date, indicators within this report suggest that change might be underway. Although engagement with the intersection of health and climate change is low compared with overall engagement with climate change more generally, political engagement with health and climate change in the European Parliament has slightly increased since 2014 (indicator 5.3). Engagement of the scientific sector (indicator 5.1) since 2014 and engagement of the corporate sector (indicator 5.4) since 1990 have also increased. These increases have been accompanied by small changes in the energy system; energy generation from renewable sources is increasing at a rate of 16% per year (indicator 3.1.3), and if this rate is maintained, Europe's energy system could almost fully decarbonise within 10 years.

Europe's response to the war in Ukraine and the energy crisis will be important in forming Europe's new geopolitical situation. The energy crisis and decades of delay in switching to low-carbon energy generation risks a change to greater coal power generation in the short term. Even as a temporary measure, an increase in coal use could add to the approximately 8000 annual deaths associated with coal-fired power plants, in the domestic sector (indicator 3.2), reversing the health gains made in the past decade and undermining efforts to meet Paris Agreement commitments. Increasing Europe's reliance on fossil fuels would further accelerate global warming, increase air pollution, and be detrimental to health and wellbeing.

The REPowerEU plan published in March, 2022, aiming to accelerate the transition to clean energy sources, provides hope, reaffirming Europe's leadership in low-carbon systems by providing direct economic benefits, energy sovereignty, and security, the net creation of more equitable jobs, and added health benefits with the reduced burning of fossil fuels. The indicators in this report show that an accelerated transition to clean energy could save lives each year.

### The biggest public health opportunity of the century

With a world dangerously close to reaching climate-driven points of no return and an increasing energy crisis, and with the health of populations increasingly undermined by global warming, Europe is at a crucial point for change. If climate mitigation and adaptation plans are designed and implemented with health, wellbeing, and equity as the main focus, this could represent the biggest public health policy opportunity of the century. Ambitious European adaptation and mitigation strategies will not only protect lives and wellbeing in Europe, but also in countries that have contributed least to anthropogenic climate change. The danger of reaching a point of no return means that Europe cannot afford to miss such opportunity.

## Introduction

Major public health gains have been made in Europe, with life expectancy increases of almost 9 years since 1980.^1^, ^2^, ^3^ However, Europe is challenged with unprecedented and overlapping crises that are detrimental to health and threaten resilience to climate change; these include the COVID-19 pandemic, the Russian invasion of Ukraine, population displacement, environmental degradation, and deepening socioeconomic inequalities.^4^, ^5^ In Europe, average surface air temperatures have increased by 2·2°C since pre-industrial times (1850–1900),^6^, ^7^ about 1°C higher compared with the corresponding global temperature increase of 1·2°C.^7^ The hottest summer on record was in 2022.^8^ Without accelerated mitigation and adaptation, ongoing climate change will have irreversible, multidimensional impacts on human health resulting from exposure to extreme climatic events, heat-related morbidity and mortality, altered environmental suitability, and exposure to infectious diseases.^5^, ^9^

As the world's third largest economy, the EU has contributed 17% of global cumulative greenhouse gas emissions (1950–2012).^7^ Europe is a key stakeholder in the world's response to climate change, and has the opportunity to lead the way in the transition to low carbon, healthier economies, and increased climate resilience.^5^ In 2021, the EU's commitment to reduce greenhouse gas emissions was accepted into law, with the aim to reduce greenhouse gases by at least 55% from emission levels in 1990 by 2030 and reach net-zero emissions by 2050.^10^ Russia's invasion of Ukraine in Feb, 2022, has brought a new context of political instability and human crisis and highlighted Europe's dependency on fossil fuel imports. This dependency highlights the urgent need to transition to clean energy sources to reduce greenhouse gas emissions while ensuring energy security and affordability.^11^

This is the first report of the *Lancet* Countdown Europe.^2^ The report draws on broad expertise, including that of epidemiologists and public health experts, climate scientists, economists, social scientists, and political scientists from 29 leading European academic and UN institutions. Together, 44 contributors report on 33 indicators, monitoring and quantifying the health impacts of climate change and the health co-benefits of accelerated action since the 1950s. The report mirrors that of the global *Lancet* Countdown report, tracking progress on health and climate change in five areas: climate change impacts, exposures, and vulnerabilities; adaptation, planning, and resilience for health; mitigation actions and health co-benefits; economics and finance; and politics and governance. The geographical coverage of each indicator is reported (appendix pp 4–5), with most indicators covering all 38 European Environment Agency (EEA) member and cooperating countries, plus the UK. Methods and underlying data are presented in the appendix (pp 12–193).

## Section 1: climate change impacts, exposures, and vulnerabilities

Europe is experiencing multidimensional health impacts due to climate change. This section presents indicators tracking the change in hazards, exposures, vulnerabilities, and risks for a selection of climate-sensitive health outcomes. The indicators are distributed into four clusters, with a total of 11 indicators monitoring health outcomes associated with rising temperatures (indicators 1.1.1 to 1.1.4), extreme events (indicators 1.2.1 and 1.2.2), climate-sensitive infectious diseases (indicators 1.3.1 to 1.3.4), and allergens (indicator 1.4).

### Indicator 1.1: health and heat

#### Indicator 1.1.1: vulnerability to heat exposure

Heat exposure poses acute health risks, particularly to older people (ie, people older than 65 years), people with underlying, chronic respiratory, kidney, or heart disease, people living in urban areas, and people with little means to access cooling mechanisms.^12^, ^13^, ^14^ These heat-related health risks are of particular relevance to Europe, as the continent is experiencing ageing populations, urbanisation, and a high prevalence of chronic diseases. In this indicator, a heat vulnerability index was computed by combining the proportion of the population who are older than 65 years, live in urban areas, and have a chronic disease (cardiovascular disease, respiratory disease, diabetes, and kidney disease). Vulnerability to heat exposure has increased steadily across all European regions, with an increase of 6% from 1990 to 2019. Although northern Europe is the most vulnerable region, the highest relative increase of 9·8% is observed in central Europe (appendix pp 12–14).

#### Indicator 1.1.2: exposure of vulnerable populations to heatwaves

European populations are being exposed to increasingly frequent, intense, and extensive heatwaves,^15^ with the unprecedented heatwaves in June and July, 2022, exceeding all-time national temperature records in multiple European countries.^16^ This indicator monitors the exposure of vulnerable populations, defined here as people older than 65 years and infants between 0 years and 1 years to heatwaves. Comparing the decadal mean average of heatwave exposure days from 2000 to 2009 with exposure days from 2010 to 2020, heatwave exposure in vulnerable groups increased by 57% across Europe (appendix pp 15–17), from a yearly mean average of 0·65 billion person-days between 2000 and 2009 to 1·07 billion person-days between 2010 and 2020 in populations older than 65 years. In some areas, the increase has exceeded 157% (appendix pp 15–17). In 2020, 1·21 billion person-days of heatwave exposure were calculated, mostly comprising exposure of people older than 65 years, with an additional 3·1 million person-days in infants under 1 year old.

#### Indicator 1.1.3: heat stress risk related to physical activity

**Figure 1:**
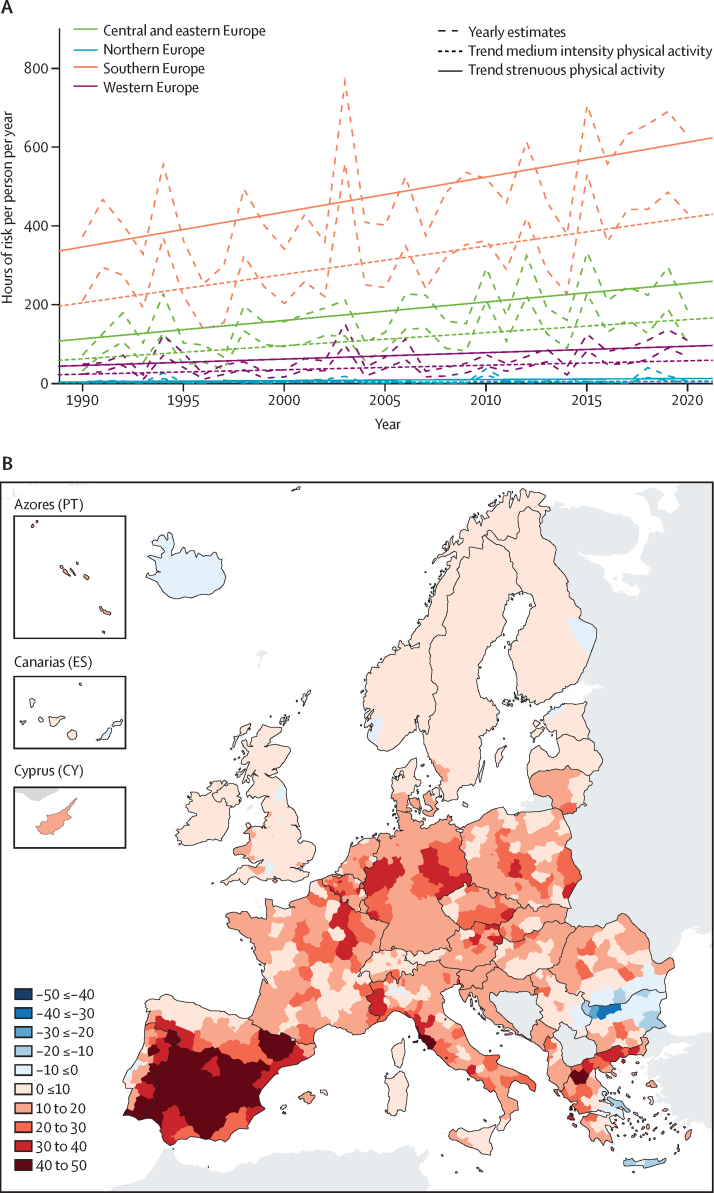
Heat and human health in Europe (A) Hours of risk per person per year (1990–2020) with 95% confidence intervals for physical-activity-related heat stress per European region, for activities of medium and high intensity. (B) Trends in heat related mortality incidence (annual death per million per decade) in Europe for the general population (2000–20).

Regular physical activity provides major physical and mental health benefits.^17^, ^18^ However, exercising during extreme heat has an acute risk of heat stress and heatstroke (figure 1A; appendix pp 18–23).^19^ This indicator reports the number of hours in which heat exposure poses a risk to health during physical exercise unless actions are taken to reduce the risk, while accounting for the intensity of the activity.^20^, ^21^ Overall, the number of hours of risk per person is increasing across all European regions. In southern Europe, the number of hours with heat-related health risks during medium-intensity activities (eg, football or tennis) increased relatively by 106% between 1990 and 2020, and increased to 429 hours per person in 2020. For strenuous activities (eg, mountain biking), there was a relative increase of 77% in southern Europe, leading to 627 hours at risk per person in 2020.^20^, ^21^

#### Indicator 1.1.4: heat-related mortality

Without accelerated mitigation and adaptation actions, climate change projections for Europe suggest a progressive reduction in cold-related deaths and a simultaneous increase in heat-related deaths,^22^, ^23^ with projections consistently indicating that the increase in heat-related deaths will exceed reductions of cold-related deaths by the second half of the 21st century.^23^ Indicator 1.1.4 combines epidemiological models with weekly European Centre for Medium Range Weather Forecasts (ERA5-Land) temperatures and Eurostat mortality counts^24^, ^25^, ^26^ to track trends in heat-related mortality. Heat-related deaths are estimated to have increased in 931 (94%) of the 990 regions monitored (appendix pp 24–27) from 2000 to 2020, with an overall mean increase of 15·1 (95% CI –1·51 to 31·6) annual deaths per million inhabitants per decade for the general population (figure 1B), and 60·4 (–17·8 to 138·6) extra deaths per million inhabitants per decade for people 65 years and older (appendix pp 24–27). Country-level figures range from 30·6 (6·32 to 54·9) annual deaths per million inhabitants per decade in Spain to –1·53 (–6·33 to 3·27) in Iceland. Assuming a linear extrapolation of the mortality trend, heat-related deaths in Europe could double in 34 years.^22^, ^23^

### Indicator 1.2: extreme events and health

#### Indicator 1.2.1: wildfire smoke

The changing climate is making weather conditions increasingly suitable for wildfires.^4^ Under a no-adaptation scenario, burned areas could increase by 200% in Europe this century compared with 2000–2008.^27^ Exposure to wildfire smoke is associated with increased mortality, morbidity, and hospital admissions and exacerbates respiratory and cardiovascular conditions.^28^, ^29^ This indicator combines atmospheric models, remote fire detection, weekly death counts, and epidemiological models on the health impacts of PM_2·5_ exposure to track the annual population-weighted exposure to wildfire-related PM_2·5_ and attributable deaths (appendix pp 28–35).^30^

**Figure 2:**
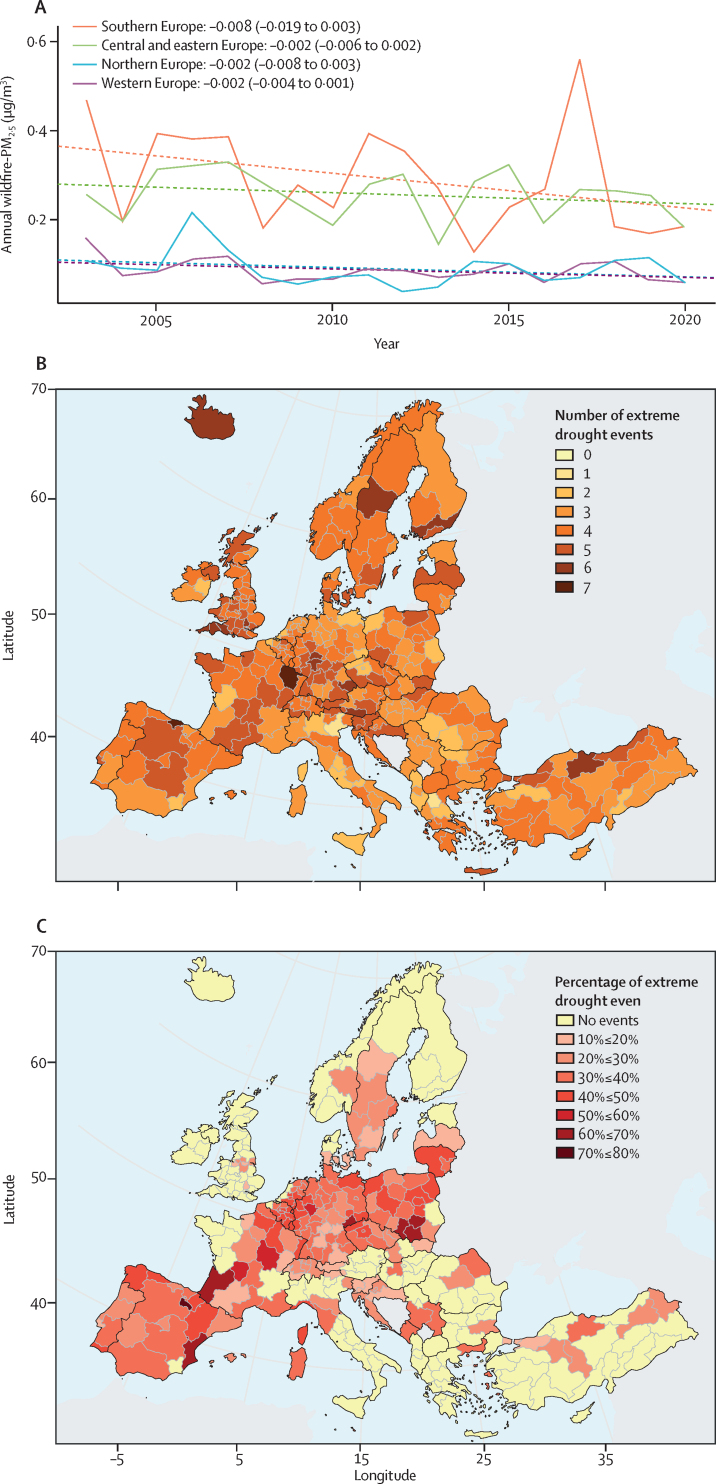
Extreme climatic events and related impacts in Europe (A) Annual average population-weighted wildfire-PM_2·5_ exposure by European region (bold) and linear trend (dashed) during the period 2003–20. Slope coefficients (95% CI) corresponding to the linear trend (wildfire-PM_2·5_ exposure change per 1-year increase) are shown as text. The negative trend lines are not statistically significant (appendix pp 28–35). (B) Total number of extreme drought events (SPEI6 ≤–1·6) during the extended summer period (April to September) in Europe between 1951 and 2020. (C) Percentage of extreme drought events (observed between 1951 and 2020) that occurred in the most recent 10 years (2011–20). SPEI6= Standardised Precipitation-Evapotranspiration Index 6.

Annual average population-weighted wildfire-PM_2·5_ exposures varied considerably each year and showed negative trends in all European regions (figure 2A), possibly because of increased effectiveness of fire prevention and suppression measures.^31^, ^32^ From 2003 to 2020, Portugal and Greece had the highest levels of wildfire smoke exposure in southern Europe, and Bulgaria and Romania had the highest levels in central and eastern Europe. Between 2015 and 2019, an average of 603 (95% CI 410–808) deaths were attributable to wildfire-related PM_2·5_ each year in Europe, showing the need for fire control measures to be strengthened as the environmental risk of wildfires continues to rise.

#### Indicator 1.2.2: drought

With increased temperatures and altered rainfall patterns, the risk of droughts is increasing in Europe.^33^ This indicator tracks the frequency of extreme to exceptional seasonal droughts in Europe (1951–2020) with the Standardised Precipitation–Evapotranspiration Index (SPEI6)^34^, which accounts for the impact of precipitation and potential heat-related evapotranspiration. Extreme-to-exceptional drought events were defined as SPEI6 values of –1·6 or less, accumulated over April to September. The indicator shows that 184 (55%) of 334 European regions (Nomenclature of Territorial Units for Statistics [NUTS] level 2) have had extreme-to -exceptional summer droughts in the past decade. In a third of the European NUTS 2 regions, more than 30% of all extreme droughts observed since 1950 have happened in the past 10 years (figure 2B and C; appendix pp 36–41).

### Indicator 1.3: climate-sensitive infectious disease

#### Indicator 1.3.1: non-cholerae Vibrio

Vibrio bacteria can lead to severe gastrointestinal infections, skin and ear infections, and more severe health outcomes, including necrotising fasciitis, amputation, sepsis, and death.^35^ In Europe, cases have steadily increased over the years in countries with national surveillance; however, vibriosis is not a notifiable disease in the EU.^36^ Increasing sea temperatures have led to higher percentages of coastal areas with brackish waters in Europe showing suitable conditions for the transmission for non*-cholerae Vibrio* bacteria. Seashores around the Baltic Sea are particularly suitable, with a steady growth in the number of days and kilometres of coast suitable since 2004 (appendix pp 42–45). Almost the entire coast of Sweden, Finland, Estonia, Latvia, Lithuania, and Poland showed suitable conditions in 2020 (values ranging between 100% and 96·6%), with Germany (92%) and Denmark (83%) also showing suitable conditions in 2020. By contrast, values in southern Europe were low, with only 4% of coast suitable for non*-cholerae Vibrio* in Spain and 2% in Italy, because of higher surface salinity in the Mediterranean. As conditions become increasingly suitable for the transmission, early warning systems (section 2) and preventive measures will be essential to protect populations from these severe infections.^36^

#### Indicator 1.3.2: West Nile virus

**Figure 3:**
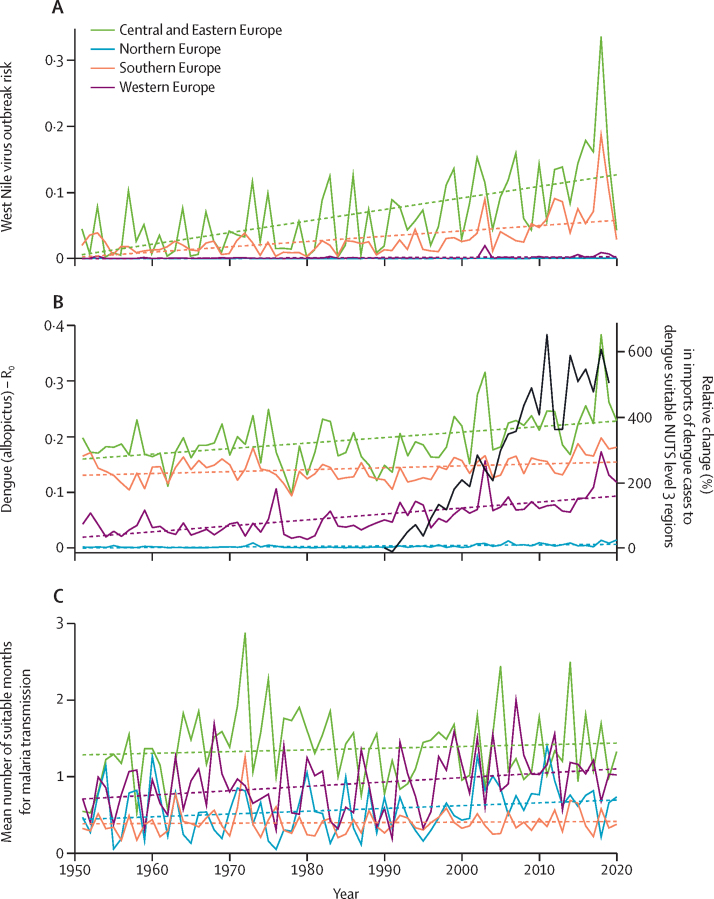
Climate suitability for West Nile virus, dengue, and malaria in Europe (A) Change in the estimated West Nile virus transmission risk probability between 1951 and 2020 in Europe. (B) R0 for dengue by European regions between 1951 and 2020. Black curve shows estimated relative change of yearly number of dengue case importations per NUTS level 3 region to transmission-suitable locations in Europe from dengue-endemic regions between 1990 and 2019. (C) Mean number of months suitable for *Plasmodium vivax* transmission between 1951 and 2020, grouped by European region. The number of suitable months was calculated as the number of months per year with precipitation more than 80 mm, average temperature between 14·5°C and 33°C, and relative humidity more than 60%, in land types highly suitable for *Anopheles* mosquitoes. Linear regression was used to estimate trends (A–C). R0=reproduction rate.

West Nile virus is a climate-sensitive multi-host and multi-vector pathogen. Human infection is associated with severe disease risk and death.^37^ In the past few decades, European countries have had a large increase in the intensity, frequency, and geographical expansion of West Nile virus outbreaks.^37^, ^38^ The 2018 outbreak has been the largest yet, with 11 European countries reporting 1584 locally acquired infections.^39^ Increasing ambient temperatures are increasing the vectorial capacity of the *Culex* mosquito vector, and thus increasing the outbreak probability.^40^, ^41^ With machine learning models that incorporate reported West Nile virus cases and climate variables (temperature, precipitation), a steady and accelerating trend of West Nile virus outbreak risk were estimated to be driven by climate factors between 1951 and 2020.^42^ Comparing 1951–85 with 1986–2020, the largest increases in West Nile virus outbreak risk was in northern Europe (445%) and western Europe (242%). However, absolute risk for West Nile virus outbreaks remain highest in southern and central and eastern Europe, with risk increases of 149% in southern Europe and 163% in central and eastern Europe in 1986–2020 compared with 1951–1985 (figure 3A; appendix pp 46–49).

#### Indicator 1.3.3: dengue

Accelerated human mobility and increasing climate suitability for arboviral disease transmission are increasing the emergence of arboviral diseases in Europe.^43^, ^44^, ^45^ In the past 5 years, sporadic autochthonous dengue outbreaks have been reported in Spain and France.^46^ Without sufficient preparedness, dengue outbreaks can lead to severe health risks and impact on society.^47^

This indicator uses a mechanistic model to estimate the basic reproduction rate (*R*_0_) and length of transmission season for dengue combining information on temperature, rainfall, mosquito abundance, and human population density.^48^, ^49^ Overall, in the period 1986–2020, (*R*_0_) has increased by 17·3% in Europe compared with 1951–1985 (figure 3B). This pattern is also observed for chikungunya and Zika virus. The greatest upward shift in transmission season is observed in central eastern Europe, with a gain of about 0·2 suitable months for dengue (appendix pp 50–54).

A sub-indicator is also included, monitoring the estimated rates of imported dengue cases to European regions by estimating the annual number of people infected with dengue moving from dengue-endemic regions around the world (yearly incidence of at least 0·5% of the region's population), into locations in Europe where conditions are suitable for dengue transmission, as defined by transmission season *R*_0_ levels higher than 1 for at least 1 month. Between 1990 and 2019, the number of estimated imported cases per NUTS 3 region increased by 600% within areas of Europe showing climate suitability for dengue transmission (figure 3B; appendix pp 55–76).

#### Indicator 1.3.4: malaria

Although Europe has been malaria-free since 1974,^50^ cases have been reported by travellers and as part of sporadic local transmission events.^51^ Monitoring climate suitability for malaria transmission, having early warning systems, and preparing health-care systems are therefore imperative to prevent the re-emergence of malaria in Europe. This indicator uses a threshold-based model to capture the number of months in which the accumulated precipitation, relative humidity, and temperature, combined with land cover type, make environmental conditions suitable for the transmission of *Plasmodium vivax*, the main malaria pathogen in Europe. Overall, the indicator showed a 4·5% increase in the number of months suitable for *P vivax* transmission, when comparing 1986–2020 with 1951–85. Although the number of suitable months increased in all European regions, the highest change was in northern and western Europe, with a 21·6% increase in suitability between 1951 and 1985 and a 25·2% increase between 1986 and 2020 (figure 3C; appendix p 77–82).

### Indicator 1.4: allergens

#### Indicator 1.4.1: allergenic trees

**Figure 4:**
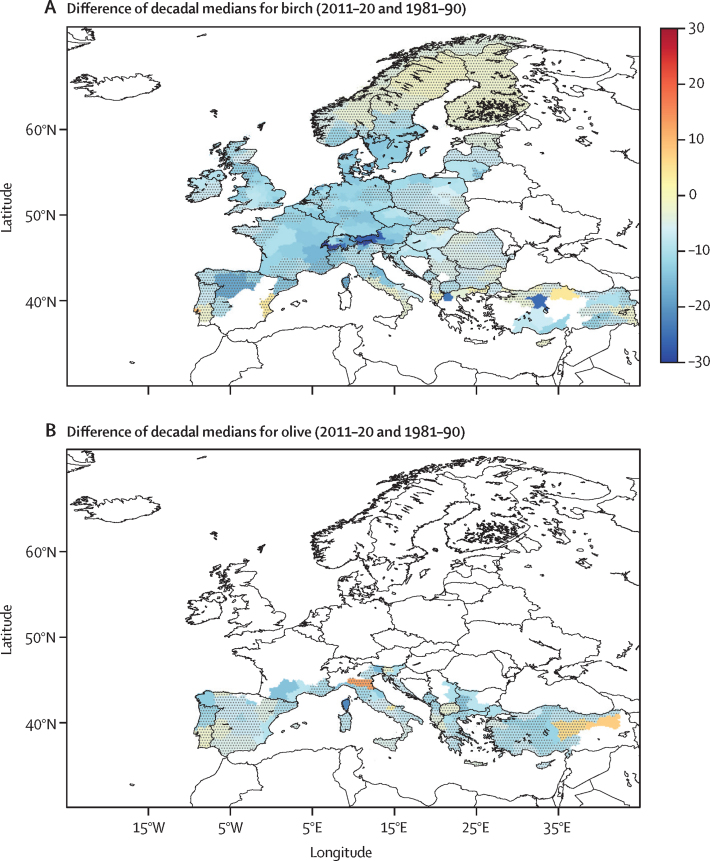
Difference of decadal medians (days) in the start of clinically relevant pollen seasons in Europe Change of decadal medians in start of clinically relevant pollen season (days) for (A) birch and (B) olive in Europe at NUTS level 2, comparing 2011–20 with 1981–90. Dot-shaded areas have statistically insignificant trends (p value >0·1). White areas (without shade) had clinically relevant seasons that occurred less than 5 times between 1981 and 1990 or 2011 and 2020.

Natural allergenic aerosols, such as pollen, are released by plants during their flowering season.^52^ Pollen proteins can exacerbate allergic rhinoconjunctivitis (pollinosis) and allergic asthma by acting as antigens for the immune system.^53^ The changing climate is associated with shifts in flowering seasons of most plants, which leads to changes in seasonal pollen allergies.^52^ This indicator monitors the temperature-induced changes of the start of the clinically relevant pollen season (ie, when concentrations of the specific pollen are high enough to cause allergy symptoms) for three types of trees (birch, alder, and olive) between 1981 and 2020, by estimating changes in the start of their flowering season and combining this with atmospheric models to estimate pollen air concentrations.^54^, ^55^, ^56^, ^57^, ^58^ Increasing temperatures during 1981–2020 have been associated with flowering seasons for the three tree species starting 10–20 days earlier. The most substantial changes occurred at high altitudes (ie, the Alps, Balkans, and Scandinavian mountains), in which the flowering season now starts on average more than 1 month earlier than it did 40 years ago (figure 4; appendix pp 83–90).

### Conclusion

The health-related hazards, exposures, vulnerabilities, and risks from climate change are showing clear, accelerating trends in Europe, but with heterogeneous geographical patterns. Although some of the relative changes are largest in northern Europe, many of the indicators show highest absolute risks or climate suitability in central and southern Europe. The heat-related indicators (indicators 1.1.1 to 1.1.4) showed substantial changes in heat exposure, the ability to safely exercise, and in heat-related mortality. The frequency of extreme drought events in regions affected by drought has increased in the past decade (indicator 1.2.2). The infectious disease indicators (indicators 1.3.1 to 1.3.4) show rapidly escalating climate suitability for water-borne and vector-borne diseases, and clinically relevant pollen seasons are starting earlier each year in Europe (indicator 1.4). These changes show the urgent need for adaptation and mitigation actions.

## Section 2: adaptation, planning, and resilience for health

The COVID-19 pandemic and the increasing climate-change-driven health risks outlined in section 1 emphasise the need to protect populations from increasing health shocks.^59^ Considering the short-term and long-term impacts of climate change on public health, adaptive capacity and interactive management provide an opportunity to create environmentally sustainable, climate resilient health systems that reduce the current and future health impacts of climate change (section 1), while reducing the risk of future pandemics.^60^

The essential functions of public health can be grouped into health assessment, policy development, resource allocation, and access to services.^61^ These functions include preparedness, under which multi-hazard national public health emergency preparedness and response plans can be developed and implemented. However, the adaptive capacity of health systems to climate change also calls for early warning systems (panel 1) and projections of future risk scenarios. Thus, health systems would benefit from long-term planning, accounting for both current and future impacts of climate change. Health adaptation requires a multisectoral approach, involving various health and government authorities, private sector entities, and civil society. In February, 2021, the European Commission adopted a new adaptation strategy on the basis of four main pillars: improving the evidence base for adaptation measures; accelerating the roll-out of adaptation solutions; mainstreaming the integration of adaptation across sectors; and increasing international action for climate resilience.^62^ However, monitoring the implementation and health co-benefits of adaptation measures is hindered by the scarcity of quantitative measures and objective indicators for climate change adaptation.

Panel 1 **The 2022 Europe Lancet Countdown report indicators**

**Climate change impacts, exposures, and vulnerabilities**

1.1 Heat and health
  1.1.1 Vulnerability to heat exposure
  1.1.2 Exposure of vulnerable populations to heatwaves
  1.1.3 Physical activity related heat stress risk
  1.1.4 Heat-related mortality
1.2 Extreme events and health
  1.2.1 Wildfire smoke
  1.2.2 Drought
1.3 Climate-sensitive infectious diseases
  1.3.1 Climate suitability of non-*cholerae* Vibrio
  1.3.2 Climate suitability of West Nile virus
  1.3.3 Climate suitability of dengue
  1.3.4 Climate suitability of malaria
1.4 Allergens
  1.4.1 Allergenic trees

**Adaptation, planning and resilience for health**

2.1 Adaptation planning and assessment
  2.1.1 National assessments of climate change impacts, vulnerability, and adaptation for health
  2.1.2 National adaptation plans for health
  2.1.3 City-level climate change risk assessments
2.2 Adaptation delivery and implementation
  2.2.1 Climate information for health
  2.2.2 Exposure to green space
  2.2.3 Air conditioning benefits and harms

**Mitigation actions and health co-benefits**

3.1 Energy system and health
  3.1.1 Carbon intensity of the energy system
  3.1.2 Coal phase-out
  3.1.3 Renewable and zero-carbon-emissions energy
3.2 Premature mortality attributable to ambient fine particles
3.3 Sustainable and healthy transport
3.4 Food, agriculture, and health
  3.4.1 Life cycle emissions from food demand
  3.4.2 Sustainable diets

**Economics and finance**

4.1 Health-linked economic impacts and mitigation of climate change
  4.1.1 Economic losses due to climate-related extreme events
  4.1.2 Heat impacts on labour supply
  4.1.3 Impact of heat on economic activity
  4.1.4 Monetised value of unhealthy diets
4.2 Economics of the transition to zero-carbon economies
  4.2.1 Net value of fossil fuel subsidies and carbon prices

**Politics and governance**

5.1 Coverage of health and climate change in scientific journals
5.2 Individual engagement with health and climate change on social media
5.3 Political engagement with health and climate change
5.4 Corporate sector engagement with health and climate change

This section explores health adaptation planning and assessment (indicators 2.1.1 to 2.1.3) and adaptation delivery and implementation (indicators 2.2.1 to 2.2.3). Because the provision of information services is one of the crucial steps in health adaptation on climate change, panel 2 outlines current operationalised early warning systems in Europe.

Panel 2 **Operationalised early warning systems for climate adaptation in Europe**

Early warning systems are an integral part of climate change adaptation and disaster risk reduction, intended to reduce the impact of hazards on public health and society at large. The process involves detection, analysis, prediction, and warnings to trigger a response by authorities and community members. Operationalised early warning systems entail detailed knowledge of the exposure–response curve, a monitoring system of climatic or environmental precursors of disease, communication and dissemination of an alert, and the capacity to respond. Harnessing the rapidly improving capability to predict seasonal climate patterns allows for increased confidence in early warning systems for public health purposes. By incorporating forecasts of relevant atmospheric or environmental indicators in health early warning systems, pre-emptive actions can be initiated. Many early warning systems have been developed and operationalised by the Joint Research Centre at the European Commission, with earth observations from the Copernicus Programme, designed to protect health and wellbeing from floods, droughts, wildfires, and infectious diseases. A market analysis of the Copernicus investments in climate, marine, atmosphere, emergency, and security shows a doubling of the returns in economic and social benefits.^63^

**The European flood awareness system** ^64^

The European flood awareness system is the first operational early warning system to monitor and forecast floods in Europe. Flood notifications are sent to hydrometeorological authorities depending on whether the forecast predicts a specific probability to exceed a pre-defined threshold and complement national monitoring systems. A cost–benefit analysis showed the monetary saving of such a cross-border continental-scale early flood warning system.^65^

The system produces several outputs for regional and national authorities, including:

• Flash flood forecasts with up to 5-day advance warnings, based on high-resolution numerical weather predictions and 6-hour radar-based precipitation monitoring
• An overview of upcoming flood events for the next 10 days, including possible flood impacts; these medium-range flood forecasts are updated twice a day
• Outlooks of the hydrological situation in the next 6 to 8 weeks as part of a sub-seasonal forecast (issued twice a week) and seasonal forecast (issued once a month); predictions of hydrological extremes of high and low flows can be used for reservoir management, navigation, irrigation, or drought risk management

**European Drought Observatory (EDO)**

EDO provides continuously updated indicators relevant to drought that are based on remotely sensed and in-situ data and hydrometeorological models. The input data include precipitation, soil moisture, reservoir levels, river flow, groundwater levels, and vegetation water stress. Key drought outputs provided by the EDO include:

• Combined Drought Indicator for monitoring agricultural and ecosystem drought
• Standardised Precipitation Index for monitoring meteorological drought
• Soil moisture and vegetation greenness for monitoring agricultural drought
• Low flow in main rivers and groundwater for monitoring hydrological drought
• Forecasts of extreme precipitation and soil moisture anomalies
• Daily temperature anomalies and heatwaves

These outputs are complemented by indicators of regional and local relevance (eg, river basin) provided by a network of partners.

**The European Forest Fire Information System**

The European Forest Fire Information System monitors forest fire activity in near-real time and provides information on the current and future fire danger forecast, active fires and burned areas, postfire damage assessments, and their ecological impacts in the European region. The system reports on a daily, monthly, and seasonal basis, and includes components starting from pre-fire state, including:

• Fire Danger Forecast
• Active fire Detection
• Rapid Damage Assessment
• Fire damage assessment
• European Fire Database

**The European Centre for Disease Prevention and Control (ECDC)** Vibrio **Map Viewer**^36^

The ECDC *Vibrio* Map Viewer provides environmental suitability maps of coastal areas globally at risk for *Vibrio* infections. The infections are caused by marine bacteria that can result in severe wound infections, sepsis, or gastroenteritis. As these *Vibrio* infections are not a notifiable disease in Europe, the ECDC *Vibrio* Map Viewer can be used as an environmental monitoring tool instead. The tool is based on a real-time model with remotely sensed sea surface temperature and salinity that has been calibrated to the Baltic region in northern Europe. The model generates a daily map and 5-day forecasts of the environmental suitability categorised as low, very low, medium, high, and very high. The findings are reported once a week in the ECDC Communicable Disease Threats Report. The report is distributed to national state epidemiologists in Europe and discusses options for public health prevention and control actions. The control actions include issuing alerts, notifying health-care providers, and encouraging individuals at risk to avoid recreational water use in those areas.

### Indicator 2.1: adaptation planning and assessment

#### Indicator 2.1.1: national assessments of climate change impacts, vulnerability, and adaptation for health

The health impacts of climate change vary by geographical location and population. Location-specific vulnerability and adaptation assessments are an essential first step for identifying, formulating, and implementing national health and climate change adaptation plans.^66^ This indicator uses data self-reported by countries in the WHO Health and Climate Change Country Profile survey to assess whether countries have completed a climate change and health vulnerability and adaptation assessment (appendix pp 91–94). In 2021, 10 (45%) of 22 countries reported doing a vulnerability and adaptation assessment. Of these, only Germany reported that the vulnerability and adaptation assessment strongly influenced the allocation of human and financial resources. In other countries, the influence was moderate or minimal.

#### Indicator 2.1.2: national adaptation plans for health

An EEA report published in 2022 indicated that, of 37 national adaptation strategies and 34 national health strategies, most national strategies made reference to the physical health impacts of climate change, whereas mental health impacts were mentioned less often.^67^

In the WHO Health and Climate Change Country Profile survey from 2021, 15 (68%) of 22 participating European countries indicated having national health and climate change strategies or plans in place (appendix pp 91–94). 8 (36%) of the 22 countries reported having moderate or low levels of implementation, with only 3 (14%) countries reporting very high implementation. 14 (64%) countries reported having an operational multi-stakeholder mechanism on health and climate change, 9 (41%) reported participation of national meteorological and hydrological services in these multi-stakeholder mechanisms, and 15 (68%) countries reported having a designated point of contact or team responsible for health and climate change at their ministries of health.

#### Indicator 2.1.3: city-level climate change risk assessments

With 75% of the population in Europe living in urban centres, city-level climate change risk assessments can provide crucial information for the development and design of city-level adaptation and mitigation. With data reported to the Carbon Disclosure Project and International Council for Local Environmental Initiatives (appendix p 95), this indicator shows that 150 (76%) of 197 European cities in 2021 reported that a climate assessment had been done, 17 (8·6%) reported that an assessment was in progress, and 18 (9·2%) reported that an assessment would be done in the next 2 years. 118 (59·9%) of the 197 cities reported that climate change posed a risk to health services or public health. Heat-related illnesses were identified most prominently as a climate-related health hazard (identified by 87 cities), followed by air-pollution-related illnesses (identified by 68 cities), direct physical injuries and death due to extreme weather events (identified by 45 cities), and exacerbation of non-communicable disease symptoms (identified by 45 cities). Older people, children, youth, and people with pre-existing medical conditions were identified as the most vulnerable. 82 (42%) cities reported doing specific health-related risk and vulnerability assessments.

### Indicator 2.2: adaptation delivery and implementation

#### Indicator 2.2.1: climate information for health

To adequately prepare and respond to climate health hazards, health systems should have access to climate information. Based on the 2021 WHO Health and Climate Change Country Profile survey, 10 (45%) of 22 countries in the WHO European region reported having climate-informed health surveillance systems (ie, health systems that include meteorological information) for heat-related illnesses, 8 (36%) reported systems for injury and mortality related to extreme climatic events, 6 (27%) had systems for vector-borne diseases, and 4 (18%) had systems for water-borne diseases (appendix pp 91–94). 10 (45%) of the 22 countries reported having formal agreements on health and climate change policy between the ministry of health and the national meteorological and hydrological services.

#### Indicator 2.2.2: exposure to green space

Buildings, roads, and other infrastructure absorb and re-emit the sun's heat more than natural landscapes, such as forests and water. This process creates urban heat islands, which have higher temperatures than outlying rural areas. To minimise this effect, both the EU Adaptation Strategy and the EU Biodiversity Strategy for 2030 emphasise the need for cities to create biodiverse and accessible urban green spaces, including parks, forests, and tree-lined streets. Urban greenness provides physical and mental health benefits; promoting physical activity, reducing stress, improving air quality, and reducing the heat island effect. Thus, increasing urban green space is part of nature-based adaptation solutions with economic and social co-benefits.

**Figure 5:**
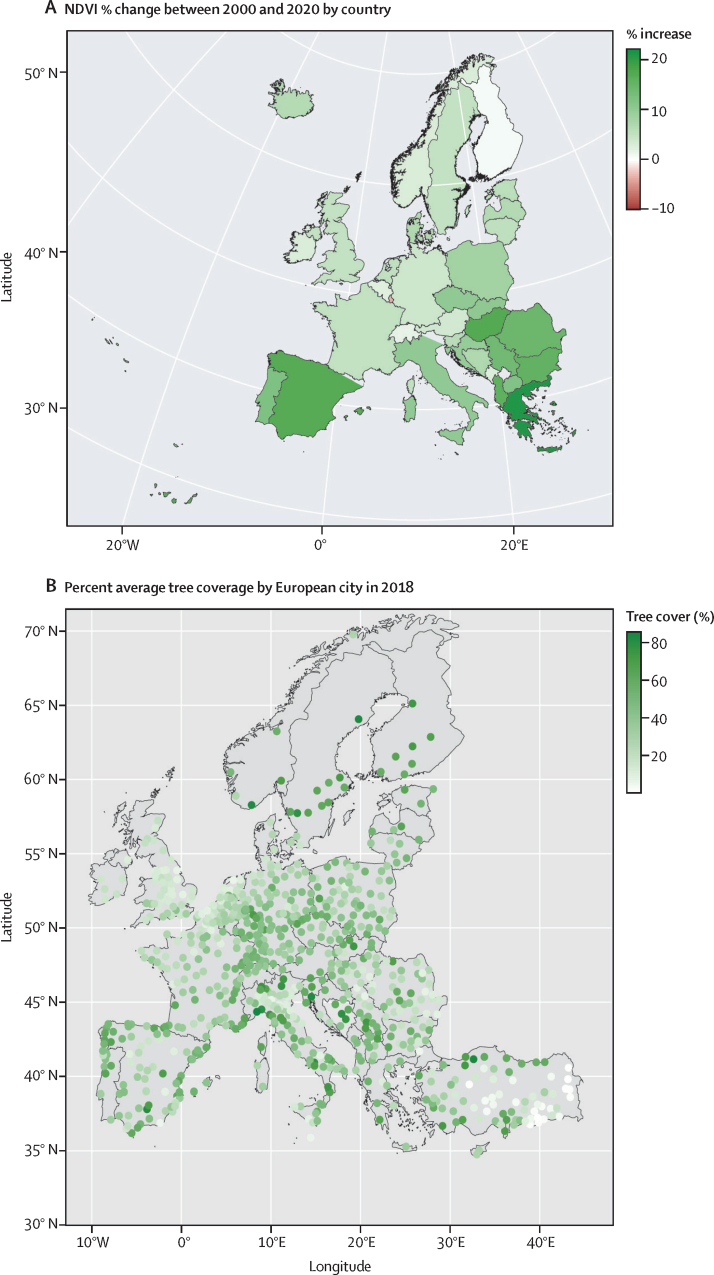
Green space exposure in Europe (A) Change of the NDVI in Europe by country comparing 2020 with 2000. (B) Percent average tree coverage by European city in 2018. NDVI=normalised difference vegetation index.

This indicator includes two components, the first one tracking exposure to green space, measured by the population-weighted normalised difference vegetation index at the country level. Populated-weighted greenness increased during 2000–20 in most European countries, with the largest percentage increases taking place in southern Europe (13% mean increase) and the smallest increases in western Europe (3% mean increase; figure 5A). The three countries with the largest increase were Greece (21%), North Macedonia (17%), and Albania (17%). Despite not having the smallest absolute exposure to green space, countries with the smallest increase in green space exposure were Switzerland (1%), Finland (0%), and Luxemburg (2% decrease). Changes in the indicator during 2000–2020 were largely explained by increasing normalised difference vegetation index over time, rather than population change (appendix pp 96–97).

The second part of this indicator monitors urban tree coverage. The mean urban tree coverage in European cities (38 EEA countries plus the UK) in 2018 was 28·5% when including cities with their commuting zone. At a city level, Savona, Italy has the highest proportion of urban tree coverage (83%), whereas the City of London, UK has the lowest (<1%; figure 5B; appendix pp 98–99). The commuting zone of a city usually has a higher tree coverage compared with the city centre, although there are exceptions (eg, Miskolc, Hungary). Urban tree cover varies widely across cities and their surrounding areas in Europe, which requires tailored urban greening strategies to ensure that biodiversity, accessibility, and climate resilience objectives are met in all urban communities.^68^

#### Indicator 2.2.3: air conditioning benefits and harms

Air-conditioning is one of the most protective factors for heat-related morbidity and mortality.^69^ However, access to air conditioning is unequal—with 50–125 million people unable to afford indoor thermal comfort in Europe, marginalised and poor populations are exposed to increased risk of heat exposure, exacerbating health inequalities.^70^ Air conditioning contributes to further greenhouse gas emissions, air pollution, peak electricity demand, and the urban heat island effect.

This indicator tracks the proportion of European households using air conditioning and the CO_2_ emissions from air conditioning between 2000 and 2019. For most European countries assessed, the proportion of households using air conditioning has increased steadily. Of the countries for which we have individual-level country data, the highest increase was observed in Finland (162%) and Germany (122%; appendix p 100). Although Italy had the highest proportion of households using air conditioning between 2000 and 2019 (40% of households in 2019), the country observed a 2% decrease in the proportion of air conditioning use in 2019 compared with 2000. Despite CO_2_ emissions from air conditioning use decreasing for most European countries in the period 2000–19, CO_2_ emissions from air conditioning still reached 9·1 megatonnes (Mt) in Italy, 2·7 Mt in the UK, and 2·5 Mt in Germany (appendix p 100).

Although countries in Europe use comparatively less air conditioning than high-income countries in other parts of the world (eg, the USA and Australia), the over-reliance on energy-intensive air conditioning can increase the health risks of energy poverty, further increase greenhouse gas emissions, and undermine the introduction of more sustainable cooling solutions (eg, nocturnal radiation cooling, geothermal cooling, ventilation, evaporative cooling, and district cooling).^71^, ^72^, ^73^, ^74^ Implementing energy-efficient, environmentally sustainable thermal comfort techniques, while minimising the over-reliance on energy intensive technologies over the use of other cooling interventions that are comparatively efficient, is essential to protect European populations from the increased health risks of rising temperatures.^71^, ^72^

### Conclusion

Although 15 (68%) of 22 European countries participating in the WHO survey have national adaptation plans in place (indicator 2.1.2), the enactment of these plans is not sufficient to advance adaptive capacity and to ensure the translation into adequate adaptation actions. The indicators presented in this section support the monitoring of adaptation actions across different sectors to find synergies between adaptation and health co-benefits. For example, indicator 2.2.2 tracks the proportion of cities covered in tree canopy, which could attenuate the impact of the heat island effect in the long term. Several national and regional civil protection authorities are using early warning systems to monitor the risk of forest fires, drought, or flood, offering the opportunity to adapt and develop new early warning systems to protect public health from climate change impacts.

Overall, the indicators in this section suggest some positive trends, with increasing exposure to green space (indicator 2.2.2), countries and cities implementing adaptation plans for health (indicator 2.1.2), the use of climate-informed health surveillance systems (indicator 2.2.1), and the development of climate change and health risks assessments (indicators 2.1.1 and indicator 2.2.1). Nevertheless, climate change adaptation remains neglected, competing for financial resources with other issues such as the response to the COVID-19 pandemic or the war in Ukraine. Building resilient health systems will be essential to confront the predicaments of our times.

## Section 3: mitigation actions and health co-benefits

Although the European region (EEA 38) is only 8% of the global population, Europe is responsible for roughly 11% of global CO_2_emissions from fossil fuels and 11% of global CO_2_ eq emissions from food demand.^75^ Greenhouse gas emissions from the EU account for 17% of global cumulative greenhouse gas emissions (1950–2020), making Europe one of the major contributors to the climate crisis, and placing the lives and health of hundreds of millions of people at risk globally.^4^ Importantly, these emissions affect the world population unequally. Countries and populations with the lowest emission contributions are disproportionately impacted by the effects of climate change, exacerbating entrenched between-country and within-country inequalities.^76^ The 2022 Intergovernmental Panel on Climate Change report^77^ highlighted the urgency of climate action and exposed how dangerously close the world is to missing the goals of achieving a safer warming of 1·5°C higher than pre-industrial levels. With little progress in the past years, the need for accelerated mitigation in Europe is as urgent as ever.

The European Climate Law entered into force in July, 2021,^78^ providing the legal basis for the European Green Deal objective to reach net-zero greenhouse gas emissions by 2050. This law increases ambitions to a 55% reduction in greenhouse gas emissions by 2030 compared with emission levels in 1990. When health and wellbeing are prioritised, meeting these targets could deliver multiple co-benefits, including improved energy security, the creation of green jobs, and improved population health with cleaner air, more plant-based diets, increased physical activity, and healthy cities. Yet, thus far, the nationally determined contributions of all European countries would not fulfil the Paris Agreement.^79^, ^80^

This section tracks European efforts to mitigate climate change and their associated health co-benefits from the reduction of ambient air pollution and transition to more sustainable and healthy forms of travel and diets. Indicators fall within four domains: the energy system and health (indicators 3.1.1 to 3.1.3), mortality impacts of ambient air pollution from fossil fuel use (indicator 3.2), sustainable and healthy transport (indicator 3.3), and food, agriculture, and health (indicators 3.4.1 and 3.4.2).

### Indicator 3.1: energy system and health

#### Indicator 3.1.1: carbon intensity of the energy system

Fossil fuel combustion accounts for around 65% of global greenhouse gas emissions.^75^ Between 1990 and 2005, emissions from fuel in Europe were at 4·3 gigatonnes (Gt)CO_2_ per year (7·3 tCO_2_ per person). However, by 2019, emissions had fallen by 14% compared with 2005, to 3·5 GtCO_2_ per year (5·6 tCO_2_per person). Comparatively, per person, emissions in Europe in 2019 were 1·3 times global average per person emissions, more than three times the emissions in south Asia, and more than seven times African emissions.^4^ Likewise, there are substantial differences in the contributions per person from different countries across Europe: in 2019, the average contribution per person was 7·7 tCO_2_ in Germany and 1·4 tCO_2_ in Albania. Germany's carbon emissions from fuel combustion in 2020 accounted for 18% of the total (3·2 GtCO_2_) for Europe, followed by Türkiye (10%), the UK (10%), and Poland (8%).

The COVID-19 pandemic temporarily reduced Europe's emissions by 8% from 2019 to 3·2 GtCO_2_ per year (5·1 tCO_2_ per person) in 2020; however the global emission reductions in 2020 are unlikely to be detectable in the growth rate of CO_2_ in the atmosphere.^75^

**Figure 6:**
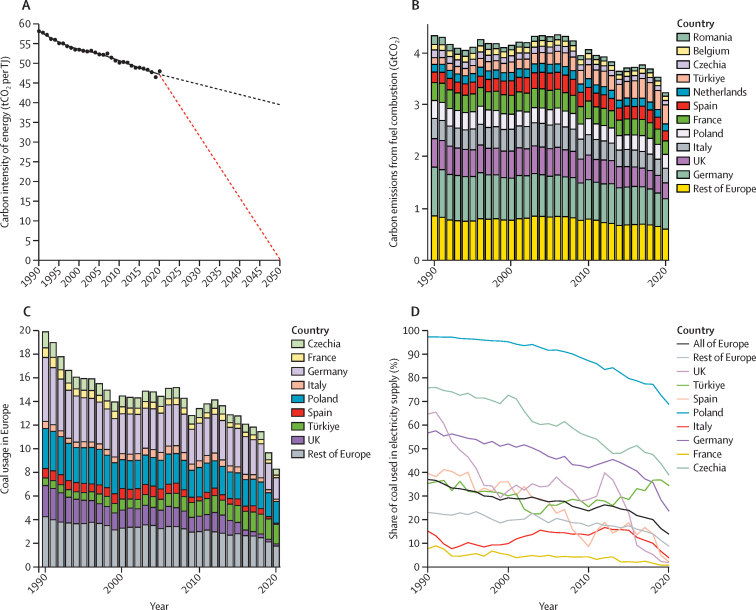
Carbon intensity of the energy system and coal phase-out in Europe (A) Carbon intensity of total energy supply in Europe from 1990 to 2020. Red dashed line shows the rate of reduction required to meet net-zero by 2050. Black dashed lines show extrapolation of current rate of reduction. (B) Carbon emissions from the combustion of fuels from 1990 to 2020 for countries with emissions greater than 0·1 GtCO_2_ per year. (C) Coal use in Europe from 1990 to 2020 by country. (D) Share of electricity (%) generated by coal combustion in Europe from 1990 to 2020 by country.

The carbon intensity of the energy system in Europe (ie, the amount of greenhouse gas emissions per unit of total energy supply) has decreased by 8% in the past 15 years, with an annual rate of change of –0·5% per year. To reach net-zero CO_2_ emissions by 2050, the European energy system should decarbonise at five times the current pace (figure 6A, B; appendix p 101).

The volatility of energy prices in Europe in 2020–22, and the energy security threats related to the Russian invasion of Ukraine, highlight the co-benefits of accelerated transition to renewable energies in terms of increased energy security and energy system diversification and resilience.^81^

#### Indicator 3.1.2: coal phase-out

Coal has the highest carbon intensity of all fuels^82^ and is responsible for 16% of particulate matter concentrations in Europe, an important contributor to premature mortality (indicator 3.2). Since 1991, coal use has decreased in Europe by around 56%. However, coal use still contributes to 12% of total European energy supply in 2020, and rates of reduction are incompatible with net-zero targets. Despite some progress, the share of electricity generated from coal in countries such as Germany (25%) and Czech Republic (40%) was high in 2020 (figure 6C, D; appendix p 102). Türkiye is the only country in Europe in which coal use for electricity generation has increased since 2010. To limit the global temperature rise to 1·5°C, all European countries should phase out coal by 2030.^83^

#### Indicator 3.1.3: renewable and zero-carbon-emissions energy

Increasing the share of Europe's energy supply from zero-carbon energy sources is crucial to meeting the targets of the European Climate Law and Paris Agreement. Although the share of electricity produced from renewable sources was just 17% in 2020, the electricity system in Europe could reach zero-carbon by 2032, if the current annual rate of increase of 16% is maintained (appendix pp 103–04). Yet, electricity is only 18% of Europe's total energy use, with the remainder largely used for heating and transport (most of which is supplied by fossil sources; indicator 3.3.2).^84^ The energy supply from zero-carbon sources in Europe was only 21% in 2020, and Europe's primary challenge is to decarbonise heating, accounting for around half of total energy demand in Europe.

### Indicator 3.2: premature mortality attributable to ambient fine particles

Exposure to PM_2·5_ is an important environmental risk factor for premature mortality. Activities that contribute to greenhouse gas emissions, including the burning of fossil fuels and waste, contribute to dangerous levels of human exposure. This indicator tracks changes in premature mortality attributable to PM_2·5_ from the combustion of coal, liquid fuels, and gaseous fuels across different economic sectors (appendix pp 105–07).

Stringent air pollution emission controls (eg, for electricity generation, industrial emissions, and agricultural practices) have resulted in reduced PM_2·5_-related mortality in Europe since 2005. Yet, despite improvements, this indicator estimates that 94% of the European population live at PM_2·5_ concentrations higher than the new WHO guideline^85^ amount (5 μg/m^3^ annual mean). Total population-weighted mean ambient PM_2·5_ concentrations in Europe are approximately 10 μg/m^3^, with the combustion of fossil fuels being directly responsible for 36% of population-weighted average PM_2·5_. Combined, approximately 117 000 deaths (about 2% of all deaths) were attributed to combustion of fossil fuels in 2020, in Europe, a decrease of 11 700 (60%) from 29 300 in 2005. As a result of coal phase-down, the annual deaths attributable to PM_2·5_ from coal-fired power plants decreased from 103 000 deaths annually in 2005, to 23 000 deaths in 2020. However, mortality levels associated with household coal use were stable at 29 000 deaths per year between 2005 and 2020. Of the PM_2·5_ caused by fossil fuel combustion, transport is the main sector responsible for 48 000 deaths in 2020. Reductions in this sector were primarily due to air pollution emissions control technology, with limited or no benefits for greenhouse gas emission reduction.^86^

### Indicator 3.3: sustainable and healthy transport

Liquid fossil fuel combustion in road transportation was responsible for 72% of transport-related greenhouse gas emissions in Europe in 2019, and is a major contributor to air-pollution-related deaths in Europe (indicator 3.2). Switching to public or active forms of travel can not only help to reduce these emissions,^87^ but also contribute to reduced noise pollution and increased physical activity, leading to improved overall health outcomes.^88^, ^89^

This indicator is based on data from the International Energy Agency to monitor the use of fossil fuels for road transportation. Data indicate that fossil fuel use per person in road transport peaked in 2007 (appendix pp 108–09), although improved vehicle efficiency, rather than changes in transport modes, accounts for much of this reduction. Increases in the past 20 years of vehicle ownership per person have seen transport energy use increase in several countries, notably Germany and Denmark.

The current EU target aims for all new cars and vans to be emission-free by 2035.^90^ However, despite increased uptake of electric vehicles in the past 5 years, electricity accounted for less than 0·1% of the energy used for road transport in Europe in 2019, and many forms of public transport are not electric and still use diesel. According to EEA mode-share data (appendix pp 108–09), median use of trains and buses was less than 17% of journeys.

These findings highlight the potential for environmental and health gains with transport policies, particularly by increasing active travel (ie, walking or cycling).

### Indicator 3.4: food, agriculture, and health

#### Indicator 3.4.1: life cycle emissions from food demand

**Figure 7:**
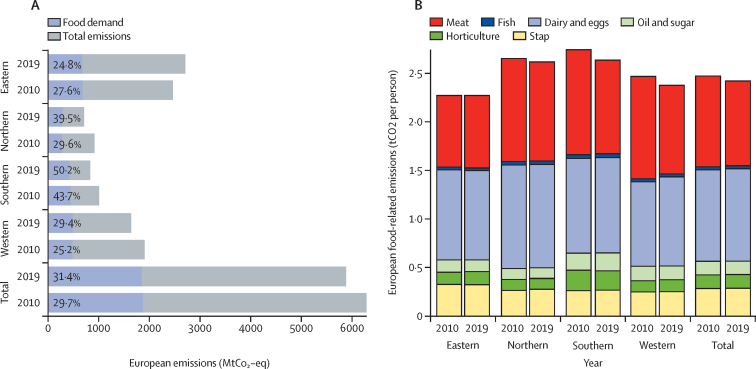
Life cycle emissions from food demand in Europe (A) Greenhouse gas emissions from food demand as a proportion of total territorial emissions (%) by European region in absolute terms (2010 and 2019). (B) Food-related CO_2_ emissions per person by European region and food group (2010 and 2019).

Mitigation in the agricultural sector has big decarbonisation potential and can lead to improved health outcomes from healthy, more plant-based diets. This indicator merges data from the Food and Agriculture Organization with lifecycle-emission estimates to report on the greenhouse gas emissions associated with food consumption. In 2019, food demand from Europe was responsible for 1·85 GtCO_2_ eq (2·47 tCO_2_ eq per person); corresponding to 31% of all European greenhouse gas emissions, with animal-based foods responsible for 77% of emissions (1·9 tCO_2_ eq per person). Southern Europe had the highest emissions per person (2·74 tCO_2_ eq) and eastern Europe the lowest (2·28 tCO_2_ eq). From 2010 to 2019, food-related emissions in Europe reduced by only 1% (20 Mt CO_2_ eq), with greatest reductions in southern Europe (–5%), then central and eastern Europe (–1%), and increases in western Europe (+0·3%) and northern Europe (+4%) (figure 7; appendix p 110).

#### Indicator 3.4.2: sustainable diets

Dietary changes leading to more plant-based food consumption is essential for decarbonisation in the agricultural sector.^91^ These changes could result in substantial reductions of diet-related morbidity and mortality.^92^ The EAT-*Lancet* Commission put forward a diet ensuring human health within planetary boundaries; diets that largely consist of vegetables, fruits, whole grains, legumes, nuts, and unsaturated oils; include low to moderate seafood and poultry; and include no or a low quantity of red meat, processed meat, added sugar, refined grains, and starchy vegetables.^93^

This indicator models the number of deaths attributable to dietary factors by merging estimates of food consumption with epidemiological models. According to these data, in 2019, 2·2 million deaths (27%) of 8·3 million total deaths among adults were attributable to imbalanced, high-carbon diets in Europe (appendix pp 111–17). 1·2 million (55%) of the 2·2 million deaths were attributable to the composition of diets, including 174 000 (8%) of 2·2 million deaths to excessive red meat consumption. Eastern Europe had the greatest relative health burden associated with poor diets, including having the greatest absolute health burden associated with a high intake of red meat (80 000, 46% of deaths attributable to red meat across Europe). Progress in reducing diet-related mortality has been slow, decreasing from 28% to 27% of all deaths from 2010 to 2019. The greatest reductions were in western Europe (2 percentage points) and central and eastern Europe (2 percentage points).

### Conclusion

Indicators in this section highlight notable progress in reducing the carbon intensity of the energy system, phasing out coal for electricity generation (indicators 3.1.1 to 3.1.3), and reducing deaths attributable to air pollution in Europe (indicator 3.2). However, decarbonisation efforts have been insufficient to meet the goals in the European Climate Law^78^ and Paris Agreement. The current pace of reduction in fossil fuel use for electricity generation, residential heating, and transport are incompatible with reaching net-zero greenhouse gas emissions by 2050 (indicators 3.1.1 to 3.1.3 and 3.3) and do not support efforts to meet WHO guidelines^94^ for safe ambient PM_2·5_ concentrations (indicator 3.2). Progress towards adopting healthy and more plant-based diets has also been slow and has led to unnecessary greenhouse gas emissions and mortality associated with excess red meat consumption (indicators 3.4.1 and 3.4.2).

Promoting health should be a top priority in guiding the specific measures implemented to meet mitigation goals, to fully realise health co-benefits of mitigation, and to avoid unintended health risks that can occur when health considerations are not adequately integrated in mitigation actions. Salient examples in past decades of efforts to reduce CO_2_ emissions in Europe without adequate consideration of health include promoting diesel over gasoline-powered vehicles, on the basis of better fuel-efficiency and the promotion of biomass for residential heating, both of which have resulted in considerable emissions of health-damaging air pollutants. However, well planned mitigation strategies that appropriately integrate health will tremendously benefit both the climate and public health. With the world dangerously close to reaching climate-driven points of no return, the opportunity to deliver climate action in line with the Paris Agreement and improved health outcomes cannot be missed.^77^

## Section 4: economics and finance

Both the drivers of climate change and climate change-related health impacts have a profound effect on European and global economies.^5^ However, the economic benefits of transforming to low-carbon economies outweigh the costs of inaction, which potentially include losses amounting to 11% of global GDP by 2050,^95^, ^96^, ^97^ and a wide range of negative health impacts (section 1). Accelerating commitment to climate change mitigation will likely prevent detrimental economic impacts, adding further health co-benefits by safeguarding the socioeconomic determinants of health.

Globally, the EU is the third largest economy (representing 16% of the world's GDP), provides the highest share of climate finance for low-income countries,^98^ and has allocated 30% of the 2021–27 EU budget to achieving mitigation targets.^99^ This proposed allocation is expected to support actions that contribute to the global objective of decarbonisation and local objectives of reduced costs from climate change, including the costs related to human health.

Importantly, the potential health co-benefits of reduced air pollution due to mitigation policies alone range from 7% to 84% of mitigation costs in EU countries when exploring the additional benefits of a mitigation target of 2°C or 1·5°C based on nationally determined contributions.^96^ As one of the biggest greenhouse gas emitters since the industrial revolution,^76^ Europe has the responsibility and opportunity to create more prosperous, equitable, and healthy economies that are based on zero-carbon energy.

This section explores two broad domains. The first set of indicators estimates the health-care costs of morbidity and mortality that might already be incurred by European populations because of climate change (indicators 4.1.1 to 4.1.4). These indicators include economic valuation of health impacts, which help frame health costs comparably across climate policy areas and evaluate the benefits of climate action.^4^ Socioeconomic costs, such as loss of labour supply and reduced economic growth per capita are also tracked. The second domain monitors the economics of the transition to zero-carbon economies (indicator 4.2.1).

### Indicator 4.1: health-linked economic impacts and mitigation of climate change

#### Indicator 4.1.1: economic losses due to climate-related extreme events

Climate-related extreme events can damage infrastructures, undermine public service provision, and result in both direct economic losses (ie, total or partial destruction of physical assets) and indirect losses (ie, subsequent or secondary results of the initial impact), which could have additional health implications (indicator 1.2.1 and 1.2.2). With data provided by Swiss Re, this indicator tracks the total economic losses (insured and uninsured) resulting from exposure to climate-related extreme events.

Between 2010 and 2021, the highest economic losses due to climate-related extreme events in Europe were observed in 2021, with an estimated absolute economic loss totalling almost €48 billion. Although €17·7 billion (37%) of the losses were insured, uninsured measurable losses were €30·2 billion (63%) (appendix p 118). Germany had the greatest economic loss, with an absolute economic loss of €30·3 billion (63% of total European losses), of which €9·8 billion was insured. This loss is mostly related to the German floods in July, 2021, which happened after heavy rainfall, destroying infrastructure and resulting in more than 200 deaths,^100^ and severe thunder and hailstorms that happened in Germany in 2021.

#### Indicator 4.1.2: heat impact on labour supply

Exposure to extreme heat can undermine people's capacity to work, both through the direct impacts on the health of workers and by reducing labour supply and productivity.^101^ The resulting losses of labour output not only affect the broad economy, but also worker incomes, which could have additional health implications.^102^, ^103^, ^104^ Appropriately designed early warning systems can reduce the negative health and labour impacts linked to heat stress. This indicator combines NUTS 2 labour supply data with temperature and precipitation data from the ERA5-Land to track the impact of temperature on labour supply (number of working hours) for highly exposed occupations (agriculture, forestry, mining and quarrying, and construction; appendix p 119).

**Figure 8:**
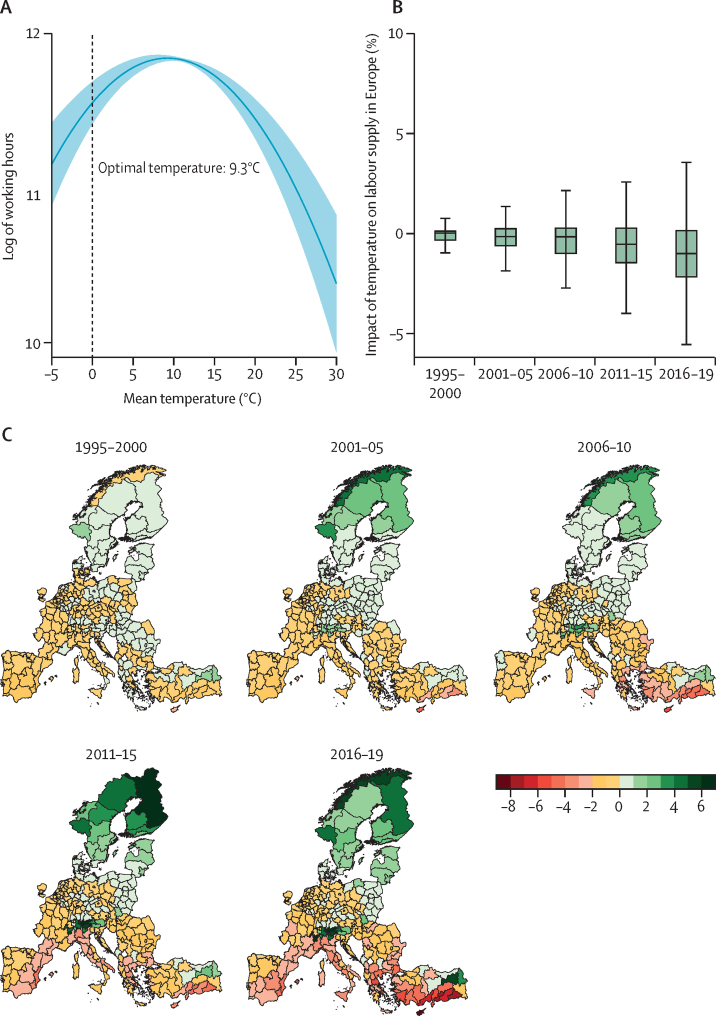
Labour supply and temperature in Europe (A) Non-linear (quadratic) relationship between mean temperature and log of number of working hours (blue line) from 1995 to 2019 with 95% confidence interval (light blue shading). The estimates are generated from a fixed-effects regression with the log of the number of working hours and ERA5-Land temperature data at the NUTS 2 level. The specification also includes precipitation and its second-degree polynomial, and NUTS level 2 and year fixed-effects. The standard errors are clustered at the country level. (B) Percentage change in the number of working hours (weighted by total number of working hours in 2019) due to change in temperature compared with the baseline period of 1965–94. (C) Change in high-exposure labour supply (%) in Europe due to temperature change; counterfactual analysis for each time period compared with the long-term mean of 1965–94. ERA5-Land=European Centre for Medium Range Weather Forecasts. NUTS 2=Nomenclature of Territorial Units for Statistics level 2.

The association between labour supply in Europe and temperature is non-linear, with the number of working hours being maximised at an annual mean temperature of 9·3°C (figure 8A). Combining the econometric estimates with change in temperature from the baseline average in 1965–94 reveals an estimated 0·23% decline in the number of working hours (just under 4 h per worker per year) due to temperature increase during the period 1995–2000 compared with the baseline. Labour supply in high-exposure sectors was 0·98% lower in 2016–19 (just under 16 h per worker) due to temperature change. The highest percentage declines in working hours (figure 8B) are estimated to be in Cyprus, South Aegean in Greece, and the Balearic Islands in Spain (figure 8C).

#### Indicator 4.1.3: impact of heat on economic activity

Economic activity is adversely affected by warming temperatures, with cascading impacts on wellbeing from unemployment, mental stress, and overall economic pressures.^105^, ^106^, ^107^, ^108^ This indicator tracks the impact of temperature anomalies (ie, a shift from the reference value or long-term average) from a long-term mean (1981–2010) on economic activity measured by real GDP per capita growth at the NUTS 2 in Europe. A time-varying coefficient regression combined with subnational economic data and high-resolution temperature and precipitation data from ERA5-Land was used to estimate this relationship. Because of the economic disparity between northern and southern Europe, the estimations were run separately for the two regions.

In 2019, GDP per capita growth in southern Europe was –0·90% (95% CI –0·87 to –0·91) lower due to positive temperature anomalies (a positive anomaly indicates that the observed temperature was warmer than the reference value) compared with 1981–2000 temperatures, and –0·106% (95% CI –0·100 to –0·111) lower in 2001. The findings show that positive temperature anomalies have not only been associated with reduced GDP per capita growth in southern Europe, but that they have also increased over time. Notably, this was not observed for northern Europe (appendix pp 120–21).

#### Indicator 4.1.4: monetised value of unhealthy diets

As shown by indicator 3.4.2, imbalanced diets are projected to have resulted in an estimated additional 2·2 million deaths annually in Europe, while also contributing to high greenhouse gas emissions from the agricultural sector (indicator 3.4.1). This indicator explores the monetised value of diet-related mortality by placing an economic value on the mortality attributable to imbalanced diets, as defined in indicator 3.4.2 (appendix pp 122–23).

In 2019, the monetised value of the deaths attributable to imbalanced diets amounted to US$9·4 trillion in 2019, equal to 32% of European GDP (appendix pp 122–23). The economic burden was highest in central and eastern Europe (56% of regional GDP, USD 4·3 trillion), and then southern Europe (25%, USD 1·4 trillion), northern Europe (24%, USD 1·4 trillion), and western Europe (24%, USD 2·5 trillion). These estimates provide evidence on the health co-benefits of dietary transition, and the benefits of policies that support healthier, more plant-based diets.^91^, ^92^, ^93^

### Indicator 4.2: economics of the transition to zero-carbon economies

#### Indicator 4.2.1: net value of fossil fuel subsidies and carbon prices

Introducing adequate carbon pricing mechanisms can internalise the negative externalities of fossil fuels (ie, when a price reflects the costs of emitting pollution), including the health impacts into prices paid for goods and services that generate these externalities. By better reflecting the actual cost of fossil fuel burning, these mechanisms can support the transition towards low-carbon economies, and support sustainable development. Not all European countries, however, set carbon prices. Furthermore, in countries where carbon prices are set, carbon prices are low, and the set prices can often be undermined by co-existing fossil fuel subsidies or a lack of carbon border adjustment mechanisms.^109^

Using data from the International Energy Agency, Organisation for Economic Co-operation and Development, the World Bank, and WHO, this indicator subtracts fossil fuel subsidies from carbon price revenues to estimate the economy-wide average net carbon revenues and prices in Europe (WHO European Region). In 2019, 32 (60·3%) of 53 European countries analysed had carbon pricing mechanisms in place (appendix p 124). However, only 15 countries had net-positive carbon prices, discouraging fossil fuel use. 28 (52·8%) of the countries had net-negative carbon prices (ie, subsidising fossil fuels). 15 countries provide net subsidies to fossil fuels that exceed one billion euros each year. The median value of subsidies in countries with a net-negative carbon price was €1·2 billion.

### Conclusion

The indicators in this section show the substantial economic losses that climate-related health impacts are already causing across Europe, including the losses due to climate-related extreme events (indicator 4.1.1), reduced labour supply (indicator 4.1.2), and reduced GDP per capita growth (indicator 4.1.3). Importantly, these impacts are unequally distributed, with southern Europe generally being the most negatively affected. Simultaneously, 28 European countries still provide overall subsidies for fossil fuels, costing a total of €70·7 billion and providing financial constraints to meeting decarbonisation goals for a healthier future (indicator 4.2.1).

Here, we provide a starting point for exploring annual economic indicators related to health and climate change in Europe. Further work is ongoing to develop indicators related to the monetised value of heat-related mortality, the monetised value of the health impacts of air pollution, and the employment in low-carbon and high-carbon industries.

## Section 5: politics and governance

The previous sections have shown the urgent need to strengthen the response to the health impacts of climate change in Europe, which requires a supportive political context in which key actors and institutions across society acknowledge and engage with the health dimensions of climate change. This section tracks engagement and coverage of health and climate change in wider political and governance structures in Europe. Adopting a broad, societal approach to the politics and governance of climate change and health, this section explores engagement across different domains that influence the shape and speed of Europe's response.^5^ In four domains, the indicators in this section assess the engagement by scientists (indicator 5.1), individuals on social media (indicator 5.2), politicians (indicator 5.3), and the corporate sector (indicator 5.4).

### Indicator 5.1: coverage of health and climate change in scientific journals

Scientific evidence is the foundation of progress on health and climate change,^110^ informing media coverage, public engagement, and government and private sector responses.^111^ This indicator tracks the number of scientific publications on climate change and health focused on Europe from 1990 to 2021 by applying machine learning and natural language processing methods to identify and classify scientific publications with Web of Science Core Collection, Scopus, and PubMed.^112^

**Figure 9:**
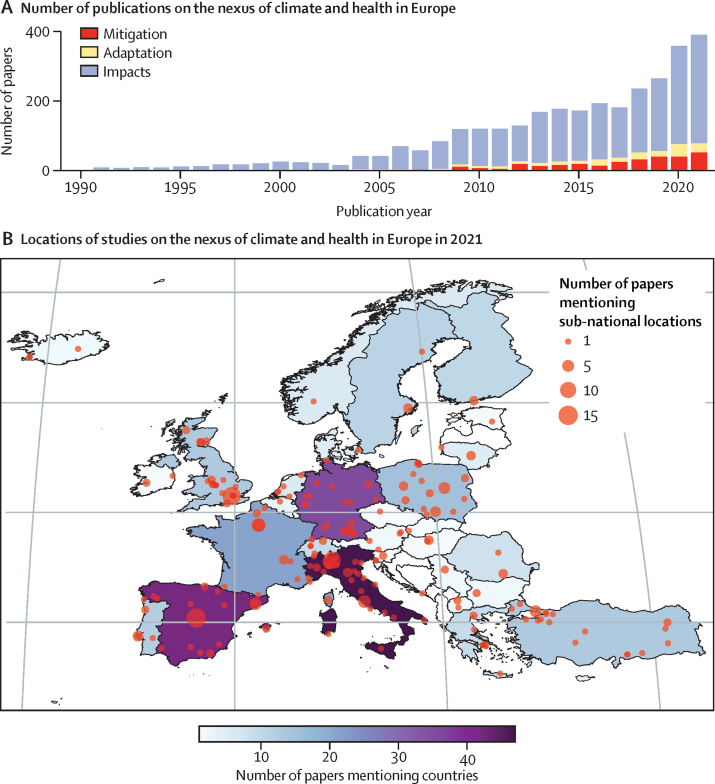
Number of academic publications per year on health and climate change, and locations of the study focus in Europe (A) Number of academic publications on health and climate change (mitigation, adaptation, impact) in Europe during the period 1990–2021. (B) Locations of study focus on the nexus of climate and health in Europe in 2021. Country shading indicates the number of academic publications on a specific country. Blue points refer to publications mentioning subnational locations (eg, cities), with point size indicating the number of publications.

There has been an increase in scientific engagement with climate change and health since the early 2000s, with a large increase in the past five years (figure 9; appendix pp 125–28). In 2021, 366 articles on the health impacts of climate change in Europe were published—an increase of 9% from 2020. The most studied countries in 2021 were Italy (66 publications), Spain (65), and Germany (47). During the period 1990–2021, most published articles focused on the health impacts of climate change. However, there is increasing research on climate solutions as well, shown by a large increase in scientific publications on adaptation and mitigation during this period.

### Indicator 5.2: individual engagement with health and climate change on social media

Little is known about how European populations engage with health and climate change. However, social media in Europe, particularly Twitter, exposes the online engagement with the topic of health and climate change.^113^ This indicator tracks the total number of tweets per month in 2021, by European users who refer to climate change and health. To construct this indicator, 22 official European languages were searched. Tweets from European languages that are used widely outside of Europe were excluded because of geolocalisation challenges (eg, English, French, Spanish, and Portuguese). Although this exclusion is a limitation of the current indicator form, this will be addressed in future iterations.

Overall, only 4711 (0·89%) of 526 993 tweets mentioning climate change also mentioned health in 2021. However, an increase in engagement was seen near the end of 2021, which could be linked to the COP26 summit (held in November, 2021) and related activities (appendix pp 129–38).

### Indicator 5.3: political engagement with health and climate change

The implementation of mitigation and adaptation measures requires political engagement with health and climate change in Europe. In the EU, the European Parliament is key for decision making on climate change.^114^, ^115^ This indicator tracks political engagement with health and climate change in the EU, by assessing mentions of climate-change-related and health-related terms in European Parliament legislators' speeches between 2014 and 2021.

Engagement with health and climate change in parliamentary speeches is generally low, with little variation over the 8-year period assessed (appendix pp 139–85). In 2021, there were 31 references to the health dimensions of climate change, compared with five references in 2014. In contrast, there were 618 references to climate change in the European Parliament in 2021. The highest engagement with climate change and health came from the Progressive Alliance of Socialists and Democrats and from German legislators.

### Indicator 5.4: Corporate sector engagement with health and climate change

Action by the corporate sector will be crucial in decreasing fossil fuel dependence.^116^ This indicator monitors corporate engagement in health and climate change by tracking mentions of terms related to climate change and health in the annual progress report that companies registered (self-report) in EEA countries or the UK submit to the UN Global Compact. The UN Global Compact is an initiative made to promote corporate social and environmental responsibility, to which corporations voluntarily sign up, although it has been criticised for possibly enabling so-called greenwashing.^117^

Since 2014, a growing proportion of EEA companies have referenced the intersection of climate change and health in their annual report, with a large increase in the past two years. In 2021, 1112 (35%) of 3206 monitored companies referenced the health dimensions of climate change, compared with 417 (18%) of 2298 companies in 2019 (appendix pp 186–93).

### Conclusion

Ensuring that health and climate change are reflected in political and governance structures across Europe is essential to reach the ambitions set out in the Paris Agreement and in the European Climate Law if societies are to adapt to the health effects of climate change.^78^ The politics and governance indicators in this section show mixed results of how different actors and institutions engage with climate change and health in Europe. There has been increasing engagement from the scientific sector (indicator 5.1) and corporate sector (indicator 5.4) with health and climate change in recent years, which has continued in 2021. Yet, individual online engagement with climate change and health (indicator 5.2) and political engagement in the EU Parliament (indicator 5.3) remain low. Therefore, a key challenge in the future will be to ensure that the developments in increasing scientific and corporate sector engagement continue and translate into a stronger policy response in Europe. To overcome these challenges, promoting increased public and political engagement with the health dimensions of climate change will be essential.

## Conclusion of the 2022 European report of the *Lancet* Countdown on health and climate change

**Figure 10:**
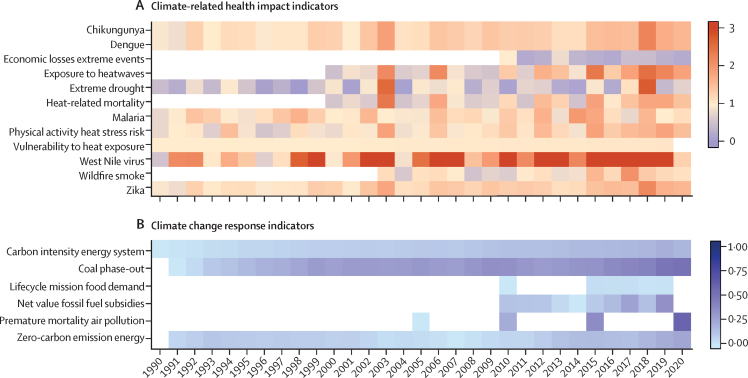
Overview of standardised impacts and responses tracked in the 2022 European report of the *Lancet* Countdown (A) Climate related health impact indicators, with higher values corresponding to worsening of the indicator tracked. (B) Climate change response indicators, with higher values corresponding to improvement in the indicator tracked (appendix p 198). Each indicator has been standardised to generate a yearly score. Standardisation was based on the yearly value divided by the baseline value for climate-related health impact indicators (A) and the yearly value divided by the worst case value (or target value) minus the worst case value for the climate change response indicators (B).

This report provides the first comprehensive assessment of progress on health and climate change in Europe by tracking 33 indicators in the domains of impact, exposure, and vulnerability (section 1); adaptation, planning, and resilience (section 2); mitigation actions and health co-benefits (section 3); economics and finance (section 4); and politics and governance (section 5). Europe is facing many catastrophic events that threaten the security and livelihoods of populations across Europe and globally. The *Lancet* Countdown in Europe highlights the accelerating trends in health-related hazards, exposures, vulnerabilities, and risks from climate change, and insufficiently ambitious adaptation and mitigation strategies (figure 10).

The health risks for almost all indicators tracked here are increasing in Europe. Clinically relevant pollen seasons are starting earlier each year (indicator 1.4) and climate suitability for water-borne and vector-borne diseases is rapidly increasing (indicators 1.3.1 to 1.3.4). Assuming no adaptation, heat exposure has increased by 57% between the first and second decade of the 21st century; exercising under extreme heat is posing acute health risks; and heat-related deaths are increasing (indicators 1.1.1 to 1.1.4). The frequency of extreme drought in affected areas has increased in the past decade (indicator 1.2.2). By contrast, wildfire smoke exposure did not increase during the period 2003–20, despite an increase in meteorological fire risk, likely resulting from effective fire prevention and suppression measures (indicator 1.2.1).^31^, ^32^ Heterogeneous geographical patterns of these impacts are observed across Europe, with many indicators reflecting highest absolute risk and increasing climate suitability for infectious diseases in central and southern Europe. Health risks paired with substantial economic losses related to climate, such as climate-related extreme events, reduced labour supply, and reduced GDP per capita growth (section 4). Without intervention, these impacts are likely to worsen in the coming years.

There are some encouraging trends in adaptation in parts of Europe, with select countries and cities adopting adaptation plans for health, doing health risks assessments, implementing early warning systems, and increasing green space exposure (section 2). Although there has been some progress in reducing the carbon intensity of the energy system and phasing out coal for electricity generation, mitigation efforts have been inadequate to meet 2030 and 2050 reduction targets (indicator 3.1.1 to 3.1.3). The pace of decarbonisation for electricity generation, residential heating, and transport in Europe does not support efforts to meet WHO guidelines^94^ for safe ambient PM_2·5_ concentrations and would need to accelerate five-fold (indicator 3.1.1) to reach net-zero carbon emissions by 2050 (indicators 3.1 to 3.3). Little progress has been made in the adoption of more sustainable, healthy diets, resulting in greenhouse gas emissions and thousands of deaths from high-carbon, animal-based diets (indicator 3.4). European countries provide overall subsidies for fossil fuels, providing financial constraints to meeting decarbonisation targets (indicator 4.2.1). Strengthening the response to the health impacts of climate change requires key actors and institutions to engage with the health dimensions of climate change to create a supportive political context. However, when comparing political engagement and individual online engagement of climate change and health with climate change engagement more broadly, engagement is still relatively low (indicators 5.2 and 5.3).

Without urgent acceleration in mitigation and adaptation efforts, the health impacts of climate change are likely to worsen in the coming years, affecting the wellbeing and lives of millions of people. The implementation of ambitious mitigation and adaptation strategies will not only protect lives and human wellbeing in Europe, but also in countries that have historically contributed least to anthropogenic climate change.^76^ The current energy security threats and volatile energy prices further highlight the co-benefits of the transition to renewable energy, increasing energy independence and resilience.^81^ This report highlights the urgent need and opportunities for accelerated action in line with climate targets; to support a healthy, climate-resilient future for all people.

## Declaration of interests

VK and OS are staff members of the WHO Regional Office for Europe. The authors alone are responsible for the views expressed in this publication and they do not necessarily represent the decisions or policies of the World Health Organization. The designations employed and the presentation of the material in this publication do not imply the expression of any opinion whatsoever on the part of WHO concerning the legal status of any country, territory, city or area or of its authorities, or concerning the delimitation of its frontiers or boundaries. Dotted and dashed lines on maps represent approximate border lines for which there may not yet be full agreement. All other authors declare no competing interests.
