# Supplementary material for: The 2022 Europe report of the Lancet Countdown on health and climate change: towards a climate resilient future
Source: Lancet Public Health. 2022 Oct 26;7(11):e942–65. doi: 10.1016/S2468-2667(22)00197-9 (PMC9597587; doi:10.1016/S2468-2667(22)00197-9)
Supplement: Supplementary appendix [file mmc1.pdf]

THE LANCET
Public Health

Supplementary appendix

This online publication has been corrected. The corrected version first appeared at thelancet.com on November 3, 2022

This appendix formed part of the original submission and has been peer reviewed. We post it as supplied by the authors.

Supplement to: van Daalen KR, Romanello M, Rocklöv J, et al. The 2022 Europe report of the *Lancet* Countdown on health and climate change: towards a climate resilient future. *Lancet Public Health* 2022; **7:** e942–65.

The 2022 Europe Report of the *Lancet* Countdown on Health and Climate Change: towards a climate resilient future

Appendix

Table of contents

Geographic definition of Europe 4

Summary of type of data utilised in each indicator 5

Overview with headline findings 8

Section 1: Climate change impacts, exposures, and vulnerabilities **12**

1.1: Heat and health 12

Indicator 1.1.1: Vulnerability to heat exposure 12

Indicator 1.1.2: Exposure of vulnerable populations to heatwaves 15

Indicator 1.1.3: Physical activity related heat stress risk 18

Indicator 1.1.4: Heat-related mortality 24

1.2: Extreme events and Health 28

Indicator 1.2.1: Wildfire smoke 28

Indicator 1.2.2: Drought 36

1.3: Climate-sensitive infectious diseases 42

Indicator 1.3.1: Climate suitability *non-cholerae* Vibrio 42

Indicator 1.3.2: Climate suitability West Nile Virus 46

Indicator 1.3.3: Climate suitability Dengue (Chikungunya, Zika) 50

Indicator 1.3.4: Climate suitability Malaria 77

1.4: Allergens 83

Indicator 1.4.1: Allergenic trees 83

Section 2: Adaptation, planning and resilience for health **91**

2.1: Adaptation planning and assessment 91

Indicator 2.1.1: National assessments of climate change impacts, vulnerability, and adaptation for health 91

Indicator 2.1.2: National adaptation plans for health 91

Indicator 2.1.3: City-level climate change risks assessments 95

2.2: Enabling conditions, adaptation delivery and implementation 96

Indicator 2.2.1: Climate information services for health 95

Indicator 2.2.2: Green space 96

Indicator 2.2.3: Air conditioning benefits and harms 100

Section 3: Mitigation actions and health co-benefits **101**

3.1: Energy system and health 101

Indicator 3.1.1: Carbon intensity of the energy system 101

Indicator 3.1.2: Coal phase-out 102

Indicator 3.1.3: Renewable and zero-carbon emission electricity 103

3.2: Premature mortality attributable to ambient fine particles 105

3.3: Sustainable and healthy transport 108

3.4: Food, agriculture and health 110

Indicator 3.4.1: Life cycle emissions from food demand 110

Indicator 3.4.2: Sustainable diets 111

Section 4: Economics and finance **118**
4.1: The health-linked economic impacts and mitigation of climate change 118
Indicator 4.1.1: Economic losses due to climate-related extreme events 118
Indicator 4.1.2: Change in labour supply 119
Indicator 4.1.3: Impact of heat on economic activity 120
Indicator 4.1.4: Monetised value of unhealthy diets 122
4.2: The economics of the transition to zero-carbon economies 124
Indicator 4.2.1: Net value of fossil fuel subsidies and carbon prices 124
Section 5: Politics and governance 125
5.1: Coverage of health and climate change in scientific journals 125
5.2: Individual engagement with health and climate change on social media 129
5.3: Political engagement with health and climate change 139
5.4: Corporate sector engagement with health and climate change 186
Additional information to Figure 10 in the main text 194
References 195

Geographic definition of Europe

In this first *Lancet* Countdown in Europe indicator report, we include all 38 European Environment Agency (EEA) member states and cooperating countries (**see table 1**), plus the United Kingdom, where possible. Over time, we aim to expand the coverage to the entire World Health Organisation (WHO) European region (**see table 2**). As data availability varies across indicators, we provide a description of the geographic coverage of Europe used for each individual indicator in the relevant appendix sections. **Table 3** and **table 4** provide an overview of the member countries that are part of the European Union (EU) and the European Trade Association (EFTA).

Table 1 European Environment Agency (EEA) member and cooperating countries[1]

**Member countries**			
Austria	Belgium	Bulgaria	Croatia
Cyprus	Czechia	Denmark	Estonia
Finland	France	Germany	Greece
Hungary	Iceland	Ireland	Italy
Latvia	Liechtenstein	Lithuania	Luxembourg
Malta	Netherlands	Norway	Poland
Portugal	Romania	Slovakia	Slovenia
Spain	Sweden	Switzerland	Türkiye
**Cooperating countries**			
Albania	Bosnia and Herzegovina	Kosovo (UNSCR 1244)	Montenegro
North Macedonia	Serbia		

Table 2 World Health Organisation (WHO) European region member countries[2]

Albania	Andorra	Armenia	Austria	Azerbaijan
Belarus	Belgium	Bosnia and Herzegovina	Bulgaria	Croatia
Cyprus	Czechia	Denmark	Estonia	Finland
France	Georgia	Germany	Greece	Hungary
Iceland	Ireland	Israel	Italy	Kazakhstan
Kyrgyzstan	Latvia	Lithuania	Luxembourg	Malta
Monaco	Montenegro	Netherlands	North Macedonia	Norway
Poland	Portugal	Republic of Moldova	Romania	Russian Federation
San Marino	Serbia	Slovakia	Slovenia	Spain
Sweden	Switzerland	Tajikistan	Türkiye	Turkmenistan
Ukraine	United Kingdom of Great Britain and Northern Ireland	Uzbekistan		

Table 3 European Union (EU) member and candidate countries[3,4]

**Member countries**			
Austria	Belgium	Bulgaria	Croatia
Cyprus	Czech Republic	Denmark	Estonia
Finland	France	Germany	Greece
Hungary	Ireland	Italy	Latvia
Lithuania	Luxembourg	Malta	Netherlands
Poland	Portugal	Romania	Slovakia
Slovenia	Spain	Sweden	
**Candidate countries**			
Albania	North Macedonia	Montenegro	Serbia
Türkiye			
**Potential candidate countries**			
Bosnia and Herzegovina	Kosovo (UNSCR 1244)	Iceland	

Table 4 European Free Trade Association (EFTA) countries[5]

**Member countries**			
Iceland	Liechtenstein	Norway	Switzerland

Summary of data inputs for each indicator

Table 5 Data inputs for each indicator

Indicator	Data inputs
**1. Climate change impacts, exposures, and vulnerabilities**	
1.1 Heat and health	
1.1.1 Vulnerability to heat exposure	- Population estimates, GBD 2019<br>- DALYs and deaths, GBD 2019<br>- Urban population, UNDP Urbanization Prospects 2018
1.1.2 Exposure of vulnerable populations to heatwaves	- Climatic data, ERA5-Land reanalysis<br>- Population data, NASA SEDAC GPWv4 & Eurostat demographic census data
1.1.3 Physical activity related heat stress risk	- Climatic data, ERA5-Land reanalysis<br>- Population data, Eurostat GEOSTAT
1.1.4 Heat-related mortality	- Climatic data, ERA5-Land reanalysis<br>- Population data, Eurostat<br>- Mortality data, Eurostat
1.2 Extreme events and Health	
1.2.1 Wildfire smoke	- Population data, Eurostat GEOSTAT<br>- Geographic data, Eurostat GISCO<br>- Mortality, Eurostat<br>- Fire smoke dispersion forecasts, IS4FIRES & SILAM models based on Thermal Anomalies and Fire MOD14/MYD14<br>- Fire weather index
1.2.2 Drought	- Climatic data, ERA5-Land reanalysis
1.3 Climate-sensitive infectious diseases	
1.3.1 Climate suitability *non-cholerae* Vibrio	- Population data, Eurostat GEOSTAT<br>- Sea surface temperature data, GHRSST Level 4 MUR Global Foundation Sea Surface Temperature Analysis (v4.1)<br>- Sea surface salinity data, Ocean Physical Reanalysis and Analysis EU Copernicus Marine Service
1.3.2 Climate suitability West Nile Virus	- Climatic data, ERA5-Land reanalysis<br>- WNV infections data, ECDC
1.3.3 Climate suitability Dengue	- Climatic data, ERA5-Land reanalysis & C3S<br>- Population data, Hybrid gridded data combining NASA SEDAC GPWv4 with ISIMIP Histsoc and World Population Prospects demographic modelling data
1.3.4 Climate suitability Malaria	- Climatic data, ERA5-Land reanalysis<br>- Land cover, CORINE<br>- Altitude data, JISAO
1.4 Allergens	
1.4.1 Allergenic trees	- Climatic data, ERA5-Land reanalysis<br>- Land cover data, ECOCLIMAP, EFI and GLC-SHARE<br>- Pollen data, European Aeroallergen Network
**2. Adaptation, planning and resilience for health**	
2.1 Adaptation planning and assessment	
2.1.1 National assessments of climate change impacts, vulnerability and adaptation for health	- Questionnaire data, WHO Health and Climate Change Survey
2.1.2 National adaptation plans for health	- Questionnaire data, WHO Health and Climate Change Survey
2.2. Enabling conditions, adaptation delivery and implementation	
2.2.1 Climate information services for health	- Questionnaire data, WHO Health and Climate Change Survey
2.2.2 Green space	- Population data, Eurostat<br>- NVDI data, MOD14Q1 from Terra MODIS<br>- Land cover data, High Resolution Layer TCD, Urban Atlas Land use/Land<br>- Cover data & Urban Atlas Street Tree layer
2.2.3 Air conditioning benefits and harms	- Airconditioning data, IEA
**Section 3. Mitigation actions and health co-benefits**	
3.1. Energy system and health	
3.1.1 Carbon intensity of the energy system	- CO2 Emission from Fuel Combustion dataset, IEA
3.1.2 Coal phase-out	- World Extended Energy Balances dataset, IEA
3.1.3 Renewable and zero-carbon emission electricity	- World Extended Energy Balances dataset, IEA
3.2 Premature mortality attributable to ambient fine particles	- Energy consumption, Eurostat & IEA<br>- Agricultural activity, FAOSTAT

	- Fertiliser use, IFASTAT<br>- Municipal waste, GAINS internal calculations<br>- Mortality data, Eurostat & UN World Population Prospects
3.3 Sustainable and healthy transport	- Fuel use, World Extended Energy Balances dataset, IEA<br>- Population data, UN World Population Prospects<br>- Model split of passenger transport, Eurostat
3.4. Food, agriculture and health	
3.4.1 Life cycle emissions from food demand	- Food demand, FAO's Food Balance Sheets<br>- Life-cycle emissions, Poore & Nemecek 2018
3.4.2 Sustainable diets	- Population data, GBD 2019<br>- Mortality data, GBD 2019<br>- Food consumption, FAO's Food Balance Sheets<br>- Health estimates, meta-analysis of prospective cohort studies
**Section 4: Economics and finance**	
4.1. The health linked economic impacts of climate change and its mitigation	
4.1.1 Economic losses due to climate-related extreme events	- Swiss Re Institute sigma catastrophe database<br>- IMF World Economic Outlook
4.1.2 Change in labour supply	- Climatic data, ERA5-Land reanalysis<br>- Labour data, Eurostat Regional Database from the EU Labour Force Survey
4.1.3 Impact of heat on economic activity	- Climatic data, ERA5-Land reanalysis<br>- GDP growth data, Eurostat Regional Database from the EU Labour Force Survey
4.1.4 Monetised value of unhealthy diets	- Values of statistical life, OECD<br>- Diet-related health impacts from indicator 3.4.2
4.2. The economics of the transition to zero-carbon economies	
4.2.1 Net value of fossil fuel subsidies and carbon prices	- Fossil fuel subsidies, IEA, OECD<br>- Carbon pricing, World Bank Carbon Pricing Dashboard<br>- CO2 emissions from fuel, IEA<br>- Health expenditure, WHO,<br>- US Dollar GDP deflator index, IMF World Economic Outlook database
**Section 5: Politics and governance**	
5.1 Coverage of health and climate change in scientific journals	- Scientific databases, Scopus, Medline, Web of Science Core Collection
5.2 Individual engagement with health and climate change on social media	- Twitter Developer API for the Academic purposes
5.3 Political engagement with health and climate change	- EU parliament debates transcripts
5.4 Corporate sector engagement with health and climate change	- UN GCCOP reports

ABBREVIATIONS:
CORINE; Copernicus Land Monitoring Service, **C3S**; Copernicus Climate Change Service, **DALY**; Disability-Adjusted Life Years, **ECDC**; European Centre for Disease Prevention and Control, **EFI**; European Forest Institute, **EU**; European Union, Eurostat; European Statistical Office, **FAO**; Food and Agriculture Organization, **FAOSTAT**; Food and Agriculture Organization Corporate Statistical Database, **GAINS**; Greenhouse Gas-Air Pollution Interactions and Synergies, **GBD**; Global Burden of Disease, **GCCOP**; Global Compact Communication Progress; **GDP**; Gross Domestic Product, **GISCO**; Geographical Information System, **GLC**; Global Land Cover, **GPW**; Gridded Population of the World, **GHRSST**; Group for High Resolution Sea Surface Temperature, **IEA**; International Energy Agency, **IFASTAT**; International Fertilizer Association Statistics, **IMF**; International Monetary Fund, **ISIMIP**; Inter-Sectoral Impact Model Intercomparison Project, **IS4FIRES**; Integrated Monitoring and Modelling System for Wildland Fires, **JISAO**; Joint Institute for The Study of Atmosphere and Ocean, **MODIS**; Moderate Resolution Imaging Spectroradiometer, **MUR**: Multiscale Ultrahigh Resolution, **NASA**; National Aeronautics and Space Administration; **NVDI**; Normalised Vegetation Index, **OECD**; Organisation for Economic Co-operation and Development, **SEDAC**; Socioeconomic Data and Applications Center, **SILAM**; System for Integrated Modelling of Atmospheric Composition, **TCD**; Tree Cover Density, **UN**; United Nations, **UNDP**; United Nations Development Programme, **WNV**; West-Nile Virus, **WHO**; World Health Organization

Overview with headline findings

In the following section we provide an overview with headline findings for each individual indicator included in the 2022 *Lancet* Countdown in Europe report.

Section 1: Climate change impacts, exposures, and vulnerabilities

1.1 Heat and health

1.1.1 Vulnerability to heat exposure

Vulnerability to heat exposure has risen steadily across all European regions, with an increase of 4% from 1990 to 2019. Older people in Northern Europe are more vulnerable to heat exposure. The highest increase over time has been observed in Central and Southern Europe.

1.1.2 Exposure of vulnerable populations to change in heatwaves

Between the first and second decades of the 21st century (2000-2009 vs 2010-2019) heatwave exposures increased 57% on average with local increases of more than 250%.

1.1.3 Physical activity and heat stress risk

Heat stress risk during physical activity has increased across Europe since 1990; particularly in Southern Europe, where risky hours per person reached 429 hours for medium intensity activities and 627 hours for strenuous activities in 2020.

1.1.4 Heat related mortality

Heat related mortality has increased by 15 annual deaths per million inhabitants per decade between 2000-2020.

1.2 Extreme events and Health

1.2.1 Wildfire smoke

Exposure to wildfire smoke showed small but uncertain negative trends in all European regions, possibly due to increased effectiveness of fire prevention and suppression measures.

1.2.2 Drought

55% (184/334) of NUTS2 regions have faced extreme to exceptional summer drought events between 2011-2020, in which one-third has experienced more than 40% of the drought events recorded over 7 decades (1950-2020) in the last decade (2011-2020).

1.3 Climate-sensitive infectious diseases

1.3.1 Climate suitability *non-cholerae Vibrio*

Suitable ecological conditions for pathogenic Vibrio is increasing across Europe over recent years both in terms season length and percentage of coastline with favourable conditions for Vibrio.

1.3.2 Climate suitability West Nile Virus

An accelerating trend of WNV outbreak risk was observed between 1951-2020, with the highest increases in Southern Europe (149%), and Central and Eastern Europe (163%), from 1951-1985 to 1986-2020.

1.3.3 Climate suitability Dengue

The basic reproduction R_0 *and length of transmission season (LTS) for dengue has increased in all European regions, from 1951-1985 to 1986-2020, with an overall increase in* R_0 *of 17.3%.*

1.3.4 Climate suitability Malaria

There has been a steady increase in the number of months suitable for Plasmodium vivax transmission in all European regions. The highest change occurred in Northern and Western Europe, with 21.6% and 25.2% increases in suitability, from 1951-1985 to 1986-2020.

1.4 Allergens

1.4.1 Allergenic trees

Warming climate during last 41 years has resulted in a 10 – 20 days earlier start of flowering season of major European allergy-relevant trees: birch, alder, and olive. The most-significant changes occurred in mountains (e.g., Alps, Balkans, Scandinavian ridge), where the season now starts more than one month earlier than in the 1980s.

Section 2: Adaptation, planning and resilience for health

2.1 Adaptation planning and assessment

2.2.1 National assessments of climate change impacts, vulnerability, and adaptation for health

In 2021, 10 (45%) of 22 countries reported having undertaken a climate change and health vulnerability and adaptation assessment. 14 (64%) of 22 countries reported having a multi-stakeholder mechanism on health and climate change that is currently operational.

2.2.2 National adaptation plans for health

In 2021, 15 (68%) of 22 assessed European countries reported having national health and climate change strategies or plans in place.

2.2.3 City-level climate change risk assessments

Out of 197 European cities in 2021, 150 (76%) reported having conducted a climate assessment. 59.9% (118/197) of cities reported that climate change threatened health services or public health, with heat-related illnesses most prominently identified as a climate related health hazard (identified by 87 cities).

2.2 Adaptation delivery and implementation

2.2.1 Climate information for health

In 2021, 45% (10/22) of countries in the WHO European region reported having climate-informed health surveillance systems for heat-related illnesses, 36% (8/22) for injury and mortality related to extreme climatic events, 27% (6/22) for vector-borne diseases, and 18% (4/22) for water-borne diseases.

2.2.2 Green space

Populated weighted greenness increased somewhat from 2000 to 2020 in most European countries with the largest increases observed in Southern Europe (13% mean increase) and the smallest in Western Europe (3% mean increase). The mean urban tree coverage in European cities in 2018 was 28.5% (EEA39) and 35.8% (EEA38) of the city (including commuting zones).

2.2.3 Air conditioning benefits and harms

For most European countries assessed, the proportion of households using air conditioning has increased steadily. Of the countries with individual-level country data, the highest increase was observed in Finland (162%) and Germany (122%). The downside of air conditioning use is that it contributes to greenhouse gas emissions, air pollution, peak electricity demand, and urban heat islands.

Section 3: Mitigation actions and health co-benefits

3.1 Energy system and health

3.1.1 Carbon intensity of the energy system

Between 2005 and 2019 emissions from fuel in Europe fell by 14% to 3.5GtCO_2 per year. The COVID pandemic had the effect of temporarily reducing Europe's emissions by 8% in 2020.

3.1.2 Coal phase-out

Coal remains a substantial fuel in the European energy mix at 12% of total energy supply in 2020. Current rates of reduction are incompatible with reaching net-zero by 2050.

3.1.3 Renewable and zero-carbon emission electricity

Share of zero-carbon total energy supply in Europe is only 21% and share of renewables in electricity is 17% in 2020, despite rapid growth in electricity generation from wind in Denmark, Ireland and the UK, and solar in Germany, Greece and Italy.

3.2 Premature mortality attributable to ambient fine particles

Air pollution related deaths from combustion of fossil fuels have decreased by 60% between 2005 and 2020, but still almost 120,000 deaths annually are attributable to these sources.

3.3 Sustainable and healthy transport

Per capita fossil fuel use in road transport peaked at 23GJ per person in 2007; electric vehicles are growing, but remain a small fraction of energy use for road transport.

3.4 Food, agriculture and health

3.4.1 Life cycle emissions from food demand

Between 2010 to 2019, there was little progress in reducing emissions associated with European food demand, which in 2019 amounted to 1.85 GtCO2-eq. Animal source foods continue to be responsible for over two thirds (77%) of those emissions.

3.4.2 Deaths attributable to dietary risks

About 2.2 million deaths in Europe were attributable to imbalanced diets in 2019, representing more than a quarter (26%) of all deaths amongst adults. Between 2010 and 2019, the proportion of diet-related deaths decreased by 2%.

Section 4: Economics and finance

4.1 The health-linked economic impacts and mitigation of climate change

4.1.1 Economic losses due to climate-related extreme events

Over the past decade, the highest economic losses due to climate-related extreme events were observed in 2021, with an absolute economic loss totalling €47,962 million. The vast majority of economic losses were experienced by Germany; €30,280 million (63% of total European losses).

4.1.2 Change in labour supply

Labour supply in high-exposure sectors was 0.98% (just under 16 hours per worker) lower in 2016-2019 than in 1965-1994 due to temperature change. In comparison, in 1995-2000 the reduction in labour supply was only 0.23% (just under 4 hours per worker).

4.1.3 Impact of heat on economic activity

In 2019, relative to 1981-2000 temperatures, GDP per capita growth in Southern Europe was 0.90% (95% CI: -0.87, -0.91) lower due to temperature anomalies and 0.106% (95% CI: -0.100, -0.111) lower in 2001

4.1.4 Monetised value of unhealthy diets

The value of imbalanced diets in Europe amounted to USD 9.4 trillion in 2019, representing 32% of GDP. The value of imbalanced diets was highest in Eastern Europe, where it amounted to over half (56%) of regional GDP.

4.2 The economics of the transition to zero-carbon economies

4.2.1 Net value of fossil fuel subsidies and carbon prices

28 (52.8%) of the 53 countries reviewed had a net-negative carbon price in 2019. Fifteen countries provided net subsidies to fossil fuels exceeding one billion euros each year.

Section 5: Politics and governance

5.1 Coverage of health and climate change in scientific journals

Growth in scientific papers investigating the intersection between health and climate change in Europe continued in 2021. 366 relevant papers were published mentioning locations in Europe, a 9% increase compared to 2020. While research on health implications of climate change impacts continues to dominate the evidence base, increasing attention has been given to climate solutions over the last 10 years.

5.2 Individual engagement with health and climate change on social media

Engagement with the health and climate change on Twitter in Europe is low, with a very small proportion of tweets on climate change mentioning health. There was a spike in engagement towards the end of 2021, linked to the COP26 summit and the publication of the Lancet Countdown annual report.

5.3 Political engagement with health and climate change

Engagement with health and climate change by legislators in the European Parliament remains low in 2021. The highest engagement with climate change and health comes from the Progressive Alliance of Socialists and Democrats (S&D) and from German legislators.

5.4 Corporate sector engagement with health and climate change

Engagement with health and climate change among European companies signed up to the UN Global Compact increased to their highest level in 2021, with 35% of corporations referencing health and climate change in their annual report.

Section 1: Climate change impacts, exposures, and vulnerability

1.1: Heat and health

Indicator 1.1.1: Vulnerability to extremes of heat

Methods

This indicator displays an index derived by taking the mean of proportion of the population over 65 years (1);[6] the prevalence of cardiovascular, diabetes and chronic respiratory diseases among the population over 65 years from the GBD study 2019 estimates (2)[7] and the proportion of the population living in urban areas (3)[8] as a measure of exposure to urban heat islands. The index ranges from 0 to 100 and is a measure of potential vulnerability of a country to heat exposure. Aggregated trends by European regions are presented for the period 1990 to 2019. Percentage change in vulnerability was also computed taking 1990 as the baseline year.

Geographic coverage of Europe

For this indicator, European Environment Agency (EEA) member and cooperating countries plus the United Kingdom were included.

Data

1. Global Burden of Disease Collaborative Network. Global Burden of Disease Study 2019 (GBD 2019) Population Estimates 1950-2019. Seattle, United States: Institute for Health Metrics and Evaluation (IHME), 2020.
2. Global Burden of Disease Collaborative Network. Global Burden of Disease Study 2019 (GBD 2019) Results. Seattle, United States: Institute for Health Metrics and Evaluation (IHME), 2020.Available from http://ghdx.healthdata.org/gbd-results-tool.
3. Urban population (% of total) The United Nations Population Division's World Urbanization Prospects. 2018 Revision.

Caveats

There is no consistent and universally accepted standard for distinguishing urban from rural areas, in part because of the wide variety of situations across countries. Most countries use an urban classification related to the size or characteristics of settlements. This indicator does not include the existence of heat early warning systems, or prevalence of cooling devices. Neither does it include the prevalence of green areas in cities.

Future form of the indicator

GBD and urbanization estimates now are revised annually; the indicator will be updated every year.

Additional analysis

Figure 1.1 shows the vulnerability to heat in Europe between 1990-2019 across European regions. Figure 1.2 displays a map contrasting the heat vulnerability between 1990-2019 for European countries. Figure 1.3 displays the trends in cardiovascular disease prevalence, proportion of the population in urban areas and the proportion of the population over 65 years old, which is used to calculate the heat vulnerability index

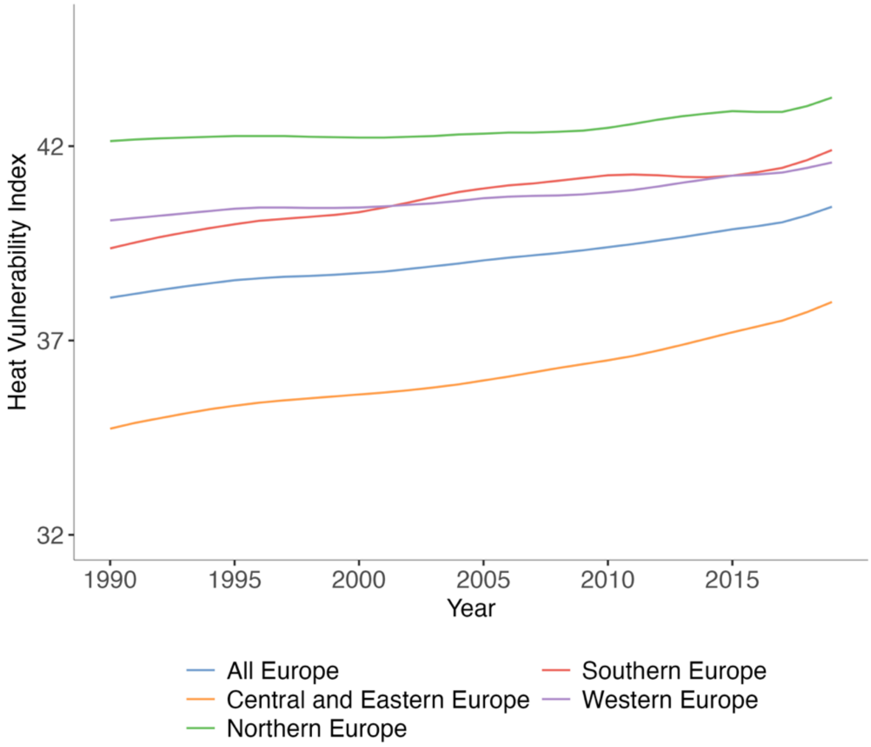

Figure 1.1. Vulnerability to heat in Europe between 1990-2019 across European regions.

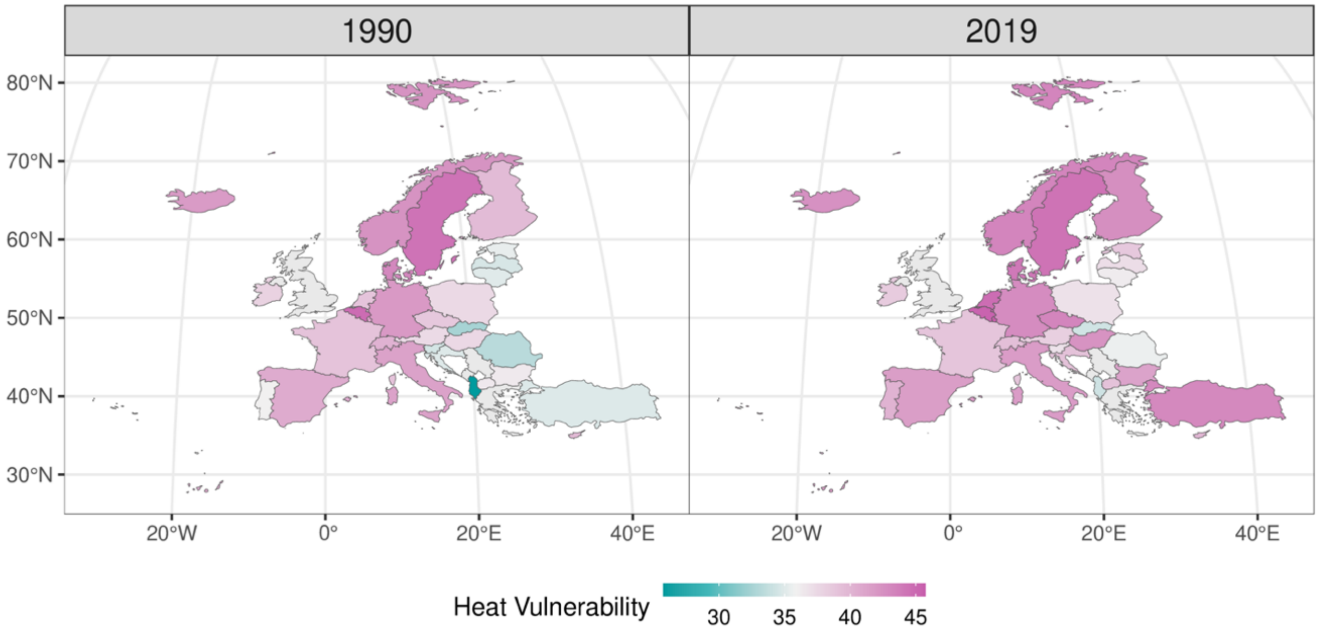

Figure 1.2 Map showing the heat vulnerability index at the country level in Europe in (a) 1990 and (b) 2019

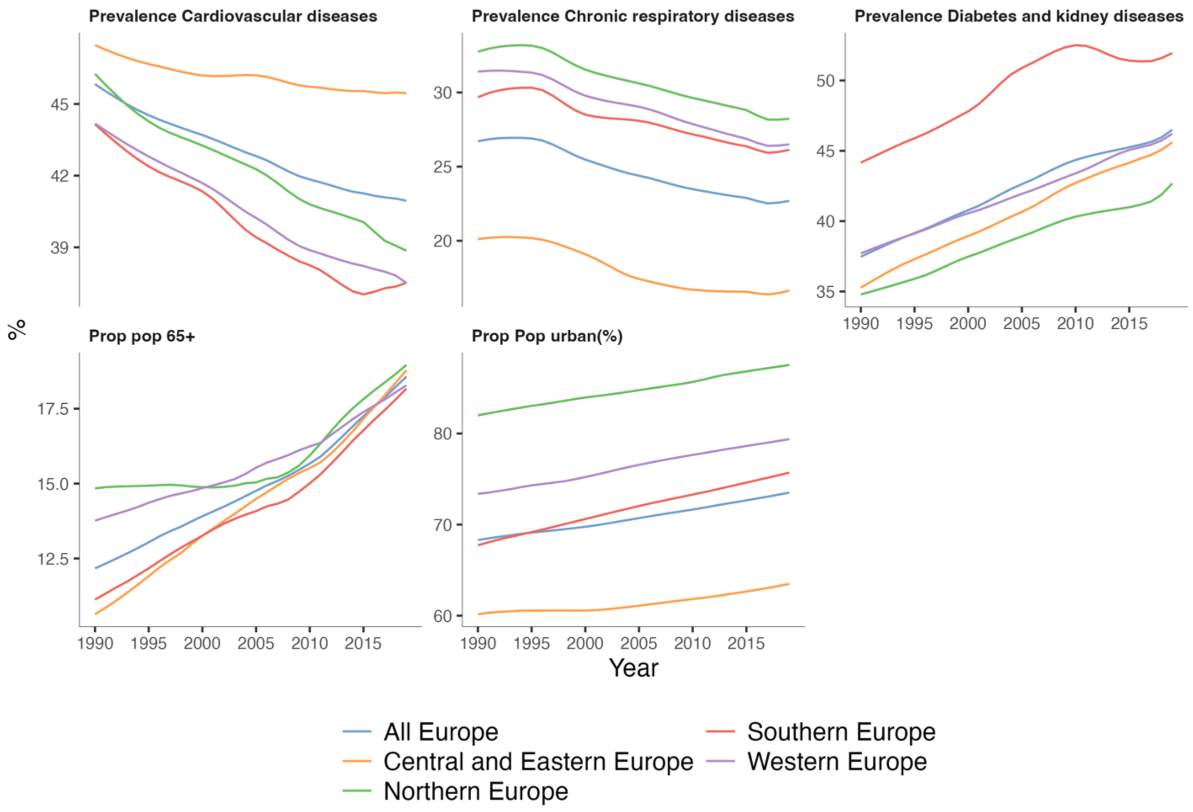

Figure 1.3 Trends in cardiovascular diseases prevalence, proportion of population in urban areas and proportion of the population 65 years and above used to calculate the heat vulnerability index between 1990-2019.

Indicator 1.1.2: Exposure of vulnerable populations to heatwaves

Methods

Heatwaves are defined as periods of 2 or more days where both the minimum and maximum temperatures are above the 95th percentile of the local climatology (defined using a 1986-2005 baseline period).

The vulnerable population is defined as those above the age of 65 and infants between 0 and 1 years old. Previous research has identified these groups as being particularly vulnerable to heatwave impacts on health.

Exposures were calculated by multiplying the number of heatwave days by vulnerable population count, producing an indicator of 'person-days' that reflects both the changes in frequency and duration of heatwaves.[9]

Geographic coverage of Europe

For this indicator we included the European Environment Agency (EEA) member and cooperating countries plus the United Kingdom.

Data

For climate data, ERA5-Land reanalysis data at 0.1° resolution was used. For each grid cell, the number of days with heatwaves per year was calculated.

To derive vulnerable population counts, spatiotemporal demographic data was derived from NASA SEDAC GPWv4 and Eurostat data. The spatial distribution of demographic age bands for 2010 was obtained from GPWv4. Eurostat demographic census data for NUTS2 regions (2016 definition) was projected onto the grid and used to adjust demographic fractions per grid cell for the years 2000-2021 relative to the 2010 baseline. The number of infants was estimated as the difference between total births and infant mortality rate. Missing values for demographic, birth, and infant death rate data obtained from Eurostat were filling using the most recent available year, preceding year, or the next available year (forward fill first then back fill).

Caveats

In order to estimate the time evolution of demographics, data from diverse sources was combined in order to obtain estimates of both the spatial and temporal characteristics. This has been subject to limited validation.

Future form of the indicator

Future versions of the indicator aim to also use Eurostat gridded population data. However, this data is only available for a selection of years, therefore it was decided for this first version to use this hybrid dataset in order to show the time series change and decade changes.

Additional analysis

Figure 1.4 shows the percentage change in heatwave exposure between the decade 2000-2009 and 2010-2019. These increases are broadly distributed across the region with particular hot spots in central and eastern Spain and in central Europe. Figure 1.5 shows the exposure to change in heatwaves (relative to a 1986-2005 baseline period) for infants and over 65s.

A

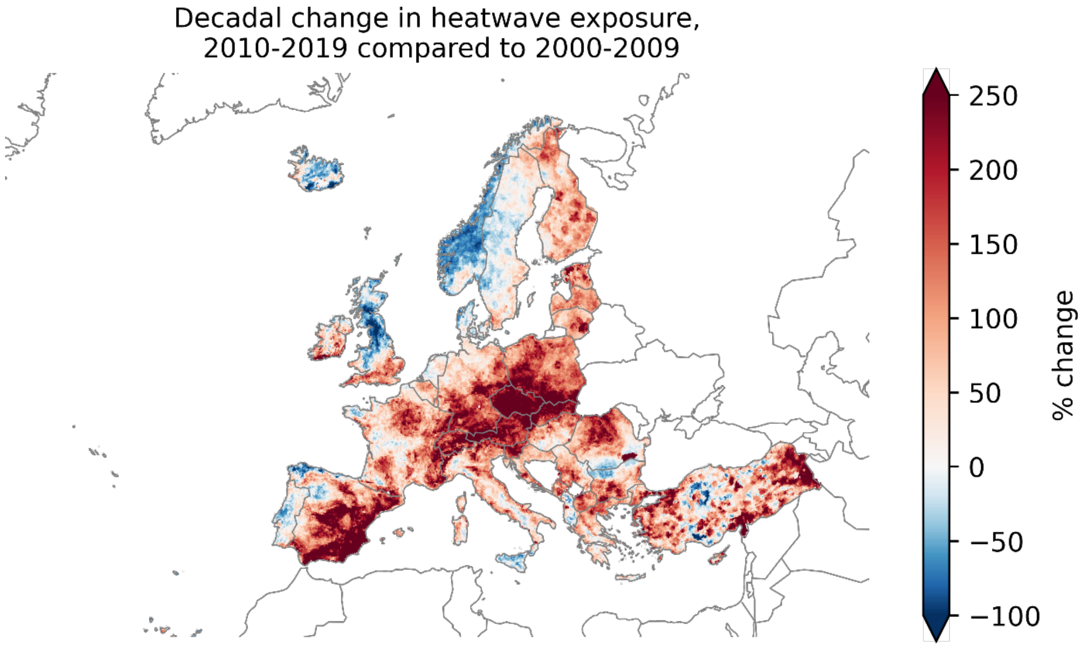
Decadal change in heatwave exposure,
2010-2019 compared to 2000-2009
250
200
150
100
50
0
−50
−100
% change

B

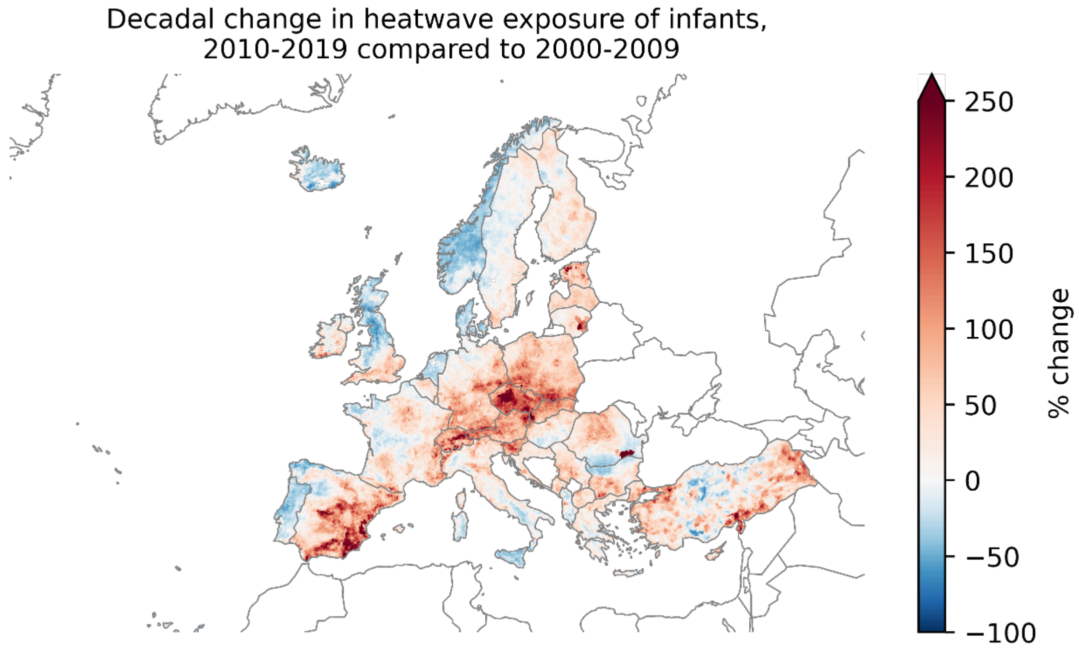
Decadal change in heatwave exposure of infants,
2010-2019 compared to 2000-2009
250
200
150
100
50
0
−50
−100
% change

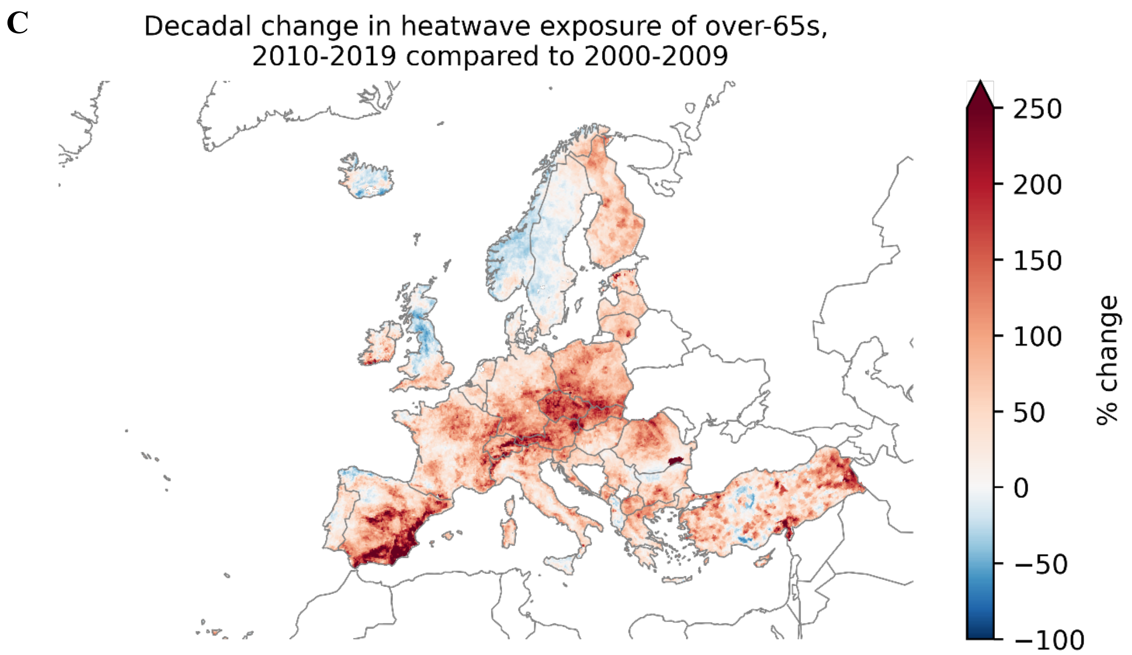

Figure 1.4 Decadal change in heatwave exposures of vulnerable people. (A) over 65 and infants combined, (B) infants, (C) over-65s, comparing the mean exposure by grid cell in 2010-2019 to 2000-2009

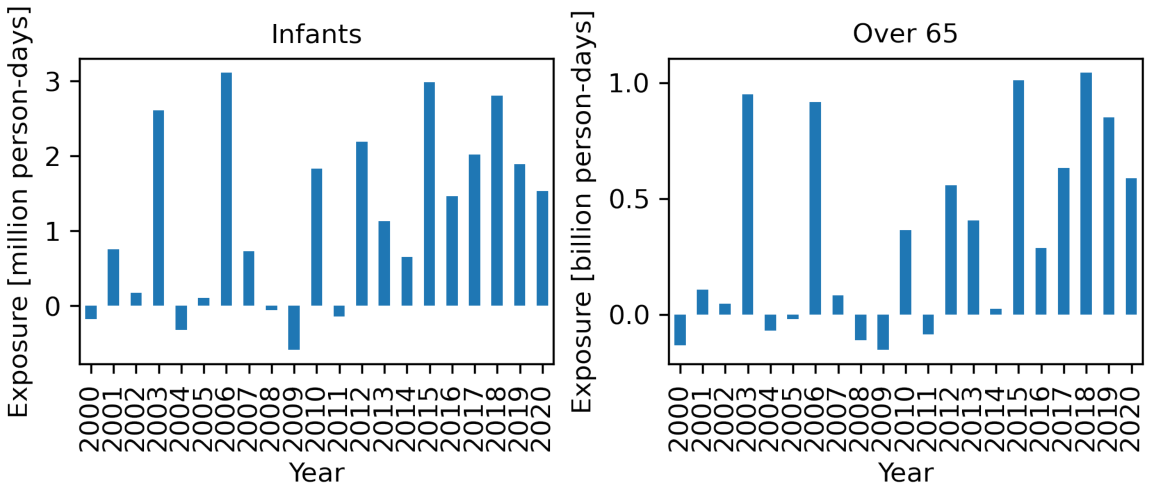

Figure 1.5 Exposure to change in heatwaves (relative to 1986-2005 baseline) for infants and over 65s.

Indicator 1.1.3: Physical activity-related heat stress risk

Methods

This indicator shows heat stress risk during physical activity as “risky hours per person per year”, based on the Sports Medicine Australia (SMA) Extreme Heat Policy.[10,11] An “hour at risk” is defined as an hour during which “heat” – represented by a combination of average 2-meter temperature and relative humidity during that hour – renders physical activity of a given intensity “risky”. In the SMA Policy,[10] risk is stratified into four levels (low, moderate, high and extreme) for each of five categories of physical activity, which are grouped according to intensity and accounting for typical clothing and equipment. Risk levels are defined in terms of actions required to avoid heat stress as follows; low risk: maintain hydration and wear light-weight clothing if needed; moderate risk: increase frequency and duration of rest breaks; high risk: use active cooling during rest breaks and/or before and after activity (e.g., drinking cold liquids; water dousing with a sponge or spray bottle); extreme risk: stop activity and seek shelter. (For reporting purposes in this indicator, moderate, high, and extreme risk were aggregated, except in figure 1.9). The indicator focuses on two categories of activity: category 3, which includes medium intensity activities such as cycling, football and tennis; and, category 5, which includes strenuous activities with specific clothing and equipment such as field hockey and mountain biking. For the indicator, these are referred to as “medium intensity activities” and “strenuous activities”, respectively.

The indicator covers the period from 1990 to 2020 but holds population levels constant at 2018 levels over the entire period. Thus, it specifically assesses temporal and spatial changes in risk that are attributable to natural and anthropogenic climate patterns and trends.

Risk functions of the following form, which define the thresholds between given levels of risk for a given category of physical activity, were derived from figures in the SMA guidelines using image processing.[12]

$$f(x) = a + bx + cx^2 + dx^3 + ex^4$$

where x is 2-meter temperature in a given hour, $f(x)$ is 2-meter relative humidity in a given hour, and a, b, c and d are coefficients that are specific to each activity category and risk transition (e.g., moderate to high). The coefficients are specified in table 1.1.

Climate data were obtained for Europe from the ERA5-Land 9km^2 grid for 2-meter temperature and 2-meter dew point temperature for each hour for the years 1990 to 2020.[13] These data were then combined to calculate hourly 2-meter relative humidity.[14] The hourly temperature and relative humidity data were used to derive the risk functions to calculate the level of heat stress risk during each hour for the two categories of physical activity in each 9km^2 grid cell.

Population data were obtained from the GEOSTAT 1km^2 population estimates for 2018 (population was fixed at these levels for all years in the analysis).[15] The total population in each ERA5-Land 9km^2 grid cell was estimated based on the location of the centroids of the GEOSTAT 1km^2 cells (See below how GEOSTAT cells falling in ERA5-Land cells with no climate data were dealt with).

For each 9km^2 grid cell, the annual number of person-hours at risk was calculated by summing the population over all hours in each year, for each level of risk, for each physical activity category. During this process, population grid cells that fell outside the ERA5-Land grid (which were principally on the coast) were assigned the risk level of the closest ERA5-Land grid cell with available climate data based on the Euclidian distance using the latitude and longitude coordinates. These estimates were then aggregated to NUTS 2 level and by broad geographic region (i.e. North, Central, West and South Europe).[16,17] For NUTS 2 regions, grid cells were assigned

to the region with which they had the greatest overlap (this could lead to some misclassification but the implications for our estimates are likely to be minimal), and the values of each cell falling in each region were summed. NUTS 2 regions were then assigned to broad geographic regions based on their centroids.

Next, risky hours per person per year was calculated by dividing the person-hours at risk by the population of the area of interest, and, total risky hours per person per year was calculated by summing risky hours for moderate, high, and extreme risk. Finally, the linear trends for annual change in risky hours per person per year were estimated by using linear regression to find the line of best fit, with uncertainty represented using 95% confidence intervals.

Table 1.1 Risk function coefficients by activity type and risk threshold

**Coefficients by physical activity type**	**Risk threshold**		
Medium intensity activity	*Low-Moderate*	*Moderate-High*	*High-Extreme*
a	1123·0830	1054·5207	1240·9576
b	-101·9229	-89·1276	-106·4948
c	3·7725	3·1001	3·7484
d	-0·0657	-0·0509	-0·0614
e	0·0004	0·0003	0·0004
Strenuous activity	*Low-Moderate*	*Moderate-High*	*High-Extreme*
a	1268·3137	1254·2166	846·5127
b	-117·2863	-110·5991	-59·4419
c	4·3161	3·8902	1·6317
d	-0·0733	-0·0626	-0·0194
e	0·0005	0·0004	0·0001

"Risk threshold" refers to the line separating one level of risk from another; for instance, low-moderate refers to the line between low risk and moderate risk. Coefficients are rounded to four decimal places.

Geographic coverage of Europe

For this indicator, we included the European Environment Agency (EEA) member and cooperating countries plus the United Kingdom, excluding Kosovo and Bosnia & Herzegovina as data was not available for these countries.

Data

1. Climate data were obtained from the European Centre for Medium-Range Weather Forecasts ERA5-Land dataset for hourly 2m temperature and hourly 2m dew point temperature, on a 9km^2 grid covering Europe, for the period 1990 to 2020.[13]
2. Population data were obtained from the Eurostat GEOSTAT data for total population estimated for the year 2018, on a 1km^2 grid covering Europe.[15]
3. Country and NUTS 2 borders were obtained from Eurostat GISCO.[16]
4. Broad European geographic regions (i.e. North, Central, West and South Europe) were available from Eurostat.[17]

Caveats

The first caveat is that the exposure-risk functions were originally developed for use in Australia. They were applied in a European-context based on the following considerations: no European-specific alternatives exist; the Australian guidelines were recently developed by leading scientists in the field based on the best available empirical evidence;[10,18] and, the functions rest on human physiology which may reasonably be assumed to be similar across high income countries (personal communication: Professor Ollie Jay, University of Sydney, e-mail, Jan 7 2022).

A second caveat is that the figures from which the risk functions were derived only consider temperatures in the range 26°C to 44°C (figure 1.6).[10] Thus it was necessary to extrapolate them based on the following justifications. For temperatures above 44°C, the underlying figures show that almost all hours (with the exception, for medium intensity activities, of the unlikely situation where relative humidity is close to 0%) with high temperatures would be classified as extreme risk for both of categories of physical activity considered in the indicator; thus, extrapolation at high temperatures is straightforward. For temperatures below 26°C, these show most hours would be low risk. However, at high levels of relative humidity, and depending on the intensity of exercise, risk may be moderate or high, or - very rarely - extreme. This means extrapolation is less certain at the low end of temperatures. In this regard, it was suggested by an author of the original guideline (personal communication: Professor Ollie Jay, University of Sydney, e-mail, Jan 10, 2022) that below 26°C risk would generally (except at very high levels of exertion) be expected to be quite low irrespective of relative humidity as dry heat loss becomes increasingly pronounced and is less dependent on sweat evaporation. Given this, it was suggested it would be reasonable to extrapolate the functions down to around 23°C and then assume that risk is "low" beyond this. Our extrapolated functions adhere to this recommendation.

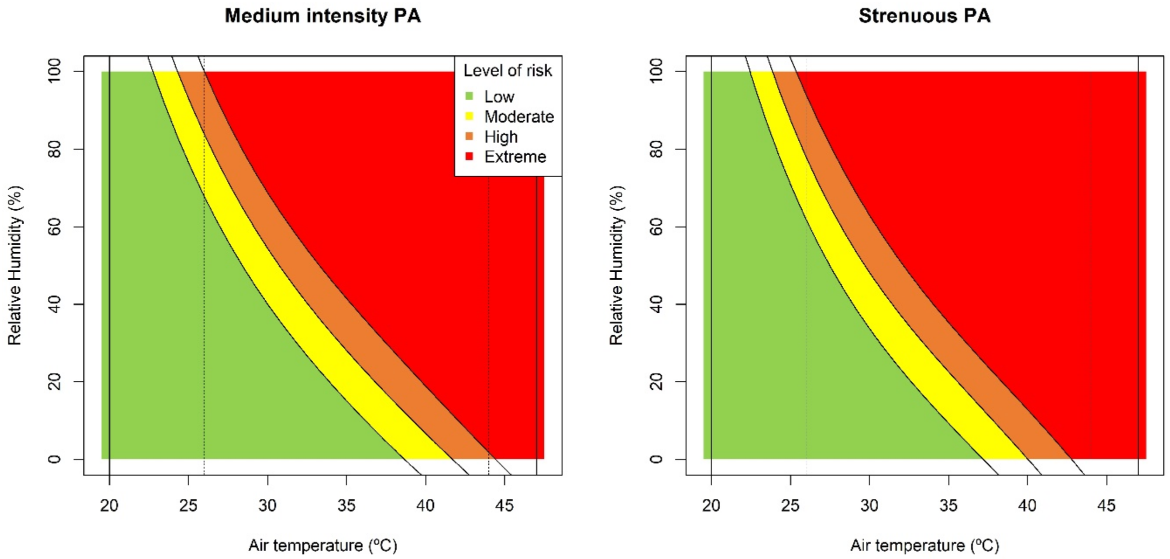

Figure 1.6 Form of the risk functions for medium intensity and strenuous physical activity.

Footnote for Figure 1.6. The vertical dashed lines indicate the temperature range covered by the figures in the SMA guidelines. The vertical solid lines show range outside which risk was always considered low (the line on the left) and extreme (the line on the right).

A third caveat is that population is fixed at 2018 levels when calculating the indicator for all years. This was partly a pragmatically based decision given the available data and resources, and 2018 was chosen as it offered the greatest spatial coverage of available options. The implication is that the indicator specifically assesses how risk has changed due to warming since 1990. This will have some influence on the spatial pattern of risk trends due to differences in population growth rates across regions, including those due to urbanization. Additionally, because of population growth since 1990, the magnitude of the change in person-hours of risk over time will tend to be lower than it would be if a moving population were used.

Future form of the indicator

Two main aspects of development will be considered. Firstly, the possibility of introducing a moving population so as to allow the assessment of how population changes are influencing risk patterns. Secondly, the incorporation of applicable new research that allows risk stratification by population group (e.g., age, gender).

Additional analysis

The results shown in the main text are complemented by, firstly, assessing risk patterns in more spatial detail, and, secondly, by looking at trends in risk of different levels at the European level.

While the figure in the main text shows that impacts have been greatest in South Europe, figure 1.7 and figure 1.8 show that there is significant heterogeneity within South Europe. Over the period 1990 to 2020, coastal regions in the Mediterranean experienced annual (absolute) rises in risky hours per person that were significantly larger - at times more than double - those seen in their more inland neighbours for both medium intensity and strenuous activities (figure 1.7). Likewise, the number of risky hours per person in the year 2020 was also considerably greater - again, often more than double - in coastal Mediterranean regions compared to adjacent inland regions (figure 1.8). That is, in Mediterranean coastal regions, physical activity-related heat stress risks are considerably higher and increasing (in absolute terms) considerably faster than in the rest of Europe.

Figure 1 in the main text aggregates risk by summing moderate, high and extreme risk. figure 1.9 shows these levels of risk separately over 1990 to 2020 for Europe as a whole. For both categories of physical activity, the majority of risk is at the moderate level: this entails taking additional rest breaks to avoid heat stress. Over time, however, the contribution of high-risk hours - which necessitate active cooling - has increased, particularly for strenuous activities. Hours at extreme risk, for which it is recommended that activity is ceased, are relatively infrequent but have increased over time for both categories of activity. In sum, while the majority of risky hours experienced so far may be addressed by taking more rest breaks, interruptions that require more intense actions or even stoppage are on the rise.

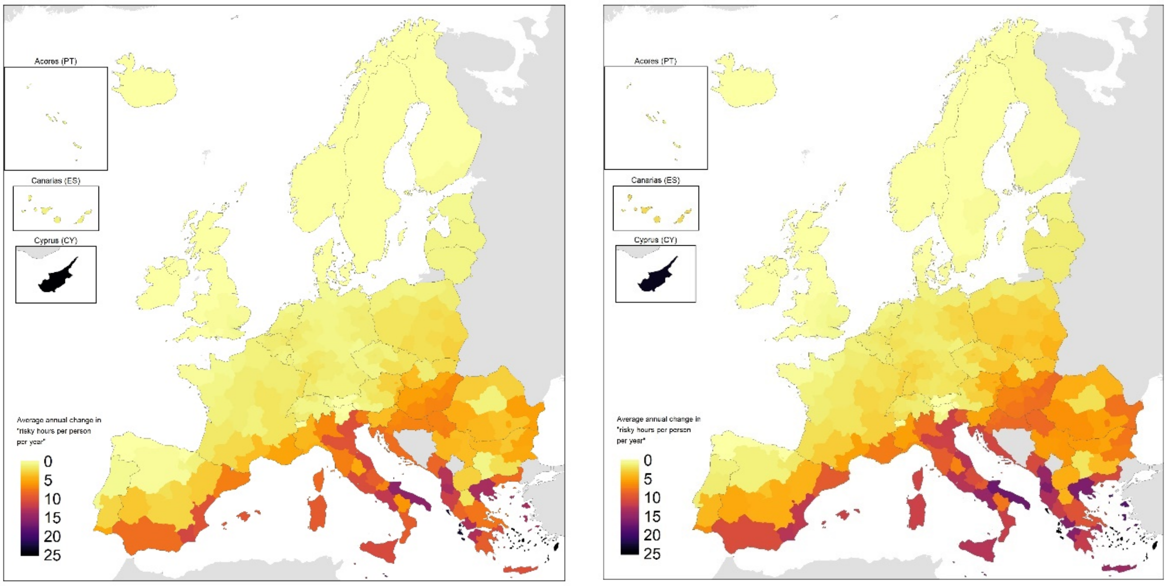

Figure 1.7 Average annual change in risky hours per person per year (1990-2020), for medium intensity (left panel) and strenuous (right panel) physical activities for NUTS 2 regions.

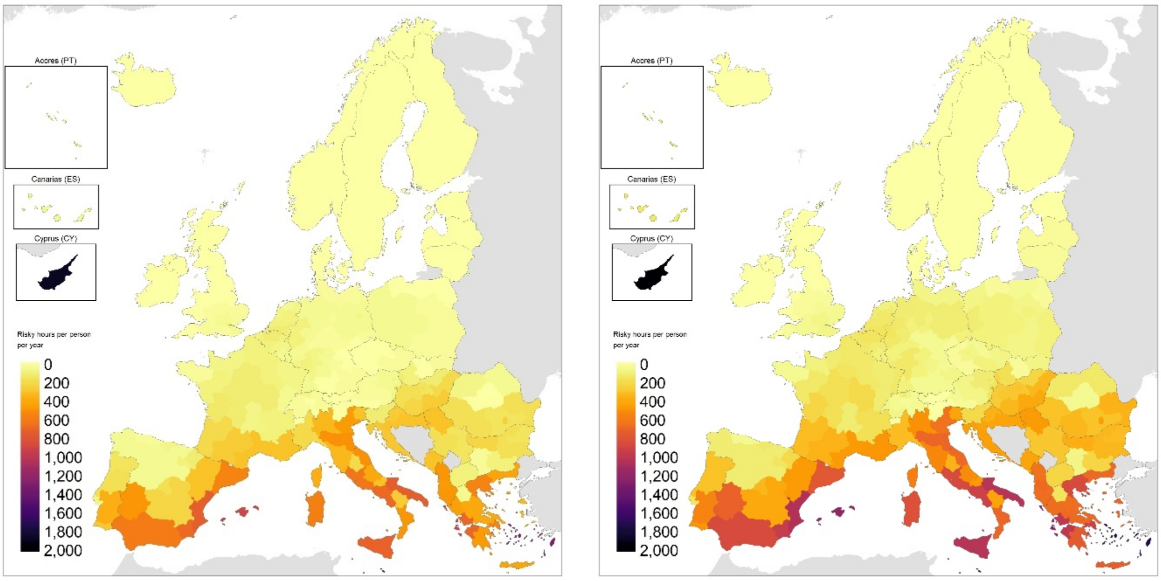

Figure 1.8 Risky hours per person per year for the year 2020, for medium intensity (left panel) and strenuous (right panel) physical activities for NUT2 regions.

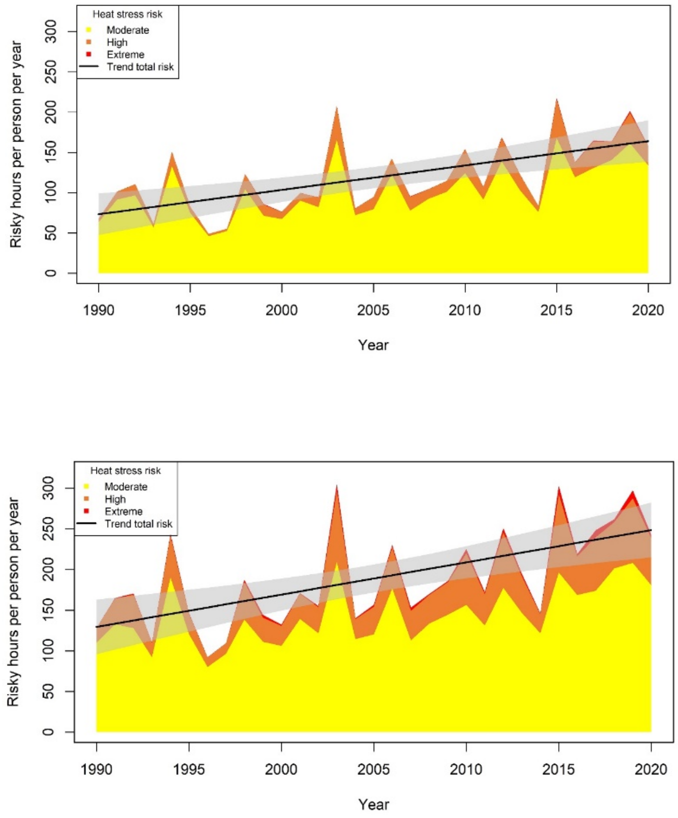

Figure 1.9 Risky hours per person per year, by risk level, for medium intensity (top panel) and strenuous (bottom panel) activities, for Europe (1990-2020). *The trend line shows total risk as the sum of moderate, high and extreme risk; the shaded area around the trend line shows the 95% confidence interval.*

Indicator 1.1.4: Heat-related mortality

Methods

First, a quasi-Poisson regression model, allowing for over dispersed death counts, is used to characterise the temperature-mortality relationship in each European region. Specifically, the model equation includes a natural cubic spline of time with 8 degrees of freedom per year to control for the seasonal and long-term trends, and a cross-basis function from a Distributed Lag Non-Linear Model to estimate the exposure-lag-response association between weekly temperatures and mortality counts.[19] The exposure-response function of the cross-basis is modelled with a natural cubic spline with three internal knots at the 10th, 75th and 90th percentiles of local weekly temperature distribution, and the lag-response function with integer lag values of 0, 1, 2 and 3 weeks.[20]

Second, a multivariate multilevel meta-analysis is performed with region-specific coefficients obtained in the first step to derive the best linear unbiased predictions of the temperature-mortality relationship in each region,[21] which are then used to calculate the annual number of deaths related to heat.[22] Heat weeks are defined as those with average temperatures above the local minimum mortality temperature. Annual heat related deaths are computed for the period 2000-2020, and given that mortality counts are not available in some countries for some of these years (see section *Data* below), the related mortality is estimated by using the mean annual cycle of mortality counts in each region.[23]

Finally, the heat related incidence is defined as the heat related number per million inhabitants. A linear trend is then fitted to the yearly time series of heat related incidence; whose slope represents the indicator expressed as annual deaths per million inhabitants per decade.

Geographic coverage of Europe

For this indicator, we included the European Environment Agency (EEA) member and cooperating countries plus the United Kingdom, excluding Turkey, Bosnia and Herzegovina, Kosovo and North Macedonia as data was not available for these countries.

Data

<u>Mortality data</u>. Weekly mortality counts by 5-year age group are obtained from Eurostat.[24] The dataset includes over 78 million counts of death from 990 contiguous regions representing 527 million Europeans in 35 countries. Weekly mortality counts are available for the period:

1. 2000-2020 in Austria, Belgium, Bulgaria, Croatia, Estonia, Spain, Finland, Germany, Hungary, Iceland, Latvia, Liechtenstein, Luxembourg, Norway, Poland, Portugal, Serbia, Slovakia, Slovenia, Sweden and Switzerland;
2. 2005-2020 in the Czech Republic and Montenegro;
3. 2007-2020 in Denmark;
4. 2010-2020 in Lithuania;
5. 2011-2020 in Italy and Malta;
6. 2013-2020 in France;
7. 2015-2020 in Albania, Cyprus, Greece, the Netherlands, Romania and the United Kingdom; and
8. 2019-2020 in Ireland.

Mortality counts for Ireland are not available by age group.

Temperature data. Weekly temperature data is derived from the ERA5-Land reanalysis, freely available in near real time at a 9km^2 grid resolution.[13] Gridded temperature data is transformed into regional average estimates by weighting the values with gridded population counts for the year 2011 from Geostat.[15]
Population data. The related incidence is calculated by using the regional population estimates from Eurostat.[25]

Caveats

The standard approach in timeseries studies of the health impacts of ambient temperatures is to calculate epidemiological models between daily temperatures and counts of death.[26] Parallel analyses using daily mortality data from a large ensemble of European regions showed that annual heat related mortality incidences are systematically underestimated in the weekly data model (manuscript in preparation). These biases are however constant throughout the years, and therefore, the linear trends of annual heat related mortality incidences from the weekly data model are not biased. This result motivated the current definition of the indicator as the trend of the yearly timeseries of heat related mortality incidence, and not based on the annual values themselves.

Future form of the indicator

In future versions of the report, the indicator can be further refined by using daily mortality data for contiguous European regions to calibrate predictive models and bias-correct the annual values of the heat related mortality incidence from weekly data models. The work is currently being done within the framework of the project EARLY-ADAPT.[27] The indicator is also planned to be stratified by sex.

Additional analysis

All the available mortality data were used to calculate the epidemiological models for the indicators shown in figure 1 (main text) and figure 1.10. Additional analyses were done in order to validate that the indicator is not sensitive to the heterogeneity of mortality data periods among countries (see section *Data* above). On the one hand, to suppress the effect of the record-breaking 2003 heat wave[28] in the Western European countries for which we have data, figure 1.11 shows the indicator for the general population in 2000-2020 when only data from 2004-2020 is used to calculate the epidemiological models. On the other hand, to suppress the effect of eventual (mal)adaptation processes (i.e. changes in relative risk),[29,30] figure 1.12 shows the same indicator when only data from 2015-2020 is used in the epidemiological models. Both sensitivity analyses show that the indicator does not depend on the period of data used to calculate the epidemiological associations, although the shorter is the period the larger are the uncertainties of the models.

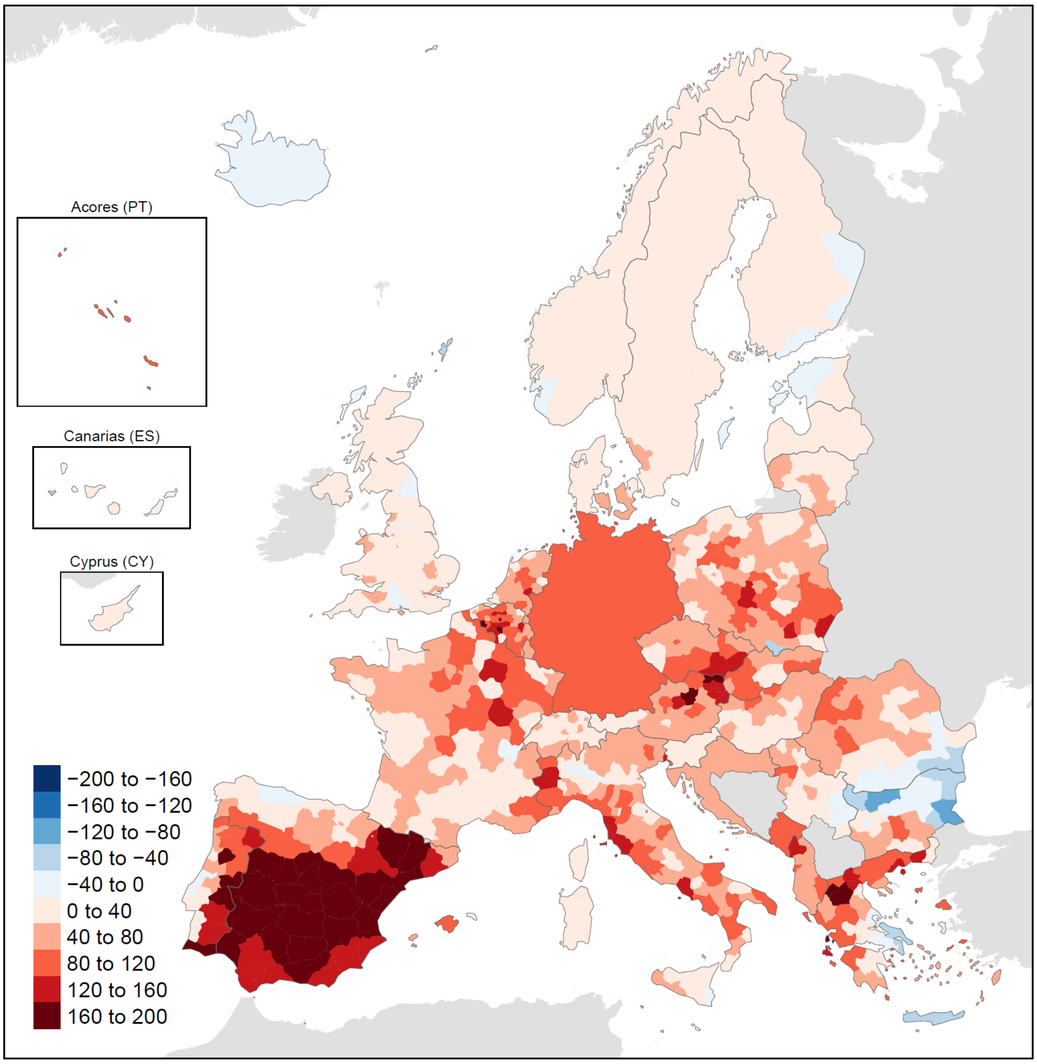

Figure 1.10 Trends in heat related mortality incidence (annual deaths/million/decade) for the elderly (65years and over), 2000-2020.

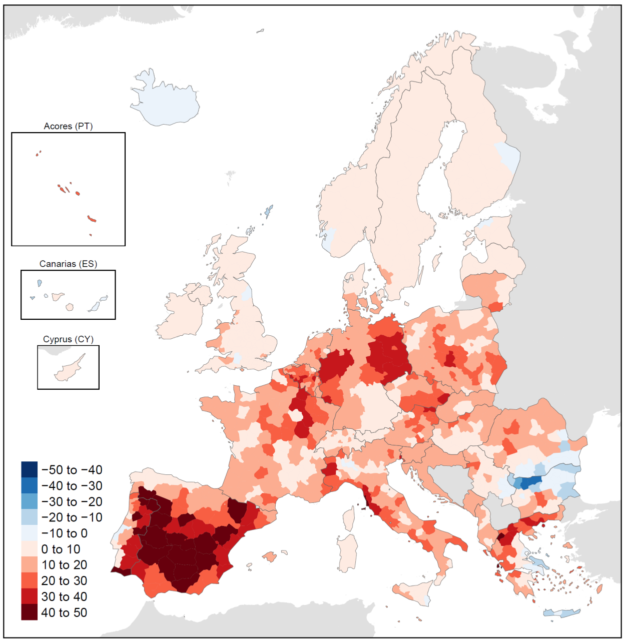

Figure 1.11 Trends in heat related mortality incidence (annual deaths/million/decade) for the general population, 2000-2020

To suppress the effect of the record-breaking 2003 heat wave in the Western European countries for which we have data, this figure shows the indicator when only data from 2004-2020 is used to calculate the epidemiological models.

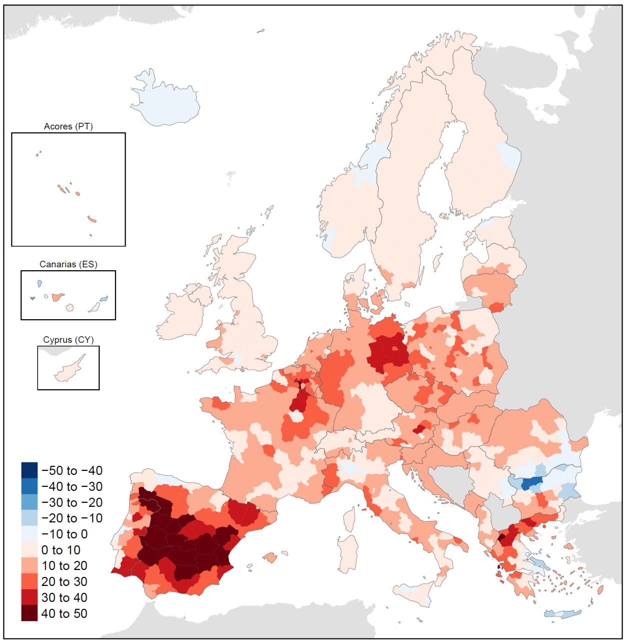

Figure 1.12 Trends in heat related mortality incidence (annual deaths/million/decade) for the general population, 2000-2020

To suppress the effect of eventual (mal)adaptation processes (i.e., changes in relative risk), this figure shows the indicator when only data from 2015-2020 is used to calculate the epidemiological models.

1.2: Health and extreme events

Indicator 1.2.1: Wildfire smoke

Data

The input data for this indicator is as follows:

1. GEOSTAT population grids for Europe in polygon format. The spatial resolution of the dataset is 1km, and the available years are 2006, 2011, 2018.
2. Eurostat GISCO NUTS boundaries for Europe at a scale 1:1M. The 2021 edition was used since regional mortality data was only available for the NUTS 2021 edition. NUTS 2 level was used for all countries except for Croatia, Germany, Ireland, and Slovenia, whose mortality data was only available at the NUTS 1 level.
3. Eurostat regional weekly total death counts for the study period 2003-2020.
4. Daily $PM_{2.5}$ – all-cause mortality exposure-response linear function (RR: 1.0065; 95% CI: 1.0044–1.0086 for a 10µg/m^3 $PM_{2.5}$ increase) extracted from a meta-analysis by Orellano *et al.* 2020.[31]
5. Finnish Meteorological Institute fire smoke dispersion forecasts ($PM_{2.5}$ concentrations) derived from the Integrated System for wild-land Fires (IS4FIRES) and the System for Integrated modelling of Atmospheric composition (SILAM) models.[32] The spatial resolution of the product is 0.1° (~10km) and the temporal resolution is 1h. A complete description of the fire system can be found in Sofiev *et al.* 2009[33] and Soares *et al.* 2015.[34] Briefly, the system is based on MODIS fire radiative power products MOD14/MYD14, which are available globally with 1-9 km^2 spatial resolution depending on the particular overpass and the instrument viewing angle. The released radiative energy is converted to smoke emissions using empirical emission factors. Emissions are then used by the SILAM atmospheric composition model for computing plume dispersion, chemical transformations, and deposition. Outputs from this model have been previously used to estimate the health burden of wildfire smoke in Europe.[35]
6. Fire weather index (FWI) computed from ERA5 climate reanalysis data for the period 1981-2020 with a 1° spatial resolution. Briefly, FWI is a unitless fire danger index based on meteorology data that estimates potential fire intensity based on fuel availability (i.e., drought and moisture conditions) and fire spread (i.e., wind conditions).

Geographic coverage of Europe

For this indicator we included the European Environment Agency (EEA) member and cooperating countries plus the United Kingdom subject to exposure, population, and mortality data availability (see below)

Methods

Three sub-indicators were constructed for the period: yearly average wildfire-$PM_{2.5}$ exposure (exposure sub-indicator 1), yearly number of days where daily population-weighted wildfire-PM2.5 exceeded 1µg/m^3 (exposure sub-indicator 2), and yearly attributable mortality to wildfire-$PM_{2.5}$ exposure (health impacts sub-indicator) for regions that met the following criteria:

- Exposure sub-indicators: To be included in the NUTS 2021 classification, to have gridded population data for at least a subset of the study period, and to have exposure data. Following these criteria, overseas

territories, Atlantic Islands (Canary Islands, Madeira, Azores), and several European countries/regions (Bosnia and Herzegovina, Kosovo, Turkey) were excluded from the sub-indicator.

- Health impacts sub-indicator: The same criteria for the exposure indicator applied, with the additional requirement of having weekly mortality time series data for at least a subset of the study period. Following this criterium, North Macedonia was further excluded from the sub-indicator.

The workflow to construct the indicators was as follows:

1. Hourly wildfire-$PM_{2.5}$ concentrations were aggregated to daily averages.
2. GEOSTAT gridded population data (2006, 2011, 2018) in polygon format (1km spatial resolution) were transformed into a regular raster matching the exposure grid (0.1° spatial resolution). Aggregation was performed via addition of GEOSTAT population counts whose polygon centroids fell within the larger exposure cell. No population estimates were available for a set of countries (Albania, Croatia, Cyprus, Montenegro, North Macedonia and Serbia) at the 2006 edition, which we imputed with data from 2011.
3. NUTS codes were assigned to each of the exposure-population grid cells with a spatial join via intersection. If a given cell intersected with more than one NUTS unit, the one with the largest overlap was taken. Country codes and regional indicators (South/Central/North/West Europe) were derived from the NUTS codes.
4. Daily exposures, spatial units and population indicators grids were transformed into a tabular format for further analysis. Since population data was only available for three years, population data from the nearest available year was assigned to the missing years without doing any interpolation.
5. EUROSAT weekly mortality data were disaggregated into daily counts (i.e., the temporal resolution of the exposure and the exposure-response function) by assigning an equal number of deaths to each day of the week, i.e. by dividing the weekly mortality counts by 7.
6. Daily population-weighted exposures at NUTS 2 level were calculated by first computing population weights adding up to 1 within each NUTS unit, and then computing a weighted average of the exposures. Daily NUTS 2 population-weighted $PM_{2.5}$ exposure datasets were aggregated to years 1) by averaging and 2) by counting the number of times daily exposures exceeded 1µg/m^3. Yearly average population-weighted $PM_{2.5}$ at the country and regional level were computed using the same approach.
7. To compute yearly attributable mortality, the following steps were taken following a comparative Health Impact Assessment framework:
   a) The point estimate Relative Risk (RR) (RR: 1.0065 per 10µg/m^3 $PM_{2.5}$ increase) was scaled to the exposure in each daily exposure cell by applying the following transformation where *i, j* and *t* indices refer to the grid cell, NUTS and day indicators, respectively; and *exposure* refers to the actual exposure for a grid cell-day:

   $$\mathrm{RR_{ijt}} = \exp\left(\ln(1.0065) * \frac{\mathrm{exposure}_{ijt}}{10}\right)$$

   b) Daily Population Attributable Fractions (PAF) were computed at the NUTS 2 level using standard HIA methods[36] for a null counterfactual exposure of 0µg/m^3 and the scaled relative risks from the previous step. Population weights p_{ijt} were computed as per step 6:

   $$\mathrm{PAF_{jt}} = \frac{\sum_{i=1}^{n} \mathrm{p_{ijt}RR_{ijt}} - 1}{\sum_{i=1}^{n} \mathrm{p_{ijt}RR_{ijt}}}$$

c) Daily NUTS 2 PAF data were merged with daily mortality counts at the same temporal and spatial scale and were multiplied to obtain the daily attributable mortality to wildfire-$PM_{2.5}$ exposure at the NUTS 2 level. We summed the counts to form yearly aggregates for the NUTS 2 regions with complete data for a given year. Counts were further added at the country level to form national estimates.

d) Confidence Intervals (CI) of the yearly attributable deaths were calculated by propagating the uncertainty of the exposure-response function via Monte Carlo simulation.[37] Briefly, we assumed the natural logarithm of the RR to come from a normal distribution with mean equal to the natural logarithm of the point estimate and Standard Deviation (SD) computed from the CI (RR CI: 1.0044–1.0086 per 10μg/m^3 $PM_{2.5}$ increase) as:

$$SD = \frac{\ln(\text{upper}) - \ln(\text{lower})}{\text{qnorm}(0.975) * 2}$$

Then, 200 realisations of that distribution were simulated and exponentiated to obtain 200 realisations of the RR. With them, steps 7a-c were repeated to have 200 estimates of the yearly attributable mortality at the NUTS 2 and country level. 95% CI were computed by taking the percentiles 0.025 and 0.975 of the attributable mortality distribution per year and spatial unit.

As an additional analysis included in the main visualization of the indicator, linear trends in regional (Western, Southern, Northern, Central and Eastern Europe) annual population-weighted wildfire-$PM_{2.5}$ exposure were computed by fitting a linear model per region with the yearly exposure as a dependent variable and the year as an independent variable. The slope coefficients (95% CI) of the linear regressions were used as trend estimates. Statistical significance of the trends was assessed via p-values of the slope coefficients under a null hypothesis of beta=0; in all four regions p-values were >0.1. Furthermore, we computed annual average FWI by European region and computed linear trends using the same methodology; in all regions p-values were >0.1.

Two sensitivity analyses were performed:

1. Annual average wildfire-$PM_{2.5}$ exposure by European region without population weighting, i.e., a purely spatial mean giving all pixels within region the same weight, was computed to verify whether proximity of population and fire events played a role in the observed trends. Similar to main results, this analysis showed weak and uncertain downward exposure trends in all regions (Figure 1.16), which suggests that the location of wildfires and population within region do not play a major role in the observed trends.
2. An alternative exposure-response function was used to examine the potential increased toxicity of wildfire smoke compared to total $PM_{2.5}$ mass concentration.[38] The chosen estimate came from a global multi-city time series study which examined the association between wildfire-$PM_{2.5}$ and all-cause mortality;[39] we used the global pooled relative risk (lag0) reported therein: 1.021 (95% CI 1.018–1.024) for 10 μg/m^3 increase of wildfire-$PM_{2.5}$. When using this estimate, the number of annual attributable deaths more than tripled (table 1.2).

The R code used to construct this indicator and create the visualizations included in this publication is available in the following repository: https://github.com/carlesmila/LCD-Europe-wildfires

Caveats

1. The list of caveats for the indicator is as follows:
2. GEOSTAT population grids included a limited set of years, and therefore assumptions had to be made for years where gridded population data was not available. Furthermore, no population estimates were available for a set of countries (Albania, Croatia, Cyprus, Montenegro, North Macedonia and Serbia) at the 2006 edition, which had to be imputed with data from 2011.
3. NUTS 2 regions were used in our analyses, except for Croatia, Germany, Ireland and Slovenia, which were analysed at the NUTS 1 level due to mortality data availability at that spatial scale.
4. The temporal resolution of the Eurostat mortality time series (weekly) was different than the temporal resolution of the exposure and the exposure-response function (daily). As a result, an equal number of deaths across the 7 weekdays had to be assumed to be able to estimate the health effects. Furthermore, the temporal availability of the weekly death count data varied widely across countries.
5. There is still currently no widely accepted exposure-response function specific to wildfire-$PM_{2.5}$. The epidemiologic body of literature linking wildfire smoke and health effects is still limited, particularly for European populations; and heterogeneous, with varying exposure assessment methods between studies. The exposure-response function used in the indicator assumes a similar toxicity and exposure range of wildfire $PM_{2.5}$ and $PM_{2.5}$ from other sources, which evidence suggests may not be true.[38]
6. Regarding the fire emission system, to-date, MODIS active fire counts and fire radiative energy products are arguably the best source of fire information worldwide. However, as every low-orbit satellite, MODIS suffers from omission errors. These have two causes: (i) cloud obscuration, including the fire obscuration by own smoke plumes, (ii) limited sensitivity of the instrument causing omission of small fires.[40] Sofiev, *in preparation* suggest that the omission error varies widely depending on the region and season, being close to ~20-30% for European regions during the local fire seasons. For clear-sky retrievals, the detection limit depends on the viewing angle and time of the day. At night, sub-satellite fires down to 4 MW are detectable, whereas during day at the edge of the viewing area, burns up to 40 MW are non-detectable.

Future form of the indicator

In order to overcome the limitations of the exposure-response function, new epidemiologic studies and meta-analyses will provide more robust estimates of the association, as well as possible effect modifiers. Once they become available, exposure-response functions will be modified accordingly. Subject to resources, European-specific exposure-response functions will be estimated in an epidemiological study, including weekly exposure-response functions that match the temporal resolution of the mortality data.

In order to rectify the fire omission problem, several options will be explored. Firstly, more satellites will be used, e.g., VIIRS and SLSTR, producing similar products but providing the data at different overpass times. Secondly, utilization of geostationary instruments, such as SEVIRI for Southern Europe, will be explored. Thirdly, the emerging technology of the fire data assimilation and fusion will allow for breakthrough improvements, essentially merging together the fire models and (incomplete) satellite observations.

Additional analysis

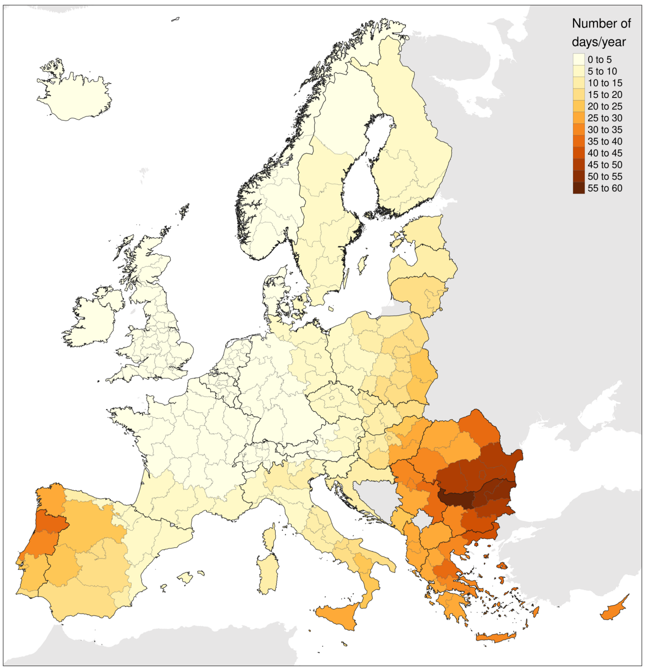

Figure 1.13 Average number of days per year where daily population-weighted wildfire-$PM_{2.5}$ exceeded 1µg/m^3 (percentile 0.968 of the daily wildfire-$PM_{2.5}$ distribution) for the period 2003-2020 by NUTS 2 region, except for Croatia, Germany, Ireland, and Slovenia, which were analysed at the NUTS 1 level due to mortality data availability.

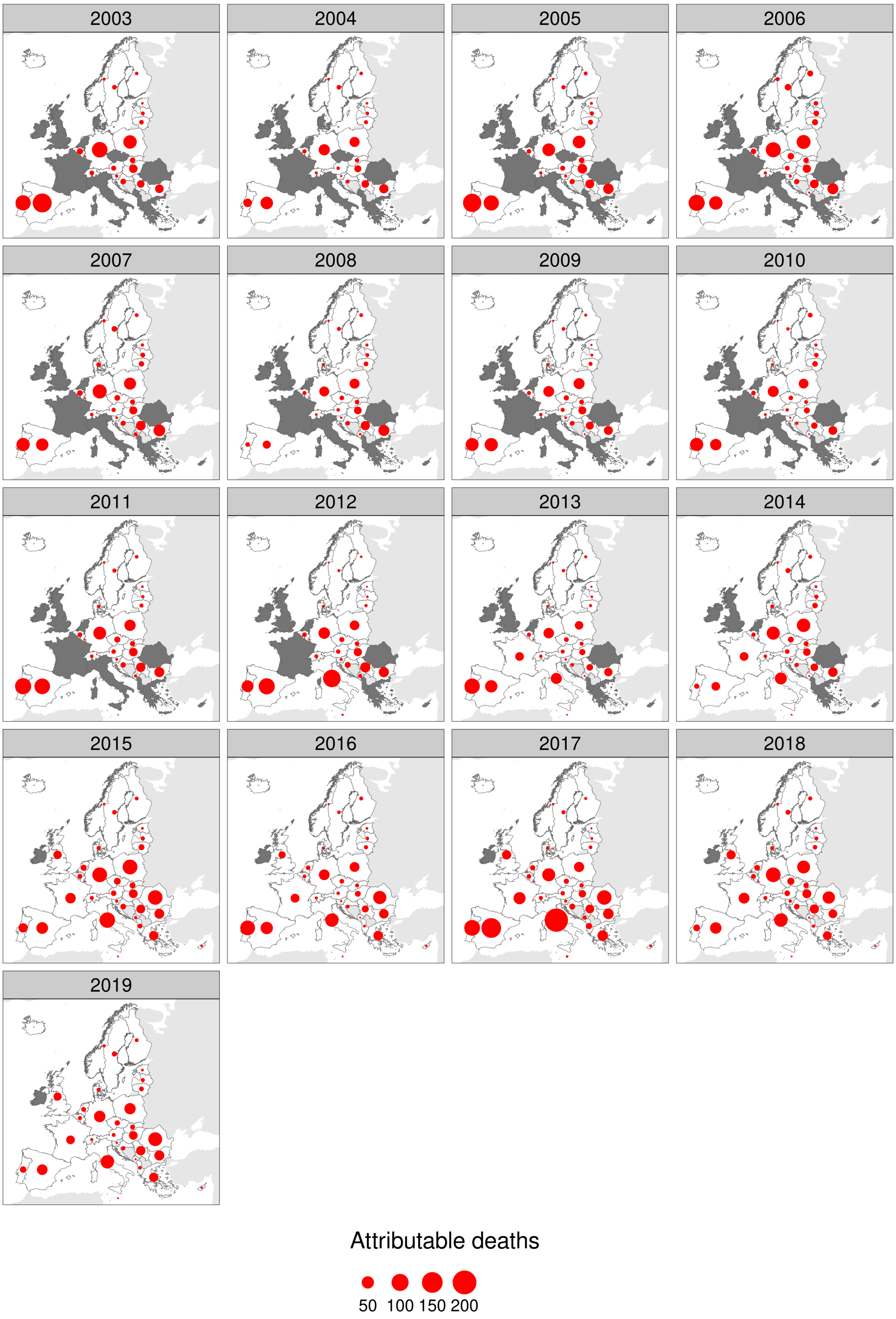

Figure 1.14 Yearly estimated attributable mortality due to wildfire-$PM_{2.5}$ exposure by country. Missing data (dark grey) are subject to mortality data availability for a given region and year. Only point estimates are shown (95% CI are omitted for visualisation purposes).

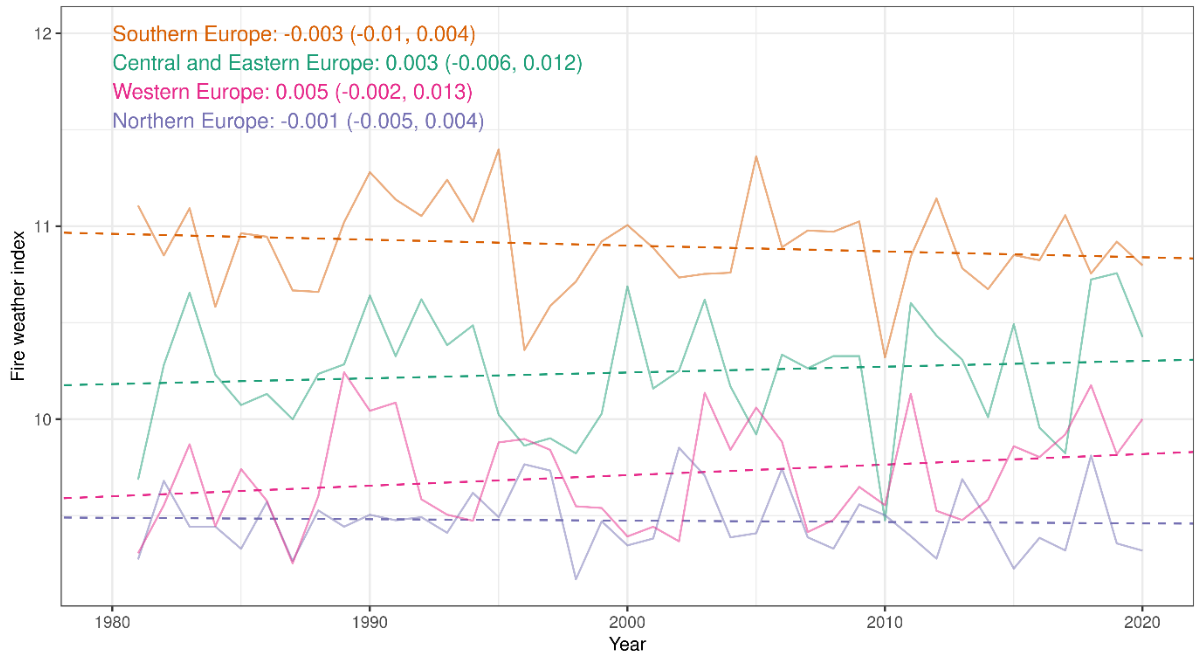

Figure 1.15 Annual average fire weather index by European region (bold) and linear trend (dashed) 1980-2020. Slope coefficients (95% CI) corresponding to the linear trend (index change per 1-year increase) are shown as text.

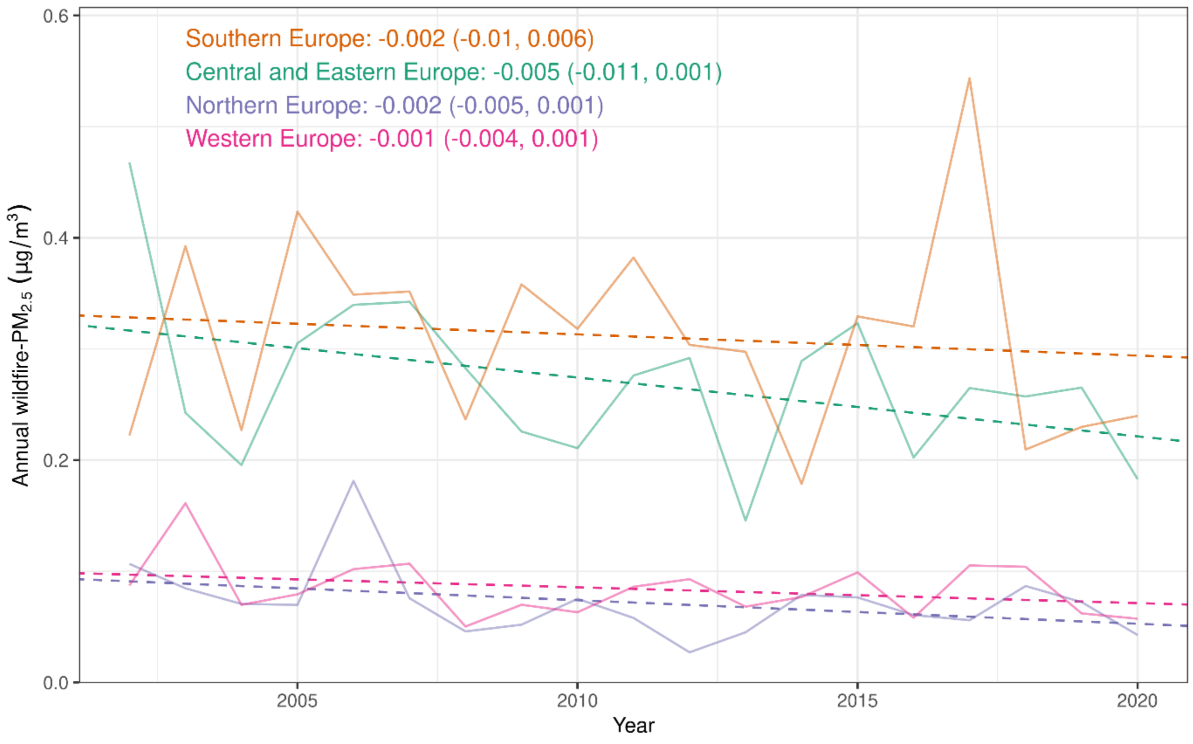

Figure 1.16 Annual average wildfire-$PM_{2.5}$ exposure by European region (bold) and linear trend (dashed) 2003-2020 without population weighting, i.e. pure spatial averaging within region. Slope coefficients (95% CI) corresponding to the linear trend (wildfire-$PM_{2.5}$ exposure change per 1-year increase) are shown as text.

Table 1.2 Annual Europe-wide estimated attributable deaths to wildfire-$PM_{2.5}$ according to the two exposure response functions used in the indicator. Note that the number of countries included in the estimate varies between years due to availability of weekly mortality data.

Year	Main analysis[1]	Sensitivity analysis[2]	Number of included countries
2003	474.14 (322.18, 635.39)	1520.12 (1306, 1747.76)	22
2004	246.54 (167.49, 330.45)	790.75 (679.44, 908.9)	22
2005	446.89 (303.73, 598.74)	1429.6 (1229.01, 1642.31)	22
2006	475.13 (323.2, 636)	1511.4 (1300.78, 1734.27)	24
2007	403.62 (274.62, 540.14)	1281.94 (1103.62, 1470.54)	25
2008	224.47 (152.51, 300.82)	719.35 (618.2, 826.68)	25
2009	304.29 (206.73, 407.85)	976 (838.59, 1121.88)	25
2010	267.65 (181.87, 358.65)	857.13 (736.71, 984.88)	25
2011	417.48 (284.03, 558.72)	1326.01 (1141.62, 1520.95)	25
2012	456.95 (310.44, 612.47)	1465.71 (1259.37, 1684.76)	27
2013	336.35 (228.58, 450.67)	1076.49 (925.36, 1236.79)	28
2014	351.48 (238.77, 471.15)	1128.46 (969.34, 1297.54)	28
2015	645.79 (438.78, 865.48)	2069.71 (1778.66, 2378.56)	34
2016	483.99 (328.86, 648.6)	1550.8 (1332.75, 1782.19)	34
2017	844.46 (573.79, 1131.68)	2705.37 (2325.17, 3108.7)	34
2018	556.2 (377.92, 745.4)	1782.33 (1531.74, 2048.22)	34
2019	484.14 (328.94, 648.87)	1552.03 (1333.71, 1783.72)	34

[1] The main analysis used a daily total mass $PM_{2.5}$ – all-cause mortality exposure-response linear function (RR: 1.0065; 95% CI: 1.0044–1.0086 for a 10μg/m^3 $PM_{2.5}$ increase) extracted from a meta-analysis by Orellano *et al.* 2020.[41]

[2] The sensitivity analysis used a daily wildfire-$PM_{2.5}$ – all-cause mortality exposure-response linear function (RR: 1.021; 95% CI: 1.018–1.024 for a 10μg/m^3 wildfire-$PM_{2.5}$ increase) extracted from multi-city global study by Chen *et al.* 2020.[39]

Indicator 1.2.2: Drought

Methods

SPEI6 helps to detect, monitor and analyse the drought severity, duration, and extent globally. It is a standardised index with no units, where positive values of SPEI6 correspond to conditions of wet periods whereas negative values correspond to dry periods. The drought severity classification is commonly done based on the negative SPEI values, as defined in table 1.3.

Table 1.3 Drought severity classification based on SPEI6 indicator.

SPEI6	Event description
-0.50 to -0.79	Abnormally dry
-0.80 to -1.29	Moderate drought
-1.30 and -1.59	Severe drought
-1.60 and -1.99	Extreme drought
less than -2.0	Exceptional drought

The computation of the SPEI6 index (as described in Vicente-Serrano *et al.*, 2010[42]) was done in two steps: accumulation and standardisation. First, the accumulation involves an estimation of monthly climatic water balance ($d_{i,j}$), which provides a measure of the water surplus or deficit for a specific month 'i' in the year 'j'. It is estimated as follows:

$d_{i,j} = P_{i,j} - PET_{i,j}$

where, P is the precipitation and PET is the Potential Evapotranspiration. The computed $d_{i,j}$ for each month is then accumulated over a period of 6 months for the years 1951 to 2020 ($A6_{i,j}$).

$A6_{i,j} = d_{i,j} + d_{i-1,j} + d_{i-2,j} + \dots + d_{i-5,j}$

The standardisation step fits the $A6_{i,j}$ to a suitable parametric probability distribution and then transforms the data into a standardised series (with mean = 0 and standard deviation = 1), where the standardised value is referred to as SPEI6 (in which the number 6 corresponds to the total number of accumulated months). SPEI6-September, where the monthly climate water balance values of April to September are accumulated, is used to study the extended summer drought conditions and SPEI6-March that accumulates climate water balance values from October to March is used to study the extended winter drought condition. In this report, the spatially averaged results are presented for European NUTS 2 level regions. When computing the index for NUTS 2 level regions, the accumulated climate water balance ($d_{i,j}^{SPEI1}$) values were aggregated prior to standardisation step, as described in Solaraju-Murali *et al.*, 2021.[43]

The Hargreaves method is chosen for estimating PET in this assessment.[44] The parameterization used for estimating PET using this approach is based on monthly maximum and minimum temperatures and latitude coordinates, the latter variable being used to calculate the maximum amount of sunshine duration. Following this, the three-parameter shifted log-logistic probability distribution function was used to build the distribution and the parameters were computed using the method of unbiased probability weighted moments. The choice of the

parametric probability distribution was verified and the assumption is shown to be valid over large parts of Europe (for e.g. Stagge et al 2015,[45] Beguería *et al.*, 2014[46]). SPEI6 is calculated using the R-package SPEI (https://cran.r-project.org/web/packages/SPEI/index.html).

Geographic coverage of Europe

For this indicator, we included the 27 European Union countries plus the United Kingdom, European Free Trade Association (EFTA) countries (Iceland, Liechtenstein, Norway, Switzerland) and EU candidate and potential candidate countries (Montenegro, North Macedonia, Albania, Serbia, Turkey).

Data

The monthly maximum and minimum temperature and precipitation from the ERA5-Land Reanalysis product (Muñoz Sabater, 2019; 2021)[13] have been used to estimate SPEI6. The data cover the period from 1950-present at a spatial resolution of 9km^2 globally. ERA5-Land is produced and updated by the European Centre for Medium-Range Weather Forecasts (ECMWF) within 3 months of real time.

Caveats

While SPEI is a commonly used indicator in drought assessment studies, it has some known limitations. The best approach for estimating PET and the choice of a particular parametric probability distribution for computing the indicator still remains an open question at all timescales especially when working on regional-to-global spatial domains. Several studies[45,46,47] have pointed out that different choices lead to slightly varied estimates of drought frequency and severity in some regions over Europe. Furthermore, the aggregation of the SPEI6 indicator at NUTS 2 level implies a smoothing of the extreme values, which could lead to an underestimation of the frequency and severity of the drought conditions over several regions.

Future form of the indicator

Future iterations will include the combination of this drought index, obtained directly from temperature and precipitation, with socio-economic datasets at European level linked with impacts in society (e.g. food production, health issues or disasters).

Additional analysis

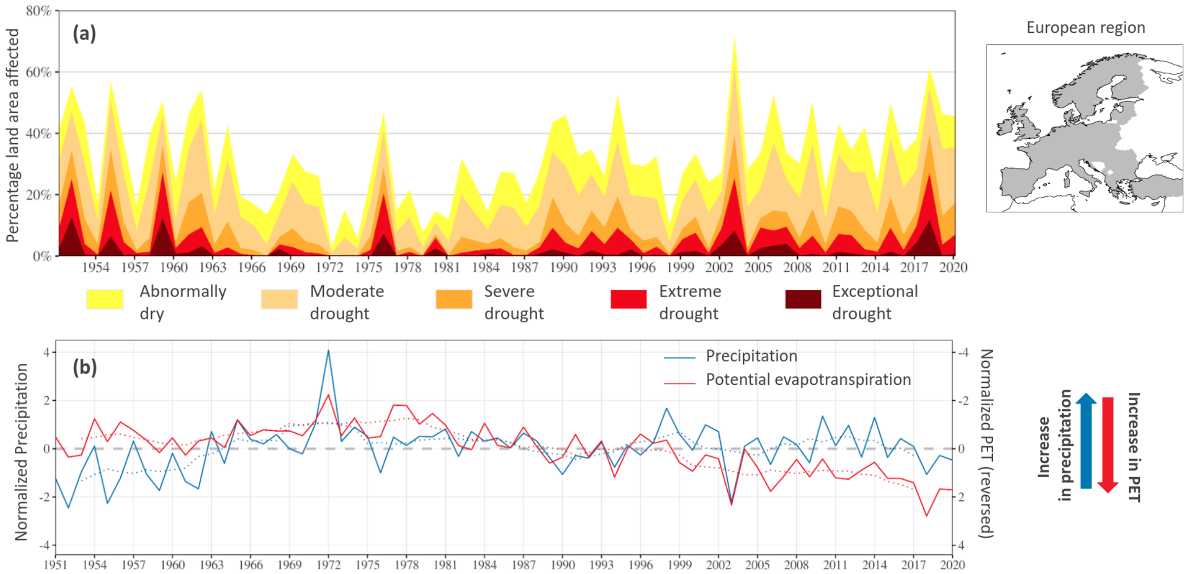

Figure 1.17 (a) Total percentage of land area affected by droughts (SPEI6 < -0.5) over Europe during the extended summer months (April to September) for the period 1951-2020. For each year, five categories representing the severity of the drought are shown (see table 1.3 for definition of the categories). The domain considered for the land area estimation is displayed on the right-side map in grey. (b) Time series of normalized six-month (April to September) accumulated precipitation (potential evapotranspiration) over Europe is presented in thick blue (red) line for the period 1951-2020. The corresponding blue (red) dotted line corresponds to the 5-year moving window average. The years in which values of accumulated precipitation or potential evapotranspiration are negative corresponds to anomalous years with values inferior to climatological mean of the respective variable. Then, for each year, the variable with the highest negative value could potentially be responsible for the drought presented in panel (a).

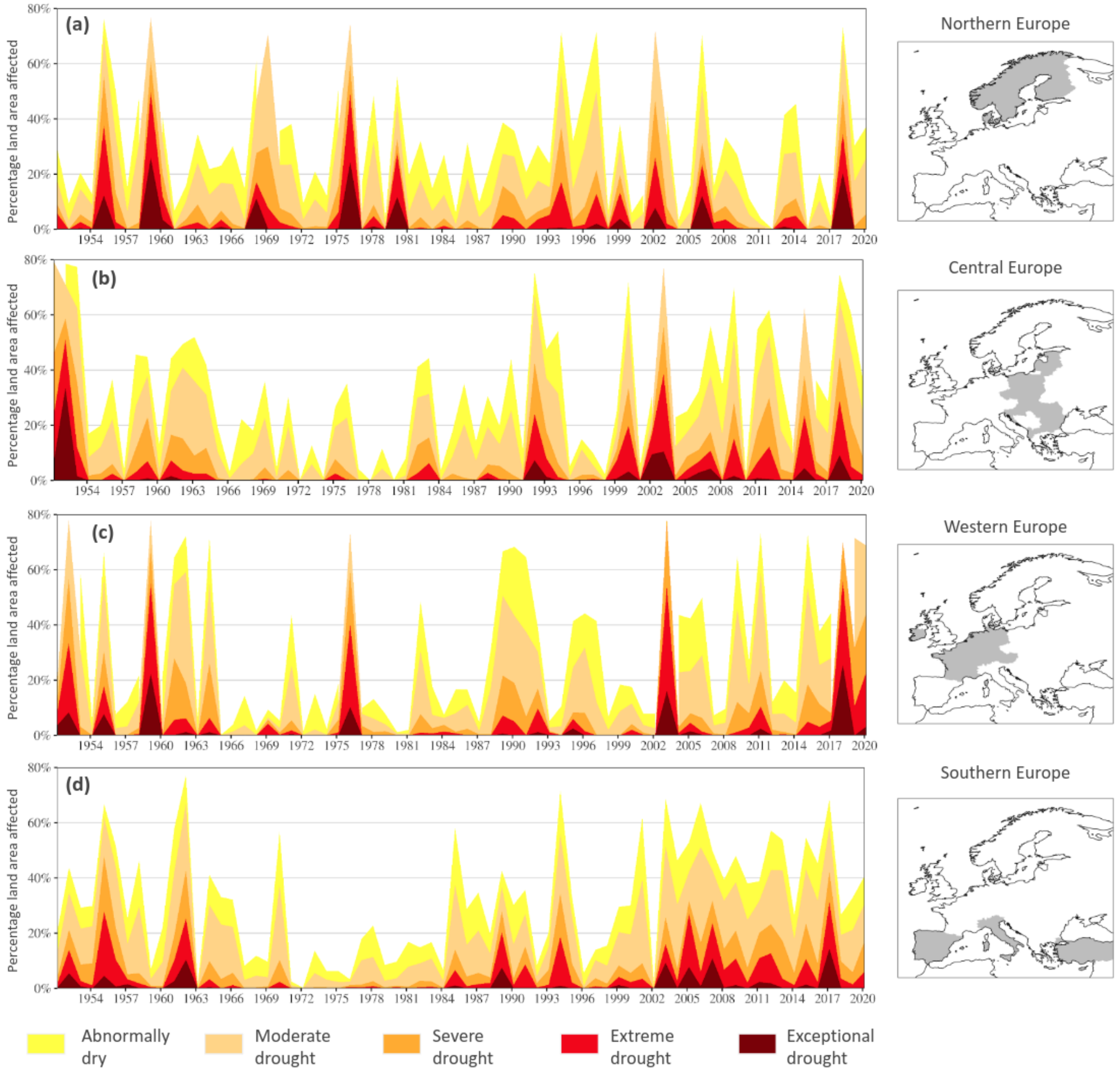

Figure 1.18 Total percentage of land area affected by droughts (SPEI6 < -0.5) during the extended summer (April to September) for the period 1951-2020 over (a) Northern, (b) Central, (c) Western and (d) Southern Europe. For each year, five categories representing the severity of drought are shown (see table 1.3 for definition of the categories). The spatial domain considered for the area estimation is displayed on the right-side map in grey.

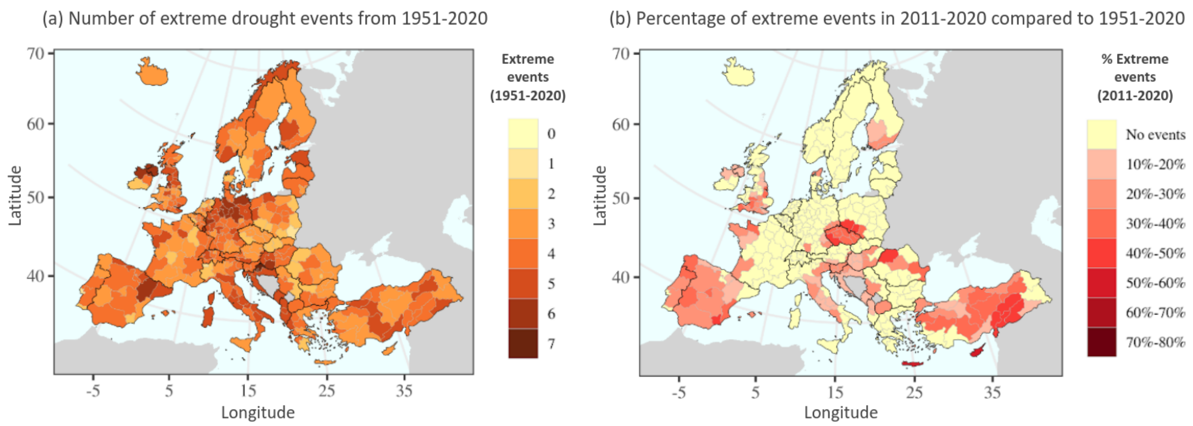

Figure 1.19 (a) Total number of extreme drought events (SPEI6 < -1.6) for extended winter (October to March) over Europe for the period 1951-2020 and (b) the percentage of extreme events that occurred in the years 2011-2020 in comparison to 1951-2020.

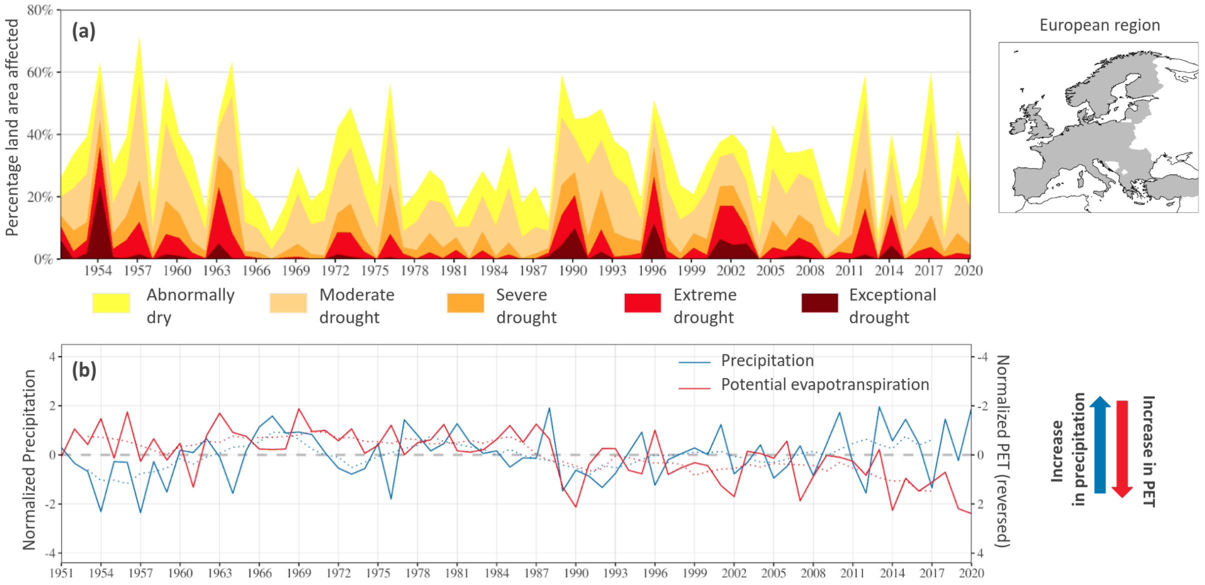

Figure 1.20 (a) Total percentage of land area affected by droughts (SPEI6 < -0.5) over Europe during the extended winter months (October to March) for the period 1951-2020. For each year, five categories representing the severity of the drought are shown (see table 1.3 for definition of the categories). The domain considered for the land area estimation is displayed on the right-side map in grey. (b) Time series of normalized six-month (October to March) accumulated precipitation (potential evapotranspiration) over Europe is presented in thick blue (red) line for the period 1951-2020. The corresponding blue (red) dotted line corresponds to the 5-year moving window average. The years in which values of accumulated precipitation or potential evapotranspiration are negative corresponds to anomalous years with values inferior to climatological mean of the respective variable. Then, for each year, the variable with the highest negative value could potentially be responsible for the drought presented in panel (a).

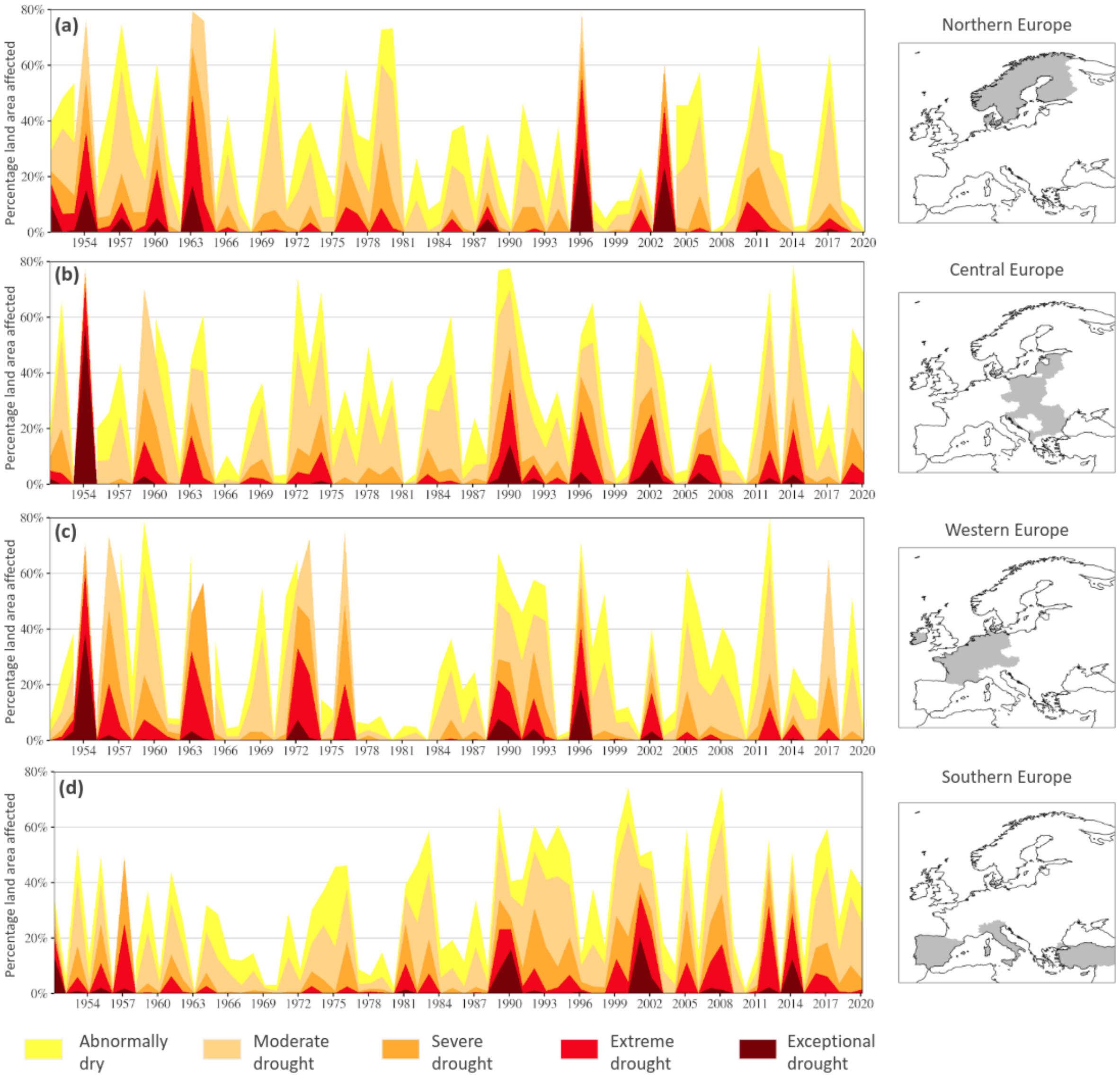

Figure 1.21 Total percentage of land area affected by droughts (SPEI6 < -0.5) during the extended winter (October to March) for the period 1951-2020 over (a) Northern, (b) Central, (c) Western and (d) Southern Europe. For each year, five categories representing the severity of drought are shown (see table 1.3 for definition of the categories). The spatial domain considered for the area estimation is displayed on the right-side map in grey.

1.3: Climate-sensitive infectious diseases

Indicator 1.3.1: Climate suitability *Vibrio*

Methods

We provide three indicators for the period 2003-2020: i) time series with the percentage coverage of the shoreline at the country level, ii) temporal presence of areas suitable for Vibrio at the country level, and iii) number of estimated cases of Vibriosis at the country level. Additionally, and as side products, we estimate the spatial distribution of areas at risk at daily, weekly and monthly scales. These fields will be available through Web Services for general access.

The steps in the workflow are the following:

1. Collect the sea surface temperature (SST) and sea surface salinity (SSS) fields from the respective sources (NASA and EU Copernicus, respectively). We change the data resolution of the SSS dataset to match the resolution of SST.
2. Convert the GEOSTAT population data to netCDF format, and regrid it using spatial densities to 0.01deg resolution.
3. Estimate the Vibrio Suitability Index using SST and SSS as inputs. The thresholds used were 18ºC for SST and 28psu for SSS.[48]
4. Following Trinanes, Martinez-Urtaza, 2021,[49] we estimate the population potentially affected by *Vibrio* infections within a distance of 100km from the *Vibrio* suitability areas. We use a conservative infection rate[50] to estimate the number of cases. These cases are summarised by country and year in the third component of the indicator.
5. To obtain Indicator part i, we use the daily *Vibrio* suitability fields to get the extent of shoreline affected. These results are aggregated by country into monthly and annual values.
6. The suitability fields also give us the opportunity to assess the presence of risk and evaluate its temporal distribution (Indicator part ii). These results are also aggregated by country for the period of study.

Geographic coverage of Europe

For this indicator we included the 27 European Union countries plus the United Kingdom, European Free Trade Association (EFTA) countries (Iceland, Liechtenstein, Norway, Switzerland) and the Western Balkans (Albania, Bosnia and Herzegovina, Kosovo, Montenegro, North Macedonia and Serbia).

Data

The input data for this indicator is as follows:

1. GEOSTAT population gridded data for Europe. The spatial resolution of the dataset is 1km^2. The dataset is available for years 2006, 2011 and 2018.
2. GHRSST Level 4 MUR Global Foundation Sea Surface Temperature Analysis (v4.1) at 1km resolution. This dataset is available on a daily basis from 2003-present. This dataset integrates temperature retrievals from multiple satellite sensors and in-situ data and provides a unique high-resolution and daily-consistent view for the coastal region.

3. Sea Surface Salinity data from the Ocean Physical Reanalysis and Analysis models provided by EU Copernicus Marine Service. The multi-source salinity inputs were pre-processed to fit the SST dataset in temporal and spatial resolutions.

Caveats

1. The infection rate applied here is based on the published data for the USA.[50] A more accurate infection rate adjusted to the epidemiological situation in Europe would be convenient to obtain a more precise picture of the impact of Vibrio illness across Europe. Additionally, a more realistic estimate of infection rate needs also to consider limitations of surveillance data and under-reporting, which has been widely reported worldwide. In Trinanes, Martinez-Urtaza, 2021[49] we corrected the estimated number of cases by applying the under-reporting ratio for the USA of 143[51]. However, an estimate of the underreporting in Europe would be also necessary. The current form doesn't take into account regional/national, socio-economic, and cultural conditions.
2. The population datasets show gaps for certain non-landlocked countries in the European continent.
3. The indicators are provided at NUTS 0 level.
4. The ocean parameters, more importantly SSS, show certain limitations in the coastal region, as models do not usually provide an accurate picture of SSS variability under heavy rainfall and/or river runoff and/or ice melting. This might be mitigated by an improved in-situ observing network in the coastal areas, and enhanced model data assimilation schemes.

Future form of the indicator

Advances in modelling, remote sensing and social/economic and environmental data collection might allow us to improve these indicators, by providing better spatial resolution, accuracy and an opportunity to develop enhanced exposure functions. Access to epidemiological data is a key element for further validating/improving these indicators. We intend to use the indicators for operational applications and improved informed decision-making and to produce the indicators at a finer spatial resolution.

Additional analysis

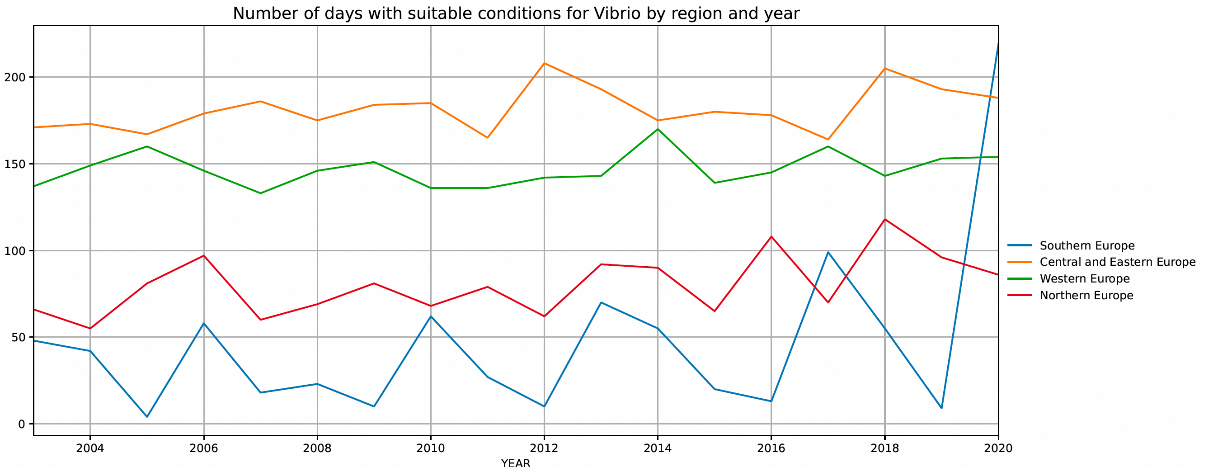

Figure 1.22 Time series of the estimated annual percentage of coastline showing suitable conditions for non-*cholerae Vibrio* (2003-2020) by European region.

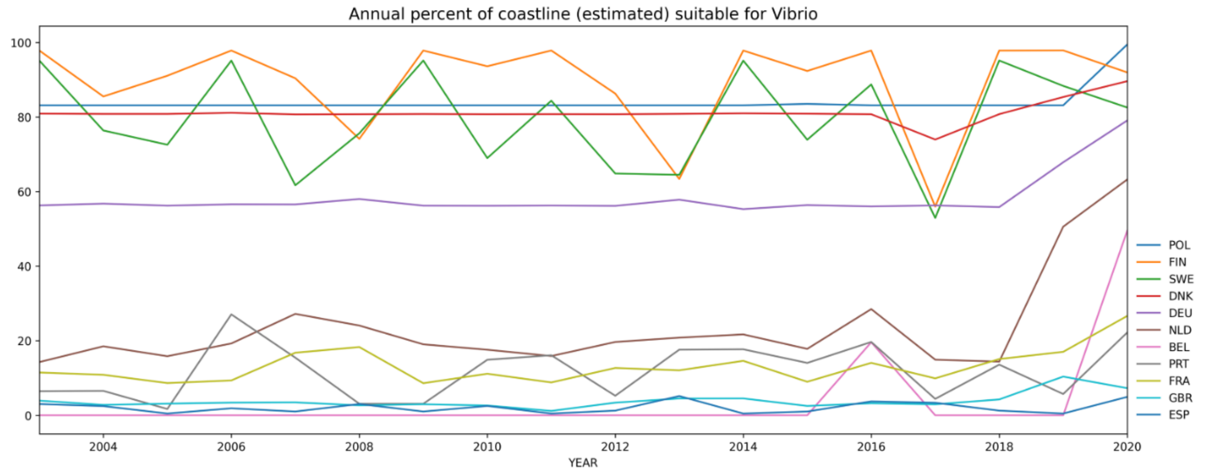

Figure 1.23 Time series of the estimated annual percentage of coastline showing suitable conditions for non-*cholerae Vibrio* (2003-2020) by country. The group showing the largest values correspond to high-latitude countries bordering the Baltic.

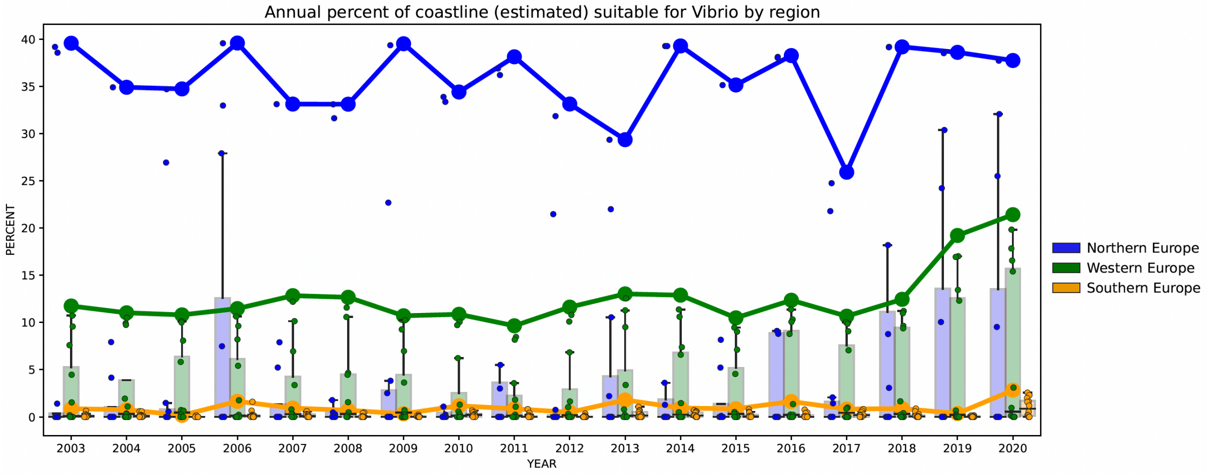

Figure 1.24 Percentage of coastline showing suitable conditions for *Vibrio* between 2003-2020.

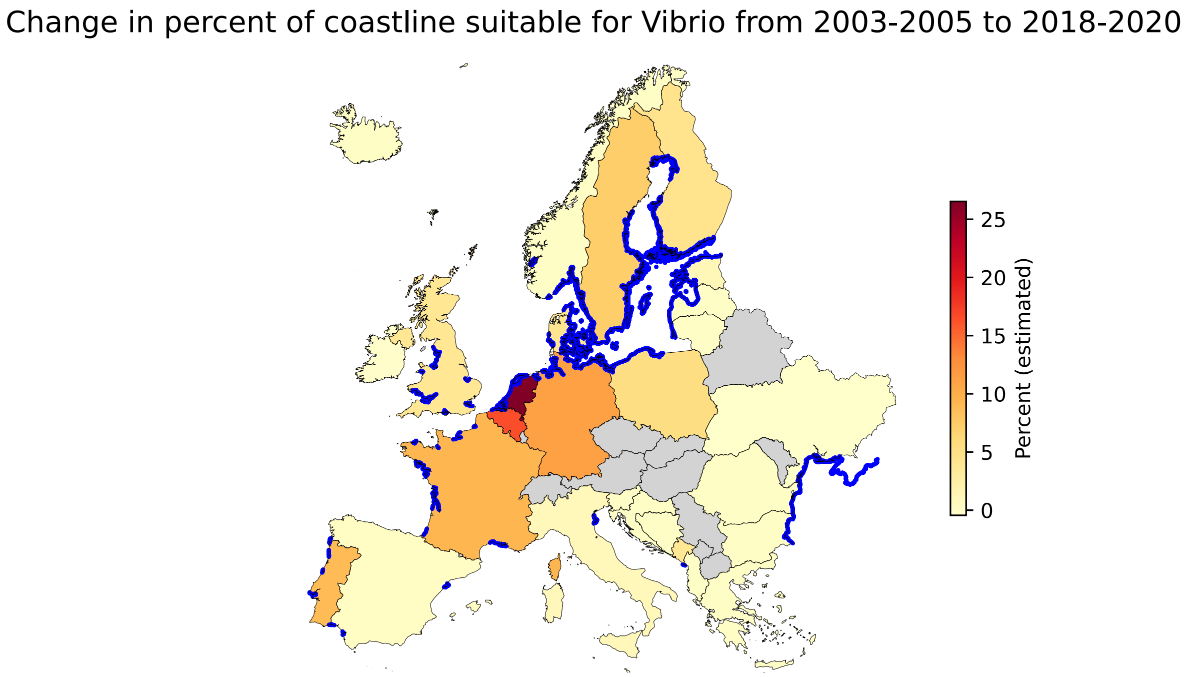

Figure 1.25 Increase in the percentage of coastline showing suitable conditions for *Vibrio* from the baseline 2003-2005 to 2018-2020 per country. The largest values correspond to the western Europe region, and more specifically to Netherlands and Belgium. The blue colour highlights the spatial distribution of *Vibrio* suitable waters along the coast for year 2020. The Baltic and Black sea regions represent hotspots, with year-round surface salinity values favourable for *Vibrio* growth.

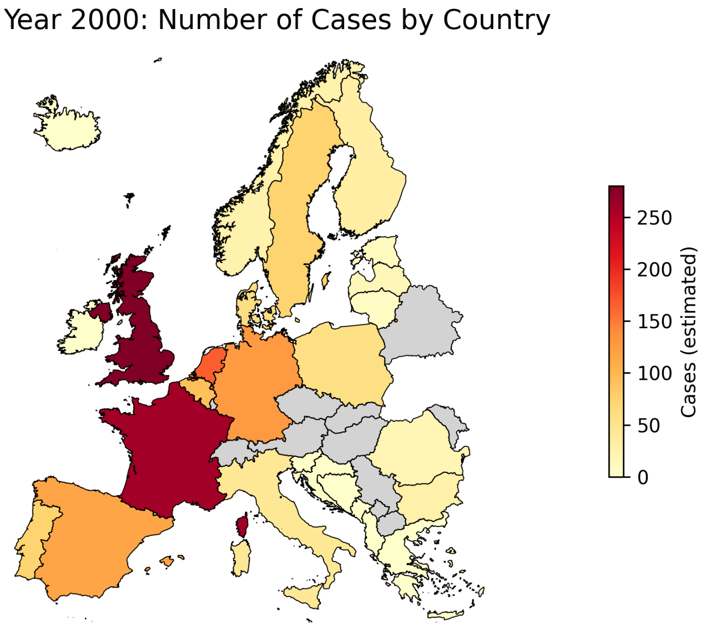

Figure 1.26 Estimated number of cases of Vibrio infections per country for year 2000. Countries in grey are landlocked were excluded from analysis. Other countries not shown in the map are not listed in the population datasets used in this study.

Indicator 1.3.2: Climate suitability West Nile Virus

Methods

The European NUTS 3 regions (2021 definition) spatiotemporal data consisting of climate and bioclimatic variables were used as predictors. All climatic variables, i.e., temperature and precipitation were averaged in four quarters. To provide covariates for the complex interactions between WNV outbreaks and climate, a set of 19 bioclimatic variables (bio01-bio19),[52] that delineate the annual tendencies were incorporated. The influence of the variables was presented by dividing regions into two groups based on transmission activity in a specific year. The transmission activity was based on the WNV human infections data. This data was obtained from the European Centre for Disease Prevention and Control (ECDC),[53] from 2010 to 2019. The regions with WNV human infections were attributed as positive class regions (1). The regions without any information on WNV human infections were classified as zero in the response variable.

A supervised machine learning classifier, extreme gradient boosting (XGBoost),[54,55] was applied to the final data set. For this, the data was split into two subsets: data from the years 2010-2018 as the training set, and 2019 as the testing set. For the model training and tuning, a 5-fold cross-validation approach was used. Further to assess the WNV risk retrospectively the model was re-trained and fine-tuned on (2010-2019) data set and tested on the 1950-2009 data set. The model output was the WNV transmission or outbreak risk probability at the NUTS 3 level. The model achieved an AUC (area under the receiver operator characteristic curve) score of 95.6% for the internal validation showing a high discriminatory power to classify a WNV-positive region. As there were no positive class instances available for the external validation data set, all the analyses were done without setting a classification-threshold in a departure from the usual practice to assess the performance of a machine learning algorithm.

Next, to interpret and explain the results of the black-box XGBoost algorithm, more advanced algorithms such as those of explainable AI (XAI) are imperative. To this end, the SHAP (Shapley Additive Explanations), a game-theoretic XAI framework,[56] developed recently, was used to disentangle the contribution of each feature to the model predictions.[57,58] SHAP ranks feature importance by comparing what a model predicts with and without the feature for all possible combinations of features at every single observation. The features are then ranked according to their contribution for each observation and averaged across observations.[59]

Geographic coverage of Europe

For this indicator, we included the European Environment Agency (EEA) member and cooperating countries plus the United Kingdom, excluding Kosovo and Liechtenstein as data was not available for these countries.

Data

For quarterly climate variables (temperatures and precipitation), ERA5-Land reanalysis data at 9km^2 resolution was used.[60] Also, the 19 bioclimatic variables were extracted as predictors from the same data set.[52] The records WNV human infections data for the response variable were obtained from ECDC.[53] This data was available for the years 2010-2019 only. This data contained a total of 597 NUTS 3 level observations of recorded human infections of which 593 were included in the main analysis as the remaining were recorded as ‘unknown’ for the NUTS 3 identification. The training data set was imbalanced in favour of negative classes (i.e., 96% regions with

no WNV infection cases) compared to the regions with WNV transmission (4% regions with WNV human infection cases).

Caveats

The WNV cases data related to human infections only were considered in the study. However, data on equines infections and host birds infections on the NUTS 3 level could also have been incorporated upon availability. The inclusion of the infections data for the equines and the host birds would further strengthen the model predictions though XGBoost performed reasonably well to handle the severe class-imbalance due to its flexibility of numerous hyperparameters tunning options.

Future form of the indicator

In future versions, the aim will be to improve by overcoming/ minimizing the caveats listed above, that is, by incorporating equines and equids infections data. This modification in the future indicators will likely increase the model's predictive power and robustness.

Additional analysis

Figure 1.27 shows the expected probability of WNV risk predicted by the model for each decade during the study period. Evidently, the trends in the increase of WNV outbreaks are evident in the regions where the virus is established, and the disease is endemic during the last decade of the 21st century.

The SHAP post-hoc analysis for the training period (2010-2019) was done to infer the influence and the contributions of the individual climatic and bioclimatic features on the model predictions. It was found that the temperature of the 2nd quarter *(Mean_temp_Q02)* of the year was the most influential climatic variable to the model predictions (figure 1.28). Figure 1.29 shows the aggregated decade-wise time trends of this variable (*Mean_temp_Q02)* for the whole study period. A statistically significant ($p < 0.05$) correlation ($R=0.64$) between the most influential variable (*Mean_temp_Q02)* and the model predicted WNV-outbreak risk probability was found using the Pearson correlation test (figure 1.30).

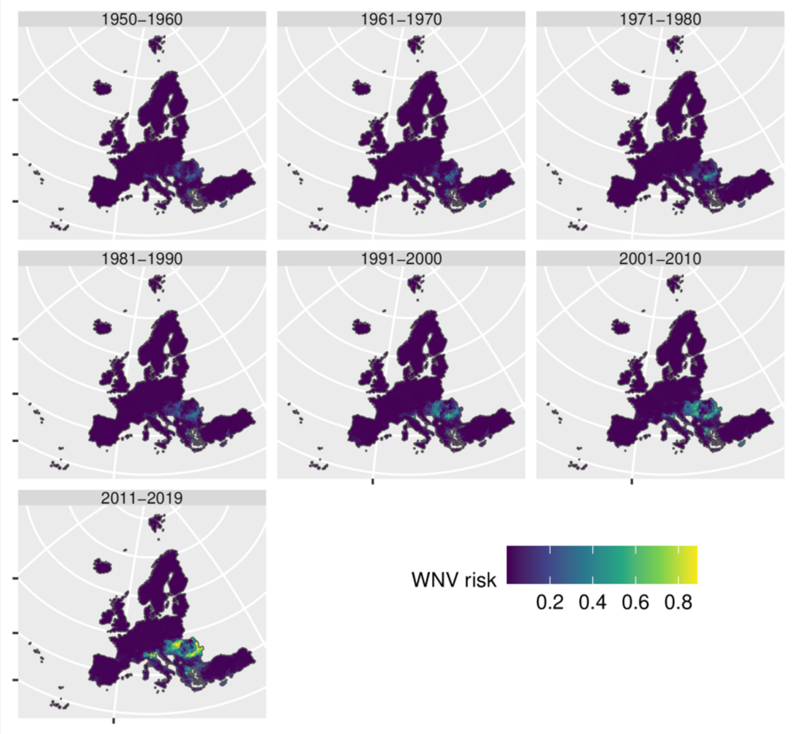

Figure 1.27 Model predicted WNV-outbreak risk for Europe from 1950 to 2019.

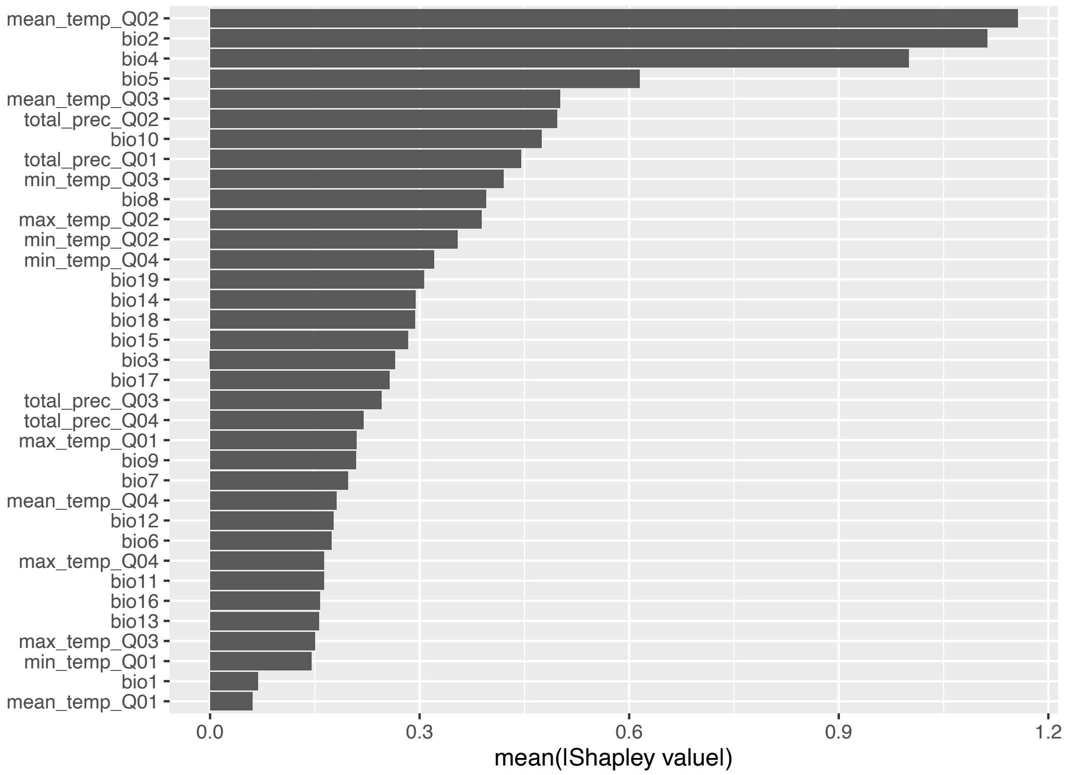

Figure 1.28 Ranking of the variables w.r.t their contribution to the model predictions estimated using SHAP.

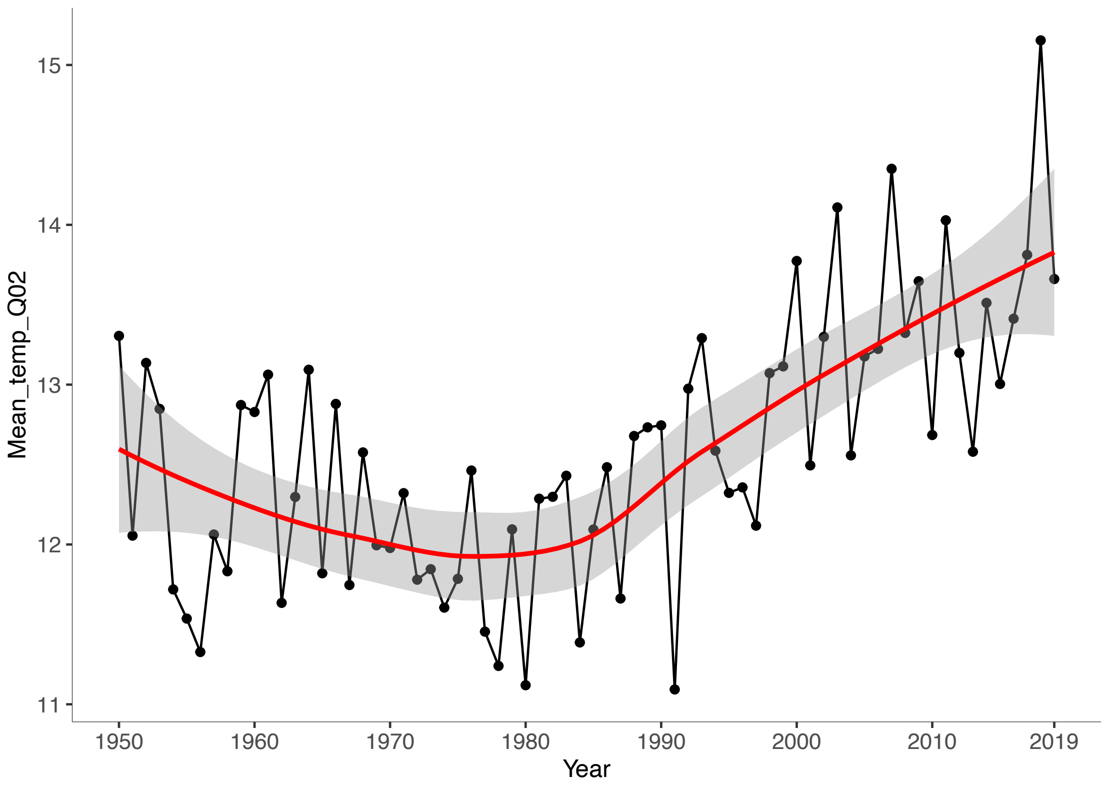

Figure 1.29 Decadal Change in the mean temperature (ºC) of the 2nd quarter of the year from 1950 to 2019

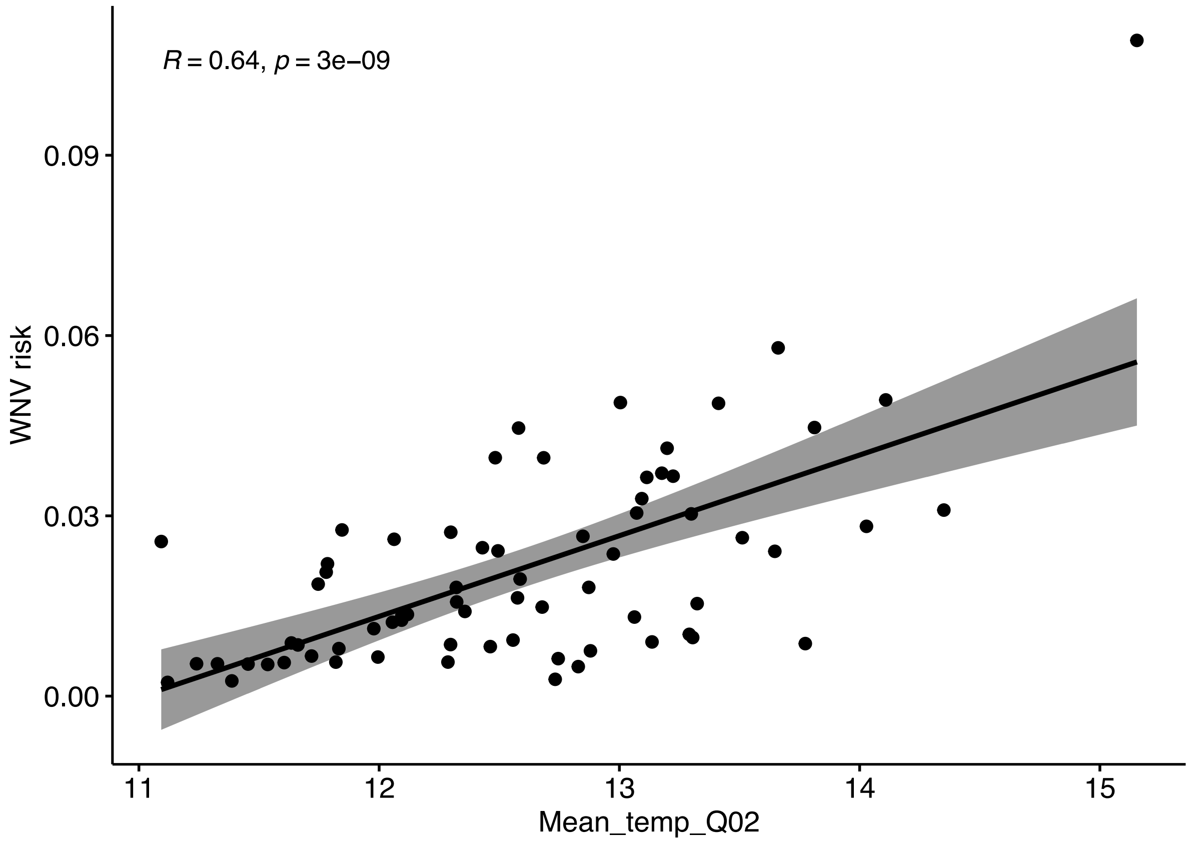

Figure 1.30 Scatter plot showing the correlation between the temperature of the 2nd quarter and the model predicted WNV risk, the correlation coefficient (R), and the p-value.

1.3.3: Climate suitability for dengue (chikungunya and Zika) *(first part indicator)*

Background

Cases of dengue have doubled every decade since 1990, with 58·4 million (23·6 million–121·9 million) apparent cases in 2013, accounting for over 10,000 deaths and 1·14 million (0·73 million–1·98 million) disability-adjusted life-years.[61] Beside global mobility, climate change has been suggested as one potential contributor to this increase in burden.[62] *Aedes aegypti* and *A. albopictus*, the principal vectors of dengue, also carry other important emerging or re-emerging arboviruses, including yellow fever, chikungunya, Mayaro, and Zika viruses, and are likely to be similarly responsive to climate change.

Methods

R_0, ie the basic reproduction number, which is the expected number of secondary infections resulting from one single primary infected person case in a totally susceptible population, was computed using the formula below[63]:

$$R_0 = Vb_h/r_h$$

The vectoral capacity (V), which express the average daily reproductive rate of subsequent cases in a susceptible population resulting from one infected case, was computed using the formula below[63]:

$$V = ma^2 b_m p^n / -lnp$$

Here, a is the average vector biting rate, b_m is probability of vector infection and transmission of virus to its saliva, n is the extrinsic incubation period while p is the daily survival probability. All these parameters are temperature dependent and are further described in Rocklöv *et al.* 2016, 2019 A, 2019 B.[63,64,65]

The ratio between number of mosquitoes to the number of humans, is central to V and the R_0 value (m), but often it is left out or estimated simplicity. Here we use a model to estimate mosquito populations of *Aedes aegypti* and *Aedes albopictus* separately. The original mosquito-population models provide results in terms of the number of individuals of Ae. aegypti per breeding site (X), or the number of Ae. albopictus per hectare (Y).[66,67] In order to appropriately estimate m, i.e., mosquito population density per human population density (p), we multiplied X by f(p,a,c) = a ∗ g(p,c) where a equals to the number of breeding-sites per human, and Y by f(p,a/b,c) = a ∗ g(p,c)/b where b equals the average number of breeding sites per hectare. The function g(p,c) = p2/(c2 +p2) is an increasing sigmoidal function that equals the viability of domesticated mosquito-populations in relation to human population density. Accordingly, f(p,a,c) is the multiplicative factor m in V, which allowed us to straightforwardly estimate correct values for a, a/b and c by fitting R_0 to R_0 -data that was available for a subset of the spatiotemporal points.[68]

Numerically V and abundance estimates was computed at a 9km^2 spatial resolution based on ERA5-Land data.[13]

We ran V and vector abundance for both *A. aegypti* and *A. albopictus* vectors. Hybrid gridded population from Chambers[69] were used in the computation of R_0. For dengue and Chikungunya, *A. albopictus* vector abundance estimates were used in the computation of m while for Zika, *A. aegypti* abundance estimates were used. Further, the annual length of transmission season was computed by summing the number of months in a year when R_0 was greater than 1.

The gridded R_0 and LST for dengue (*A. albopictus*), chikungunya (*A. albopictus*) and Zika (*A. aegypti*) were extracted and averaged by NUTS regions, European regions (e.g. ….) and by country.

Geographic coverage of Europe

For this indicator, European Environment Agency (EEA) member and cooperating countries plus the United Kingdom were included.

Data

1. ERA5-Land monthly averaged data from 1950 to 202[13]
2. Copernicus Climate Change Service (C3S) precipitation and temperature data
3. HYBRID gridded population data[69]

Caveats

Key caveats and limitations of the V model and its parameterisation are fully described in Liu-Helmersson *et al.* 2014[70] and 2016,[71] and Rocklöv *et al.* 2019.[63] The predicted R_0 should not be confused with actual dengue cases, although it is an indicator of the potential for outbreaks.[64,65]

Additional analysis

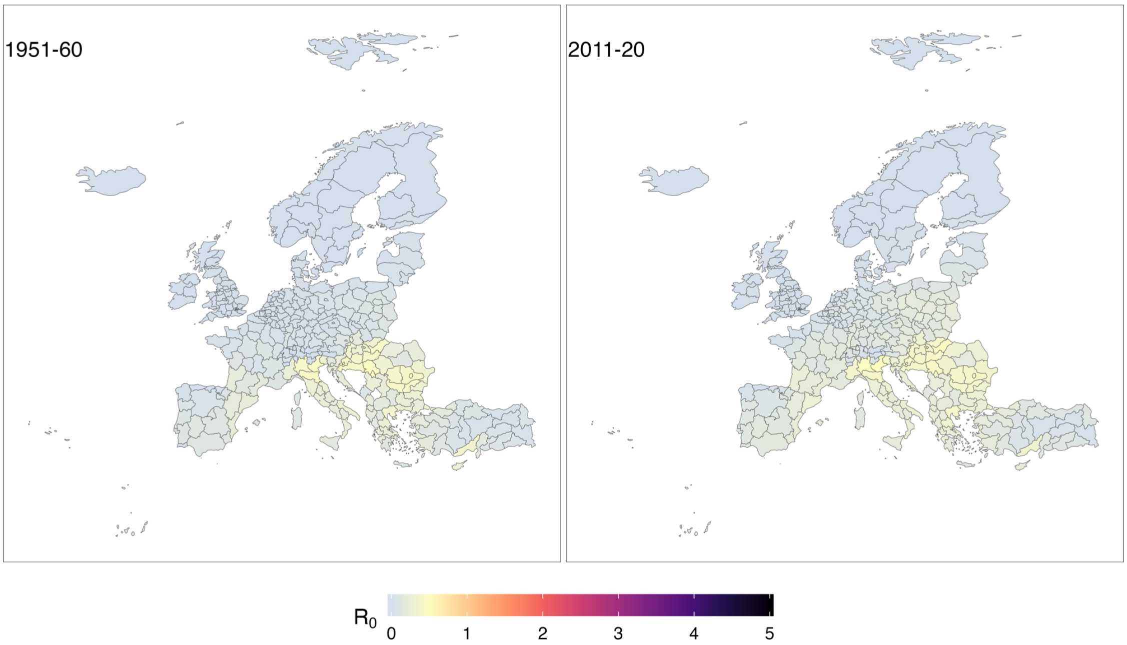

Figure 1.31 R_0 by decade 1951-1960 vs 2011-2020 for dengue by NUTS 2 regions.

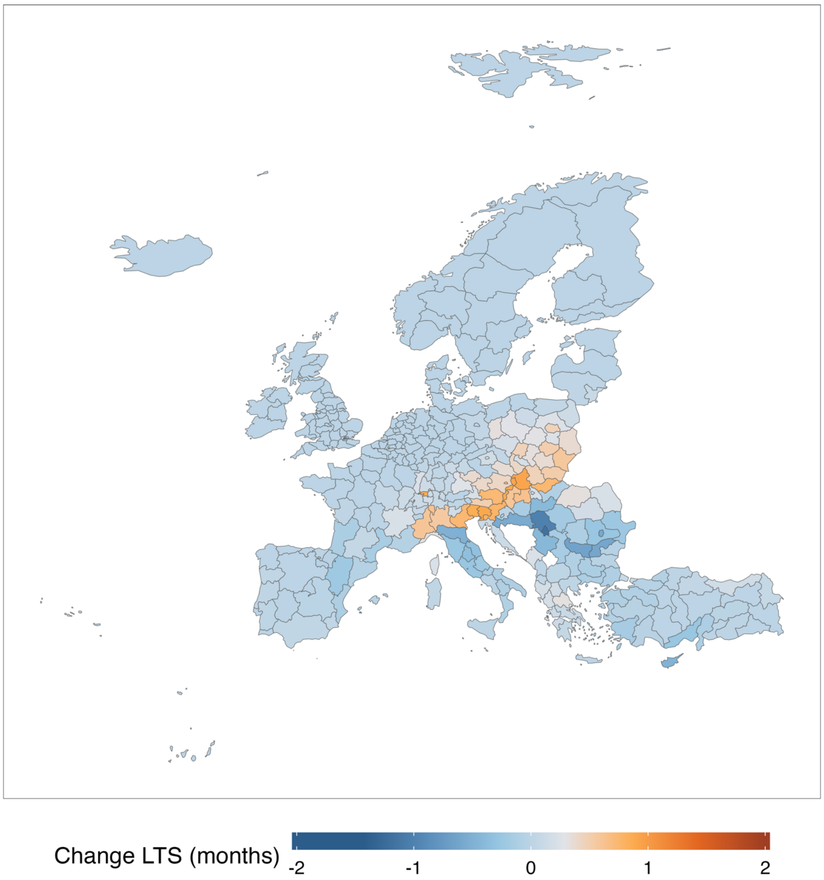

Figure 1.32 Change in length of transmission season (LST) in months per year by decade 1951-1960 and 2011-2020 for dengue by NUTS 2 regions

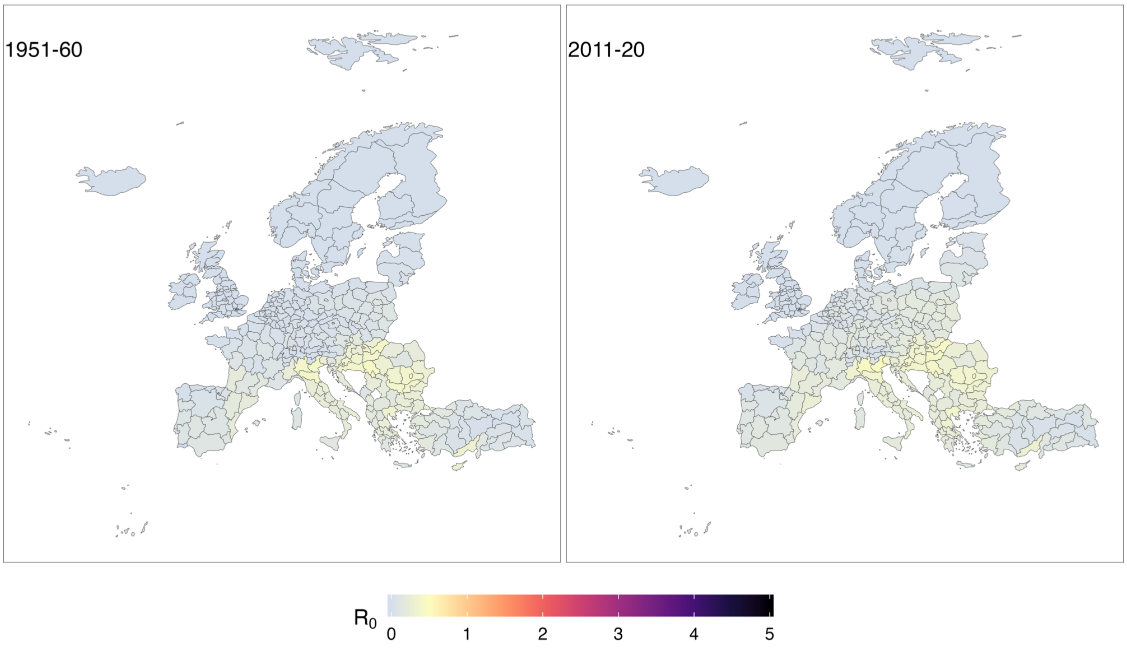

Figure 1.33 R_0 by decade 1951-1960 vs 2011-2020 for chikungunya by NUTS 2 regions.

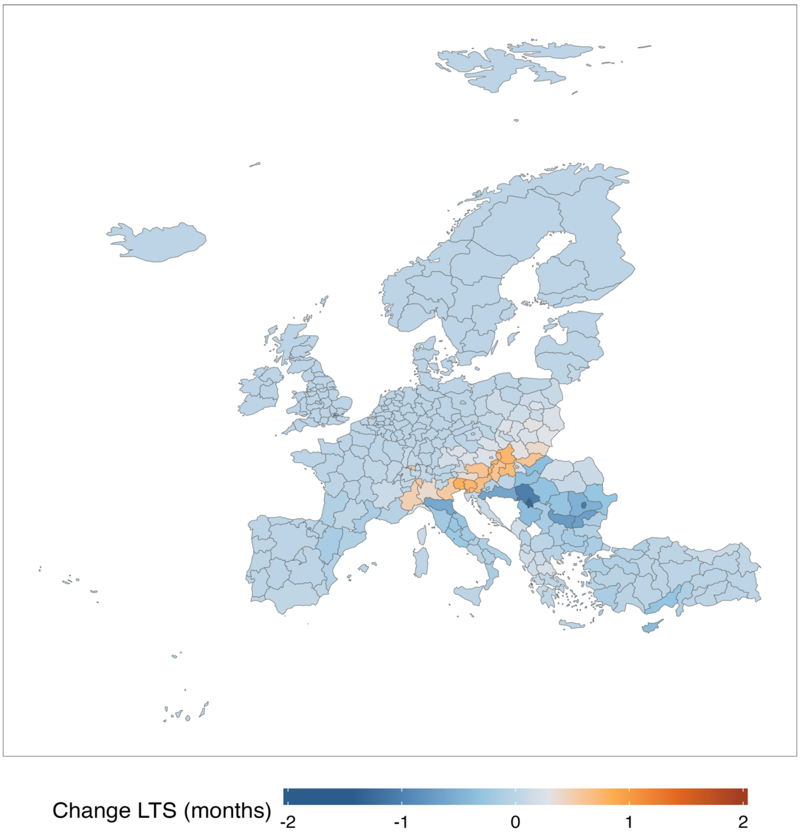

Figure 1.34 Change in length of transmission season (LST) in months per year by decade 1951-1960 and 2011-2020 for Chikungunya by NUTS2 regions.

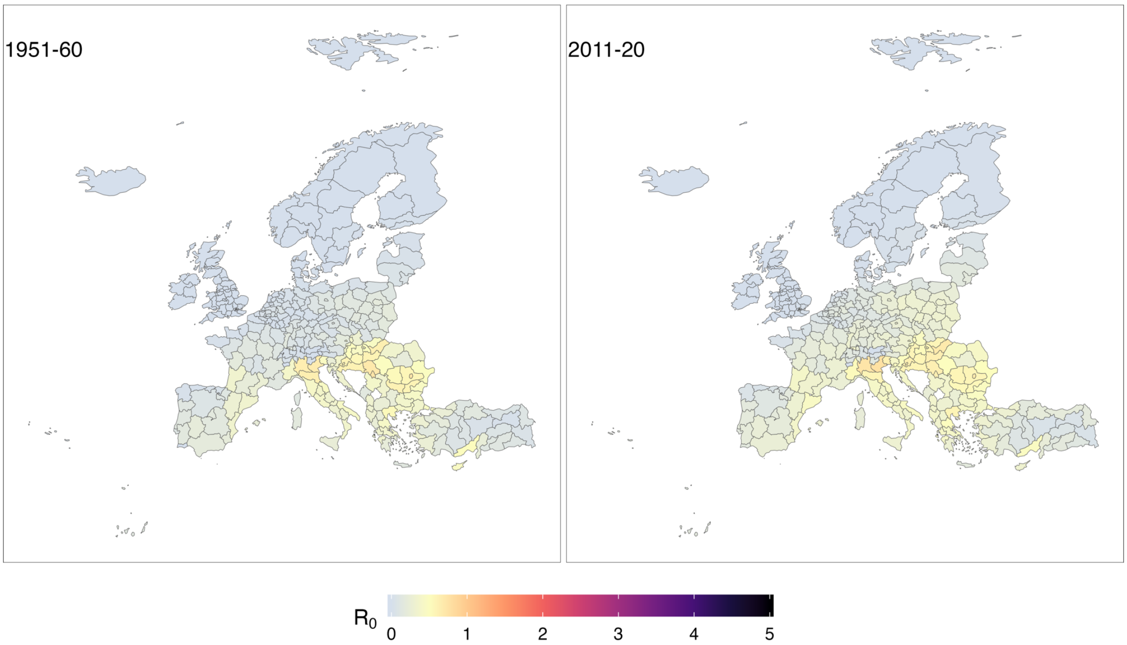

Figure 1.35 R_0 by decade 1951-1960 vs 2011-2020 for Zika by NUTS2 regions.

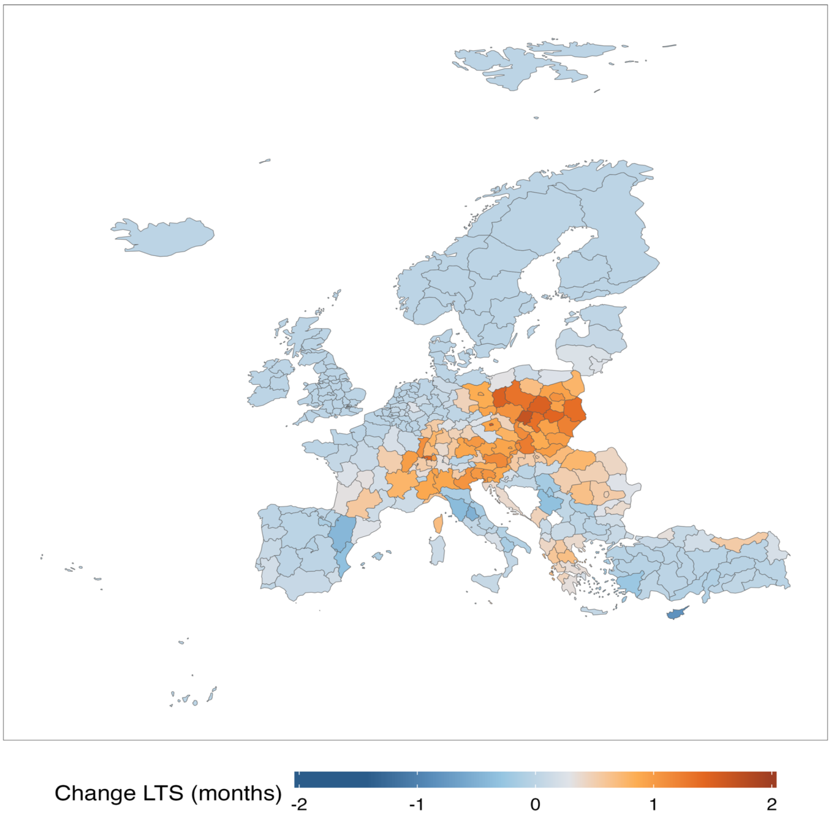

Figure 1.36 Change in length of transmission season (LST) in months per year by decade 1951-1960 and 2011-2020 for Zika by NUTS2 regions.

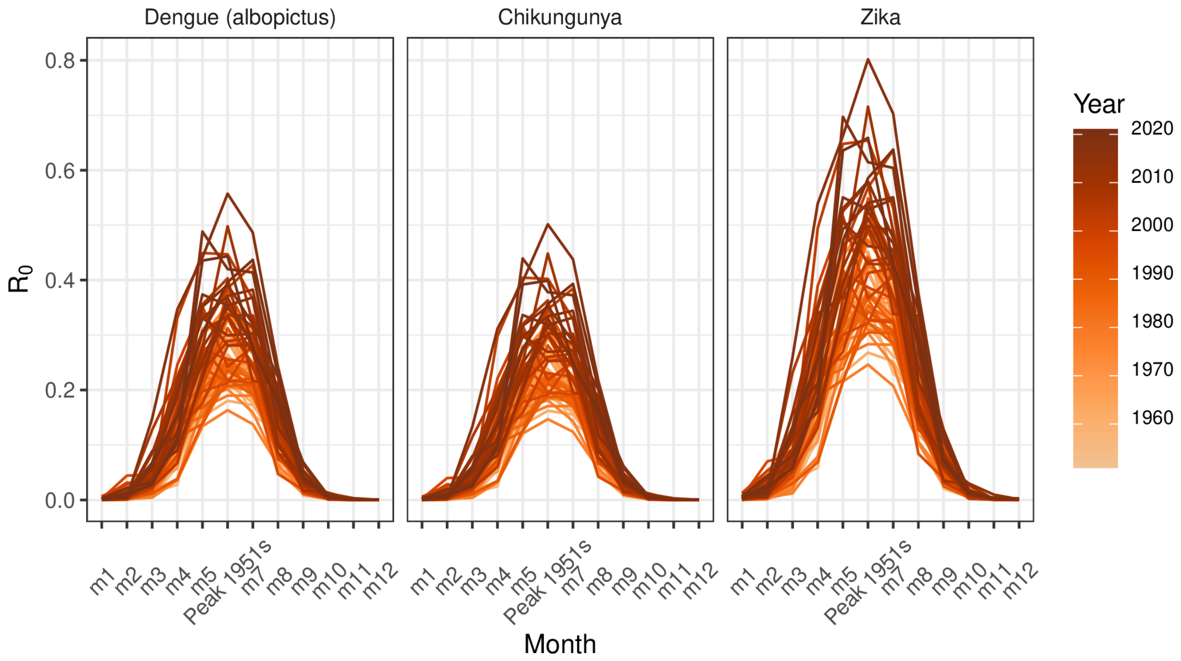

Figure 1.37 Seasonal pattern of R_0 for the period 1951-2021 for dengue, Chikungunya and Zika

Indicator 1.3.4: Dengue (Chikungunya and Zika) *(second part indicator)*

Human mobility is a factor in the importation risk for Europe, and arises here from the interplay between travel propensity, prevalence in endemic regions across the world, and environmental suitability for vectors and pathogens in Europe.

Methods

The average travel rate from source location i to destination location j is given by the radiation model

$$\langle U_{ij} \rangle = U_i \frac{n_i\, n_j}{(n_i + s_{ij})(n_i + n_j + s_{ij})}, \qquad (1)$$

where n_i is the population size at the source location, n_j that at the destination location, and s_{ij} the population size that is encompassed by a circle around location i with a radius given by the distance between locations i and j.[72] With $U_i = n_i t_i u_i$, where t_i is the proportion of the population at location i that is travelling, and u_i the proportion of the population at location i that is infected, $\langle U_{ij} \rangle$ is the average travel rate of infected individuals from i to j, i.e., the number of infected individuals that travels between location i and location j per unit time. Here, we have used one year as unit time.

For every year in the range 1990 to 2019, the variables n_i, n_j, s_{ij}, t_j, and u_i was given by data or derived from data at a yearly temporal resolution (see section Data). Country-level population size was given by aggregating gridded data[69] to country level or NUTS 3 level. Country-level population sizes were used for any location outside of Europe. Within Europe, NUTS 3 level population sizes were used. Accordingly, for any travel into Europe, n_i was country-level population size, and n_j NUTS 3 level population size. As s_{ij} is the population size that is encompassed by a circle around location i with a radius given by the distance between locations i and j, it includes both country-level and NUTS-3-level population sizes. The average proportion of individuals u_i, at any point in time for a given year in source location i, that is infected within a phase such that viremia may set in during international travels, was derived from dengue incidence data (individuals per 100,000 per year) reported in the Global Burden of Disease.[7] Specifically, it was assumed that $u_i = \frac{v_i}{n_i} = \frac{d_i}{3650000}$, where the average number of infected individuals at any day within a year $v_i = d_i \frac{10}{365} \frac{n_i}{100000}$, which assumes that incidence was uniformly distributed over any year and that a travels within a 10-day window is plausible for viremia to set in during international visits.

The proportion of international travellers t_i for any year, was derived by linear interpolation of international travel data,[73,74] together with the condition that $t_i > 0$.

To obtain a relevant measure of the mobility indicator that takes the environmental suitability for vectors and pathogens into account, the average travel rate of infected individuals from i to j was overlaid with the length of transmission season (LTS) at destination location j, denoted here by L_j. The LTS was derived in *Lancet* Countdown Europe indicator: Climate suitability for dengue, chikungunya, Zika, and is measured in the unit of months per year. This overlay means, specifically, that the mobility considers (1) only NUTS 3 regions where L_j was at least one month, and (2), that the monthly number of inbound infected individuals in any such NUTS 3 region j was multiplied with the corresponding $L_j > 1$. The resulting mobility measure:

$$M_{ij} = \langle U_{ij} \rangle L_j / 12 \qquad (2)$$

is thus an estimation of the yearly number of infected inbound individuals from source location i to any NUTS 3-region j where the environmental suitability is such that transmission is plausible. This assumes that travels are uniformly distributed over time. From equation (2), we can derive the corresponding yearly total number of relevant infected inbound individuals to any NUTS 3- egion j and write

$$I_j = \sum_{i \in \Omega} M_{ij}, \tag{3}$$

where Ω denotes the set of dengue endemic countries. Similarly, we can derive the yearly total number of relevant infected outbound individuals from location i and write

$$O_i = \sum_{j \in \Psi} M_{ij}, \tag{4}$$

where Ψ is the set of NUTS 3 regions with environmentally suitable conditions for vectors and pathogens.
Code development and numerical computation were done in Matlab 2021b.

Geographic coverage of Europe

For this indicator we included the European Environment Agency (EEA) member and cooperating countries plus the United Kingdom - excluding Kosovo and Liechtenstein as data was not available for these countries.

Data

See section previous section, i.e., Methods.

Caveats

The radiation model is one of the most suitable mobility models to use as it is tractable, and it is essentially parameter free (only a scaling parameter). It has been shown to provide general and robust results.[72,75] The rate estimations are still based only on population-sizes, and do not account for travel processes dependent on other aspects. Whereas some deviations from realizations naturally should be expected, the radiation model could still be expected to capture the important trends.
This analysis was data driven and based on data from across the world. One should note that any deficiencies in the applied data could affect the results computed and presented here. To our knowledge, however, the data applied here were of high quality.

Future form of the indicator

In this novel attempt to provide indications on the contribution of mobility to disease transmission in Europe, only unidirectional commuting has been considered. This means that only visits to Europe have been considered, and not commuting from Europe to endemic countries. For a more complete picture, bidirectional commuting should be considered, and is therefore an important objective for further development and analysis.
Future forms could also consider secondary or tertiary recursions, i.e., account for exportation within Europe from NUTS 3 regions where disease transmission arises due to primary importation pressures.

Additional analysis

For the years 1990 to 2019, figures 1.38 to 1.42 show degrees of importation to European NUTS 3 regions, and figures 1.43 to 1.57 show world maps where both degrees of exportation from endemic regions and degrees of importation to European NUTS-3 regions are indicated.

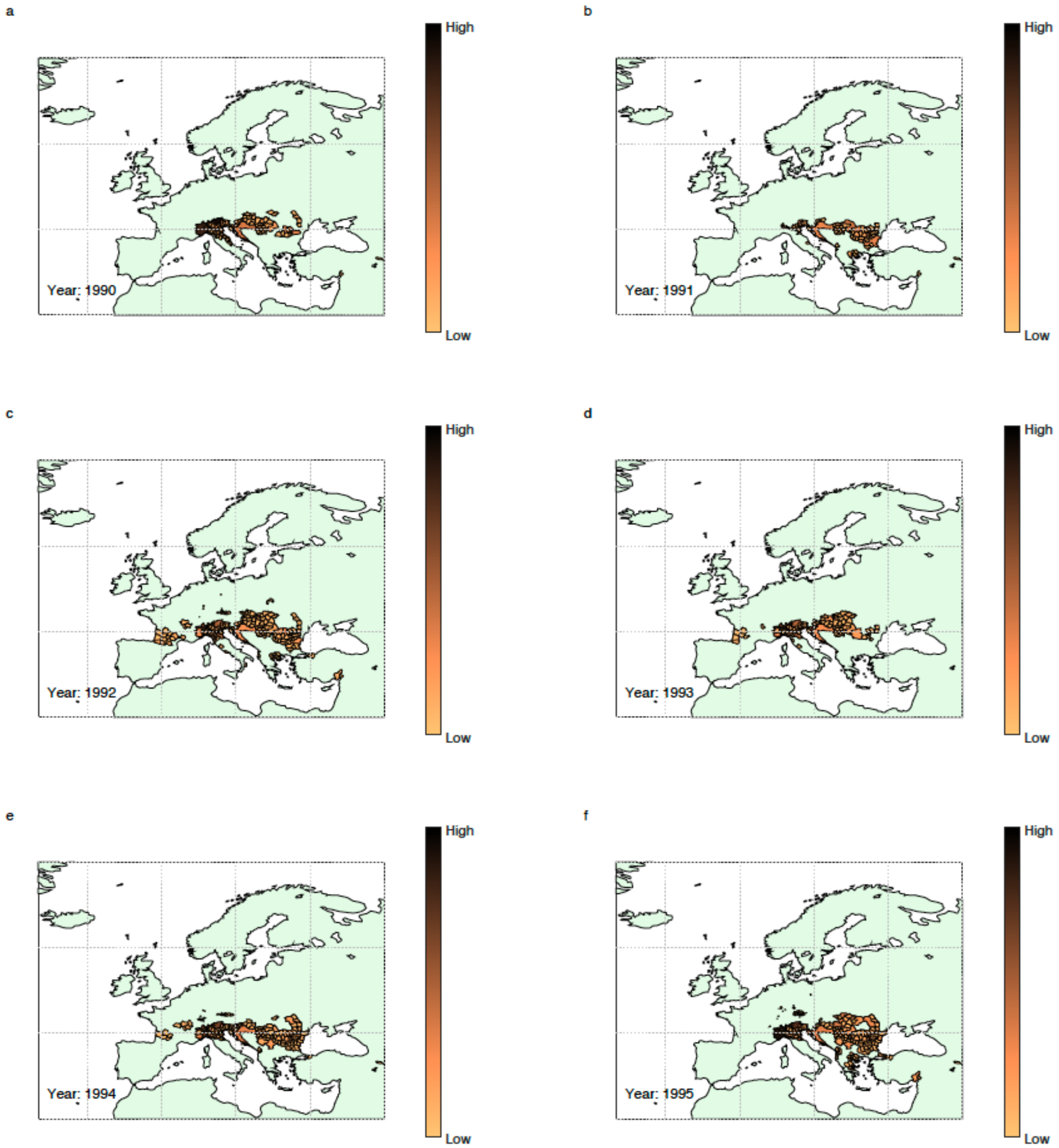

Figure 1.38 Dengue importation in Europe at NUTS-3 level 1990-1995. The European NUTS 3 regions where the length of transmission season was at least one month, for the respective years as given by the in-figure annotations, are indicated by shaded NUTS 3 shapes. The degree of shading, ranging from low to high as shown in the bar legend, indicates a value that is proportional to the number of infected inbound travellers from dengue-endemic countries across the globe during a time that is equal to the length of transmission season for the respective NUTS-3 regions.

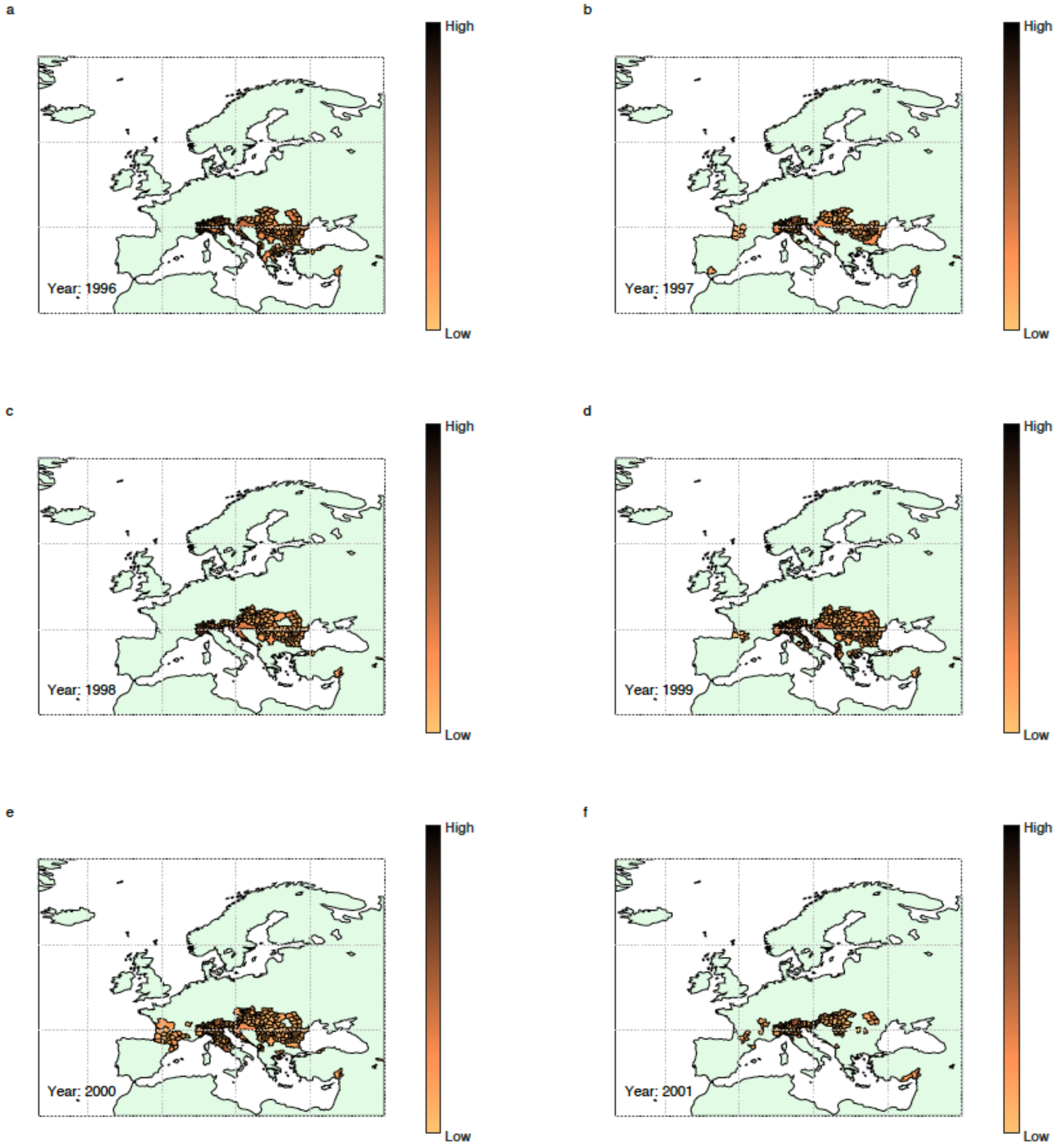

Figure 1.39 Dengue importation in Europe at NUTS-3 level 1996-2001. The European NUTS 3 regions where the length of transmission season was at least one month, for the respective years as given by the in-figure annotations, are indicated by shaded NUTS-3 shapes. The degree of shading, ranging from low to high as shown in the bar legend, indicates a value that is proportional to the number of infected inbound travellers from dengue-endemic countries across the globe during a time that is equal to the length of transmission season for the respective NUTS-3 regions.

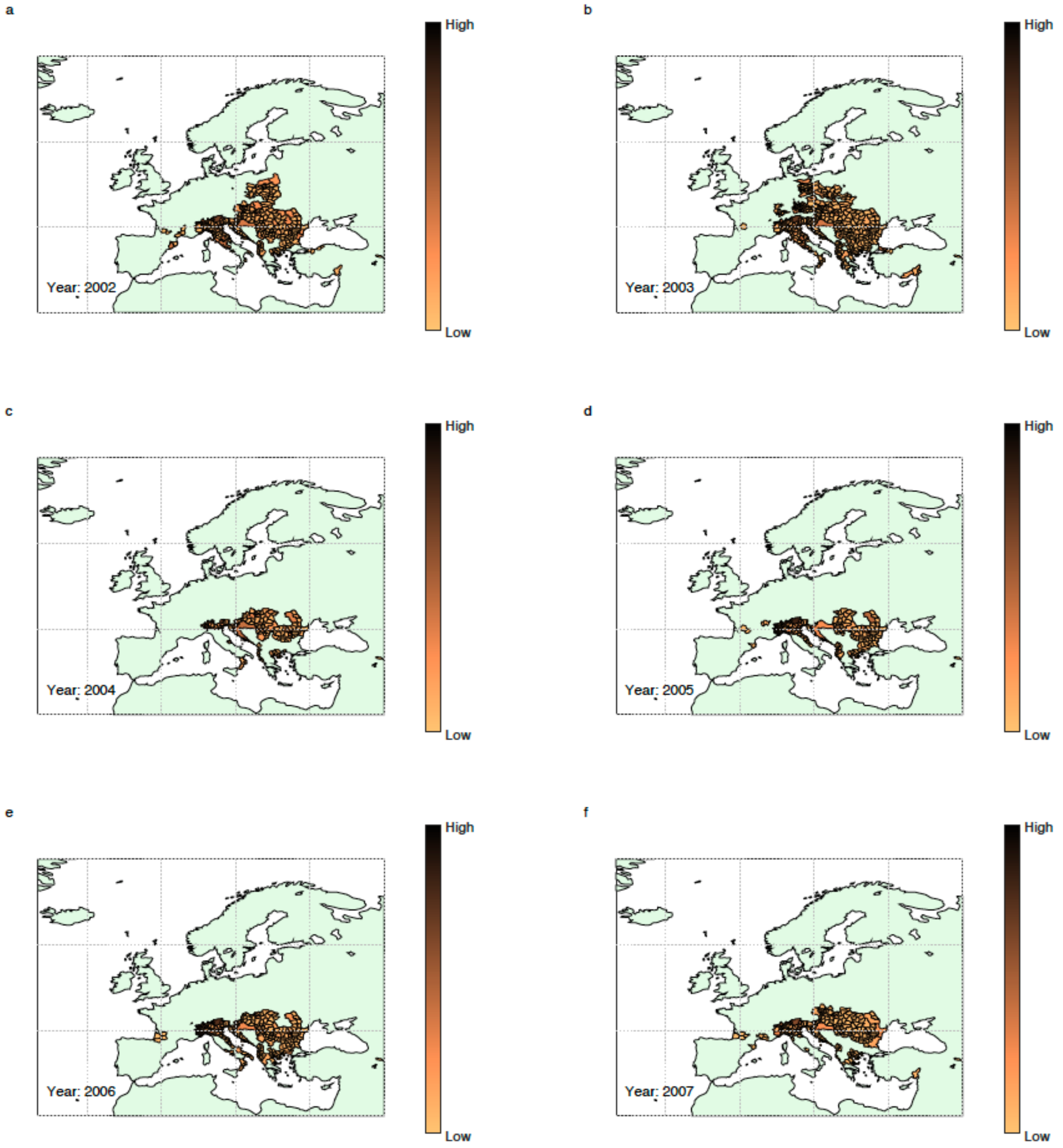

Figure 1.40 Dengue importation in Europe at NUTS-3 level 2002-2007. The European NUTS-3 regions where the length of transmission season was at least one month, for the respective years as given by the in-figure annotations, are indicated by shaded NUTS-3 shapes. The degree of shading, ranging from low to high as shown in the bar legend, indicates a value that is proportional to the number of infected inbound travellers from dengue-endemic countries across the globe during a time that is equal to the length of transmission season for the respective NUTS-3 regions.

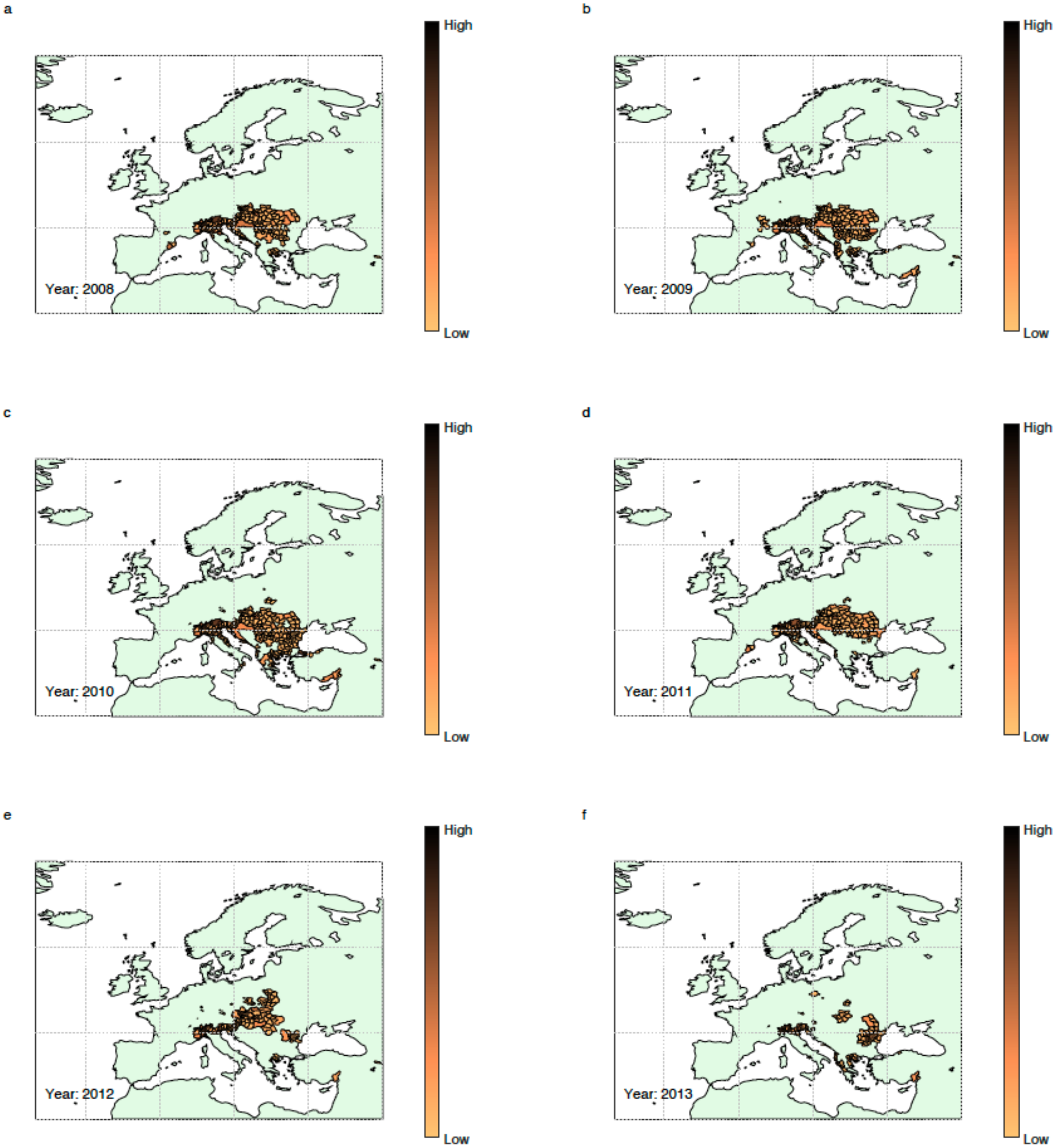

Figure 1.41 Dengue importation in Europe at NUTS 3 level 2008-2013. The European NUTS 3 regions where the length of transmission season was at least one month, for the respective years as given by the in-figure annotations, are indicated by shaded NUTS 3 shapes. The degree of shading, ranging from low to high as shown in the bar legend, indicates a value that is proportional to the number of infected inbound travellers from dengue-endemic countries across the globe during a time that is equal to the length of transmission season for the respective NUTS-3 regions.

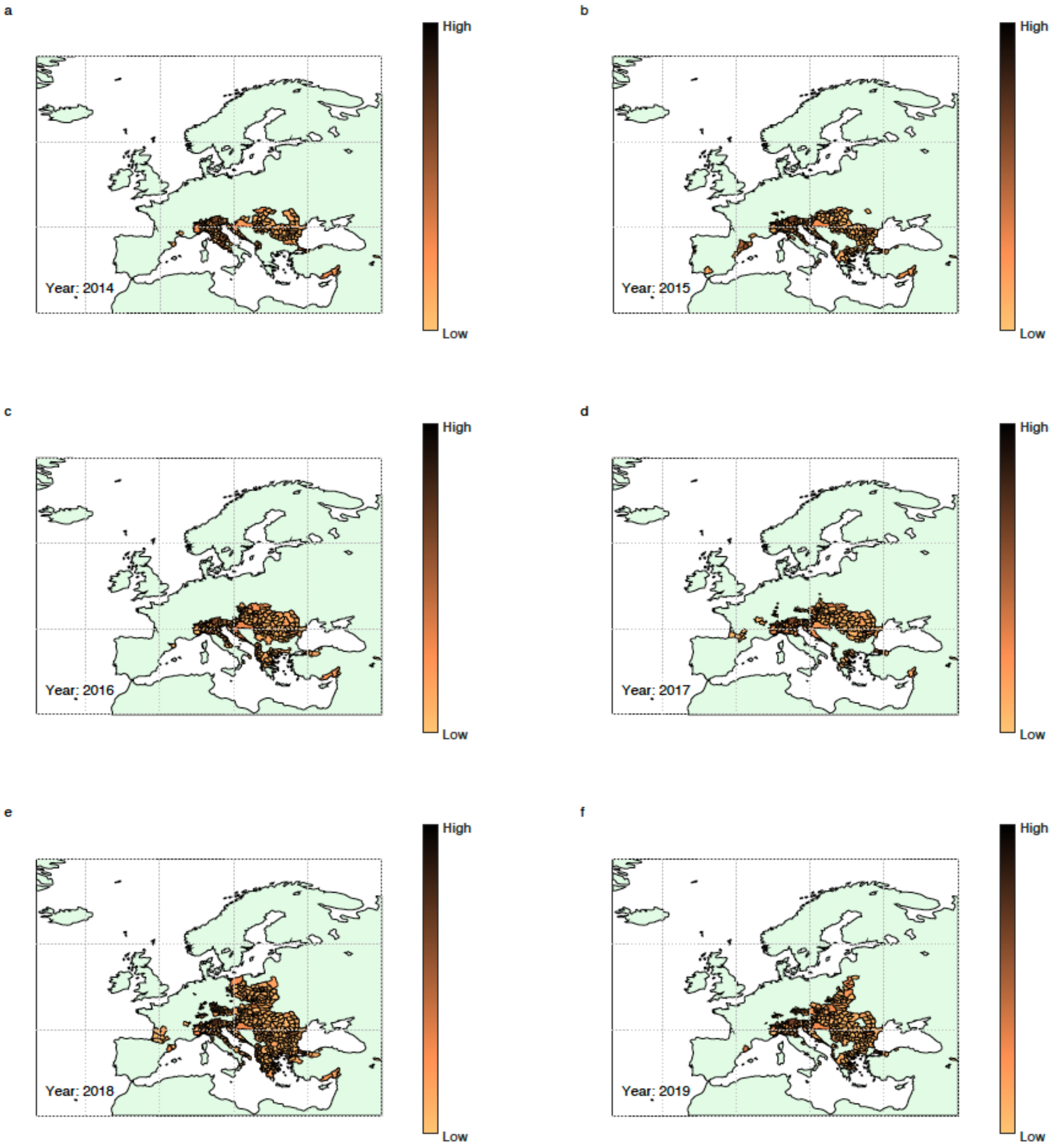

Figure 1.42 Dengue importation in Europe at NUTS 3 level 2014-2019. The European NUTS 3 regions where the length of transmission season was at least one month, for the respective years as given by the in-figure annotations, are indicated by shaded NUTS 3 shapes. The degree of shading, ranging from low to high as shown in the bar legend, indicates a value that is proportional to the number of infected inbound travellers from dengue-endemic countries across the globe during a time that is equal to the length of transmission season for the respective NUTS-3 regions.

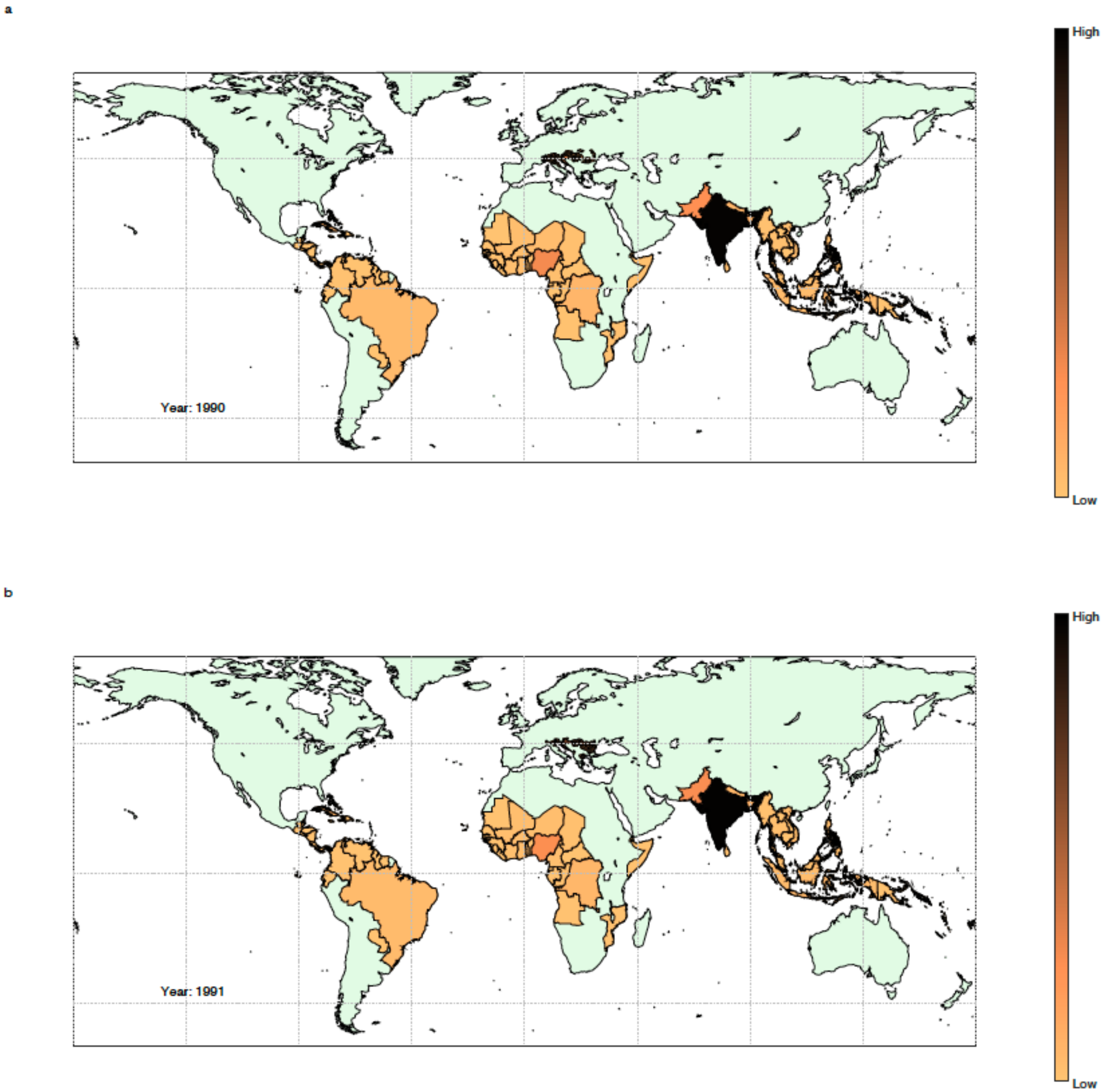

Figure 1.43 World map showing dengue-endemic export regions (countries) outside of Europe, and environmentally suitable import regions (NUTS 3) in Europe, for the respective years (1990, 1991) as indicated by in-figure annotations. The degree of shading, ranging from low to high as shown in the bar legend, indicates a value that is proportional to the number of infected inbound travellers to any given import region from export regions (for NUTS 3 regions), or a value that is proportional to the number of infected outbound travellers from export countries (for countries outside of Europe).

a

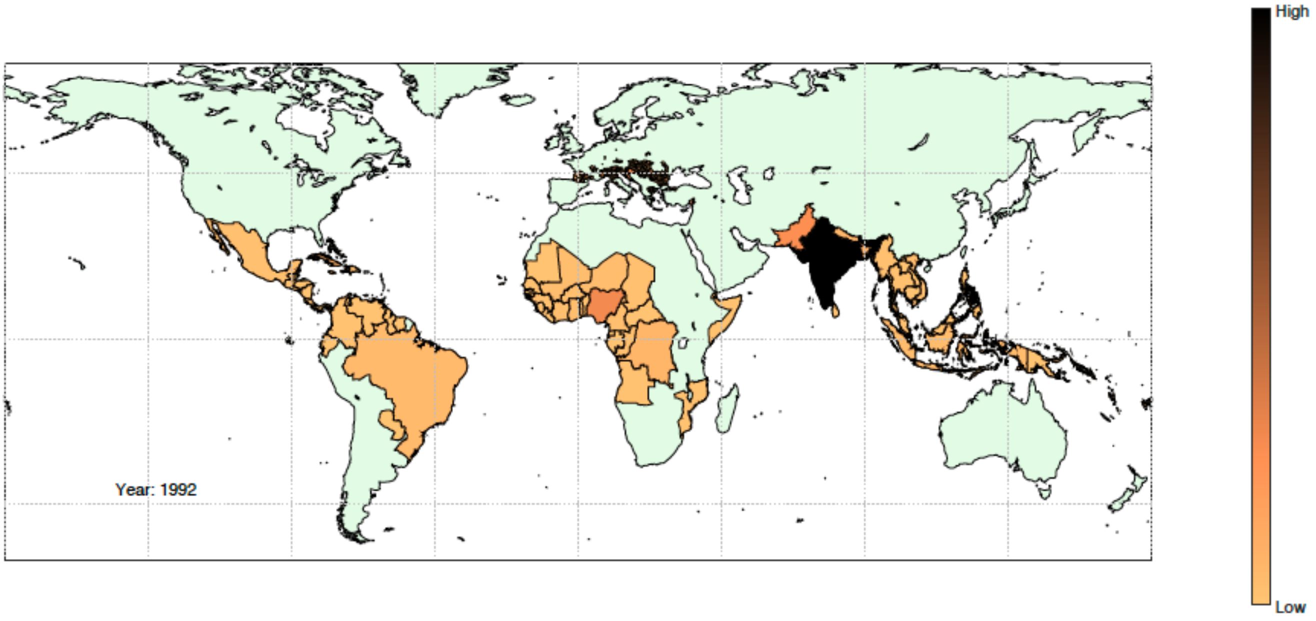

b

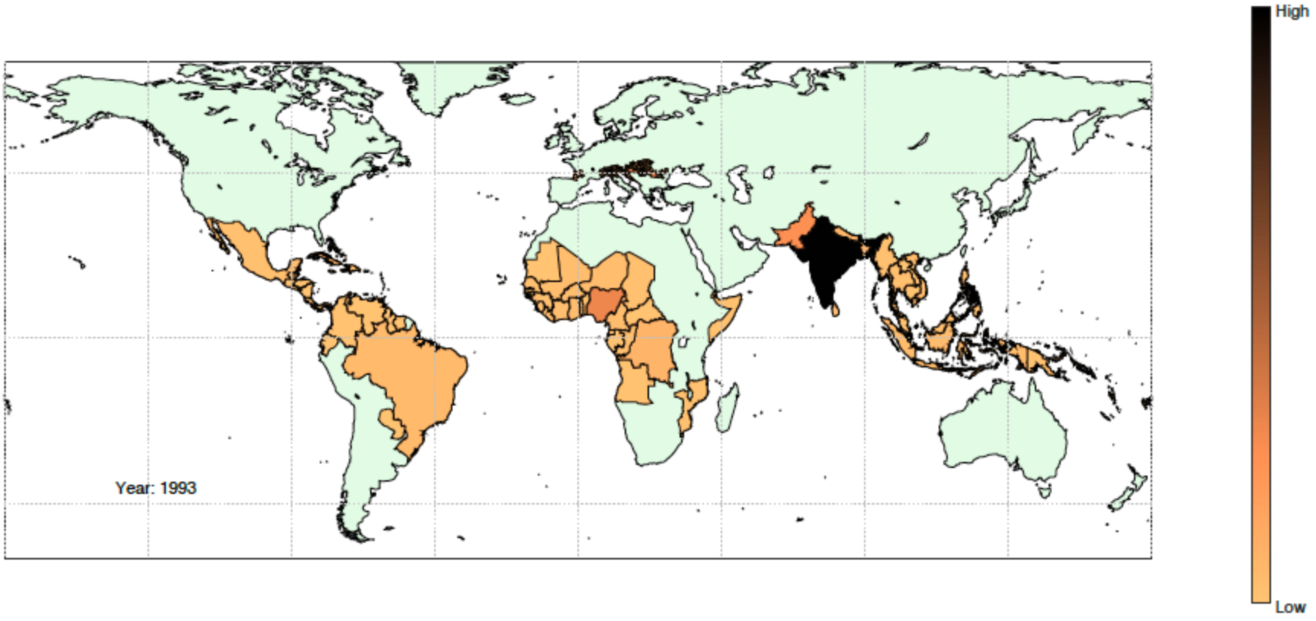

Figure 1.44 World map showing dengue-endemic export regions (countries) outside of Europe, and environmentally suitable import regions (NUTS-3) in Europe, for the respective years (1992, 1993) as indicated by in-figure annotations. The degree of shading, ranging from low to high as shown in the bar legend, indicates a value that is proportional to the number of infected inbound travellers to any given import region from export regions (for NUTS-3 regions), or a value that is proportional to the number of infected outbound travellers from export countries (for countries outside of Europe).

a

b

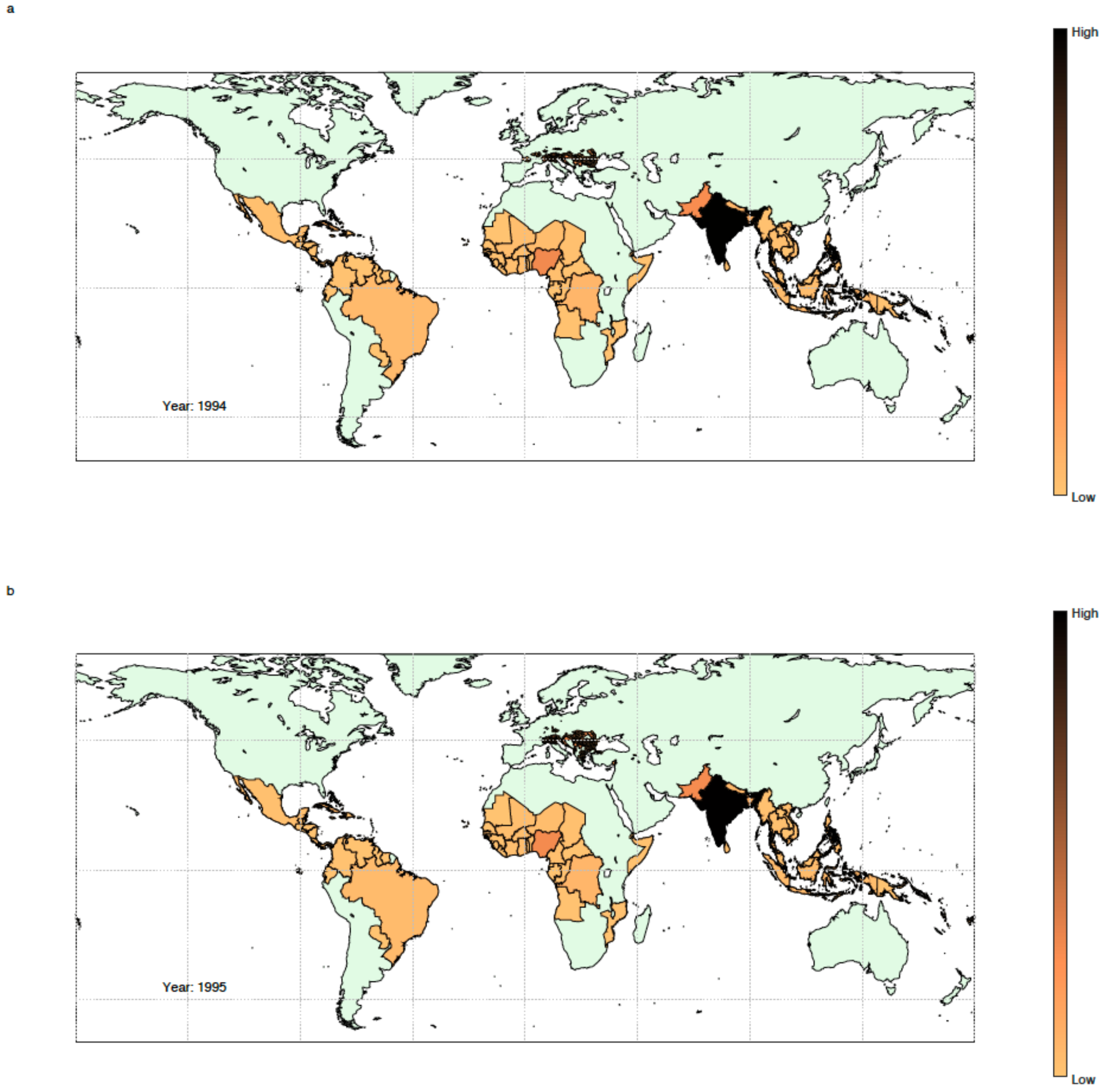

Figure 1.45 World map showing dengue-endemic export regions (countries) outside of Europe, and environmentally suitable import regions (NUTS-3) in Europe, for the respective years (1994, 1995) as indicated by in-figure annotations. The degree of shading, ranging from low to high as shown in the bar legend, indicates a value that is proportional to the number of infected inbound travellers to any given import region from export regions (for NUTS-3 regions), or a value that is proportional to the number of infected outbound travellers from export countries (for countries outside of Europe).

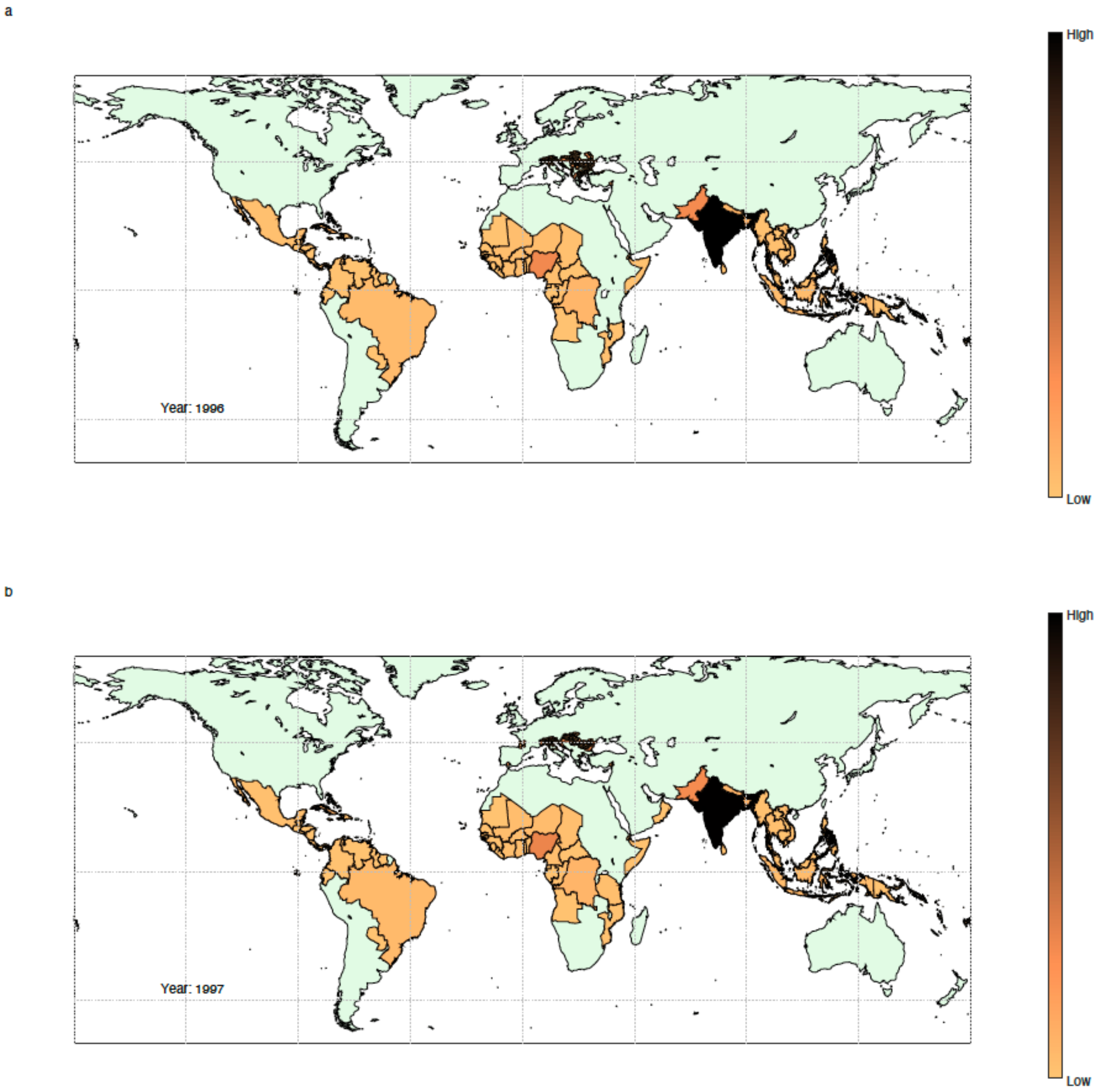

Figure 1.46 World map showing dengue-endemic export regions (countries) outside of Europe, and environmentally suitable import regions (NUTS-3) in Europe, for the respective years (1996, 1997) as indicated by in-figure annotations. The degree of shading, ranging from low to high as shown in the bar legend, indicates a value that is proportional to the number of infected inbound travellers to any given import region from export regions (for NUTS-3 regions), or a value that is proportional to the number of infected outbound travellers from export countries (for countries outside of Europe).

a

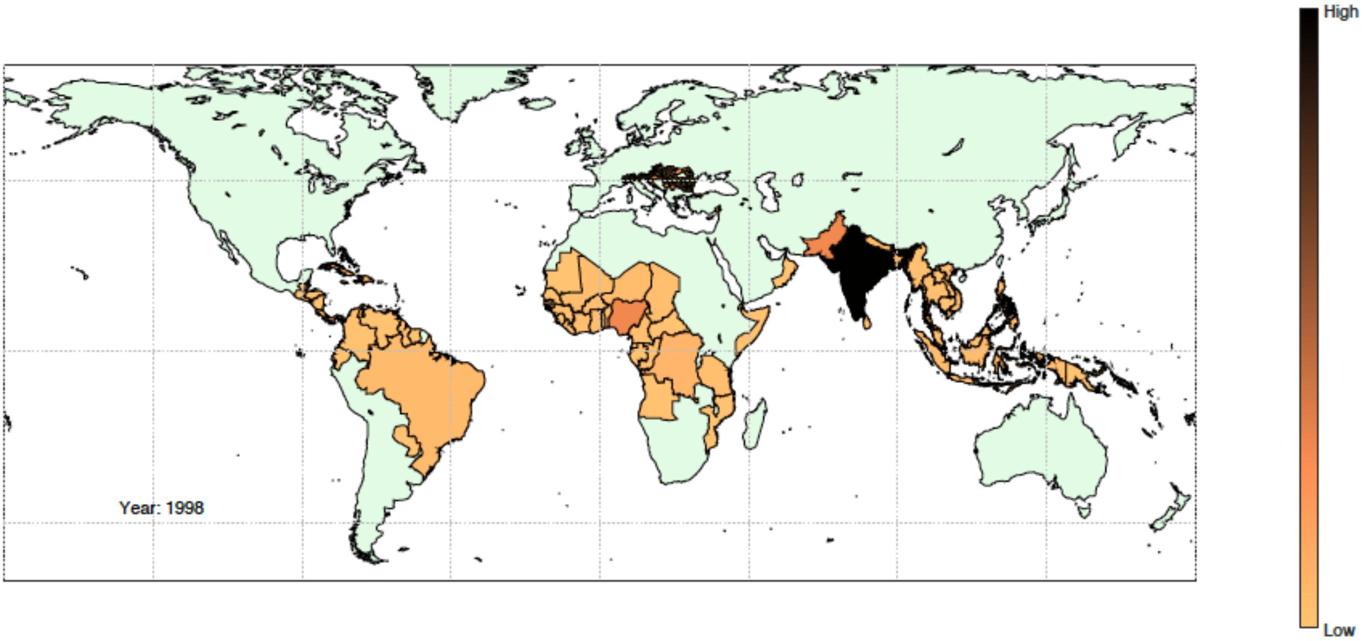

b

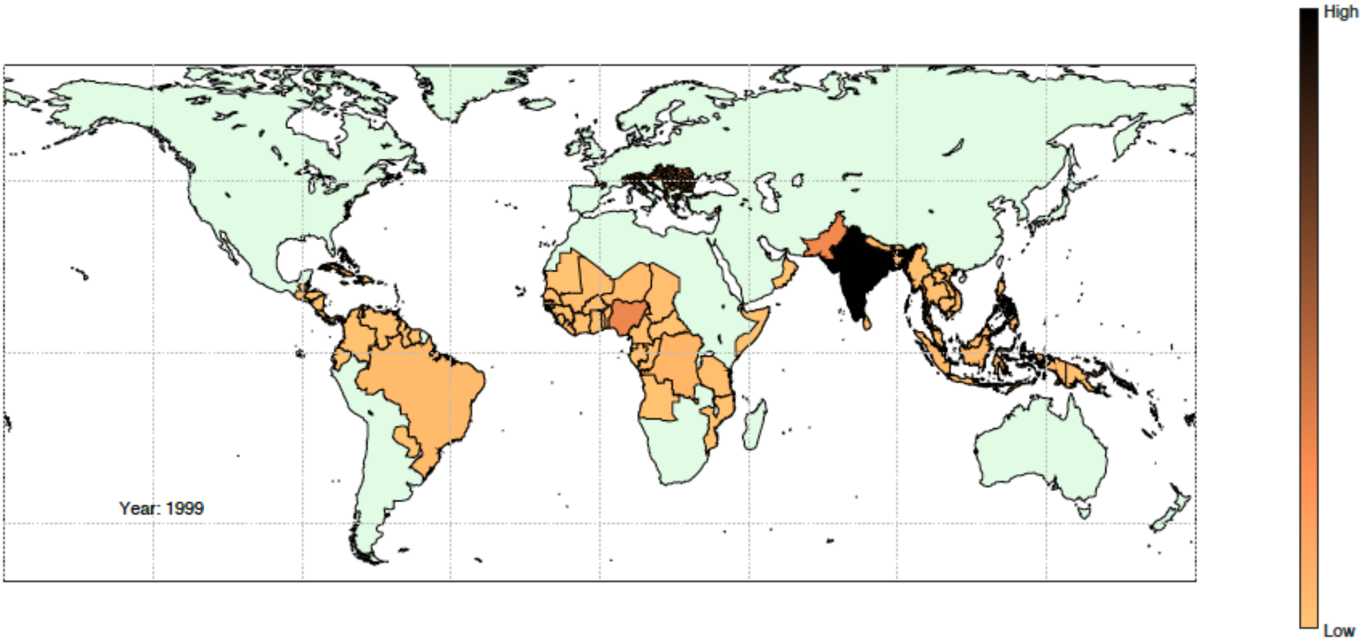

Figure 1.47 World map showing dengue-endemic export regions (countries) outside of Europe, and environmentally suitable import regions (NUTS-3) in Europe, for the respective years (1998, 1999) as indicated by in-figure annotations. The degree of shading, ranging from low to high as shown in the bar legend, indicates a value that is proportional to the number of infected inbound travellers to any given import region from export regions (for NUTS-3 regions), or a value that is proportional to the number of infected outbound travellers from export countries (for countries outside of Europe).

a

b

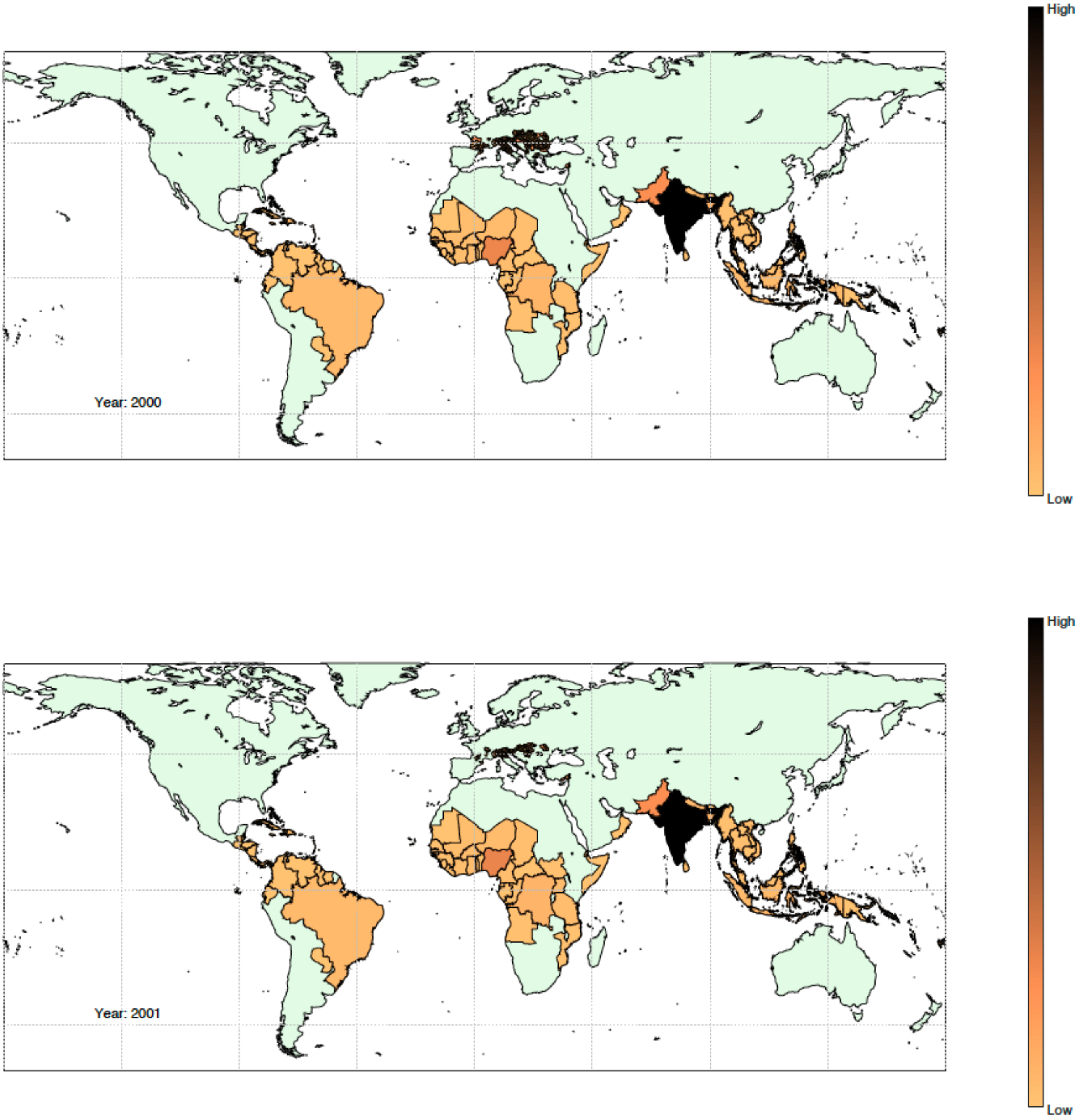

Figure 1.48 World map showing dengue-endemic export regions (countries) outside of Europe, and environmentally suitable import regions (NUTS-3) in Europe, for the respective years (2000, 2001) as indicated by in-figure annotations. The degree of shading, ranging from low to high as shown in the bar legend, indicates a value that is proportional to the number of infected inbound travellers to any given import region from export regions (for NUTS-3 regions), or a value that is proportional to the number of infected outbound travellers from export countries (for countries outside of Europe).

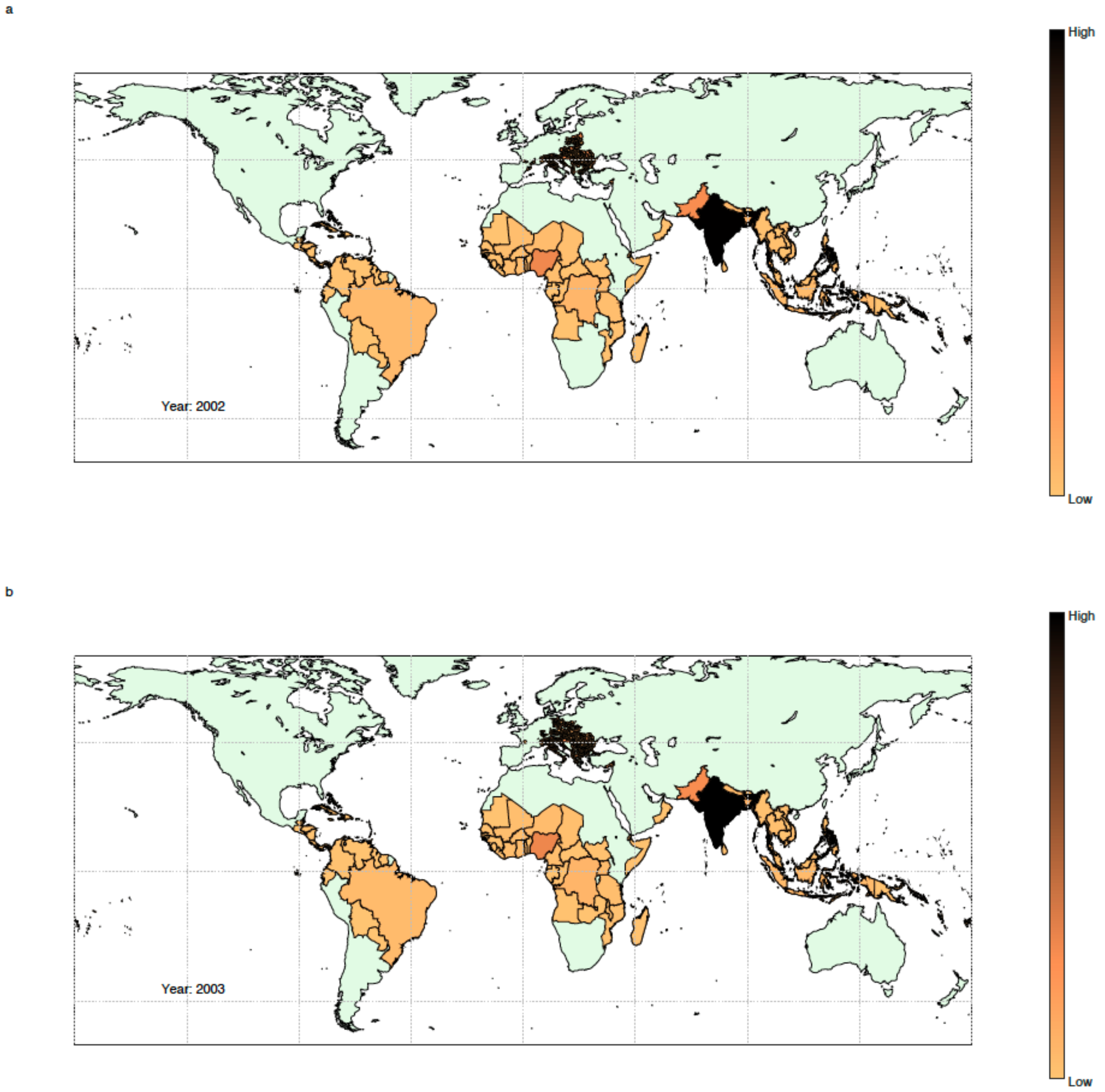

Figure 1.49 World map showing dengue-endemic export regions (countries) outside of Europe, and environmentally suitable import regions (NUTS-3) in Europe, for the respective years (2002, 2003) as indicated by in-figure annotations. The degree of shading, ranging from low to high as shown in the bar legend, indicates a value that is proportional to the number of infected inbound travellers to any given import region from export regions (for NUTS-3 regions), or a value that is proportional to the number of infected outbound travellers from export countries (for countries outside of Europe).

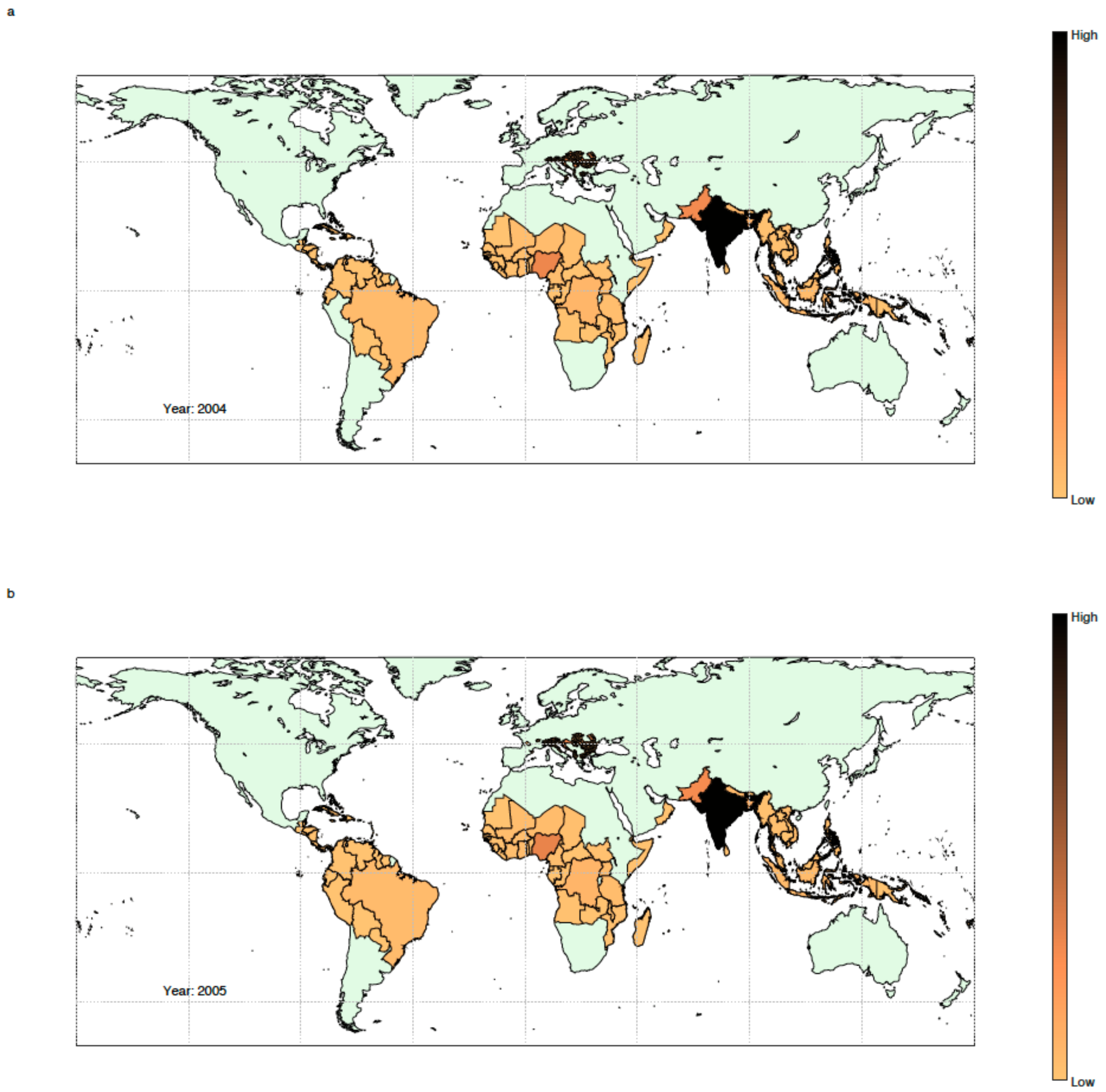

Figure 1.50 World map showing dengue-endemic export regions (countries) outside of Europe, and environmentally suitable import regions (NUTS-3) in Europe, for the respective years (2004, 2005) as indicated by in-figure annotations. The degree of shading, ranging from low to high as shown in the bar legend, indicates a value that is proportional to the number of infected inbound travellers to any given import region from export regions (for NUTS-3 regions), or a value that is proportional to the number of infected outbound travellers from export countries (for countries outside of Europe).

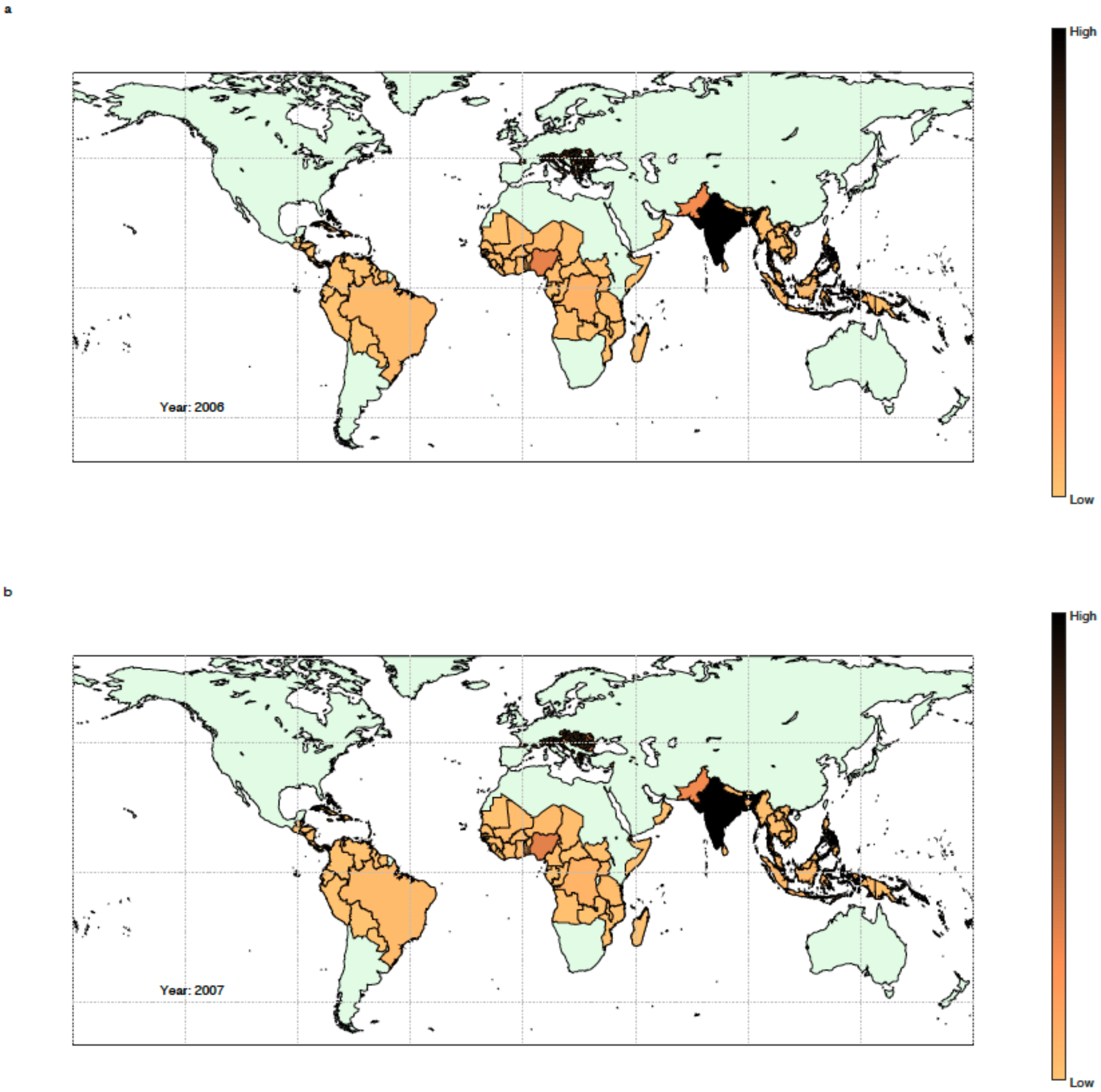

Figure 1.51 World map showing dengue-endemic export regions (countries) outside of Europe, and environmentally suitable import regions (NUTS-3) in Europe, for the respective years (2006, 2007) as indicated by in-figure annotations. The degree of shading, ranging from low to high as shown in the bar legend, indicates a value that is proportional to the number of infected inbound travellers to any given import region from export regions (for NUTS-3 regions), or a value that is proportional to the number of infected outbound travellers from export countries (for countries outside of Europe).

a

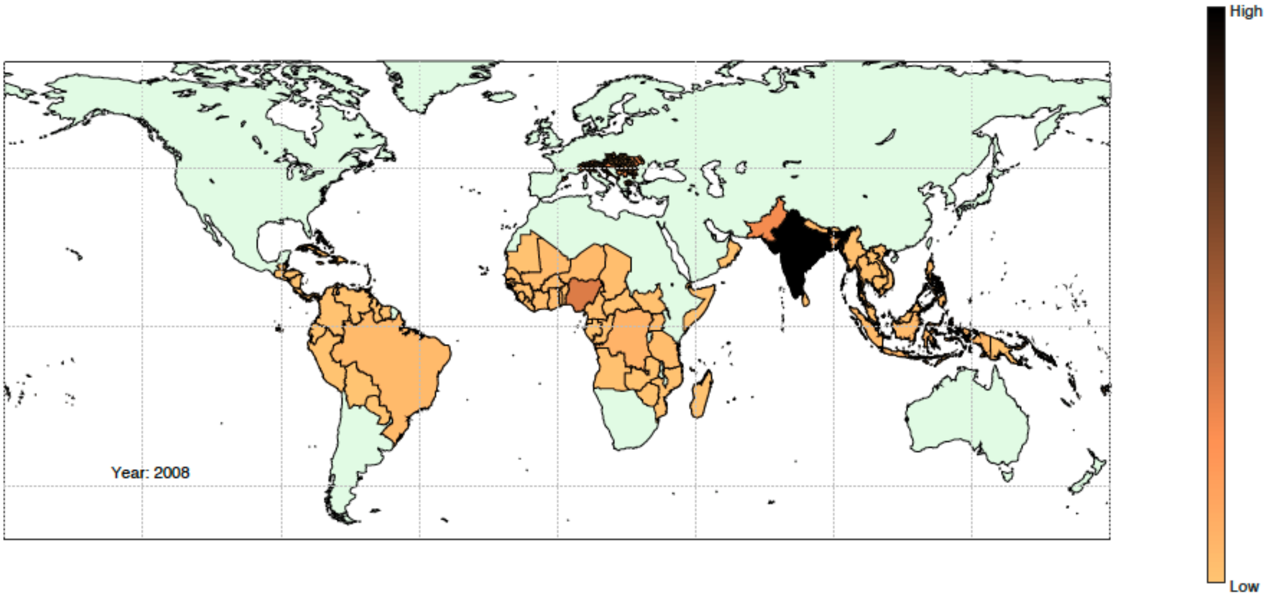

b

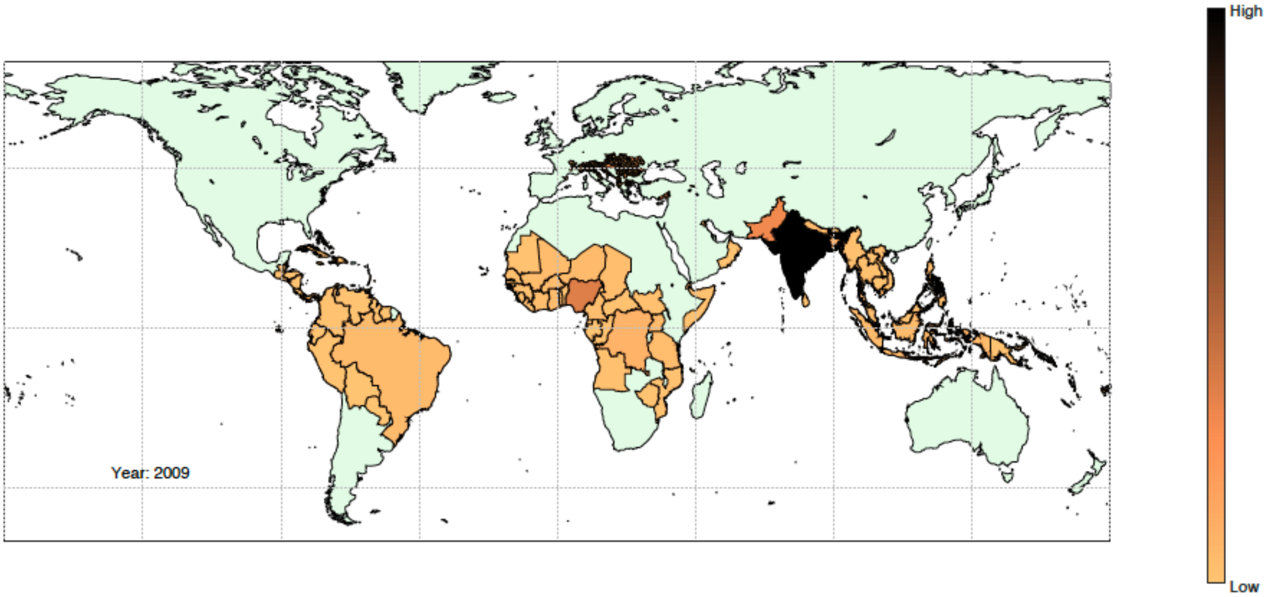

Figure 1.52 World map showing dengue-endemic export regions (countries) outside of Europe, and environmentally suitable import regions (NUTS-3) in Europe, for the respective years (2008, 2009) as indicated by in-figure annotations. The degree of shading, ranging from low to high as shown in the bar legend, indicates a value that is proportional to the number of infected inbound travellers to any given import region from export regions (for NUTS-3 regions), or a value that is proportional to the number of infected outbound travellers from export countries (for countries outside of Europe).

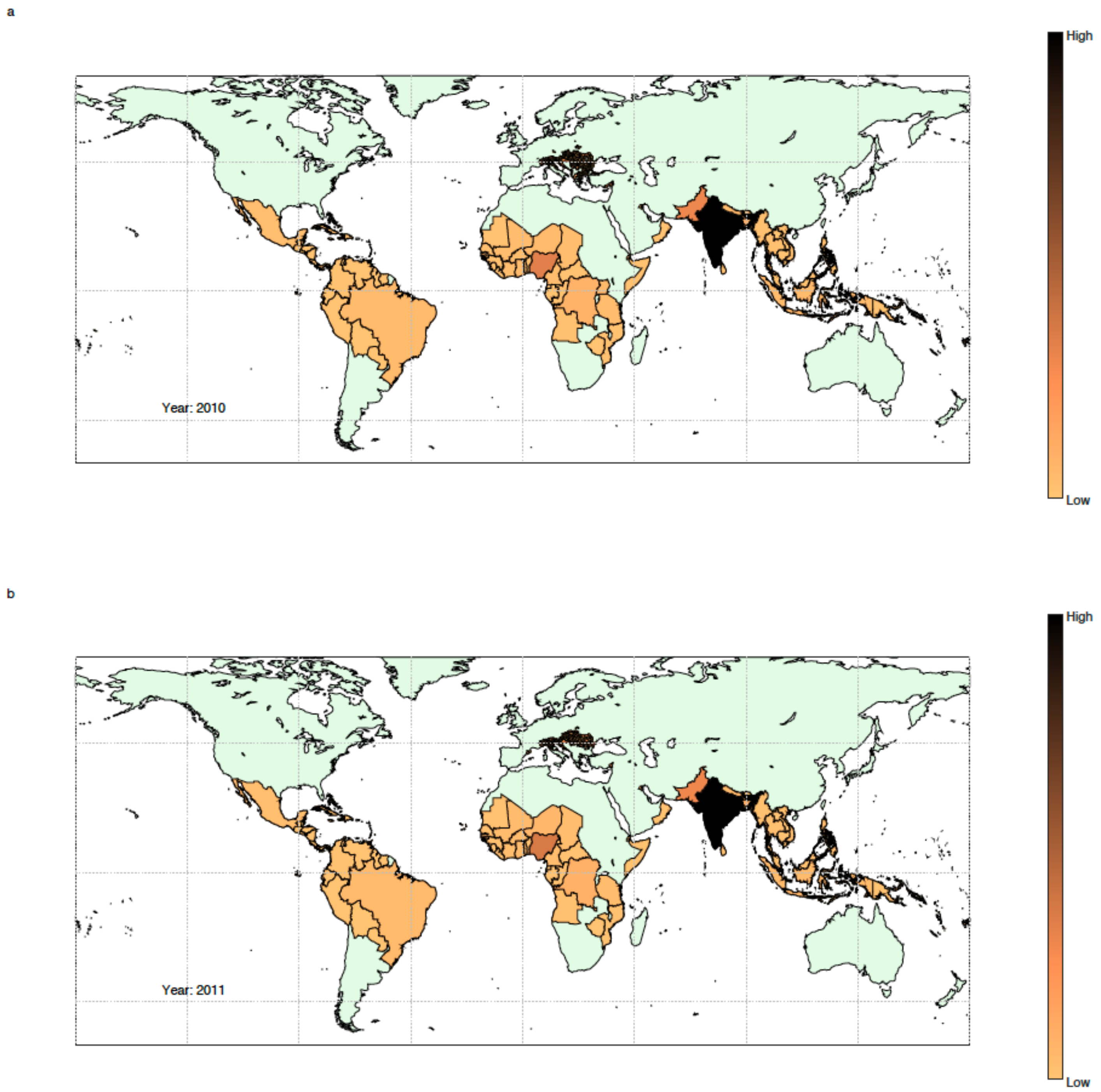

Figure 1.53 World map showing dengue-endemic export regions (countries) outside of Europe, and environmentally suitable import regions (NUTS-3) in Europe, for the respective years (2010, 2011) as indicated by in-figure annotations. The degree of shading, ranging from low to high as shown in the bar legend, indicates a value that is proportional to the number of infected inbound travellers to any given import region from export regions (for NUTS-3 regions), or a value that is proportional to the number of infected outbound travellers from export countries (for countries outside of Europe).

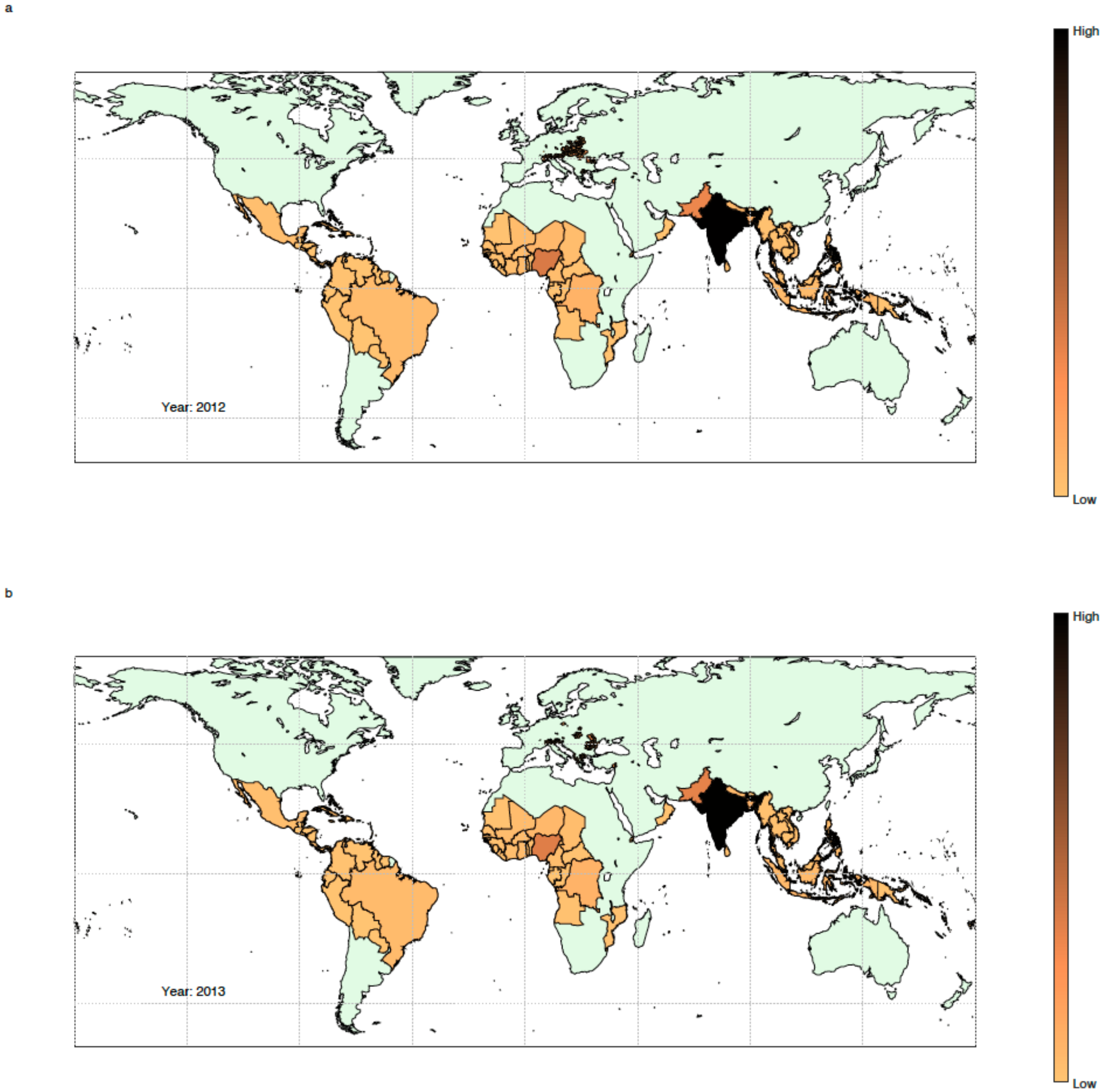

Figure 1.54 World map showing dengue-endemic export regions (countries) outside of Europe, and environmentally suitable import regions (NUTS-3) in Europe, for the respective years (2012, 2013) as indicated by in-figure annotations. The degree of shading, ranging from low to high as shown in the bar legend, indicates a value that is proportional to the number of infected inbound travellers to any given import region from export regions (for NUTS-3 regions), or a value that is proportional to the number of infected outbound travellers from export countries (for countries outside of Europe).

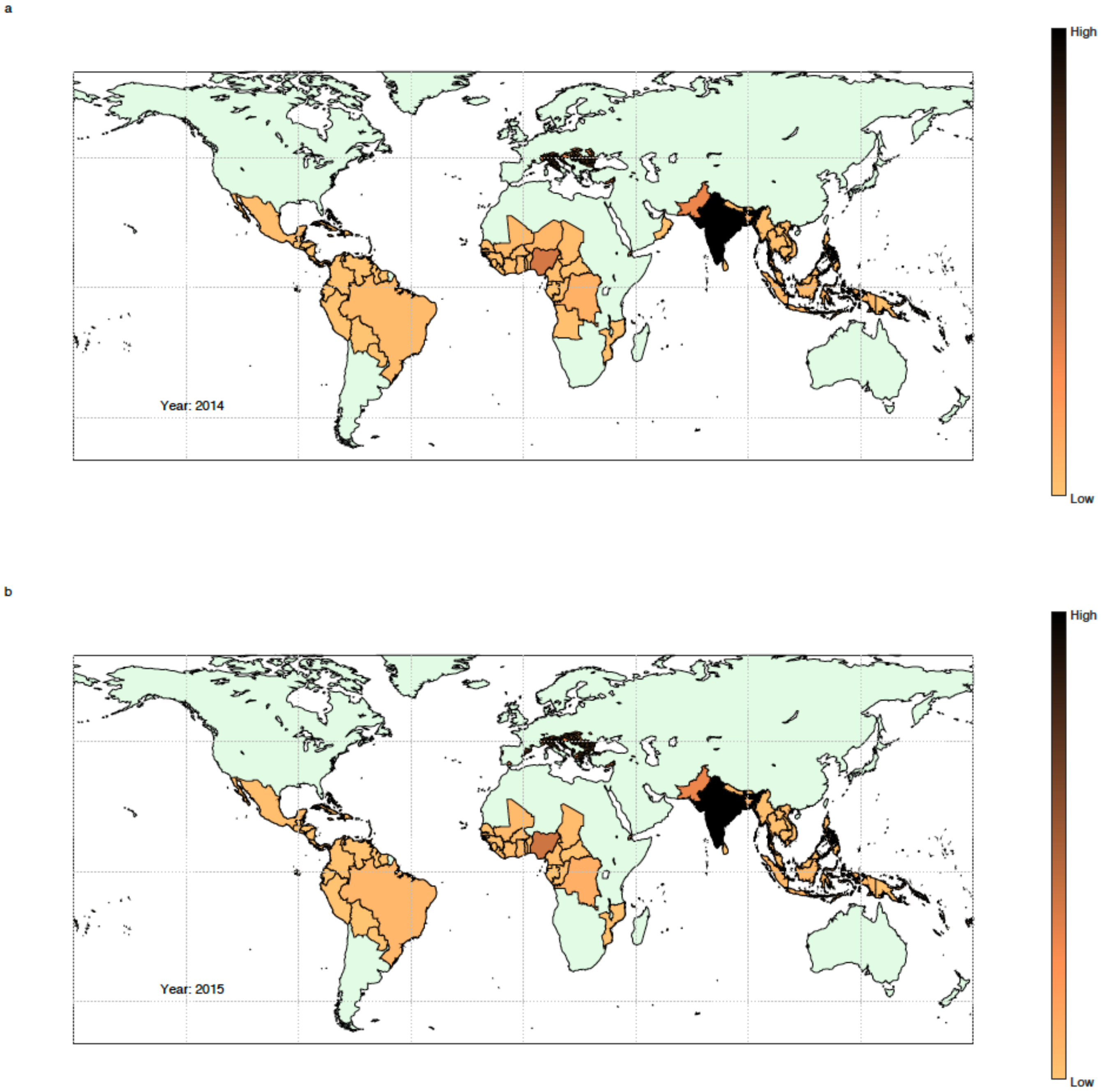

Figure 1.55 World map showing dengue-endemic export regions (countries) outside of Europe, and environmentally suitable import regions (NUTS-3) in Europe, for the respective years (2014, 2015) as indicated by in-figure annotations. The degree of shading, ranging from low to high as shown in the bar legend, indicates a value that is proportional to the number of infected inbound travellers to any given import region from export regions (for NUTS-3 regions), or a value that is proportional to the number of infected outbound travellers from export countries (for countries outside of Europe).

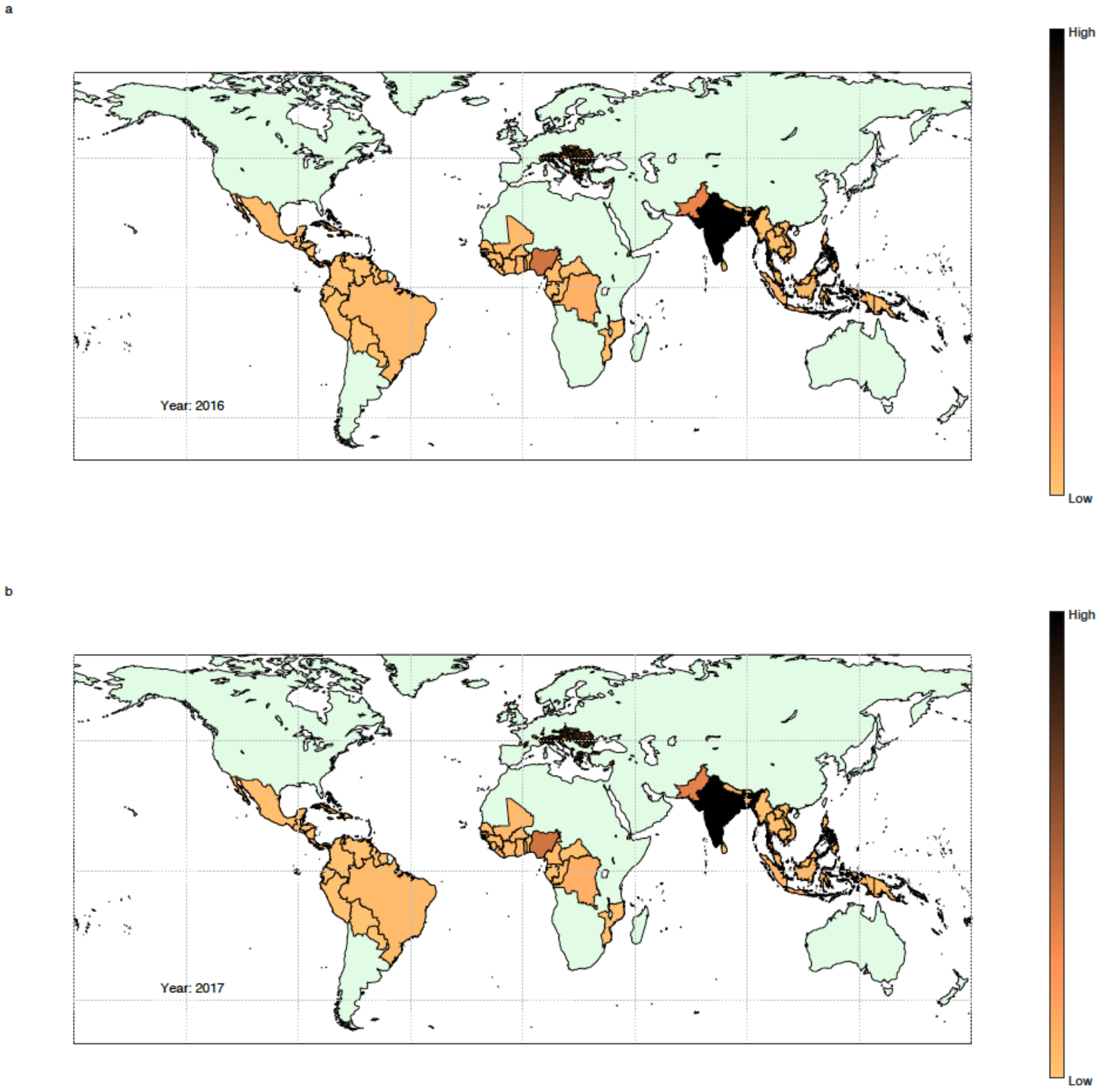

Figure 1.56 World map showing dengue-endemic export regions (countries) outside of Europe, and environmentally suitable import regions (NUTS-3) in Europe, for the respective years (2016, 2017) as indicated by in-figure annotations. The degree of shading, ranging from low to high as shown in the bar legend, indicates a value that is proportional to the number of infected inbound travellers to any given import region from export regions (for NUTS-3 regions), or a value that is proportional to the number of infected outbound travellers from export countries (for countries outside of Europe).

a

b

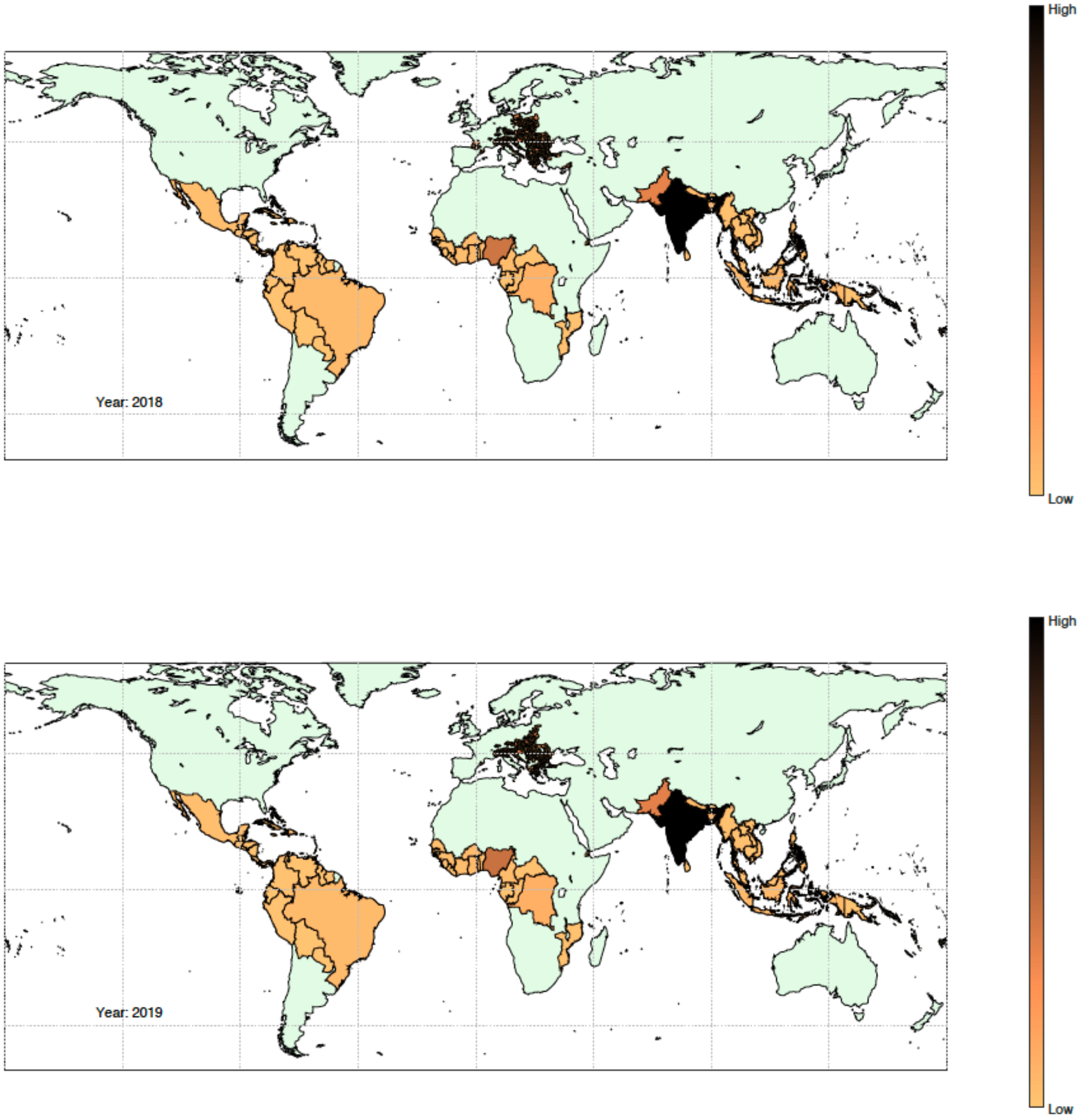

Figure 1.57 World map showing dengue-endemic export regions (countries) outside of Europe, and environmentally suitable import regions (NUTS-3) in Europe, for the respective years (2018, 2019) as indicated by in-figure annotations. The degree of shading, ranging from low to high as shown in the bar legend, indicates a value that is proportional to the number of infected inbound travellers to any given import region from export regions (for NUTS-3 regions), or a value that is proportional to the number of infected outbound travellers from export countries (for countries outside of Europe).

Indicator 1.3.4: Climate suitability Malaria

Background

Malaria is widely recognised as a climate-sensitive infectious disease due to the climate sensitivity observed in both the vector, *Anopheles* mosquitoes, and the *Plasmodium* parasites.[76] Although there are five species within the *Plasmodium* genus, two of them are of major public health concern: *Plasmodium vivax* and *Plasmodium falciparum*.[77]

Until the implementation of the Global Malaria eradication program, Europe was endemic to malaria, with *P. vivax* being the cause of malaria cases in the continent.[77] Since 1974, Europe has been malaria free officially, although cases have been reported among travellers or as part of sporadic local transmission events in Germany, the Netherlands, Spain, France, Italy, Greece and the UK.[78] Among the determinants of malaria elimination in the continent, socio-economic conditions and increased life expectancy in the population have been recognised as main factors allowing Europe to prevent the re-emergence of malaria.[79] In contrast, climatic conditions have become more permissive to the surge of mosquito-borne diseases, indicating that climate had a minor role in removing malaria from Europe.[79,68]

P. vivax and *P. falciparum* differ slightly in their temperature requirements for development inside *Anopheles* mosquitoes. While *P. vivax* requires temperatures between 14.5ºC and 33ºC, *P. falciparum's* extrinsic incubation ranges from 18ºC to 32ºC. Additionally, relative humidity needs to be greater than 60% to allow mosquitoes to survive long enough to carry infectious parasites. Rainfall and availability of water bodies are necessary for adult mosquitoes to lay eggs and for larvae to survive.[80] Evidence suggests that at least 80 mm of monthly rainfall is necessary for suitable transmission.[81] Moreover, land type is a determinant of the spatial distribution of *Anopheles* mosquitoes, as restrictive biomes prevent populations from successfully settling down.[82]

As climate projections suggest a more permissive climate for disease transmission, monitoring environmental and climatic conditions in Europe is vital for detecting increases in climate suitability that could require rapid decision-making to prevent malaria from returning. For this purpose, an adaption to the European context of the malaria climate-suitability indicator, published in the *Lancet* Countdown reports, is presented here at a finer scale and considering local conditions.[83,84,85,86]

Methods

Although *P. vivax* has historically been the cause of autochthonous malaria cases in Europe, there have been reports of infections with *P. falciparum* in Germany and The Netherlands, without travel history.[87,88] As a result, the malaria suitability indicator in this report estimates the number of months suitable for transmission of each parasite, calculated from empirically derived thresholds of precipitation, temperature and relative humidity.

Monthly climate information between 1951 and 2020 were obtained from the ERA5-Land repository at a 9km^2 resolution.[89] Relative humidity (%) was calculated using the August-Roche-Magnus equation, which derives this value by combining dew point temperature and temperature, using the formula.[90]

$$RH = 100 * \frac{\exp\left(\frac{aT_d}{b+T_d}\right)}{\exp\left(\frac{aT}{b+T}\right)}$$

Where a and b are the coefficients 17.625 and 243.04, respectively, and T and T_d are temperature and dew point temperature in °C.

Elevation data were extracted from the JISAO repository, University of Washington (http://research.jisao.washington.edu/data_sets/elevation/). Land cover data were downloaded from the Copernicus Land Monitoring Service project repository at 100m resolution (https://land.copernicus.eu/pan-european/corine-land-cover). The land cover raster from 2018 was downloaded and assumed to be constant throughout the time series. Land classes were determined according to the criteria used by Benalli and colleagues[82] to define environments suitable for *Anopheles* breeding , namely rice fields, permanently irrigated croplands and sport and leisure facilities (figure 1.58).

Suitability for a particular month was defined as the coincidence of precipitation accumulation greater than 80 mm, average temperature between 14.5°C and 33°C for *P. vivax* and between 18°C and 32°C for *P. falciparum*, relative humidity greater than 60%, and highly suitable land class. These combined values reflected the limits for potential transmission of each of the mentioned *Plasmodium* parasites. The number of months with suitable conditions was calculated at the finest possible resolution, nine kilometres, and later averaged to European region (figure 1.59), country and NUTS-2 level. The yearly products were later stratified by elevation using a threshold of 700 m.a.s.l. for splitting low- from highland (highlands >= 700 m.a.s.l.).

Results were visualised using time series line plots and maps containing the percentage changes between the periods 1951-1985 and 1986-2020.

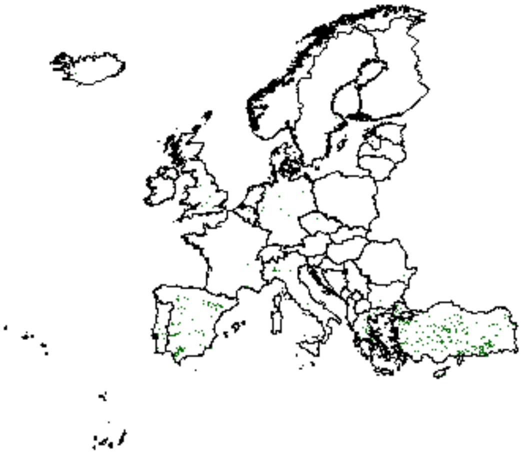

Figure 1.58 Highly suitable environments for Anopheles mosquito breeding. Grid cells were classified as highly suitable according to Benalli *et al.*, 2014. Highly suitable areas were rice fields, permanently irrigated croplands and sport and leisure facilities.

.

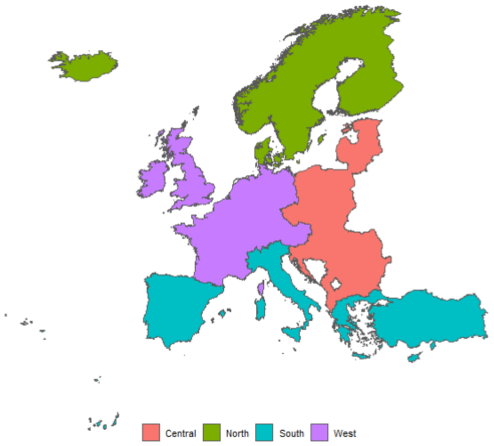

Figure 1.59 European regions

Geographic coverage of Europe

This indicator was computed for all member and cooperating countries included in the European Environment Agency (EEA), plus the United Kingdom.

Data

Variable	Source	Frequency of update	Spatial resolution	Temporal range
Monthly 2-meter dew point temperature	ERA5-Land	Monthly with a 3-month delay relative to present	0.1°/ 9 km^2	Jan 1951 to Dec 2020
Monthly 2-meter temperature	ERA5-Land	Monthly with a 3-month delay relative to present	0.1°/ 9 km^2	Jan 1951 to Dec 2020
Monthly total precipitation	ERA5-Land	Monthly with a 3-month delay relative to present	0.1°/ 9 km^2	Jan 1951 to Dec 2020
Land cover	CORINE	Every 6 years	100 m	2018
Altitude	JISAO	-	0.5°	-

Caveats

This indicator reflects the state of conditions that would potentially allow malaria transmission to occur, had there not been public health efforts to control it. In this regard, this is indicator should be interpreted in the context of the current efforts that are allowing Europe to keep malaria from returning to the continent.

Future form of the indicator

Malaria re-emergence in the continent is highly related to the risk of importation, as *Plasmodium* parasites are still endemic to other parts of the world. This information is important to be included in future versions of this indicator in the form of human mobility, density and/ or incoming flights from high-risk areas.

Results and additional analysis

Overall, there has been an increasing trend in the number of months suitable for malaria transmission in Europe since 1951, in lands highly suitable for *Anopheles* mosquito breeding (figure 1.60 A). The trend was maintained after stratifying by European regions (figure 1.60 B).

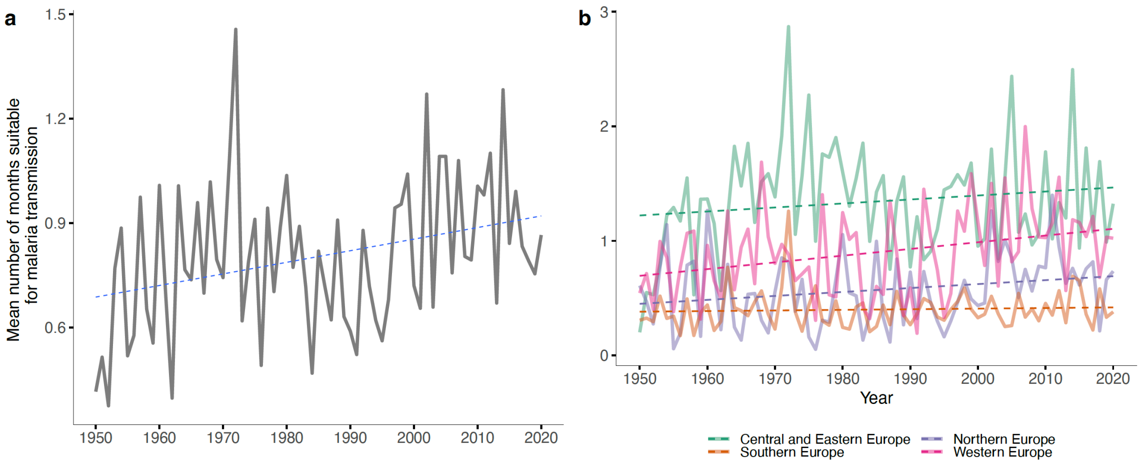

Figure 1.60 Mean number of months suitable for *P. vivax* transmission between 1951 and 2020, (a) for the entire continent and (b) grouped by European regions. The number of suitable months was calculated as the number of months per year in with precipitation above 80 mm, average temperature between 14.5°C and 33°C, and relative humidity above 60%, in land types highly suitable for Anopheles mosquitoes. Linear regression was used for trend estimation.

Central and Western Europe have had the highest mean suitability throughout the considered period. In the period 1986-2020, Europe's overall suitability increased in 4.5%, compared to the period 1951-1985. The highest increase was observed in Northern and Western Europe, with 21.6% and 25.2%, respectively (table 1.4). Particularly, the United Kingdom and Ireland showed marked rises between periods, with 106% and 104%, respectively (table 1.5). Several countries indicated an overall decrease in malaria suitability since 1951. Romania, for example, had a marked decrease in suitability for malaria transmission of 25.8% in between the considered periods (figure 1.61).

Table 1.4 Percentage change in mean number of months suitable for malaria transmission, when comparing the period 1951-1985 to the period 1986-2020, stratified by European region

European region	Percentage change (%)
North	21.6%
Central	-5.27%
South	2.84%
West	25.2%

Table 1.5 Top five countries with highest percentage change in mean number of months suitable for malaria transmission, when comparing the period 1951-1985 to the period 1986-2020

Country	Percentage change (%)
United Kingdom	106%
Ireland	104%
Portugal	55.9%
Norway	52%
Sweden	29.7%

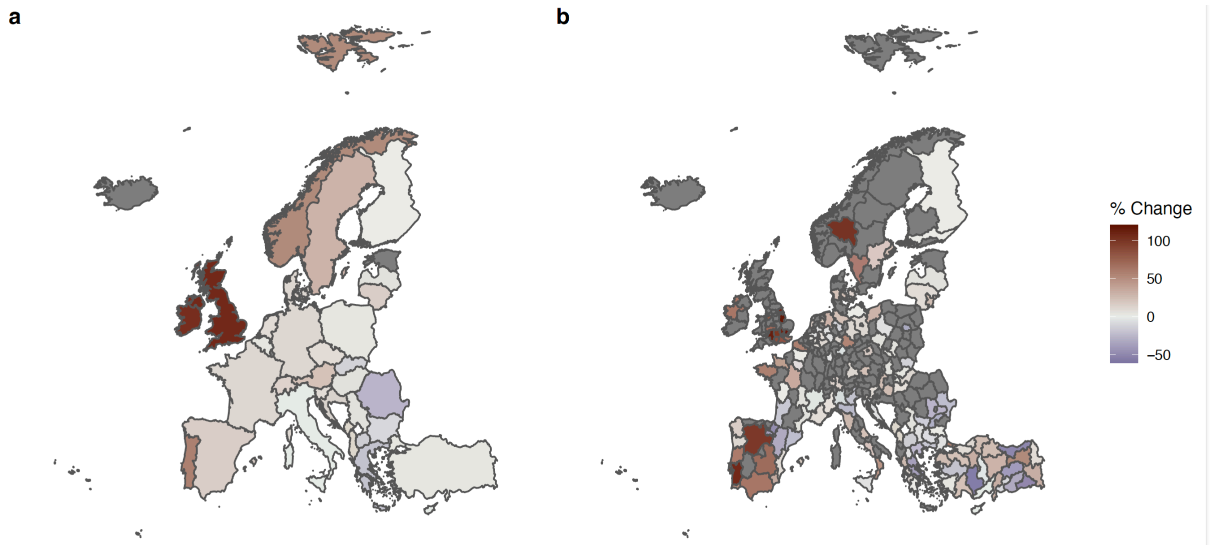

Figure 1.61 Percentage change in the mean number of months suitable for *P. vivax* transmission, when comparing the decade 1951-1960 to the decade 2011-2020. Outcomes are grouped by (a) country and (b) NUTS 2 regions.

Suitability at altitudes 700 m.a.s.l. and above increased in Central and Western Europe, which concentrate most of the highlands in the continent, while it remained constant in Northern and Southern Europe (figure 1.62). On the other hand, lowlands, i.e., altitude below 700 m.a.s.l., had an increasing trend in all regions since 1951.

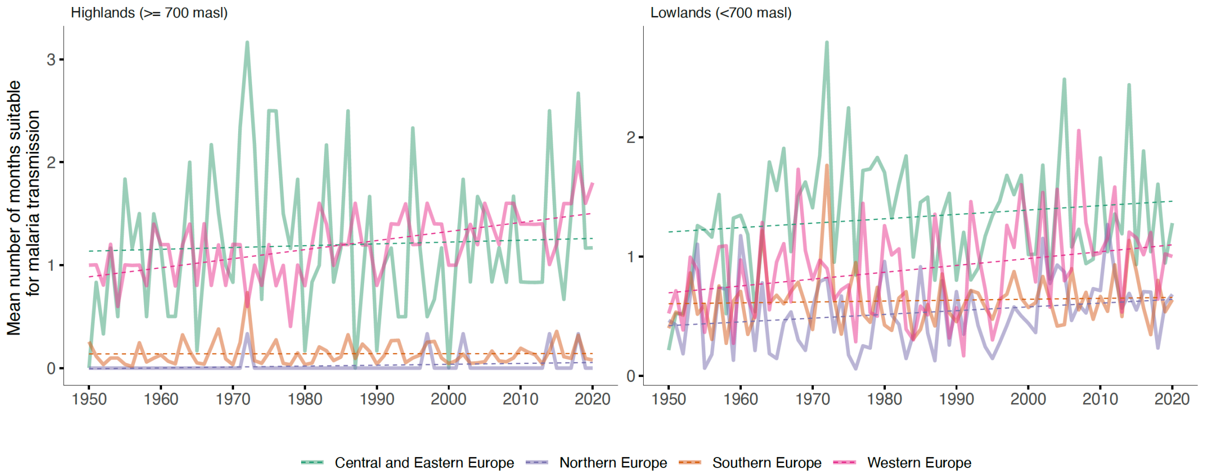

Figure 1.62 Mean number of months suitable for *P. vivax* transmission between 1951 and 2020, grouped by European regions and stratified by high-/ lowlands. The number of suitable months was calculated as the number of months per year in with precipitation above 80 mm, average temperature between 14.5°C and 33°C, and relative humidity above 60%, in land types highly suitable for Anopheles mosquitoes. Linear regression was used for trend estimation.

Stratifying the indicator by a NUTS 2 level highlighted a marked heterogeneity in the spatial distribution of the suitability. Central, Northern and Eastern Europe had districts that surpassed the 300% increase in the number of months suitable for malaria transmission (figure 1.60 B)

Although *P. vivax* has historically been the cause of autochthonous malaria cases in Europe, there have been reports of infections with *P. falciparum* in Germany and The Netherlands, without travel.[87,88] As a result, the suitability indicator for the latter was also calculated from empirically derived thresholds of precipitation, temperature and relative humidity.

Suitability for *P. falciparum* has increased slightly since 1951 in Northern, Southern and Western Europe, although there was a marked increase in Central Europe (figure 1.63 (a)). Similarly, to *P. vivax*, there is heterogeneity in *P. falciparum*'s spatial distribution, with The Netherlands, Belgium, Germany, Switzerland and Austria concentrating the highest percentage change, when comparing the period 1951-1985 to the period 1986-2020 (figure 1.63 (b,c)).

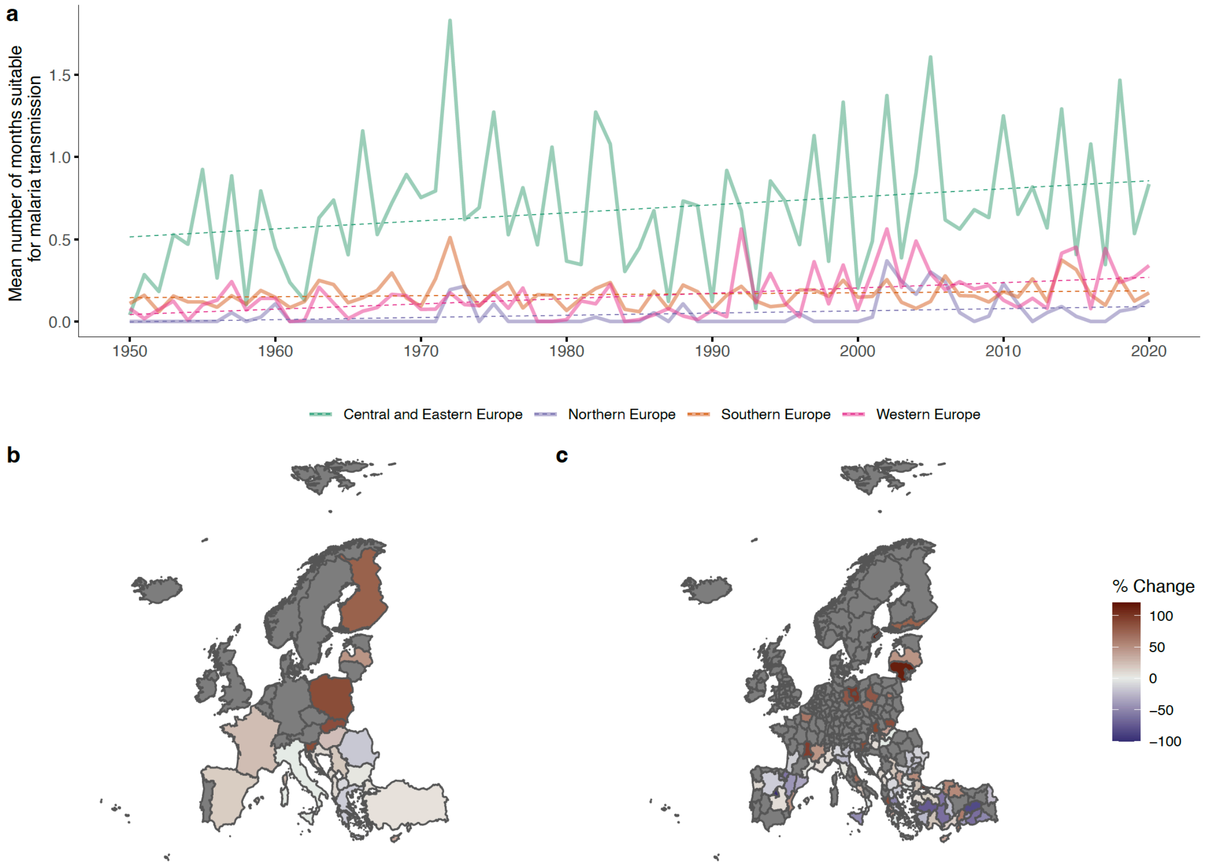

Figure 1.63 (a) Mean number of months suitable for *P. falciparum* transmission between 1951 and 2020, grouped by European regions. The number of suitable months was calculated as the number of months per year with precipitation above 80 mm, average temperature between 18°C and 32°C, and relative humidity above 60%, in land typed highly suitable for *Anopheles* mosquitoes. Linear regression was used for trend estimation. (b, c) Percentage change in the mean number of months suitable for *P. falciparum* transmission, when comparing the period 1951-1985 to the period 1986-2020, stratified by (b) country and (c) NUTS 2 regions.

1.4: Allergens

Indicator 1.4.1: Allergenic trees

Method

Rising temperature leads to substantial changes in the flowering season of European trees, releasing large amounts of allergenic pollen into the air. Some pollen proteins can exacerbate allergenic rhinoconjunctivitis (pollinosis) and allergic asthma by acting as antigens for the immune system.[91] The most consistent signal is visible at the start of the season, the target variable of the current indicator. The start of the pollen season is relevant also because patients are alerted in many regions by warning systems and mass media.

Pollen season has many definitions, depending on the target use.[92] The definition relevant in the current context is a so-called "clinically-relevant" pollen season, i.e., the period when concentrations of a specific pollen are sufficiently high to cause allergy symptoms. The general definition and the concentration thresholds for several aeroallergens were identified by Pfaar *et al.* 2016[93] as a result of a Task Force of the European Academy of Allergology and Clinical Immunology (EAACI).

Calculation of the clinically-relevant season is performed in several steps using the SILAM atmospheric composition model (Sofiev *et al.* 2015[94] and 2012[95] http://silam.fmi.fi, visited 5.3.2022; open-source code http://github.com/fmidev/silam-model visited 5.3.2022):

1. phenological season of trees is computed by SILAM based on the concept of accumulated heat as a period when a specific plant releases pollen into the atmosphere;
2. atmospheric dispersion of the released pollen is computed by SILAM, thus obtaining pollen concentration as a function of time across Europe;
3. clinically-relevant season definition of Pfaar *et al.* 2016[93] is calculated based on the SILAM-computed concentrations as input;
4. the calculations are repeated independently for each year through 1980-2020 and each type of tree;
5. differences of the last/first decadal medians of the clinicallyrelevant season start day are calculated for each grid cell or NUTS 2 region.

Details of each step are given below.

1. Phenological season

SILAM currently possesses phenological parameterizations for three European trees: alder, birch, and olive.[96] For all of them, the phenological season is computed based on the concept of accumulated heat as the main trigger and the driver of the flowering season.[95,97,98] For heat accumulation, the Daily Temperature Sum model is written in the following form[95]:

(1) $$H(D) = \sum_{d=D_s}^{D} \left(\overline{T(d)} - T_{co}\right) * U\left(\overline{T(d)} - T_{co}\right)$$

where D is day, D_s is the day of start of heat accumulation, $T(d)$ is daily mean temperature for the day d, T_{co} is cut-off temperature, and U is unity cut-off function:

$$U(x) = \begin{cases} 0, & x \leq 0 \\ 1, & x > 0 \end{cases} \quad (2)$$

Equations (1) – (2) imply that the heat accumulation rate is proportional to an excess of daily-mean temperature above a cut-off level. Same equations can be written also for hourly averaging, which for alder leads to a better agreement with the observed phenological timing than the daily model.

Phenological flowering season starts at the day D_s when heat sum exceeds a start-season threshold: $H(D_s) > H_{start}$. The season ends at the day when the accumulated heat reaches the end-season threshold: $H(D_e) > H_{end}$. For all trees, these thresholds depend on location, i.e., they are represented via maps rather than scalar values. This approach reflects the variety of the European climate zones as well as the marine-vs-continental climate gradient. The thresholds were empirically identified based on long-term SILAM simulations and pollen measurements across Europe by the European Aeroallergen Network (Sofiev *et al.* 2012,[95] 2015[99] and 2017[100]).

2. Dispersion computations

Release into the air and atmospheric dispersion of pollen was computed by SILAM driven by the ECMWF ERA5 meteorological reanalysis. Spatial resolution was 0.1° lat-lon for the domain covering the whole of Europe. Output included hourly-mean pollen concentrations, cumulative dry and wet deposition. Details of the computation procedure can be found in Sofiev *et al.* 2012,[95] 2015[99] and 2017.[100]

3. Clinically relevant season

Computations of the clinically-relevant season follows the definition of Pfaar *et al.* 2016[93]: Start of season is the 1st day of 5 days – out of 7 consecutive days – each of these five days with concentration exceeding C_{daily_clin} and with a sum of these five days exceeding C_{5days_clin}.. The authors identified the values of the two constants for 5 species: Birch, Grass, Cypress, Olive, and Ragweed. For this indicator, only Birch and Olive were used (Table .6), whereas the constants for Alder were roughly estimated using mean seasonal exposure to this pollen in comparison to Birch and Olive. For the trend of the season start, this is a satisfactory approximation. For actual season computations, the consensus thresholds for alder must be identified.

Table 1.6 Numerical constants for concentration thresholds, modified from Pfaar *et al.* 2016[93]

	*Alder**	*Birch*	*Olive*
C_{daily_clin}	3 pollen m^{-3}	10 pollen m^{-3}	20 pollen m^{-3}
C_{5days_clin}	30 pollen m^{-3}	100 pollen m^{-3}	200 pollen m^{-3}

Note: The Task Force of Pfaar et al did not identify the constants for Alder. The values were estimated by extrapolating the typical pollen concentrations of birch and olive and comparing them to those of Alder.

4. Multi-annual computations and 5. Trend analysis

The SILAM computations have been made for 1980-2020 using the European Reanalysis ERA5.[60] Each year is computed independently, with the same amount of pollen released as a seasonal integral. Upon completion, the clinically-relevant season start, end, and duration were computed from daily pollen concentrations for each grid

cell and for each NUTS region independently. Based on the obtained time series of season characteristics, the difference between the medians of the first and last decades was calculated at every grid cell/NUTS region.

$$DIFF = MEDIAN_{2011\_2020} - MEDIAN_{1981\_1990}$$

The obtained trends show a significant spatial variability, both in the trend absolute value and its statistical significance (figure 1.64). The high variability has been shown in to originate from year-to-year meteorological variability.[101] However, over the areas with a statistically significant trends (shaded areas in the left column of figure 1.64), the shift of the season reached 10-20 days during the last 40 years. It exceeds one month for e.g., birch season start in the Alpine region. A highly systematic shift was found also for olives: in practically all olive-rich regions, the shift reached about 10 days over last 40 years.

Geographic coverage of Europe

For this indicator we considered the European Union member countries, candidate and potential candidate countries plus the United Kingdom and EFTA countries.

Data

The SILAM computations rely on two major data sets:

1. Meteorological European Reanalysis ERA5[60] of European Centre of Medium-Range Weather Forecasting ECMWF. The period 1980-2020 was used for the indicator. It is based on computations of the ECMWF Integrated Forecasting System, IFS, with assimilation of large amount of in-situ and remote-sensing data. The ERA5 temporal resolution is one hour, and spatial resolution is about 25 km.
2. The global land-use dataset ECOCLIMAP,[102] which provides 1 km global classification of land use. The land-use categories of ECOCLIMAP do not distinguish the individual tree species and do not provide temporal evolution of the land-use. Therefore, the ECOCLIMAP maps were combined with species-specific data of European Forest institute EFI[103] and Global Land Cover data GLC.[104] The final step of adaptation was an inverse problem solution with the SILAM model, which procedure was described by Prank *et al.* 2013,[105] aiming at the climatologically unbiased concentration predictions. The same map was used throughout the simulated period.
3. Evaluation of the model predictions for 2020 was made using the data of European Aeroallergen Network provided to Copernicus Atmosphere Monitoring Service for model evaluation.

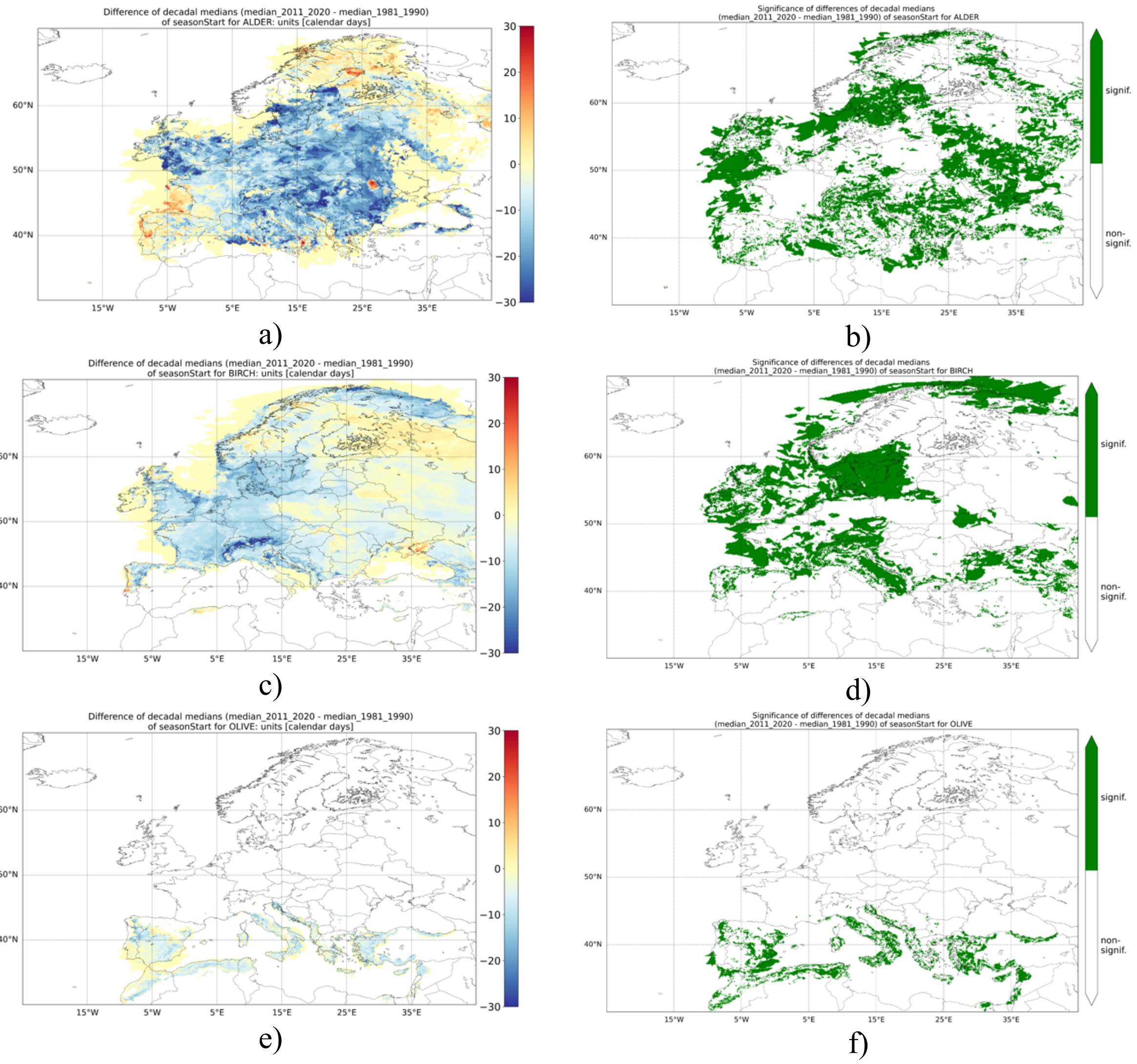

Figure 1.64 Difference of the decadal medians of start of the clinically-relevant season and their statistical significance for alder (upper panel), birch (middle panel), olive (lower panel). Left column: trends, unit: [days]; right column: significance with p-value < 0.1, i.e., trends over all green-colour areas are significant with p<0.1.

Caveats

The indicator employs two major simplifications: (i) the same amount of pollen is released into the air every year; (ii) the same vegetation distribution map is applied for all years.

The clinically relevant season depends not only on the phenological season but also on absolute pollen concentrations. For trees, strong fluctuations between years are common: strong-pollination years are often followed by weak years and vice versa. However, predicting the absolute level of pollen season for each specific year is a very difficult task, and no adequate European-wide solution exists for it this-far. The first-ever regional model of Ritberga *et al.* 2017[106] covers only Northern Europe. Therefore, the indicator is based on the season timing only, always assuming that there is enough pollen for the clinical relevance. This assumption is reasonable in the areas of the main sources of specific pollen, but could lead to increased uncertainty in remote areas.

However, the season start is, in most cases, sharp and well synchronised over a substantial territory, i.e., the clinical relevance will anyway be achieved at practically the same day, whether the season is low or high.
The fixed vegetation map is also a simplification that is important for the absolute level of pollen concentrations but also less significant for timing of the pollen season, for the same reason.

Future form of the indicator

The indicator will be developed in four directions.
The inter-annual season-strength forecasting model will be expanded to the whole of Europe and applied to obtain a more realistic season severity prediction for each specific year.
The fixed land-use map will be switched to a series of annual maps, each adjusted to the specific forest distribution pattern obtained from satellites for each specific year. It is anticipated that such maps will not be available for the pre-satellite era, but an effort will be made to obtain reference distributions at least every 5-10 years from the in-situ observations and inventories of EFI.
Work is on-going to develop an operational procedure for pollen data assimilation.[107] Upon finalization of this technology, the absolute level of pollen season will be included in the indicator.
The ERA5 archive is being extended by ECMWF towards 1950. Upon completion of this work, the indicator will be extended to cover additional 30 years in the past. With time passing, the indicator will be updated on annual basis to include the recent-most years.

Additional analysis

Apart from the season start time, the season duration and the season end time are of high interest for allergy sufferers. As follows from Eq. (2) and the definition of Pfaar *et al.* 2016,[93] the season end can be obtained following the same procedure as for the season start. This signal is less articulated in the time series, partly due to pollen resuspension, partly due to regional and long-range transport. However, it is still possible to compute its trends with reasonable statistical significance: figure 1.65.
Comparing figure 1.64 and figure 1.65, one can see that both the season start and end are shifting synchronously in most of European regions, but the start is shifting slightly faster. As a result, the season duration stays almost constant (figure 1.66), with a tendency towards a longer season (albeit not statistically significant).

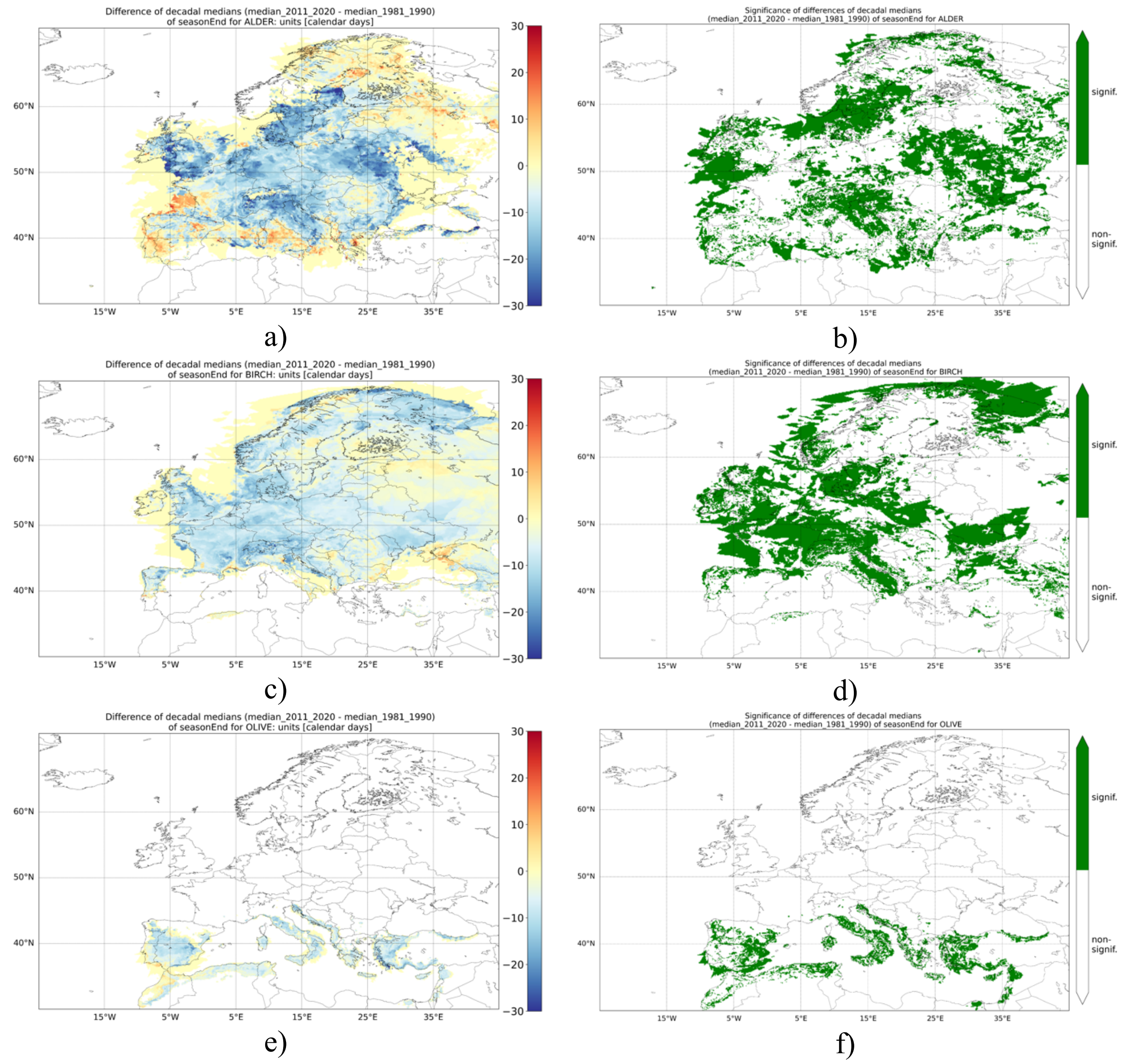

Figure 1.65 Difference of the decadal medians of end of the clinically-relevant season and their statistical significance for alder (upper panel), birch (middle panel), olive (lower panel). Left column: trends, unit: [days]; right column: significance with p-value < 0.1, i.e., trends over all green-colour areas are significant with p<0.1.

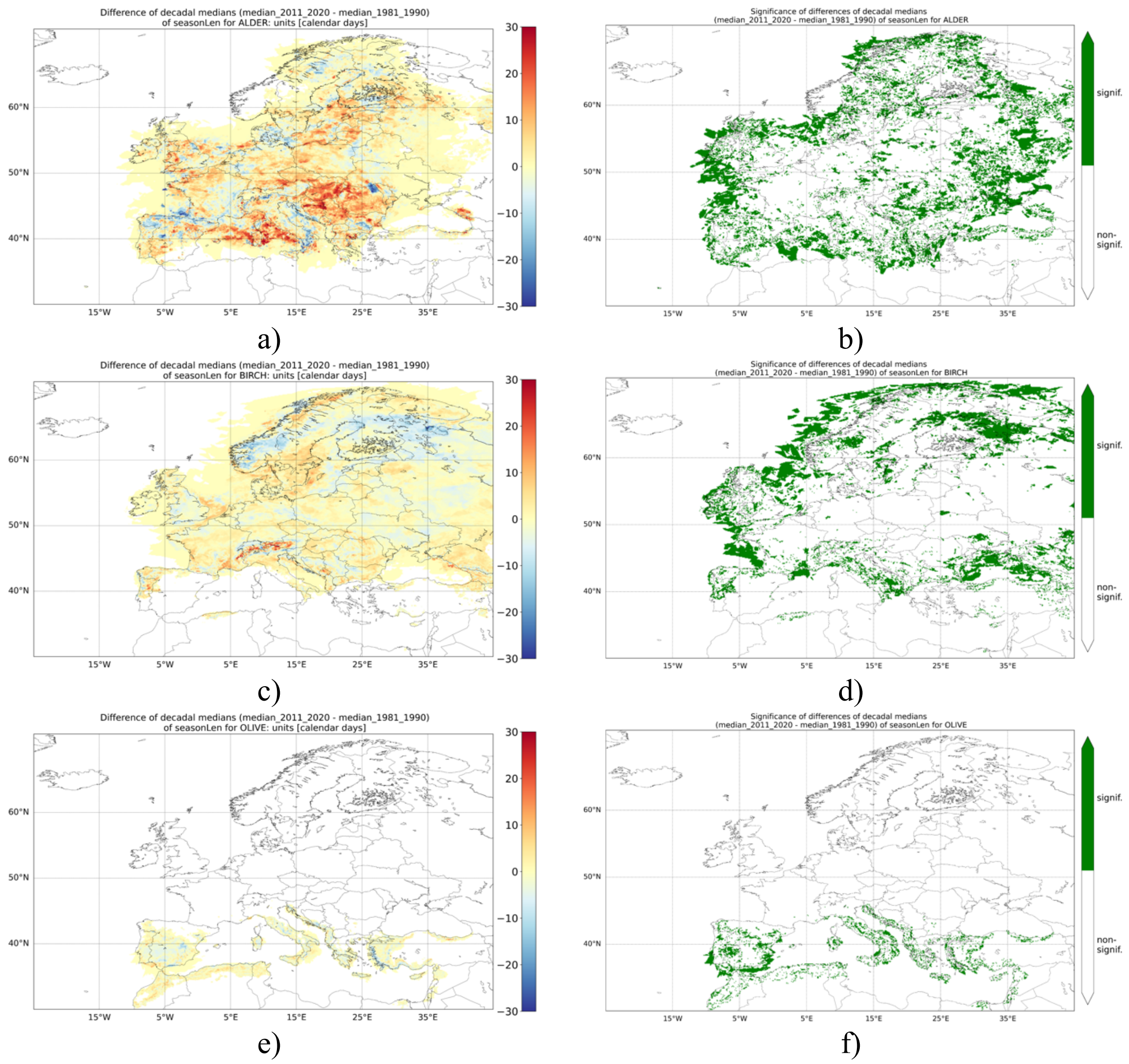

Figure 1.66 Linear trends of duration of the clinically-relevant season and their statistical significance for alder (upper panel), birch (middle panel), olive (lower panel). Left column: trends, unit: [days]; right column: significance with p-value < 0.1, i.e., trends over all green-colour areas are significant with p<0.1.

The SILAM pollen predictions have been extensively evaluated in a series of international projects.[95,99,100,108,109] An example of the last-year evaluation of birch season representation withing the Copernicus Atmosphere Monitoring Service CAMS is shown in figure 1.65. That year, the birch season was very late in Central and Northern Europe, but the model still reproduced the season start within a few days of uncertainty, both the start and the end.

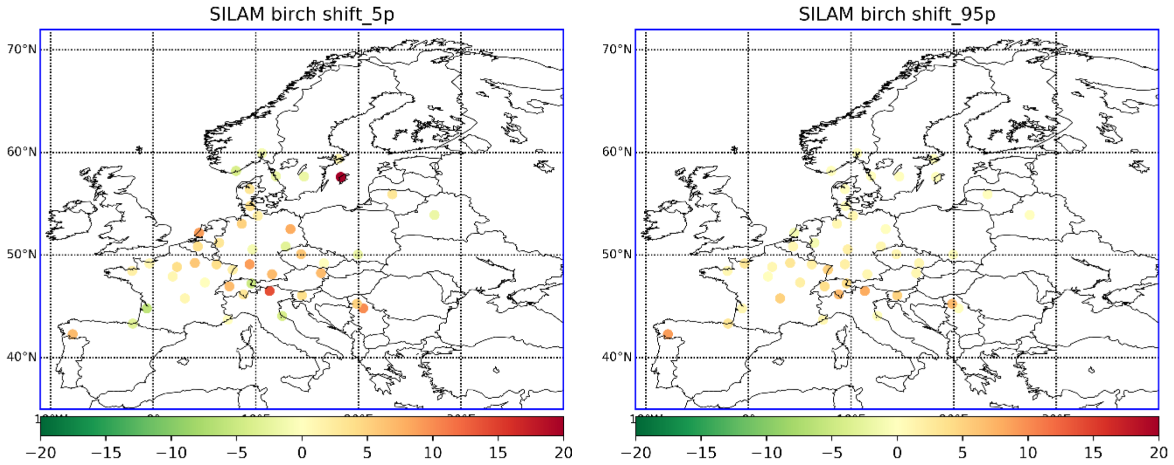

Figure 1.67 Quality of the season identification in 2020. Left-hand panel: season start error; right-hand panel: season end error. [day]. Observational data are provided by European Aeroallergen Network to CAMS for the model evaluation within the scope of CAMS-23 service contract.

Several studies reported local/regional trends in the pollen season timing,[110,111,112,113] but no long-term European-scale trends have been published this-far due to a lack of historical pollen observations. Comparing the current trend estimates with the published local assessments, there is a broad consensus in a tendency towards the earlier season in most of Europe. Specific trends vary between the stations and species, similar to patterns calculated for this indicator.

Acknowledgements
The work has been performed within the scope of Academy of Finland project PS4A, grant nbr 318194. The long-term simulations were supported by Academy of Finland project ALL-IMPRESS, grant 329215.

Section 2: Adaptation, planning, and resilience for health

2.1: Adaptation planning and assessment

The following section describes the methods, data, caveats, geographic coverage, and future forms of the indicators for three working group 2 indicators that are based on the WHO Health and Climate Change Global Survey.

Indicator 2.1.1 National assessments of climate change impacts, vulnerability and adaptation for health

Indicator 2.1.2 National adaptation plans for health

Indicator 2.2.1 Climate information for health

Methods

The global survey report is based on a triennial, voluntary survey sent to all 194 WHO Member States. It is completed by ministries of health in consultation with other health stakeholders, ministries and institutions.

The WHO health and climate change global survey is conducted every three years. The 2021 Global Survey was launched in March 2021 and final submissions were requested by August 2021.[114] A number of countries requested extensions due to the ongoing pandemic response and therefore additional submissions were received in September and October 2021.

The survey questionnaire was translated and available online in all six official UN languages.

Participation in the survey has grown substantially over the years. In 2015, 40 countries responded to the survey. This grew to 101 country respondents in 2018. Despite the COVID-19 pandemic and its demands on ministries of health, participation remained high in this third cycle (2021), with 95 country respondents.

Qualitative analysis of climate change and health V&A assessment documents.

This indicator draws on the 2021 WHO Health and Climate Country Survey.[114] It tracks the development of national health and climate change strategies, health impacts and vulnerability assessments, and the multi-stakeholder mechanism on health and climate change in place (table 2.2).

Geographic coverage of Europe

For this indicator, we include countries that completed the WHO survey. The 2021 survey was completed by 22 member states with representation from the WHO European Region out of the 53 member states. See table 2.1.

Table 2.1 The list of participating countries in 2021 global survey from the WHO European Region

List of participating countries and areas	Number of participating countries	Proportion of WHO region represented
Azerbaijan, Bulgaria, Croatia, Cyprus, Czechia, Estonia, Germany, Israel, Italy, Kazakhstan, Kyrgyzstan, Lithuania, Netherlands, North Macedonia, Poland, Portugal, Republic of Moldova, San Marino, Serbia, Slovakia, Sweden, Turkmenistan	22	42%

Source: 2021 WHO health and climate change global survey report.[114]

Table 2.2 Extracted data for the participating member states of the WHO European Region from the 2021 WHO health and climate change global survey report[114] on the development and implementation of national health and climate change strategies, health impacts and vulnerability assessments, multi-stakeholder mechanism on health and climate change, and focal points responsible for health and climate change within the Ministry of Health.

	**Countries reporting that climate change and health vulnerability and adaptation assessment(s) was conducting/ year**	**Countries reporting that the assessment results strongly influenced resource allocation**	**Countries reporting that the assessment results strongly informed health policy development**	**Countries reporting having a national health and climate change plan/strategy in place?/ YEAR**	**Countries reporting level of implementation of the national health and climate change plan (none; low, moderate, high, and very high); na (no info**	**Reporting having a multi-stakeholder mechanism on health and climate change that is currently operational**	**Reporting having a designated focal point responsible for health and climate change at the Ministry of Health.**
**Azerbaijan**	no	no	no	no	Unknown	no	No
**Bulgaria**	yes/2020	no	no	yes/2019	Unknown	Unknown	Unknown
**Croatia**	yes/2019	no	no	yes/2020	moderate	yes	yes
**Cyprus**	no	no	no	yes/2017	none	no	no
**Czechia**	yes/2017	no	no	yes/2015	low	yes	yes
**Estonia**	yes/2016	no	no	yes/2016	low	yes	no
**Germany**	yes/2021	yes	yes	yes/2020	Very high	yes	yes
**Israel**	Under development	no	no	yes/2018	Low	yes	yes
**Italy**	Under development	no	no	Under development	no	yes	yes
**Kazakhstan**	Unknown	no	no	Unknown	Unknown	Unknown	yes
**Kyrgyzstan**	Under development	no	no	Unknown	Unknown	yes	yes
**Lithuania**	yes/2014	no	no	yes/2012	Very high	yes	yes
**Netherlands**	yes/2014	no	no	yes/2016	Unknown	yes	yes
**North Macedonia**	yes/2010	no	yes	yes/2011	Very high	yes	yes
**Poland**	yes/2020	no	no	yes/2013	Unknown	no	no
**Portugal**	Under development	no	no	Under development	Unknown	yes	yes
**Republic of Moldova**	Unknown	no	no	Under development	no	no	no
**San Marino**	Under development	no	no	yes/2015	moderate	yes	yes
**Serbia**	no	no	no	no	no	no	yes
**Slovakia**	no	no	no	yes/2019	moderate	no	yes
**Sweden**	yes/2021	no	no	yes/2018	moderate	yes	yes
**Turkmenistan**	no	no	no	yes/2020	high	yes	no

Source: 2021 WHO health and climate change global survey report.[114]

Data

Validation of the 2021 country reported data was undertaken in multiple steps. First, survey responses were reviewed for missing information or inconsistencies with follow-up questions directed to survey respondents. A summary of responses was shared with WHO regional focal points and key informants for review, comments and validation. Source documents including national health strategies and plans, and climate change and health vulnerability and adaptation assessments were collected. A desktop review of these source documents was

conducted to compare with survey results with follow-up to survey respondents to seek clarification or additional documentation. Findings were also cross referenced with existing external publications. Data were collected detailing all the ministries, institutions and national stakeholders that provided contributions to or review of the survey responses in order to provide insight into the national consultation process of each survey submission. Finally, all respondents reviewed and acknowledged the WHO data policy statement on the use and sharing of data collected by WHO in Member States outside the context of public health emergencies.

Of note, due to the ongoing pandemic, the standard data collection procedures were modified to reduce the reporting burden on countries that wished to participate in the Global Survey but that were facing human resource constraints due to pandemic response. In eight cases, WHO prepared pre-filled survey questionnaires with data provided by ministries of health in the previous 2018 survey cycle or using data the countries had published in the 2020/2021 WHO UNFCCC health and climate change country profile when available. These countries were requested to review, revise, and complete the hard copy questionnaires. These hard copy questionnaires were then entered into the online platform by WHO. The same data validation steps as described above were then followed. Additionally, a number of countries requested an extension of the reporting period. As such, there may be a slight increase in the total number of participating countries in the WHO health and climate change global survey report after the time of the publication of the report and an online dynamic data dashboard will reflect any updated data and findings as required with specified version time and date.

Caveats

1. The global survey is conducted every three years, and not all the same countries participate every year.
2. The survey sample may not be a representative sample of all European countries as the survey is administered on a voluntary basis.

Future form of the indicator

1. For the next iteration of the indicator, self-reported data for adaptation plans, assessments, and climate information services can be disaggregated according to social determinants of health. This disaggregation will enable public health interventions to actively identify and support the populations most vulnerable to the effects of climate change. The WHO/UNFCCC Health and Climate Change Country Profiles, developed in collaboration with national health services, are data-driven snapshots of the climate hazards and the expected health impacts of climate change countries are facing. The data from the country profiles can me marge with the survey data to track current policy responses and summarize key priorities for climate and health action by country.
2. In 2022, WHO will publish at least 12 new country profiles for the WHO European Region, and more than half are countries that are not participating in the 2021 survey which was the base for this indicator. We hope that we will extend the number of countries for the indicator for 1/3 with information of countries that report having done a climate change, health vulnerability, and adaptation assessment or report having climate change and health plans based on the data from the countries' profiles.

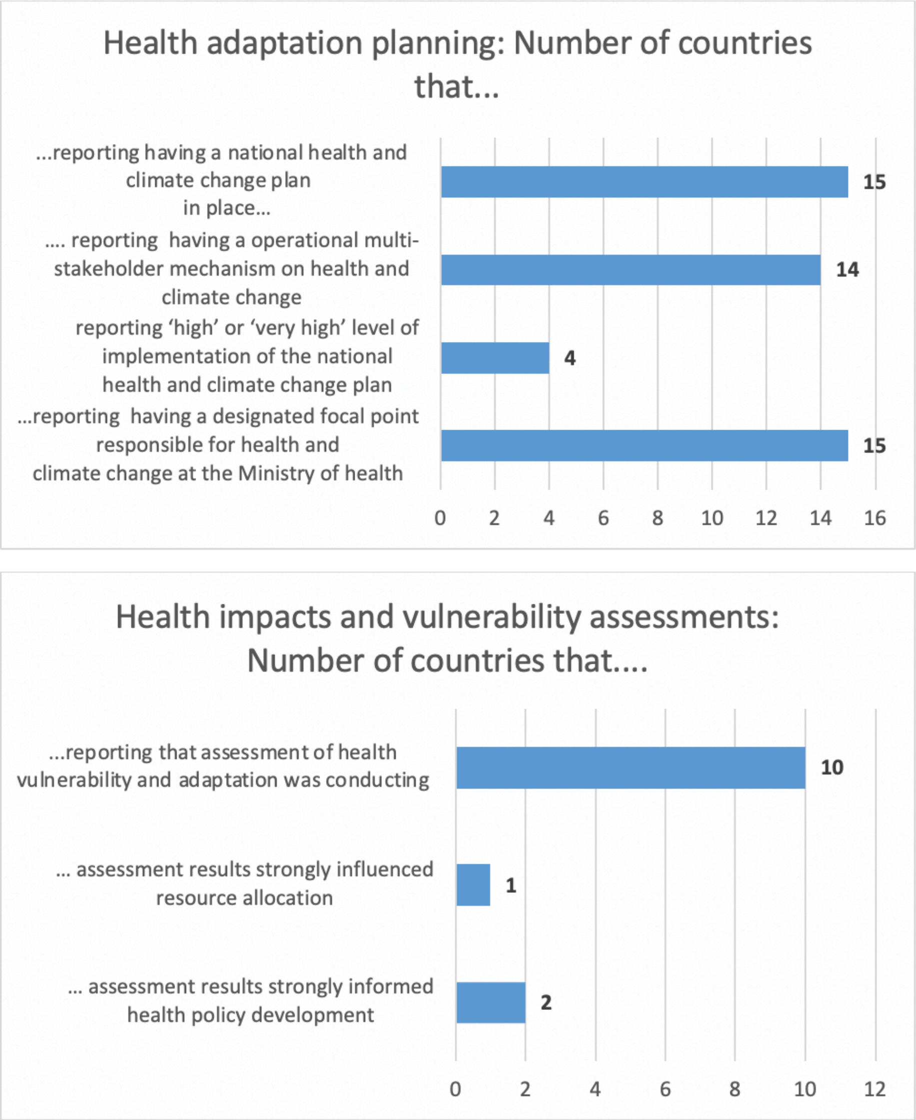

Figure 2.1 National adaptation planning and assessment in Europe.

Indicator 1.1.3 City-level climate change risks assessments

Methods

This indicator is based on the annual Carbon Disclosure Project (CPD) and International Council for Local Environmental Initiatives (ICLEI) Cities questionnaire. Data is collected through a CDP-ICLEI Unified Reporting System, in which cities can report their responses on questions regarding city-level climate change mitigation and adaptation. The dataset is updated daily to reflect new submissions, and is publicly accessible through: https://data.cdp.net/browse

The indicator is based on the following parts of the questionnaire.

- Has a climate change risk and vulnerability assessment been undertaken for your city?
- Is your city facing risks to public health or health systems associated with climate change?
    - Identify the climate-related health issues faced by your city.
    - Health-related risk and vulnerability assessment undertaken.
    - Please identify which vulnerable populations are affected by these climate-related impacts.

Data

1. 2021 CPD-ICLEI Annual Cities Survey

Geographic coverage of Europe

For this indicator we included cities in countries that are part of CPD Europe: Belgium, Bulgaria, Croatia, Denmark, Finland, France, Georgia, Germany, Gibraltar, Greece, Iceland, Ireland, Italy, Latvia, Lithuania, Monaco, Montenegro, Netherlands, Norway, Poland, Portugal, Romania, Russian Federation, Serbia, Slovenia, Spain, Sweden, Switzerland, Turkey, and the United Kingdom.

Caveats

- Participation of cities in this survey is on voluntary basis and self-reported. Hence data captured may be subjective to different forms of bias (i.e., response bias, social desirability bias), and not geographically representative of the entire European region.

Future form of the indicator

Firstly, for further iterations of the Lancet Countdown in Europe report, we hope to combine data from the Global Convent of Mayors for Climate and Energy, and the CPD-ICLEI Annual Cities Survey to capture information on a broader set of cities within Europe. Secondly, we are planning to perform additional analyses utilising the CPD annual survey to explore a broader set of indicators and monitor associations between city-level health vulnerabilities. Thirdly, in the next iteration of the Europe report we aim to track trends over time.

2.2: Adaptation delivery and implementation

Indicator 2.2.2: Exposure to green space

This indicator is divided into two sections 1) population weighted exposure to green space and 2) city level tree coverage.

Population weighted exposure to green space

Methods

NDVI quantifies photosynthetically active vegetation by measuring the difference between near-infrared (which vegetation strongly reflects) and red light (which vegetation absorbs). NDVI values range from +1.0 to -1.0. Areas of barren rock, sand, or snow usually show very low NDVI values (for example, 0.1 or less). Sparse vegetation such as shrubs and grasslands or senescing crops may result in moderate NDVI values (approximately 0.2 to 0.5). High NDVI values (approximately 0.6 to 0.9) correspond to dense vegetation such as that found in temperate and tropical forests or crops at their peak growth stage. Negative values of NDVI (values approaching -1) correspond mainly to water and ice and snow cover.

NDVI was derived from the Vegetation Indices (MOD13Q1) product of the Terra Moderate Resolution Imaging Spectroradiometer (MODIS) with 250 m x 250 m resolution.[115] MOD13Q1 16-day composite vegetation indices provide one NDVI value every 16 days allowing the composition of gap-free temporal-series. The per-pixel compositing algorithm is described in the MOD13Q1 product user guide.[116] The MOD13Q1 product includes the following relevant bands, among others: a) NDVI and b) quality assessment (QA) information. More information can be found here: https://lpdaac.usgs.gov/products/mod13q1v006/. Per-pixel Quality Assessment (QA) band provides information about how reliable the acquired data is and allows for removal of pixels with less accuracies. QA values of 0 and 1 mean a good quality level, whereas values of 2 and 3 represent pixels covered by ice, snow or clouds, which are discarded. Water features have typically negative signal values for NDVI, so they add noise to the negative edge when conducting spatial analysis. In order to identify and remove the influence of water bodies in the analysis, the Land Water Mask derived from MODIS and SRTM (MOD44W) was used to filter out the water bodies from the NDVI maps. This mask is a well-described satellite imagery with the same spatial resolution (250m) provided by the U.S. Geological Survey (USGS). MOD44W is a raster binary mask with land pixels represented as 0 and water as 1.

After the previous adjustments, median surrounding greenness was calculated using 5-year time points (2000, 2005, 2010, 2015 and 2020) for the entire Europe at 250m resolution.

Data

1. Population data for years 2006, 2011 and 2018 at 1km^2 resolution were based on data from Eurostat.[15]
2. Population data at 5-years age groups was at NUTS 3 level,[118] or when not available, at national level (country). A proportion/percentage for each 5-years age group was calculated for each NUTS 3 or country level and applied to each 1km^2 population grid cell, assuming a same age population structure within the NUTS 3 / country.
3. NDVI was derived from the Vegetation Indices (MOD13Q1) product of the Terra Moderate Resolution Imaging Spectroradiometer (MODIS) with 250 m x 250 m resolution [115]

4. Weighted-mean NDVI was computed at 1km^2 grid cell level using median NDVI value and the corresponding total population count and aggregated and country level.

Geographic coverage of Europe

Population-weighted exposure was calculated for all EEA countries (except Turkey), cooperating countries (Albania, Bosnia and Herzegovina, Kosovo, Montenegro, North Macedonia, Serbia) and United Kingdom.

For the missing countries in the population grid 2006 (Balkans) we used the 2011 grid datasets and the corresponding 2000 and 2005 NDVI data.

Caveats

NDVI is a fairly crude measure of green space and is context dependent. However, it is the greenspace exposure indicator for which there is the best evidence base linking green space to health.[119]

Future form of the indicator

Mortality attributable to lack of green space will be added to future versions of the indicator.

Additional analysis

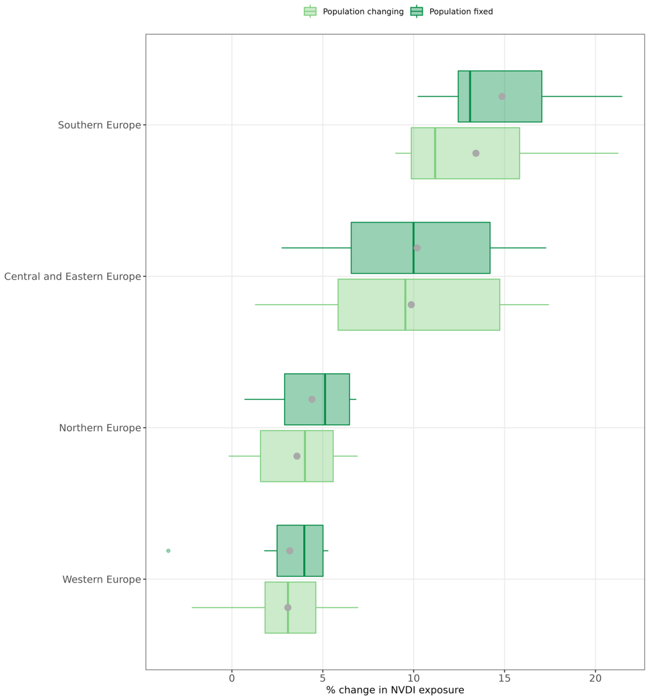

Figure 2.2 Change (%) of exposure to green space measured by the normalised difference vegetation index (NDVI) between 2020 and 2000 by European Region. Boxplots with median, interquartile range (IQR), maximum/minimum and outliers are presented. The mean by region is depicted in purple. % change is presented both when assuming a fixed population and when assuming changing populations.

City level tree coverage

Data

Heat islands are urbanized areas that experience higher temperatures than outlying areas. Structures such as buildings, roads, and other infrastructure absorb and re-emit the sun's heat more than natural landscapes such as forests and water bodies; urban development is therefore the cause of urban heat islands. Urban tree cover is the area in cities covered by tree crowns, if seen from above. Trees provide multiple benefits to the urban environment and the quality of life in cites. Amongst others, trees can help to adapt to the changing climate, by reducing air temperatures through shading and evapotranspiration, managing stormwater and reducing wind speeds. They can also act as a carbon sink, thus contributing to climate change mitigation efforts. Further, trees benefit mental and physical health, e.g., by lowering stress levels, reducing the amount of toxic particles in the ambient air or providing pleasant environment for cycling and walking. Lastly, trees – especially old, native ones - provide habitats for wildlife and increase biodiversity in urban areas. The importance of urban trees is recognised in the EU Biodiversity Strategy 2030, which requires that cities with over 20,000 inhabitants develop urban greening plans, including measures to create biodiverse and accessible urban green spaces, including parks, forests and tree-lined streets. In addition, the EU Forest Strategy, foreseen to be published in 2021, will include a roadmap for planting 3 billion new trees by 2030. The EU Strategy on Adaptation to Climate Change is strongly promoting nature-based solutions as "no regret" options for adaptation that simultaneously provide environmental, social and economic benefit.

The urban tree cover presented as part of this indicator includes trees on both public and private land and combines data from the following Copernicus Land Monitoring System products (all from 2018):

1. High-Resolution Layer Tree Cover Density (HRL TCD)[120]
2. Urban Atlas Land use/Land Cover data[121]
3. Urban Atlas Street Tree Layer[122]

Geographic coverage of Europe

For this indicator, we included the European Environment Agency (EEA) member and cooperating countries plus the United Kingdom.

Methods

The area and proportion of urban tree cover in cities is calculated for over 1000 cities in almost 800 urban areas in 38 countries and three different spatial reference units, i.e., city, the city's Commuting Zone and the Functional Urban Area (FUA), which is the combination of these two.

1. *City:* Densely populated area. Short definition: a city is a local administrative unit (LAU) where at least 50 % of the population lives in one or more urban centres.
2. *Commuting zone:* a commuting zone contains the surrounding travel-to-work areas of a city where at least 15 % of employed residents are working in a city.
3. *Functional urban area (FUA):* a city and its commuting zone. Short definition: a functional urban area consists of a city and its commuting zone. Functional urban areas therefore consist of a densely inhabited

city and a less densely populated commuting zone whose labour market is highly integrated with the city (OECD, 2012).

Caveats

Remotely sensed data as opposed to ground-level measurements.

Future form of the indicator

This indicator relies on various Copernicus products, which show the data from 2018 (among other Urban Atlas, which, whilst based on data from 2018, only became available in 2021). Thus, it is not clear when and update will be produced that can be compared to 2018.

Indicator 2.2.3: Air conditioning benefits and harms

This indicator is based on the data and methods of indicator 2.3.2 of the global *Lancet* Countdown 2021 Report.

Romanello, M., McGushin, A., Di Napoli, C., Drummond, P., Hughes, N., Jamart, L., ... & Hamilton, I. (2021). The 2021 report of the *Lancet* Countdown on health and climate change: code red for a healthy future. *The Lancet*, *398*(10311), 1619-1662.

A full description of the methods, data, caveats and future form of the indicator can be found in the Appendix of the global *Lancet* Countdown 2021 report: https://www.theLancet.com/cms/10.1016/S0140-6736(21)01787-6/attachment/ac088142-4570-4d4d-aeb8-d760254f9a1a/mmc5.pdf

Geographic coverage of Europe

1. For this indicator we include Italy, France, Germany, Finland, United Kingdom, Norway, Sweden, Denmark and Iceland as individual countries.
2. "Other Europe" is an International Energy Agency defined region including Albania, Austria, Belarus, Belgium, Bosnia and Herzegovina, Bulgaria, Croatia, Cyprus, Czech Republic, Estonia, Former Yugoslav Republic of Macedonia, Gibraltar, Greece, Holy See, Hungary, Ireland, Israel, Kosovo, Latvia, Lithuania, Luxembourg, Malta, Moldova, Monaco, Montenegro, Netherlands, Poland, Portugal, Romania, San Marino, Serbia, Slovak Republic, Slovenia, Spain, Switzerland, Turkey, Ukraine.

Analysis

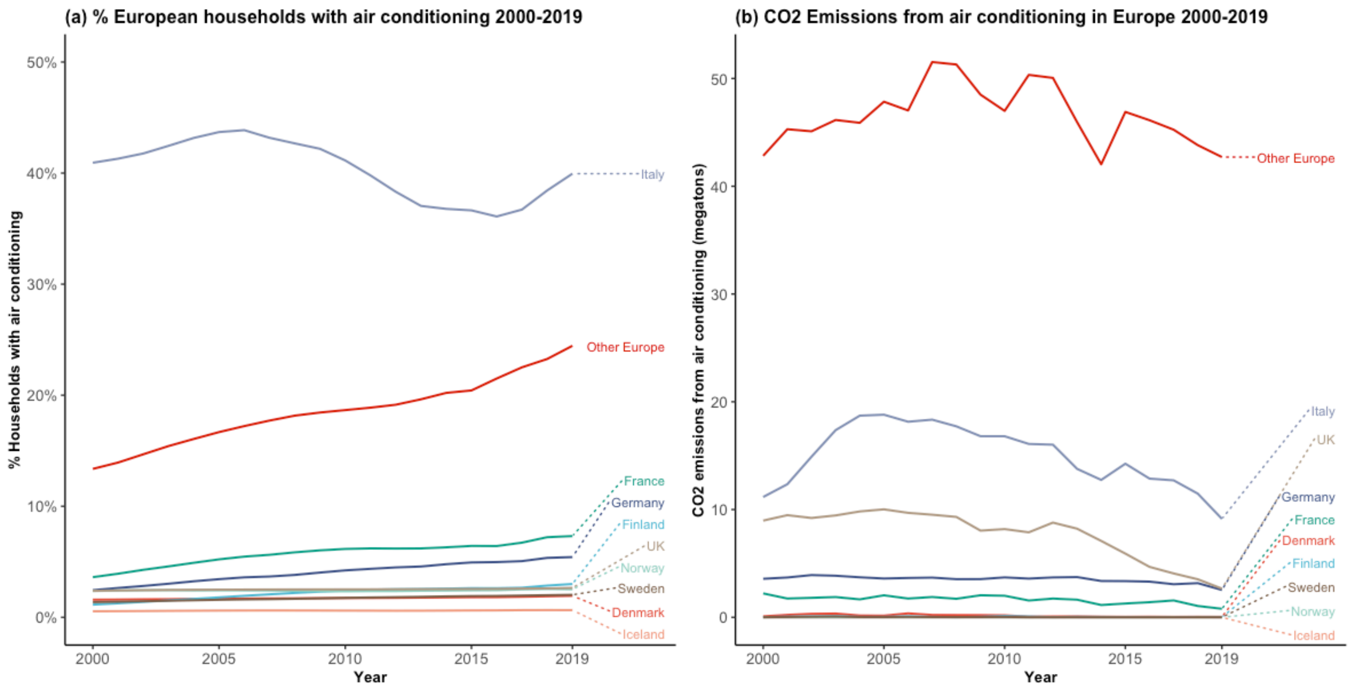

Figure 2.3 (a) Proportion of European households with air conditioning between 2000-2019. (b) CO_2 emissions from air conditioning in Europe over 2000-2019.

Section 3: Mitigation actions and health co-benefits

3.1: Energy system and health

This indicator outlines the fundamental drivers of the relationship between climate change and health, namely the use fossil fuels in the energy system. It comprises three sub-indicators.

Indicator 3.1.1: Carbon intensity of the energy system

Methods

This sub-indicator contains two metrics:

1. Carbon intensity of the energy system in Europe, (1990-2019), in tCO_2/TJ; and CO_2 emissions from energy combustion by fuel, in $GtCO_2$ (1990-2019).

2. Technical definition is the tonnes of CO_2 emitted for each unit (TJ) of primary energy supplied.

The rationale for the sub-indicator choice is that carbon intensity of the energy system will provide information on the level of fossil fuel use, which has associated air pollution impacts (explored in indicator 3.2). Higher intensity values indicate a more fossil fuel dominated system, and one that is likely to have a higher coal share. As countries pursue climate mitigation goals, the carbon intensity is likely to reduce, leading to a reduction in harmful air pollution emissions.

The indicator is calculated based on total CO_2 emissions from fossil fuel combustion divided by Total Energy Supply (TES). TES reflects the total amount of primary energy used in a specific country, accounting for the flow of energy imports and exports.

Furthermore, **figure 6 (main text)** includes a projection of the recent rate of reduction in carbon intensity, as well as the rate of reduction required to meet net-zero by 2050. The current rate of reduction of carbon intensity is incompatible with meeting the goals of the European Climate Law and the Paris agreement.

Countries with high overall emissions are highlighted in the figure. The remainder of countries are aggregated as "Rest of Europe". All countries in the EEA are given under the "All of Europe" region.

Geographic coverage of Europe

For this indicator, we included the European Environment Agency (EEA) member and cooperating countries plus the United Kingdom, for the period 1990-2019.

Data

This sub-indicator is based on the International Energy Agency (IEA) dataset, CO_2 Emissions From Fuel Combustion: CO_2 Indicators, accessed via the UK data service.[123]

Caveats

The sub-indicator does not provide information on the share of different fossil fuels, their use in different sectors, or the absolute levels of usage. These are all important elements in understanding air pollution emissions and their impacts. Additional sub-indicators (3.1.2 & 3.1.3) provide additional complimentary information.

Future form of the indicator

The data are updated annually by the IEA. There is currently no expectation that sub-national figures will become available.

Indicator 3.1.2: Coal phase-out

This sub-indicator is based on two metrics:

1. Total primary coal supply by country (in exajoules, EJ).
2. Share of electricity generation from coal (% of total generation from coal).

These metrics are important to enable tracking of changes in coal consumption at a regional and country level. As countries pursue climate mitigation goals, the use of coal is likely to reduce with resulting benefits for air pollution. The metric on primary energy coal supply is an aggregation of all coal types used across all sectors (from IEA energy balances). The data are available for all EEA member countries for the period 1990-2019.

The metric on the share of electricity generation from coal is estimated based on electricity generated from coal power plants as a percentage of total electricity generated.

Countries with high overall use of coal are highlighted in the figure. The remainder of countries are aggregated as “Rest of Europe”. All countries in the EEA are given under the “All of Europe” region.

Geographic coverage of Europe

For this indicator, we included the European Environment Agency (EEA) member and cooperating countries plus the United Kingdom

Data

This sub-indicator is based on the extended energy balances from the International Energy Agency. The specific dataset is called World Extended Energy Balances (for 2021), and is sourced via the UK data service.[124]

Caveats

As data collection methodologies across European countries are not harmonised, comparability between countries is limited. Differences between the collected modal data collected and further caveats are described here: https://ec.europa.eu/eurostat/cache/metadata/en/tran_hv_psmod_esms.htm[125]

Future form of the indicator

The data are updated annually by the IEA. There is currently no expectation that sub-national figures will be available.

Indicator 3.1.3: Renewable and zero-carbon emission energy

Methods

This sub-indicator comprises two metrics:

1. Total low carbon electricity generation, as a % share of total electricity generated (including nuclear, and all renewables); and
2. Total renewable generation (wind, solar PV and solar thermal, geothermal), as % share of total electricity generated.

The increase in the use of low carbon and renewable energy for electricity generation will push other fossil fuels, such as coal, out of the mix over time, resulting in improved air quality and associated health benefits.

The renewables indicator has been used to allow for the tracking of rapidly emergent renewable technologies. For both metrics, generation, rather than capacity, has been used as the electricity generated from these technologies is what actually displaces fossil-based generation.

Data are based on the IEA extended energy balances.[124] The absolute level data are total gross electricity generated aggregated from the relevant technology types. The share data are estimated as the low carbon or renewable generation as a % of total generation.

The data are available for most countries of the world, for the period 1971-2019. Only the period from 1990 has been used, due to data gaps for selected countries prior to 1990.

Geographic coverage of Europe

For this indicator, we included the European Environment Agency (EEA) member and cooperating countries plus the United Kingdom

Data

This sub-indicator is based on the extended energy balances from the IEA. The specific dataset is called World Extended Energy Balances, and is sourced via the UK data service.[124]

Caveats

This sub-indicator does not provide information on the air pollutant emissions displaced due to the increasing share of renewable energy generation.

Future form of the indicator

The data are updated annually by the IEA. There is currently no expectation that sub-national figures will become available.

Analysis

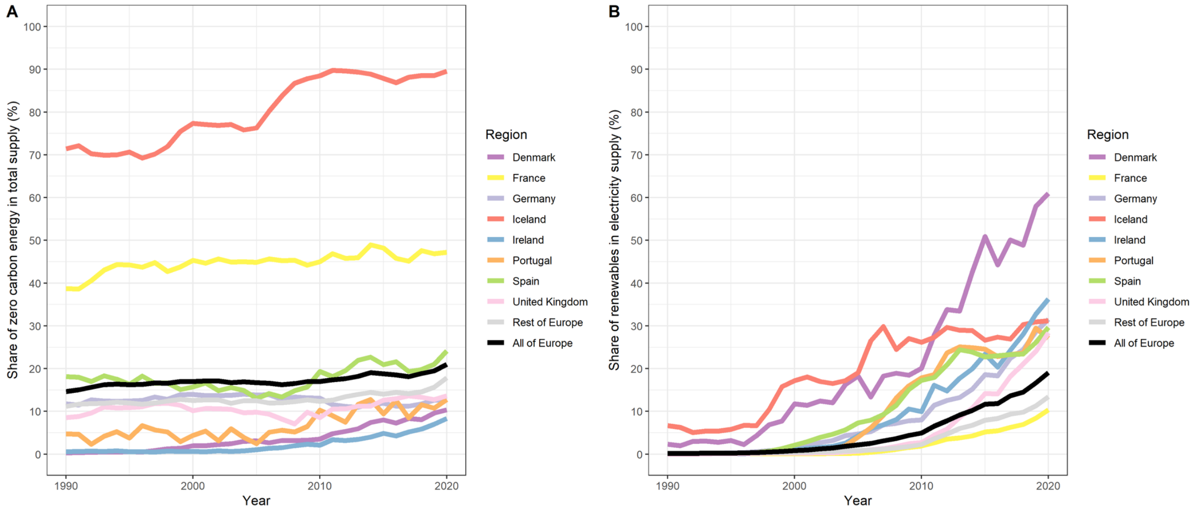

Figure 3.1 (A) Share of total energy supply provided by zero-carbon sources (renewables with the addition of hydroelectricity and nuclear) in Europe over 1990 to 2020. (B) Share of electricity supply provided by renewables (wind, solar, geothermal and tidal power) in Europe over 1990 to 2020. Zero-carbon energy sources are those which have effectively no carbon emissions associated with their production operations.

3.2: Premature mortality attributable to ambient fine particles

Methods

This indicator reports estimates of mortality attributable to long-term exposure to $PM_{2.5}$ originating from different fuels and sectors. It relies on calculations with the Greenhouse Gas-Air Pollution Interactions and Synergies (GAINS) model,[126] which combines bottom-up emission calculations with atmospheric chemistry and dispersion coefficients.

Data on energy consumption and other activities have been imported into GAINS from Eurostat data and mapped to detailed sectors in GAINS. They are then merged with GAINS internal information on application of emission control technologies in each country and year, reflecting the appropriate fleet/stock composition and legislation at that time. Each technology is associated with country-specific emission factors to calculate emissions of $PM_{2.5}$ and PM precursor gases SO_2, NOx, NH_3, and non-methane VOC.

Ambient $PM_{2.5}$ concentrations are calculated from the region and sector specific emissions by applying atmospheric transfer coefficients, which are a linear approximation of full chemistry-transport models. Calculations include both primary PM as well as secondary inorganic and organic aerosols. Atmospheric transfer coefficients in GAINS-Europe are based on full year perturbation simulations with the EMEP Chemistry Transport Model[127] run at a resolution of 0.5°×0.25° and include a downscaling for low-level emission sources of primary PM (residential emissions, road traffic and non-road machinery) to a resolution of 0.125°×0.0625° or roughly 7×7km based on a full-year simulation with the CHIMERE CTM.[128]

GAINS atmospheric calculations are described in detail by Kiesewetter *et al.* 2015.[129] For the *Lancet* Countdown indicator, contributions from individual (aggregated) source sectors such as road traffic, power plants, industry, and households, as well as from different fuel types (coal, liquids, gas, biomass, others) used in each sector, are kept separate in the calculation.

Attributable deaths are calculated through a comparative risk assessment framework. GAINS uses a linear approximation of the attributable fraction calculation,

$$AF_{csf} = \beta\ [PM]_{csf}$$

where AF_{csf} is the attributable fraction in country c from sector s and fuel f, $[PM]_{csf}$ is the population-weighted mean concentration of $PM_{2.5}$ in country c from emissions of sector s and fuel f, and β is the risk coefficient of the linearized concentration-response function. Here we use the concentration-response function reported by the systematic review by Chen and Hoek 2020[130] which found a relative risk for natural-cause mortality from long-term exposure to $PM_{2.5}$ of 1.08 per $10\mu gm^{-3}$, thus the coefficient $\beta = 0.008/\mu gm^{-3}$. Attributable deaths ad are calculated as

$$ad_{csf} = AF_{csf}\ nd_c$$

with nd_c the total natural-cause deaths over 30 years of age in country c.

Geographic coverage of Europe

For this indicator, we included the European Environment Agency (EEA) member and cooperating countries, excluding Liechtenstein, Bosnia and Herzegovina, Serbia, Kosovo and Montenegro as data was not available for these countries.

Data

1. Energy consumption by fuel and sector, industrial production: Eurostat,[131] IEA energy statistics[124] for countries missing in Eurostat
2. Other activities:
    a. Agricultural activities: Food and agriculture organization FAOSTAT[132]
    b. Fertilizer use: IFASTAT[133]
    c. Municipal waste, other minor sources: GAINS internal calculations
3. Mortality data: Eurostat. Data gaps for individual countries and years were filled with UN World Population Prospects 2017 estimates.

Caveats

1. The indicator relies on model calculations which are inherently uncertain and use linear approximations for atmospheric processes which are partly non-linear. See Amann et al (2012)[126] for a discussion of the linearity approximations.
2. Meteorological conditions vary from year to year and are one important factor for inter-annual variability of ambient $PM_{2.5}$ concentrations. The indicator does not consider this variability and reports only trends attributable to emission changes. Atmospheric coefficients in GAINS are based on CTM simulations for five different meteorological years (2006-2010) in order to represent a reasonable average of meteorological conditions.
3. Also, the spatial distribution of emissions is fixed to the base year
4. Concentration-response functions used in the attributable mortality calculation are uncertain. To minimize this, the analysis relies on the latest systematic review of epidemiologic data.

Future form of the indicator

Activity data are updated annually. In this report, five-year steps from 2005 onwards have been reported, but the time series can be extended annually. Currently the resolution of mortality results is by country (aggregated to European regions in the main text) but may be refined to sub-national level. The atmospheric coefficients in GAINS are currently undergoing an update which will improve the sectoral resolution. These will be available from next year.

Analysis

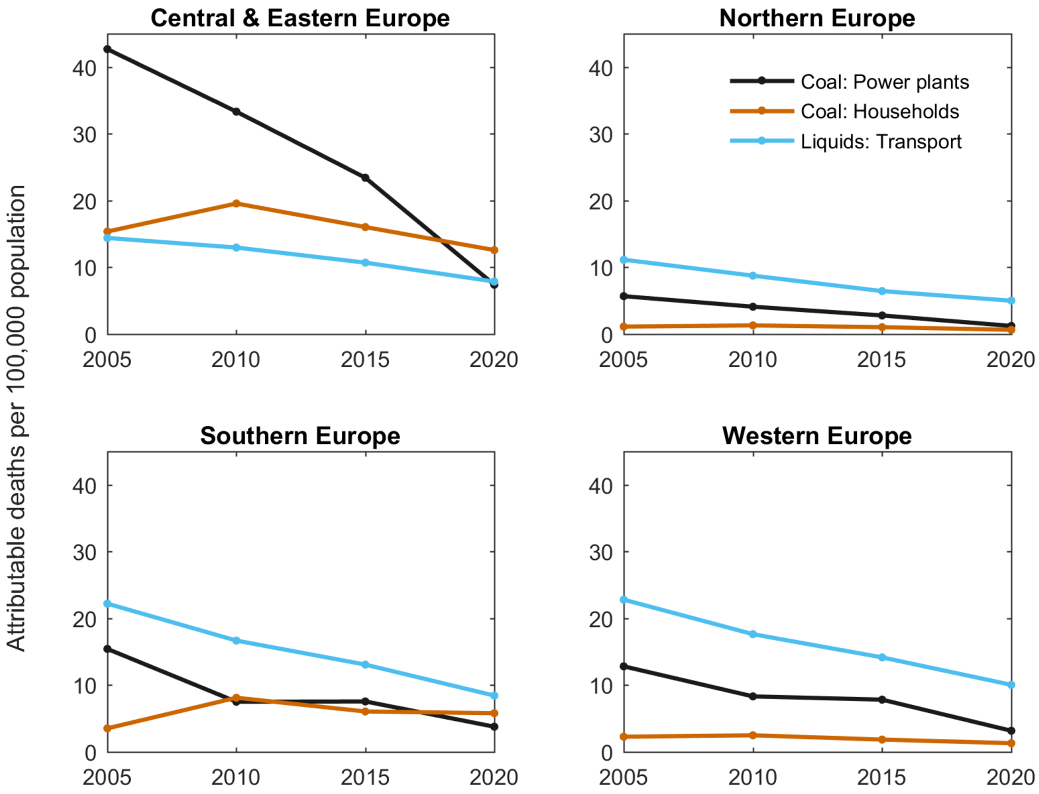

Figure 3.2 Premature mortality (annual attributable deaths per 100,000 population) due to $PM_{2.5}$ by fuel type, economic sector, and European region calculated in five-year steps. Fuel combustion in industry resulted in less than 5 deaths per100,000 population at each time point between 2005-2020.

Indicator 3.3: Sustainable and healthy transport

Methods

This indicator is comprised of two metrics:

1. Per capita fuel use on road transport data (by fuel type) from the IEA World Extended Energy Balances are divided by corresponding population statistics from the UNDP.
2. Mode share by country over time (2010 to 2019, 2019 shown in figure) by passenger-kilometres, provided by Eurostat

Geographic coverage of Europe

For this indicator, we included the European Environment Agency (EEA) member and cooperating countries plus the United Kingdom.

Data

1. Fuel use data is from the IEA, World Extended Energy Balances[124]
2. UN Population estimates, 2019 edition[134]
3. Modal split of passenger transport[125]

Caveats

Metric 1 of this indicator captures change in total fuel use and type of fuel use for transport, but it does not capture shifts in modes of transport used.

Metric 2 captures mode share for passenger vehicle trips but does not capture active travel such as walking and cycling for short trips, which can yield substantial health benefits through increased physical activity.

Further methodological information about how modal share is calculated is available here https://ec.europa.eu/eurostat/cache/metadata/en/tran_hv_psmod_esms.htm

Future form of the indicator

The data are updated annually by the IEA and Eurostat. There is currently no expectation that sub-national figures will be available, but the possibility of including regional (NUTS 2) survey data for modal-share exists.

Analysis

(a)

(b)

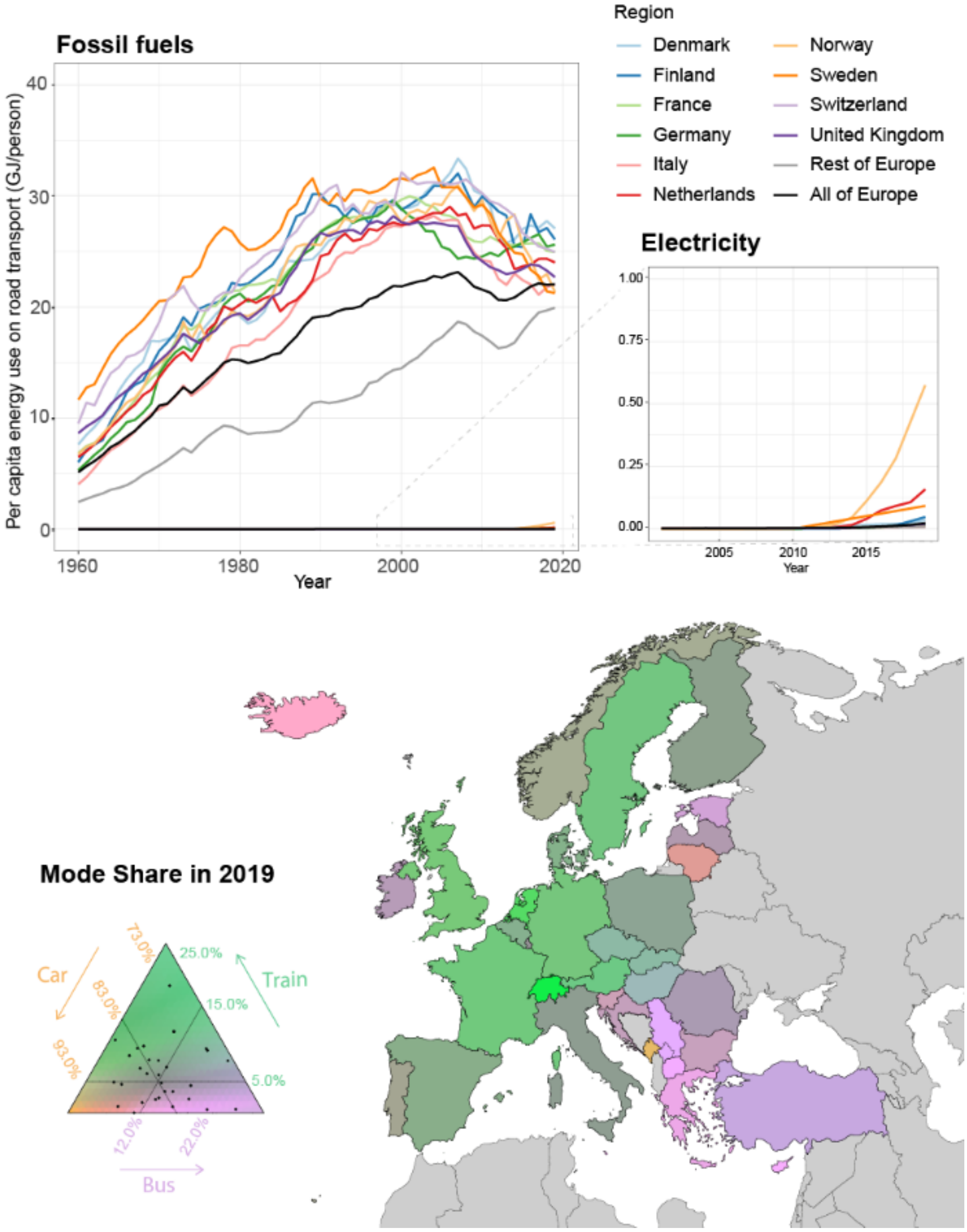

Figure 3.3 (a) Per capita fossil fuel use in road transport 1960-2019 in Europe (GJ/person). Inset: Electricity use in road transport 2000 – 2019 (GJ/person). (b) Mode share by European country in 2019. Colours correspond to percentage split between car, train and bus in passenger km. The central cross provides the Europe-wide median.

3.4: Food, agriculture and health

Indicator 3.4.1 Life cycle emissions from food demand

Methods

Evidence linking dietary changes to changes in environmental and health impacts is well established (e.g. Tilman and Clark, 2014[135]; Springmann et al, 2016[136]). Estimates from meta-analyses of life-cycle assessments for different food groups are typically combined with estimates of food consumption differentiated by food group and country. We used this approach to track the life-cycle emissions of food demand at an annual level.

Estimates of life-cycle emissions per food group and region were be paired with estimates of food demand by food group and country. Life-cycle estimates were based on regionalised data from a comprehensive meta-analyses,[137] and estimates of food demand were based on the Food Balance Sheets from the Food and Agriculture Organization (FAO) which are updated annually.

Geographic coverage of Europe

For this indicator, we used the M49 classification of Europe of the United Nations Statistical Division.

Data

Food demand from FAO's Food Balance Sheets, reported annually[132]

Life-cycle emissions from Poore and Nemecek (2018)[137]

Caveats

Life-cycle estimates are highly context-dependent. The analysis was based on a meta-analysis of life-cycle assessments to address this. However, large uncertainties for food groups and regions remain due to the limited availability of individual assessments. Improvements in farm management over time are not reflected in the estimates of life-cycle emissions, because they are not available over time in a consistent way.

Future form of the indicator

The estimates of life-cycle footprint will be updated as new data becomes available.

Indicator 3.4.2 Sustainable diets

Baseline consumption data

We estimated baseline food consumption by adopting estimates of food availability from the FAO's food balance sheets, and adjusting those for the amount of food wasted at the point of consumption.[138,139] We disaggregated this proxy for food consumption by age and sex by adopting the same age and sex-specific trends as observed in dietary surveys.[140]

An alternative would have been to rely on a set of consumption estimates that has been based on a variety of data sources, including dietary surveys, household budget and expenditure surveys, and food availability data.[141,142] However, neither the exact combination of these data sources, nor the estimation model used to derive the data have been made publicly available. For some individual countries, using dietary surveys would also have been an alternative. However, underreporting is a persistent problem in dietary survey,[143,144] and regional differences in survey methods would have meant that our results would not be comparable between countries. In contrast to dietary surveys, waste-adjusted food-availability estimates indicate levels of energy intake per region that reflect differences in the prevalence of overweight and obesity across regions.[145]

Food balance sheets report on the amount of food that is available for human consumption.[138] They reflect the quantities reaching the consumer, but do not include waste from both edible and inedible parts of the food commodity occurring in the household. As such, the amount of food actually consumed may be lower than the quantity shown in the food balance sheet depending on the degree of losses of edible food in the household, e.g., during storage, in preparation and cooking, as plate-waste, or quantities fed to domestic animals and pets, or thrown away.

We followed the waste-accounting methodology developed by the FAO to account for the amount of food wasted at the household level that was not accounted for in food availability estimates.[139] Table 3.1 provides and overview of the parameters used in the calculation.

For each commodity and region, we estimated food consumption by multiplying food availability data with conversion factors (*cf*) that represent the amount of edible food (e.g., after peeling) and with the percentage of food wasted during consumption (*1-wp(cns)*). For roots and tubers, fruits and vegetables, and fish and seafood, we also accounted for the differences in wastage between the proportion that is utilised fresh (pct_{frsh}) and the proportion that utilised in processed form (pct_{prcd}). The equation used for each food commodity and region was:

$$Consumption = Availability \cdot \frac{pct_{frsh}}{100} \cdot cf_{frsh} \cdot \left(1 - \frac{wp(cns_{frsh})}{100}\right) + Availability \cdot \frac{pct_{prcd}}{100} \cdot cf_{prcd} \cdot \left(1 - \frac{wp(cns_{prcd})}{100}\right)$$

Table 3.1 Percentage of food wasted during consumption (cns), and percentage of processed utilisation (pctprcd). The percentage of fresh utilisation is calculated as 1-pct_{prcd}. Conversion factors to edible portions of foods are provided below the table.

Food group	Item	Region						
		Europe	USA, Canada, Oceania	Indus-trialized Asia	Sub-Saharan Africa	North Africa, West and Central Asia	South and Southeast Asia	Latin America
cereals	wp(cns)	25	27	20	1	12	3	10
roots and tuber	pct_{prcd}	73	73	15	50	19	10	80
	wp(cns)	17	30	10	2	6	3	4
	wp(cns_{prcd})	12	12	12	1	3	5	2
oilseeds and pulses	cns	4	4	4	1	2	1	2
fruits and vegetables	pct_{prcd}	60	60	4	1	50	5	50
	wp(cns)	19	28	15	5	12	7	10
	wp(cns_{prcd})	15	10	8	1	1	1	1
milk and dairy	wp(cns)	7	15	5	0.1	2	1	4
eggs	wp(cns)	8	15	5	1	12	2	4
meat	wp(cns)	11	11	8	2	8	4	6
fish and seafood	pct_{prcd}	*40% for low-income countries, and 96% for all others.*						
	wp(cns)	11	33	8	2	4	2	4
	wp(cns_{prcd})	10	10	7	1	2	1	2

Conversion factors: maize, millet, sorghum: 0.69; wheat, rye, other grains: 0.78; rice: 1; roots: 0.74 (0.9 for industrial processing); nuts and seeds: 0.79; oils: 1; vegetables: 0.8 (0.75 for industrial processing); fruits: 0.8 (0.75 for industrial processing); beef: 0.715; lamb: 0.71; pork: 0.68; poultry: 0.71; other meat: 0.7; milk and dairy: 1; fish and seafood: 0.5; other crops: 0.78

Comparative risk assessment

We estimated the mortality and disease burden attributable to dietary and weight-related risk factors by calculating population impact fractions (PIFs) which represent the proportions of disease cases that would be avoided when the risk exposure was changed from a baseline situation to a counterfactual situation. For calculating PIFs, we used the general formula:[146,147,148]

$$PIF = \frac{\int RR(x)P(x)dx - \int RR(x)P'(x)dx}{\int RR(x)P(x)dx}$$

where $RR(x)$ is the relative risk of disease for risk factor level x, $P(x)$ is the number of people in the population with risk factor level x in the baseline scenario, and $P'(x)$ is the number of people in the population with risk factor level x in the counterfactual scenario. We assumed that changes in relative risks follow a dose-response relationship,[147] and that PIFs combine multiplicatively, i.e. $PIF = 1 - \prod_i (1 - PIF_i)$ where the i's denote independent risk factors.[147,149]

The number of avoided deaths due to the change in risk exposure of risk *i*, $\Delta deaths_i$, was calculated by multiplying the associated PIF by disease-specific death rates, *DR*, and by the number of people alive within a population, *P*:

$$\Delta deaths_i(r,s,a,d) = PIF_i(r,s,a,d) \cdot DR(r,s,a,d) \cdot P(r,s,a)$$

where PIFs are differentiated by region r, sex s, age group a, and disease/cause of death d; the death rates are differentiated by region, sex, age group, and disease; the population groups are differentiated by region, sex, and age group; and the change in the number of deaths is differentiated by region, sex, age group, and disease.

We used publicly available data sources to parameterize the comparative risk analysis. Mortality and population data were adopted from the Global Burden of Disease project.[150] Baseline data on the weight distribution in each country were adopted from a pooled analysis of population-based measurements undertaken by the NCD Risk Factor Collaboration.[145]

The relative risk estimates that relate the risk factors to the disease endpoints were adopted from meta-analyses of prospective cohort studies for dietary and weight-related risks.[151,152,153,154,155,156,157] In line with the meta-analyses, we included non-linear dose-response relationships for fruits, vegetables, and nuts and seeds, and assumed linear dose-response relationships for the remaining risk factors. As our analysis was primarily focused on mortality from chronic diseases, we focused on adults aged 20 year or older, and we adjusted the relative-risk estimates for attenuation with age based on a pooled analysis of cohort studies focussed on metabolic risk factors,[158] in line with other assessments.[148,159]

Table 3.2 provides an overview of the relative-risk parameters used. For the counterfactual scenario, we defined minimal risk exposure levels (TMRELs) as follows: 300 g/d for fruits, 500 g/d for vegetables, 100 g/d for legumes, 20 g/d for nuts and seeds, 0 g/d for red meat, and no underweight, overweight, or obesity. The TMRELs are in line with those defined by the Nutrition and Chronic Diseases Expert Group (NutriCoDE),[159] with the exception that we used a higher value for vegetables, and we used zero as minimal risk exposure for red meat, in each case based on a more comprehensive meta-analysis.[153,154]

The selection of risk-disease associations used in the health analysis was supported by available criteria used to judge the certainty of evidence, such as the Bradford-Hill criteria used by the Nutrition and Chronic Diseases Expert Group (NutriCoDE),[159] the World-Cancer-Research-Fund criteria used by the Global Burden of Disease project,[160] as well as NutriGrade (table 3.3).[161] The certainty of evidence supporting the associations of dietary risks and disease outcomes as used here were graded as moderate or high with NutriGrade,[154,155,156] and/or assessed as probable or convincing by the Nutrition and Chronic Diseases Expert Group,[159] and by the World Cancer Research.[162] The certainty of evidence grading in each case relates to the general relationship between a risk factor and a health outcome, and not to a specific relative-risk value.

We did not include all available risk-disease associations that were graded as having a moderate certainty of evidence and showed statistically significant results in the meta-analyses that included NutriGrade assessments.[154,155,156] That was because for some associations, such as for milk and fish, more detailed meta-analyses (with more sensitivity analyses) were available that indicated potential confounding with other major dietary risks or health status at baseline.[163,164,165] Such sensitivity analyses were not presented in the meta-analyses that included NutriGrade assessments, but they are important for health assessments that evaluate changes in multiple risk factors.

Table 3.2 Relative risk parameters (mean and low and high values of 95% confidence intervals) for dietary risks and weight-related risks.

Food group	Endpoint	Unit	RR mean	RR low	RR high	Reference
Red meat	CHD	100 g/d	1.15	1.08	1.23	Bechthold et al (2019)
	Stroke	100 g/d	1.12	1.06	1.17	Bechthold et al (2019)
	Colorectal cancer	100 g/d	1.12	1.06	1.19	Schwingshackl et al (2018)
	Type 2 diabetes	100 g/d	1.17	1.08	1.26	Schwingshackl et al (2017)
Fruits	CHD	100 g/d	0.95	0.92	0.99	Aune et al (2017)
	Stroke	100 g/d	0.77	0.70	0.84	Aune et al (2017)
	Cancer	100 g/d	0.94	0.91	0.97	Aune et al (2017)
Vegetables	CHD	100 g/d	0.84	0.80	0.88	Aune et al (2017)
	Cancer	100 g/d	0.93	0.91	0.95	Aune et al (2017)
Legumes	CHD	57 g/d	0.86	0.78	0.94	Afshin et al (2014)
Nuts	CHD	28 g/d	0.71	0.63	0.80	Aune et al (2016)
Underweight	CHD	15<BMI<18.5	1.17	1.09	1.24	Global BMI Collab (2016)
	Stroke	15<BMI<18.5	1.37	1.23	1.53	Global BMI Collab (2016)
	Cancer	15<BMI<18.5	1.10	1.05	1.16	Global BMI Collab (2016)
	Respiratory disease	15<BMI<18.5	2.73	2.31	3.23	Global BMI Collab (2016)
Overweight	CHD	25<BMI<30	1.34	1.32	1.35	Global BMI Collab (2016)
	Stroke	25<BMI<30	1.11	1.09	1.14	Global BMI Collab (2016)
	Cancer	25<BMI<30	1.10	1.09	1.12	Global BMI Collab (2016)
	Respiratory disease	25<BMI<30	0.90	0.87	0.94	Global BMI Collab (2016)
	Type 2 diabetes	25<BMI<30	1.88	1.56	2.11	Prosp Studies Collab (2009)
Obesity (grade 1)	CHD	30<BMI<35	2.02	1.91	2.13	Global BMI Collab (2016)
	Stroke	30<BMI<35	1.46	1.39	1.54	Global BMI Collab (2016)
	Cancer	30<BMI<35	1.31	1.28	1.34	Global BMI Collab (2016)
	Respiratory disease	30<BMI<35	1.16	1.08	1.24	Global BMI Collab (2016)
	Type 2 diabetes	30<BMI<35	3.53	2.43	4.45	Prosp Studies Collab (2009)
Obesity (grade 2)	CHD	30<BMI<35	2.81	2.63	3.01	Global BMI Collab (2016)
	Stroke	30<BMI<35	2.11	1.93	2.30	Global BMI Collab (2016)
	Cancer	30<BMI<35	1.57	1.50	1.63	Global BMI Collab (2016)
	Respiratory disease	30<BMI<35	1.79	1.60	1.99	Global BMI Collab (2016)
	Type 2 diabetes	30<BMI<35	6.64	3.80	9.39	Prosp Studies Collab (2009)
Obesity (grade 3)	CHD	30<BMI<35	3.81	3.47	4.17	Global BMI Collab (2016)
	Stroke	30<BMI<35	2.33	2.05	2.65	Global BMI Collab (2016)
	Cancer	30<BMI<35	1.96	1.83	2.09	Global BMI Collab (2016)
	Respiratory disease	30<BMI<35	2.85	2.43	3.34	Global BMI Collab (2016)
	Type 2 diabetes	30<BMI<35	12.49	5.92	19.82	Prosp Studies Collab (2009)

Table 3.3 Overview of existing ratings on the certainty of evidence for a statistically significant association between a risk factor and a disease endpoint. The ratings include those of the Nutrition and Chronic Diseases Expert Group (NutriCoDE),[159] the World Cancer Research Fund,[162] and NutriGrade.[154,155,156] The ratings relate to the risk-disease associations in general, and not to the specific relative-risk factor used for those associations in this analysis.

Food group	Endpoint	Association	Certainty of evidence
Fruits	CHD	reduction	NutriCoDE: probable or convincing;<br>NutriGrade: moderate quality of meta-evidence
	Stroke	reduction	NutriCoDE: probable or convincing<br>NutriGrade: moderate quality of meta-evidence
	Cancer	reduction	WCRF: strong evidence (probable) for some cancers<br>NutriGrade: moderate quality of meta-evidence for colorectal cancer
Vegetables	CHD	reduction	NutriCoDE: probable or convincing<br>NutriGrade: moderate quality of meta-evidence
	Cancer	reduction	WCRF: strong evidence (probable) for non-starchy vegetables and some cancers<br>NutriGrade: moderate quality of meta-evidence for colorectal cancer
Legumes	CHD	reduction	NutriCoDE: probable or convincing<br>NutriGrade: moderate quality of meta-evidence
Nuts and seeds	CHD	reduction	NutriCoDE: probable or convincing<br>NutriGrade: moderate quality of meta-evidence
Red meat	CHD	increase	NutriGrade: moderate quality of meta-evidence
	Stroke	increase	NutriGrade: moderate quality of meta-evidence
	Cancer	increase	WCRF: strong evidence (probable) for colorectal cancer<br>NutriGrade: moderate quality of meta-evidence for colorectal cancer
	Type-2 diabetes	increase	NutriCoDE: probable or convincing<br>NutriGrade: high quality of meta-evidence

NutriCoDE: Nutrition and Chronic Diseases Expert Group
NutriGrade: Grading of Recommendations Assessment, Dvelopment, and Evaluation (GRADE) tailored to nutrition research
WCRF: World Cancer Research Fund

For the different diet scenarios, we calculated uncertainty intervals associated with changes in mortality based on standard methods of error propagation and the confidence intervals of the relative risk parameters. For the error propagation, we approximated the error distribution of the relative risks by a normal distribution and used that side of deviations from the mean which was largest. This method leads to conservative and potentially larger uncertainty intervals as probabilistic methods, such as Monte Carlo sampling, but it has significant computational advantages, and is justified for the magnitude of errors dealt with here (<50%) (see e.g. IPCC Uncertainty Guidelines).

Geographic coverage of Europe

For this indicator, we used the M49 classification of Europe of the United Nations Statistical Division.

Data

Table 3.4. Overview of data sources

Type	Coverage	Source
Exposure data:		
Food consumption data	Country-level	Food availability data adjusted for food waste at the household level and for age and sex-specific trends.[138,139,140] Estimates of energy intake were in line with trends in body weight across countries.[145]
Weight estimates	Country-level	Baseline data from pooled analysis of measurement studies differentiated by sex and age with global coverage.[145]
Health analysis:		
Relative risk estimates	General	Adopted from meta-analysis of prospective cohort studies.[151,152,153,154,155,156,157] The certainty of evidence for the risk-disease associations were rated as moderate to high by NutriGrade.[154,155,156]
Mortality and population data	Country-level	Adopted from the Global Burden of Disease project by country, sex, and age group.[150]

Caveats

In the comparative risk assessment, we used relative risk factors that are subject to the caveats common in nutritional epidemiology, including small effect sizes and potential measurement error of dietary exposure, such as over and underreporting and infrequent assessment.[166] For our calculations, we assumed that the risk-disease relationships describe causal associations, an assumption supported by the existence of statistically significant dose-response relationships in meta-analyses, the existence of plausible biological pathways, and supporting evidence from experiments, e.g. on intermediate risk factors.[151,153,154,155,156,159,167,168,169,170] However, residual confounding with unaccounted risk factors cannot be ruled out in epidemiological studies. Additional aspects rarely considered in meta-analyses are the importance of substitution between food groups that are associated with risks, and the time lag between dietary exposure and disease.

To address potential confounding, we omitted risk-disease associations that became non-significant in fully adjusted models, in particular those related to milk intake,[163,164] and to fish intake.[165,171,172,173,174] The quality of evidence in meta-analyses that covered the same risk-disease associations as used here was graded with NutriGrade as moderate or high for all risk-disease pairs included in the analysis (table 5.1).[154,155,156] In addition, the Nutrition and Chronic Diseases Expert Group and the World Cancer Research Fund graded the evidence for a causal association of ten of the 12 risk-disease associations included in the analysis as probable or convincing.[159,162] The relative health ranking of leading risk factors found in our analysis was similar to existing rankings that relied on different relative-risk parameters and exposure data.[160,175]

As exposure data, we used a proxy of food consumption that was derived from estimates of food availability that were adjusted for the amount of food wasted at the point of consumption.[138,139] An alternative would have been to rely on a set of consumption estimates that has been based on a variety of data sources, including dietary surveys, household budget and expenditure surveys, and food availability data.[141,142] However, neither the exact combination of these data sources, nor the estimation model used to derive the data have been made publicly available. For some individual countries, using dietary surveys would also have been an alternative. However, underreporting is a persistent problem in dietary survey,[143,144] and regional differences in survey methods would have meant that our results would not be comparable between countries. In contrast to dietary surveys, waste-adjusted food-availability estimates indicate levels of energy intake per region that reflect differences in the prevalence of overweight and obesity across regions.[145]

Future form of the indicator

The estimates of diet-related disease burden will be updated annually based on new data on food intake, mortality, and population numbers.

Analysis

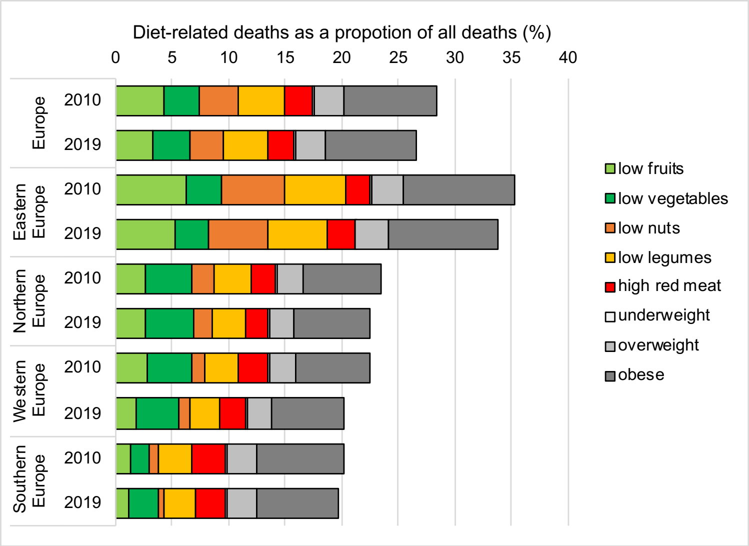

Figure 3.4 Burden of diet-related deaths in Europe by region and risk factor for 2010 and 2019.

Section 4: Economics and Finance

4.1: The health linked economic impacts of climate change and its mitigation

Indicator 4.1.1 Economic losses to climate-related extreme events

This indicator is based on the data and methods of indicator 4.1.1 of the global *Lancet* Countdown 2021 Report.

Romanello, M., McGushin, A., Di Napoli, C., Drummond, P., Hughes, N., Jamart, L., ... & Hamilton, I. (2021). The 2021 report of the *Lancet* Countdown on health and climate change: code red for a healthy future. *The Lancet*, *398*(10311), 1619-1662.

A full description of the methods, data, caveats and future form of the indicator can be found in the Appendix of the global *Lancet* Countdown 2021 report: [https://www.the*Lancet*.com/cms/10.1016/S0140-6736(21)01787-6/attachment/ac088142-4570-4d4d-aeb8-d760254f9a1a/mmc5.pdf](https://www.theLancet.com/cms/10.1016/S0140-6736(21)01787-6/attachment/ac088142-4570-4d4d-aeb8-d760254f9a1a/mmc5.pdf)

Geographic coverage of Europe

For this indicator, European Environment Agency (EEA) member and cooperating countries plus the United Kingdom were included.

Exchange rates

As data reported by Swiss Re is provided in USD, historical average market exchange rates per year for 2010-2021 (OECD) are used to convert USD in euros.

Analysis

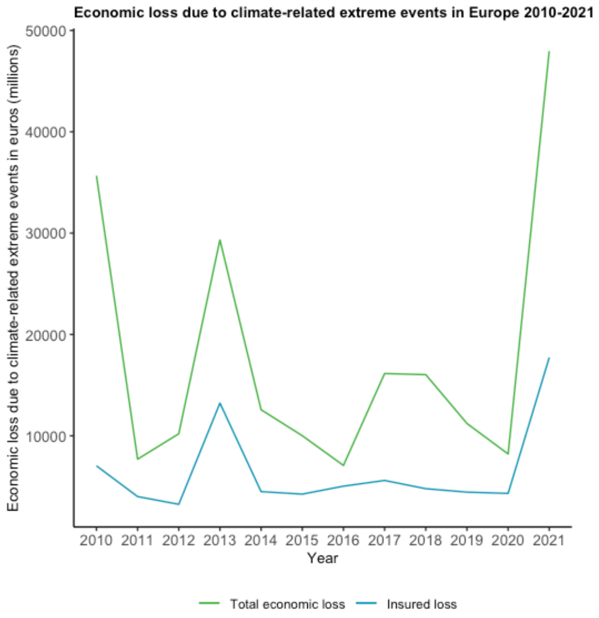

Figure 4.1 Total and insured economic loss in euros (millions) due to climate-related extreme events in Europe over 2010-2021.

Indicator 4.1.2 Change in labour supply

Methods

To track the impact of climate change on labour supply in Europe, we use a panel data fixed-effects regression. We control for mean temperature and its second-degree polynomial and total precipitation and its second-degree polynomial as climatic stressors (following Dasgupta *et al.*, 2021[176]). Our dependent variable is the log of the number of working hours at the NUTS 2 level. Our specification also controls for both NUTS 2 year fixed effects to account for unobserved heterogeneity such as changes in labour policies. The standard errors are clustered at the country-level. Our panel data specification can be written as follows:

$$\ln(y_{it}) = f_s(T_{it}) + \boldsymbol{X}\beta_{(it)} + \alpha_{(i)} + \gamma_{(t)} + \mu_{(it)}$$

where y_{it} is the log of number of hours worked at NUTS 2 region i at year t. $f_s(T_{it})$ represents the non-linear effect of sub-national temperature on labour supply, $\boldsymbol{X}\beta_{it}$ is a vector of precipitation terms and log of working population (between 15-64). $\alpha_{(i)}$ and $\gamma_{(t)}$ are NUTS 2 and year fixed effects to control for unobserved heterogeneity, while μ_{it} is a random error term. In the second step, we conduct a counterfactual analysis of the change in labour supply due to change in temperature from a long-term mean in Europe. We combine our econometric estimates with differences in periodic warming from a long-term mean of 1965-1994 to estimate the impact of temperature change on the number of hours worked in each NUTS-2 region. These data are presented as a percentage change in labour supply compared to 1965-1994.

Geographic coverage of Europe

For this indicator, we included all European Union member countries.

Data

1. Labour data: Eurostat Regional Database, 1995-2019. The original labour data comes from EU Labour Force Survey (EU-LFS). This is a quarterly household sample survey conducted in all Member States of the EU, the United Kingdom, EFTA, and candidate countries.
2. Climate data: ERA5-Land 0.1°×0.1 spatial and hourly temporal resolution.[13]

Caveats

The main caveat in this indicator is that the labour supply data is available only at the annual level, as such within year heterogeneity cannot be accounted for.

Future form of the indicator

Future iterations of this indicator will use micro-survey data from the EU Labour Force Survey (EU-LFS) and European Union Statistics on Income and Living Conditions (EU-SILC) to account for temporal heterogeneity and various household and societal characteristics. Second, impacts on labour supply and labour productivity will be estimated separately. Third, impacts of adaptation will be explicitly accounted for.

Indicator 4.1.3 Impact of heat on economic activity

Methods

To track the impact of climate change on economic activity in Europe, we use a panel data regression with coefficients that vary over time. Our measure of climate change is temperature anomaly, defined as the annual temperature difference, in °C, from a mean temperature of a 30-year period between 1981-2010. Our dependent variable is the real GDP per capita growth at the NUTS-2 level. Our specification also controls for drought measured by twelve-month Standardized Precipitation Index (SPI), and precipitation and its second-degree polynomial. To account for unobserved heterogeneity, we include both NUTS 2 year fixed effects. The standard errors are clustered at the country-by-year level. Our panel data specification can be written as follows:

$$y_{it} = \beta_1(\tau_t) + V_{(it)} + \gamma'(\tau_t)X_{(it)} + \alpha_{(i)} + \mu_{(it)}$$

where y_{it} is the real GPD per capita growth at NUTS-2 region *i* at year *t*, $\boldsymbol{V}_{it}$ is the temperature anomaly, and $\boldsymbol{X}_{it}$ is a vector of relevant variables affecting economic activity (droughts), while $\boldsymbol{\mu}_{it}$ is a random error term. All variables are recorded for different locations with index $i = 1, \dots, N$ and over a number of years $t = 1, \dots, T$. Our specification also includes location (NUTS 2) and time (year) fixed effects to control for unobserved heterogeneity and factors influencing GDP growth such as technological or policy changes from year-to-year and natural resources endowments. The time-varying coefficients allow us to examine whether the relationship between temperature anomaly economic activity has evolved over time.

Geographic coverage of Europe

1. South European countries: Albania, Austria, Belgium, Bulgaria, Croatia, Cyprus, Czech Republic, Germany, Greece, France, Hungary, Italy, Luxembourg, Malta, Netherlands, North Macedonia, Poland, Romania, Slovakia, Slovenia, Spain, and Turkey.
2. North European countries: Estonia, Latvia, Lithuania, Denmark, Finland, Iceland, Norway, and Sweden

Data

1. GDP growth data: Eurostat Regional Database, 2001-2019.
2. Climate data: ERA5-Land 9km^2 spatial and hourly temporal resolution.[13]

Caveats

The main caveat in this indicator is that the GDP growth data is available only at the annual level, as such temporal heterogeneity cannot be accounted for.

Future form of the indicator

Future iterations of this indicator will use micro-survey data from the European Union Statistics on Income and Living Conditions (EU-SILC) to account for temporal heterogeneity and various household and societal characteristics.

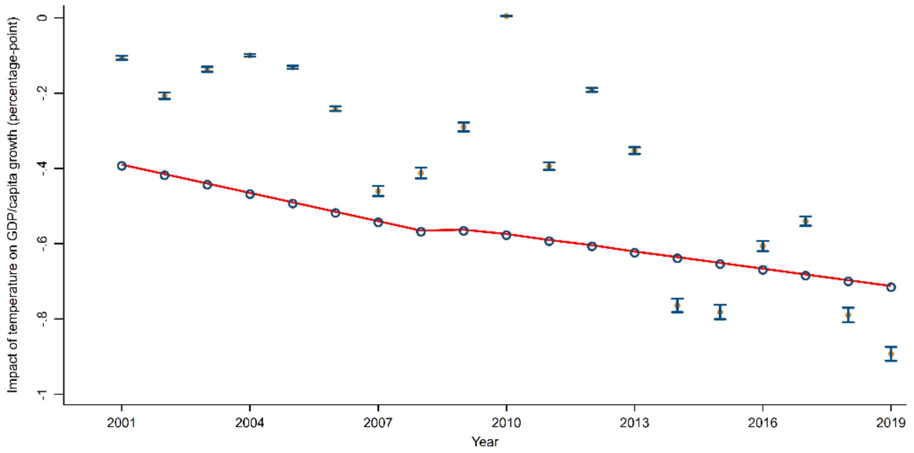

Figure 4.1 Reduction in real GDP per capita growth due to actual annual temperature anomaly from the reference period of 1981-2010 in Southern Europe. The yellow circles represent the change in GDP per capita for each year and the blue bars show the 95% confidence interval. The red line connects the regression coefficients for each year due to 1°C temperature anomaly. The estimates are generated from a time-varying coefficient regression using GDP per capita growth and ERA5-Land temperature data at the NUTS 2 level. The specification also includes NUTS 2 and year fixed effects. The standard errors are clustered at the country-by-year level.

Northern Europe results

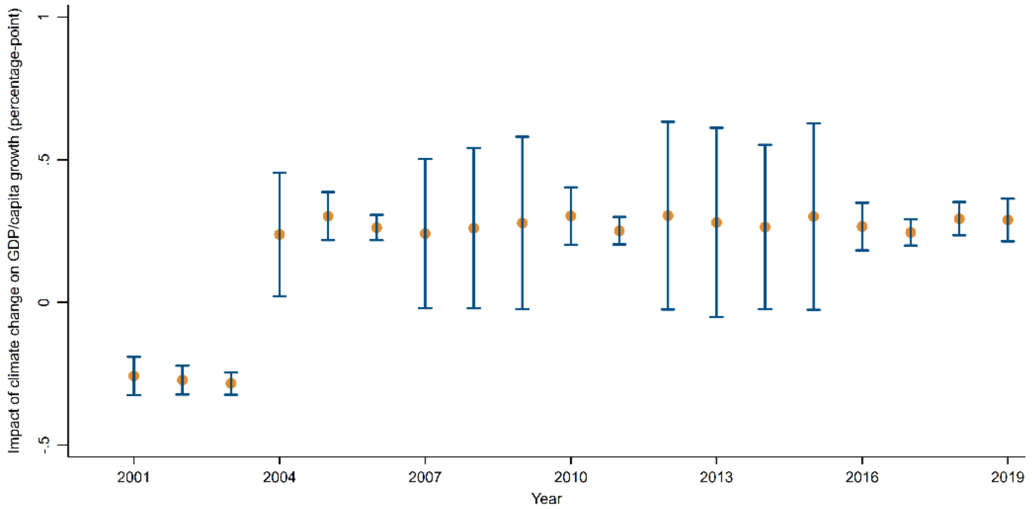

Figure 4.2 Reduction in real GDP per capita growth due to annual temperature anomaly from the reference period of 1981-2010 in Southern Europe. The yellow circles represent the coefficients for each year and the blue spikes show the 95% confidence interval. The red line shows the coefficients for each year. The estimates are generated from a time-varying coefficient regression using GDP per capita growth and ERA5-Land temperature data at the NUTS-2 level. The specification also includes NUTS-2 and year fixed-effects. The standard errors are clustered at the country-by-year level.

Indicator 4.1.4 Monetised value of unhealthy diets

Methods

Valuing the cost of unhealthy diets is relevant for tracking the health co-benefits of dietary changes towards more sustainable diets. According to model-based analyses, dietary changes towards more sustainable diets are associated with health benefits,[177] have a significant climate-change mitigation potential,[137] and they are needed as a part of a combination of mitigation measure to stay on track to limit global warming to less than 2 degrees Celsius.[178]

This indicator uses the estimates of health co-benefits of dietary change from the previous working group as a starting point, and combines those with standard methods to value the health impacts of dietary changes.[136] The most common method for valuing health impacts in monetary terms, and the one used here, makes use of the so-called value of statistical life, a measure based on the aggregate willingness to pay for health risk reduction.

To estimate the value of health co-benefits, estimates of the diet-related mortality (from WP2) are combined with estimates of the value of statistical life. The value of statistical life (VSL) is a measure for the willingness to pay for a mortality risk reduction defined as the marginal rate of substitution between money and mortality risk in a defined time period.[179] The VSL does not represent the value of life itself, but rather the value of small risks to life which can be estimated either from market decisions that reveal the implicit values reflected in behaviour (revealed preference studies), or by using surveys which elicit respondents' willingness to pay for small reductions in mortality risks directly (stated preference studies).

The VSL values are based on a comprehensive global meta-analysis of stated preference surveys of mortality risk valuation undertaken for the Organisation for Economic Co-operation and Development (OECD).[180] Following OECD recommendations, we adopt a VSL base value for the EU-27 of USD 3.5 million (1.75-5.25 million) and use the benefit-transfer method to calculate VSLs in other regions.[179] In the benefit-transfer method, the VSL base value is adjusted by income (Y) subject to an elasticity of substitution (β):

$$VSL_r = VSL_{base}\left(\frac{Y_r}{Y_{base}}\right)^{\beta}$$

Following OECD recommendations, we use GDP per capita adjusted for purchasing power parity (PPP) as a proxy for income, and we adopted an elasticity of 0.8 for benefit transfers to high-income countries and an elasticity of 1.0 for benefit transfers to low and middle-income countries.[179] Baseline data on GDP per capita were sourced from the World Bank Development Indicator database. In line with World Bank methodology, we defined the income classification of countries depending on their GDP per capita (adjusted for purchasing power parity).

Geographic coverage of Europe

For this indicator, we used the M49 classification of Europe of the United Nations Statistical Division.

Data

1. Values of statistical life: OECD[179]
2. Diet-related health impacts: estimates based on indicator 3.4.2

Caveats

There are uncertainties related to both the VSL estimates and the estimates of diet-related mortality. The former relates to the benefit transfer method that adjusts an aggregate value for differences in income levels, whilst the latter relates to epidemiological uncertainty related to the relative risk estimates.

Future form of the indicator

The estimates of the value of diet-related disease burden will be updated annually based on new data estimates of the burden of diet-related diseases (see indicator 3.4.2) and new economic data on national income.

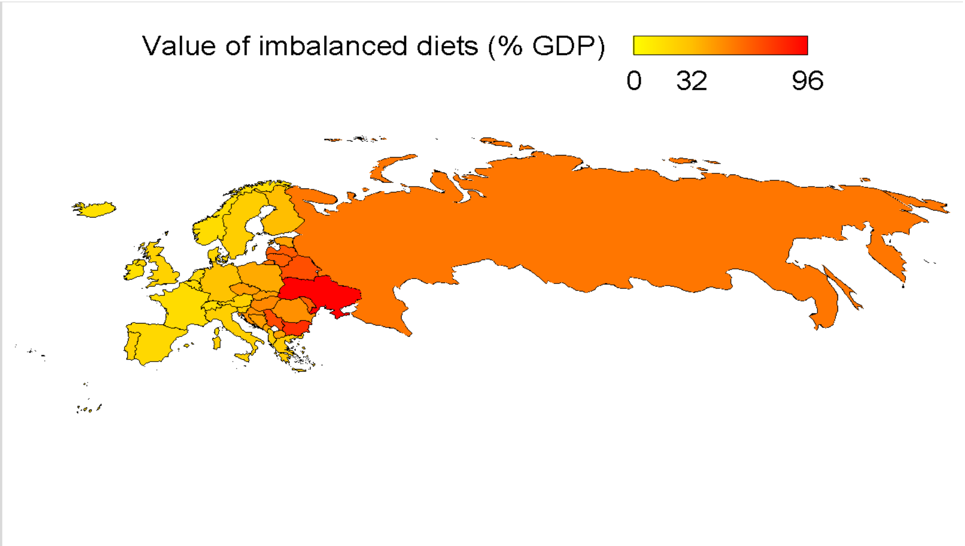

Figure 4.3 Map of the percentage (%) of imbalanced diets of the country's GDP by country in Europe.

4.2: The economics of the transition to zero-carbon economies

Indicator 4.2.1 Net value of fossil fuels subsidies and carbon prices

This indicator is based on the data and methods of indicator 4.2.4 of the global *Lancet* Countdown 2021 Report.

Romanello, M., McGushin, A., Di Napoli, C., Drummond, P., Hughes, N., Jamart, L., ... & Hamilton, I. (2021). The 2021 report of the *Lancet* Countdown on health and climate change: code red for a healthy future. *The Lancet*, *398*(10311), 1619-1662.

A full description of the methods, data, caveats and future form of the indicator can be found in the Appendix of the global *Lancet* Countdown 2021 report: https://www.the*Lancet*.com/cms/10.1016/S0140-6736(21)01787-6/attachment/ac088142-4570-4d4d-aeb8-d760254f9a1a/mmc5.pdf

Geographic coverage of Europe

For this indicator, we included the 53 member states of the WHO European Region.

Exchange rates

The International Monetary Fund (IMF) exchange rate of 0.8455 euros to the dollar (2021) is used to covert dollar into euros (https://data.imf.org/?sk=4c514d48-b6ba-49ed-8ab9-52b0c1a0179b).

Section 5: Public and Political Engagement

Indicator 5.1: Scientific engagement

Code and replication materials for Scientific engagement indicator (indicator 5.1) are available here: https://github.com/mcallaghan/scientific-engagement-climate-health/tree/main and for the other indicators (indicator 5.2 to 5.4) here: https://github.com/sjankin/*Lancet*_Europe.

Background

Scientific engagement in health and climate change is central to the *Lancet* Countdown mission: this is to facilitate, support and track progress on health and climate change. Scientific evidence is the major resource on which such progress rests; it also informs engagement in the key domains of global action, including the public, governmental and corporate domains. We quantify engagement in the topic of climate change and health by tracking the number of publications over time. Using a machine-learning assisted approach,[181] we identify relevant literature and classify it according to its subject.

Geographic coverage of Europe

For this indicator, we include the European Environment Agency (EEA) member and cooperating countries plus the United Kingdom.

Results

The growth of scientific publications investigating the intersection between health and climate change in Europe continued. Average annual growth in publications mentioning locations in Europe between 2000 and 2021 was 14%. In 2021, about 370 relevant articles were published, an increase of 9% compared to 2020. Authors affiliated with research organisations in Europe contributed to more than 800 publications on the climate and health nexus in 2021 - comprising 25% of all climate and health publications. The largest number of new publications studied locations in Italy, Spain, and Germany (66, 65 and 47 respectively, while the largest proportional increases compared to the average number of studies published over the preceding 5 years occurred in North Macedonia, Ireland and Denmark (53%, 43% and 43% increases respectively, albeit from low baselines). The majority of new papers concerned - in line with previous years - the health implications of climate change impacts (79%) of all relevant documents. Mitigation and adaptation accounted for 14% and 7% of new relevant documents respectively. However, over the last 10 years there is a pronounced increase in attention given to such research on climate solutions.

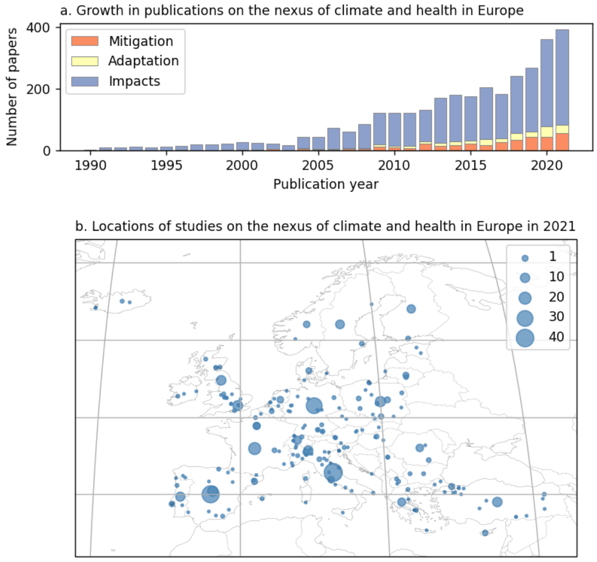

Figure 5.1 Trends in scientific publishing on health and climate change, and locations of the study focus in Europe (main text)

Annex

We replicate the analysis pipeline of a recent study of the literature on climate and health.[181] First, we repeat the search query - reproduced below - retrieving results from Scopus, Medline, and the Web of Science Core Collection. The aforementioned study screened 3,730 documents by hand, labelling whether relevant documents were related to

- The impacts of climate change on health;
- The effect on health of actions to mitigate climate change; or
- The effect on health of actions to adapt to climate change

Relevant documents were those which were indexed in English; provided a clear link to actual, projected or perceived impacts of climate change, responses to reduce the impacts of climate change (adaptation), or the mitigation of greenhouse gas emissions; and included substantial focus on a perceived, experienced, or observed eligible health-related outcome or health system; and presented empirically driven research or a review of such research.

A support vector machine (SVM) classifier[182] was trained to reproduce the inclusion/exclusion decisions as well as the impacts/mitigation/adaptation labels. Classifier performance was evaluated using 10-fold cross-validation. The inclusion/exclusion classifier achieved an accuracy of 87.1% with 80% recall and 76% precision.

Here we apply the same machine learning model to classify new studies which were not available when the original paper was produced. Those studies predicted to be irrelevant were discarded. Then we apply the multilabel impacts/mitigation/adaptation classifier to those documents which were included.

Finally, a neural network based "geoparser"[183] was applied to titles and abstracts of the texts to extract the geographical entities mentioned in the texts. These locations were allocated to countries, and then to continents. Country names were also extracted from the institutional affiliations recorded by the bibliographic databases.

Table 5.1 Search Query

Theme	Key concepts	String (Scopus)	Attributable Hits (scopus)
**Climate change** *(contains at least one of the following climate terms, from any category)*	General climate change terms	(climat* OR "global warming" OR "greenhouse effect*")	35,052
	Greenhouse gasses, including short-lived greenhouse gasses, when linked to emission or mitigation. Some astronomy results are filtered out.	(("carbon dioxide" OR co2 OR methane OR ch4 OR "nitrous oxide" OR n2o OR "nitric oxide" OR "nitrogen dioxide" OR nox OR *chlorofluorocarbon* OR *cfc* OR refrigerant OR hydrofluorocarbon* OR hfc* OR *chlorocarbon* OR "carbon tetrachloride" OR ccl4 OR halogen* OR ozone OR o3 OR ammonia OR nh3 OR "carbon monoxide" OR co OR "volatile organic compounds" OR nmvoc OR "hydroxyl radical" OR "oh" OR "pm2.5" OR aerosol OR "black carbon" OR "organic carbon" OR "sulphur dioxide" OR "oxidized sulphur" OR "so2" OR "sox" OR "sulphuric acid" OR so4*) W/2 (emit* OR emission OR releas* OR mitigat*) AND NOT(star OR "solar system"))	7,871
	Climate variability indicators/climate indices	(temperature* OR precipitat* OR rainfall OR "heat ind*" OR "extreme-heat event*" OR "heat-wave" OR "extreme-cold*" OR "cold ind*" OR humidity OR drought* OR hydroclim* OR monsoon OR "el ni$o" OR enso OR SOI OR "sea surface temperature*" OR sst)	199,558
	Complex climate indices, including extreme weather events, floods, wildfire, and coastal changes. Some paleo-climatic events are excluded.	(snowmelt* OR flood* OR storm* OR cyclone* OR hurricane* OR typhoon* OR "sea-level" OR wildfire* OR "wild-fire*" OR "forest-fire*" OR ((extreme W/1 event*) AND NOT paleo*) OR "coast* erosion" OR "coastal change*" OR (disaster* W/1 (risk OR manag* OR natural)))	22,031
**AND**	General health terms	(health* OR well?being OR ill OR illness OR disease* OR syndrome* OR infect* OR medical*)	49,773
**Health** *(contains at least one of the following health terms, from any category)*	General health outcomes	(mortality OR daly OR morbidity OR injur* OR death* OR hospital* OR {a&e} OR emergency OR emergencies OR doctor OR gp)	33,571
	Nutrition, including obesity and undernutrition	(obes* OR over?weight OR under?weight OR hunger OR stunting OR wasting OR undernourish* OR undernutrition OR anthropometr* OR malnutrition OR malnour* OR anemia OR anaemia OR "micronutrient*" OR "micro?nutrient*" OR diabet*)	2,239
	Cardio-vascular terms. Some studies on Chemical Vapour Deposition (CVD) are excluded.	(hypertension OR "blood pressure" OR stroke OR *vascular OR (cvd AND NOT(vapour or vapor)) OR "heart disease" OR isch?emic OR cardio?vascular OR "heart attack*" OR coronary OR chd)	6,047
	Renal health terms	(ckd OR renal OR cancer OR kidney OR lithogenes*)	4,934
	Effects of temperature extremes	((heat W/2 (stress OR fatigue OR burn* OR stroke OR exhaustion OR cramp*)) OR skin OR fever* OR renal* OR rash* OR eczema* OR "thermal stress" OR hypertherm* OR hypotherm*)	23,846
	Maternal health outcomes	(pre?term OR stillbirth OR birth?weight OR lbw OR maternal OR pregnan* OR gestation* OR *eclampsia OR sepsis OR oligohydramnios OR placenta* OR haemorrhage OR hemorrhage)	2,041

Vector-borne diseases	(malaria OR dengue* OR mosquito* OR chikungunya OR leishmaniasis OR encephalit* OR vector-borne OR pathogen OR zoonos* OR zika OR "west nile" OR onchocerciasis OR filiariasis OR lyme OR tick?borne)	2,257
Bacterial, parasitic and viral infections, including waterborne and foodborne diseases	(waterborne OR "water borne" OR diarrhoea* OR diarrhe*l OR gastro* OR enteric OR *bacteria* OR viral OR *virus* OR parasit* OR vibrio* OR cholera OR protozoa* OR salmonella OR giardia OR shigella OR campylobacter OR food?borne OR aflatoxin OR poison* OR ciguatera OR((snake* OR adder*) W/2 bite*))	46,064
Respiratory outcomes	(respiratory OR allerg* OR lung* OR asthma* OR bronchi* OR pulmonary* OR copd OR rhinitis OR wheez*)	3,432
Mental health outcomes	(mental OR depress* OR *stress* OR anxi* OR ptsd OR psycho* OR *trauma* OR suicide* OR solastalgi*)	12,616
Health systems	[no additional terms needed]	

Indicator 5.2: Individual engagement on social media

We used social media data extracted from the Twitter platform to explore the rhetoric about climate change and to understand the extent to which public audience in different countries across Europe mentions health and health issues in line with the climate change discussions.

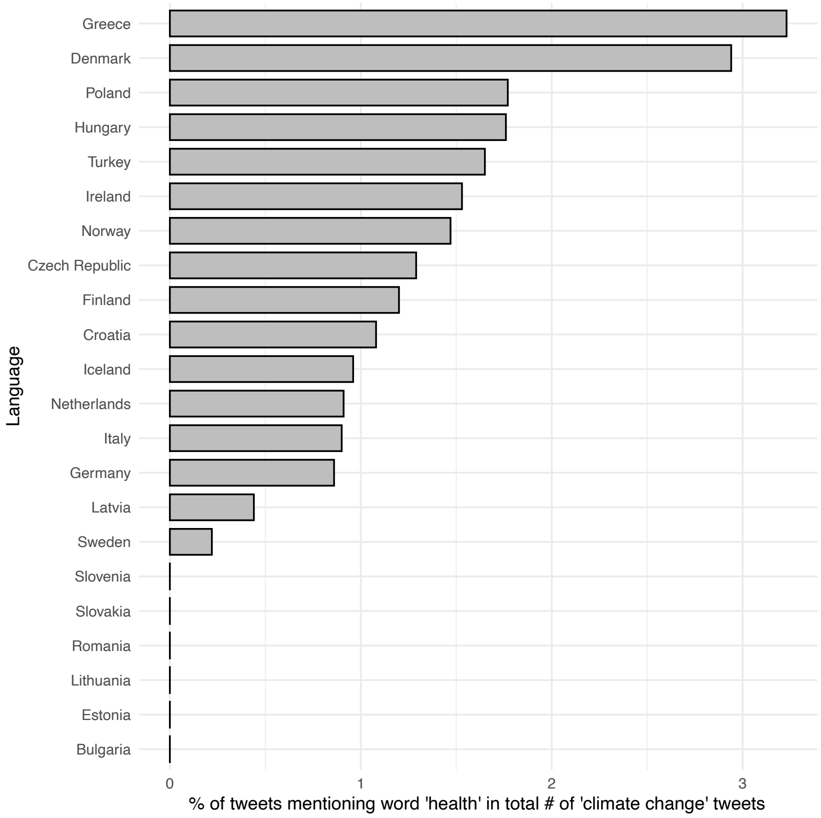

Figure 5.2 Percentage of tweets that mention "health" word in all the tweets that mention "climate change" in a given language.

Fundamentally, we are interested in public opinion about health attribution to climate change at the country level. To construct a first iteration of such an indicator, we used countries-members of the European Environment Agency. Due to the complications related to precise geolocation of individual tweets and users, we proxy it with the language on which the tweets are written. Hence, we used official languages of countries-members of the European Environment Agency to extract relevant country-specific tweets. There are evident measurement errors related to such approach. First, we excluded those languages that are widely used outside of the European continent, such as English, French, Spanish, and Portuguese. Second, some languages are spoken by several European countries (e.g., German language in Germany, Austria, and Switzerland, or Dutch language in Netherlands and Belgium), and here we guessed the country based on the population size speaking this language. Finally, users can migrate around the world and still use their native languages to tweet; or some portion of users in the analyzed countries can tweet in English to reach a wider audience. We aim to address and improve these measurement errors in the next iteration of the indicator.

For querying climate change related tweets, we used the direct translations of ‘climate change’ connotation to working languages. We queried all original tweets (including quotes) for the period from January 1, 2021 to December 31, 2021. Using raw string search, we selected the tweets that mention ‘health’ in the climate change tweets corpora for all the languages (figure 5.3-5.4 show the counts for all analyzed tweets split by months). Figure 5.2 indicates the percentage of tweets mentioning ‘health’. The results vary for different countries and in absolute numbers often do not exceed even 5 tweets, however the percentage overall varies from under 1% to about 3% (Table 5.3 in the appendix shows the exact percentages for each country). The supplementary materials provide the results for another health-related key word search - ‘disease’. For countries that returned a large amount of climate change and health associated tweets (Netherlands, Finland, Germany, Italy, and Turkey), we deployed a Twitter-Demographer model,[i] that allows to predict demographics of the twitter users who wrote the corresponding tweets. In the Twitter Demographer results, we observe that the majority of health-related tweets were written by males and by users older then 40 years old (see figure 5.6-5.10).

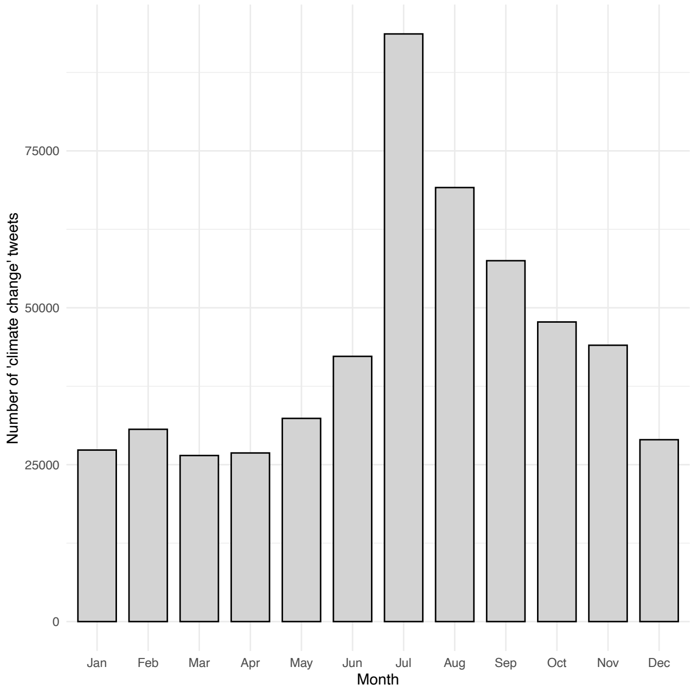

Figure 5.3 Total number of tweets that mention “climate change” across all the analyzed languages.

[i] We adapted the following code for Twitter-Demographer framework to our data: https://github.com/MilaNLProc/twitter-demographer (Last access on February 27, 2022).

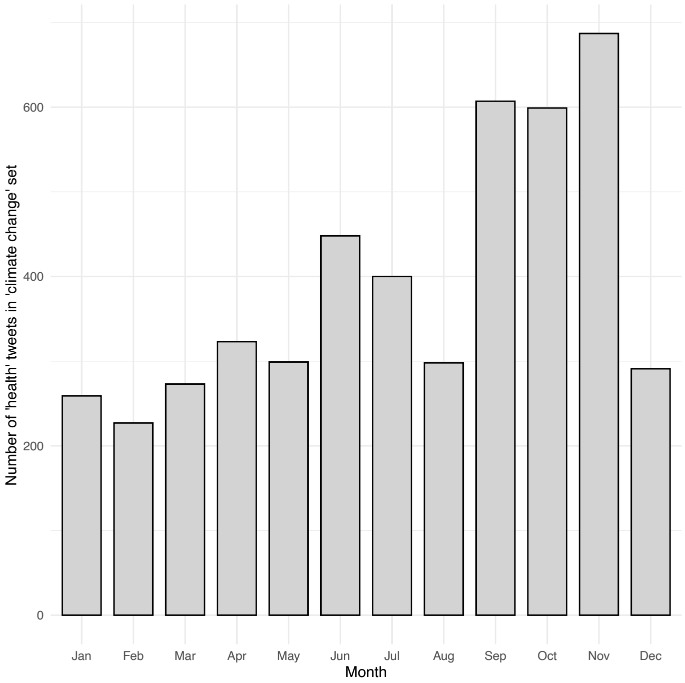

Figure 5.4 Total number of tweets that mention "health" in "climate change" across 22 analyzed languages.

Background

This indicator tracks the audience perception of the climate change impact on health by analyzing the discussion and rhetoric about it in the social media. The public has been recognizing the effects from climate change on health for already quite some time. The survey studies conducted in 2008-2010 in multiple countries indicated that a large proportion of respondents have seen themselves, their families, and their communities vulnerable to the harm from climate change.[184] Surveys can be helpful to understand the attitudes of the pubic, however they do not reveal the full discussion around climate change, mainly because a lot of respondents do not express their opinion in the open-ended questions or cannot connect climate change to any specific type of harm.[185] Hence, working with the social media data can bring more information. Additionally, social media data captures a larger cross-sectional time-series scope that allows us to track the public perception of climate change impact on health in dynamic and across different geographic territories.

Social media has become a wide-spread new source of information that allows the scientists and experts to reach a broader audience and allows the audience to be more engaged in the discussions about climate change, express their opinions about climate change and its impact on individual health. The existing studies show that only based on the Twitter data there can be tracked certain correlations between information about climate change and the audience sentiment,[186] also some recent analysis of the Twitter data shows that there exists an immediate response on the climate change regulations and discussions in public tweets.[187] That is another example of social media data relevance in understanding the audience perception of climate change consequences.

However, most of the existing studies that utilize Twitter data are concentrated on analyzing the sentiment around the climate change discussion. We contribute to this plethora of growing research by not just concentrating on the sentiment per se, but rather developing an indicator that measures to what extent the audience relates climate change to the existing or potential health problems. We utilize the Twitter data that discusses climate change in

the official languages of the countries-members of the European Environment Agency and analyze the extent to which these discussions mention health and health issues along the climate change mentions.

Geographic coverage of Europe

For this indicator, we include the core member countries of the European Environment Agency.

Methods and Data

We use the social media data extracted from the Twitter social media platform. To pull Twitter data, we use Twitter Developer API available for the Academic purposes.[ii] We query the exact translations of the 'climate change' connotation for 22 European languages, excluding languages that are wid ly-used beyond the European continent, such as English, French, Portuguese, and Spanish. We proxy the country-specific geolocation of tweets singularly based on the language, that is why it is quite hard to identify the location for world spread languages. There exist various models that adapt linguistic specifics, tweets meta-data and users' network to identify a more precise location of users and their tweets.[188,189,190] However, geolocation identification is a peculiar and computationally intensive process on its own. Hence, for this iteration of the indicator we decided to proxy the countries' location on the language itself and excluded languages that are commonly used in various territories outside of Europe. We will address and improve this measurement error in the further iterations of an indicator. We pulled the tweets according to the direct translation of the connotation ``climate change'' from English language to each of the corresponding languages. Table 5.2 shows the exact connotations that have been used to query tweets for each language. The timeframe of the pulled tweets is from January 1, 2021 to December 31, 2021. Querying included original tweets and quotes but excluded retweets. Retweets bring a lot of repetitions of the text corpora in the data. They also do not show the intentions to originally express opinions about climate change, but rather reflect the component of agreement. That is why we narrowed down the search only to original tweets and the quotes which add originality on the top of a retweet. All the promotional tweets written as part of Twitter advertisements were also excluded from querying.

The number of tweets varies severely depending on the proxied country (see table 5.2 for exact numbers of pulled tweets). There can be several possible explanations for that. First is related to the salience of climate change issues in different states. In the next iterations of this study, we aim to explore these predictions and try to identify what country-specific features can explain the salience of climate change topics in the public discussions. Secondly, it can be related to the fact that often when expressing opinions on Twitter platform, users prefer writing in English language, even when they are located outside of the English-speaking country, just to reach a wider audience. Hence, it is possible that a lot of tweets about climate change specifically ended up being in the pool of English-language tweets. Finally, Twitter querying does not allow to query wild cards (various forms of endings for words with the same root). At the same time a lot of languages have various grammatic forms for nouns and adjectives and verb conjugations, which might decrease the number of tweets pulled on the exact wordings. We plan to adjust and improve the querying procedure considering the aforementioned linguistical aspects for the next iterations of the current indicator.

[ii] Twitter Developer Portal: https://developer.twitter.com (Last access on February 27, 2022)

Table 5.2 Information on the pulled tweets for all the working languages.

Country	``climate change'' connotation translation	Number of tweets pulled
Bulgaria	изменението на климата	94
Croatia	klimatske promjene	1643
Czech Republic	klimatická změna	1446
Denmark	klima forandring	89
Estonia	kliimamuutus	68
Finland	ilmastonmuutos	38991
Germany	klimawandel	331603
Greece	την αλλαγή του κλίματος	134
Hungary	klímaváltozás	903
Iceland	loftslagsbreytingar	223
Ireland	athrú aeráide	279
Italy	cambiamento climatico	28977
Latvia	klimata izmaiņas	229
Lithuania	klimato kaita	57
Netherlands	Klimaatverandering	82291
Norway	klima forandringer	146
Poland	zmiana klimatu	2197
Romania	schimbarea climei	32
Slovakia	zmena podnebia	1
Slovenia	sprememba podnebja	1
Sweden	klimatförändring	899
Turkey	iklim değişikliği	36670

Table 5.3 Percentage of ‘climate change’ tweets that mention corresponding key words

Country	health	disease	illness
Bulgaria	NA	1.06	NA
Croatia	1.08	0.05	NA
Czech Republic	1.29	0.09	0.46
Denmark	2.94	NA	NA
Estonia	NA	NA	NA
Finland	1.2	0.03	NA
Germany	0.86	0.01	0.04
Greece	3.23	NA	NA
Hungary	1.76	0.59	NA
Iceland	0.96	NA	NA
Ireland	1.53	NA	NA
Italy	0.9	0.01	0.06

Latvia	0.44	NA	NA
Lithuania	NA	NA	NA
Netherlands	0.91	0.08	NA
Norway	1.47	NA	NA
Poland	1.77	0.44	NA
Romania	NA	3.85	NA
Slovakia	NA	NA	NA
Slovenia	NA	NA	NA
Sweden	0.22	0.22	NA
Turkey	1.65	0.92	NA

Empirical Analysis and Results

Key Words Search

The first part of the analysis includes key words search in each tweet and return of percentages of tweets that mention such key words. The search was based on the exact string search for every working language. The key words included five key words related to health issues: health, disease, illness. All words were searched in a singular infinitive form. Table 5.4 indicates the translations for each of the words for corresponding languages:

Table 5.4 Key Search Terms Translation.

Country	Language	health	disease	illness
Bulgaria	Bulgarian	здраве	заболяване	заболяване
Croatia	Croatian	zdravlje	bolest	bolest
Czech Republic	Czech	zdraví	chorob(a)	nemoc
Denmark	Danish	sundhed	sygdom	ildebefindende
Estonia	Estonian	tervist	haigus	haigus
Finland	Finnish	terveys	sairaus	ildebefindende
Germany	German	gesundheit	erkrankung	krankheit
Greece	Greek	υγεία	νόσος	ασθένεια
Hungary	Hungarian	egészség	betegség	betegség
Iceland	Icelandic	heilsu	sjúkdómur	veikindi
Ireland	Irish	sláinte	galar	tinneas
Italy	Italian	salute	patologia	malattia
Latvia	Latvian	veselība	slimība	slimība
Lithuania	Lithuanian	sveikata	liga	liga
Netherlands	Dutch	gezondheid	ziekte	ziekte
Norway	Norwegian	helse	sykdom	sykdom
Poland	Polish	zdrowi(e)	chorob(a)	chorob(a)
Romania	Romanian	sănătate	boala	maladie
Slovakia	Slovak	zdravie	choroba	choroba
Slovenia	Slovenian	zdravje	bolezen	bolezen

Sweden	Swedish	hälsa	sjukdom	sjukdom
Turkey	Turkish	sağlık	hastalık	hastalık

We estimated the percentages of tweets that mentions each of these words in the climate change tweets corpora. Mentions of the word 'health' in corresponding translations shape the core of an indicator. However, we also provide summary statistics for other health-related key words.

Figure 5.5 shows the percentages of mentions for word 'disease'. The word 'illness' was often translated in a similar way as 'disease' and did not return many results, hence we omitted a diagram for that term. Searching through these words can allow us to later estimate the extent to which an audience attributes various diseases to effects of climate change. Mentions of the word 'disease' (or often its synonym 'illness') occur not as consistently often across all the analyzed languages as mentions of the word 'health'.

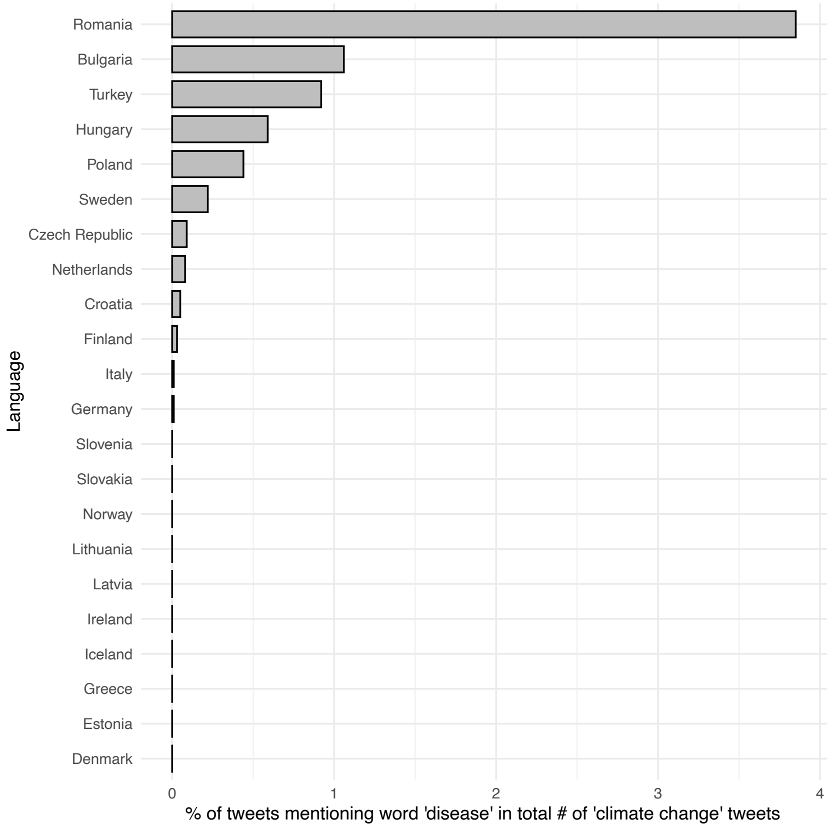

Figure 5.5 Percentage of tweets that mention "disease" word in all the tweets that mention "climate change" in a given language.

Twitter Demographer

We deploy a twitter-demographer model to the climate change tweets that mention 'health'. Twitter-Demographer model is a new framework introduced by Bianchi et al, 2022.[iii] We use their source code to adapt it to our Twitter

[iii] Twitter Demographer Library Description: https://twitter-demographer.readthedocs.io/en/latest/ (Last access February 27, 2022).

data. The model uses tweet IDs, reconstructs the tweet texts and predicts the demographics of users that published such tweets. Twitter Demographer uses the embedded in it M3 Inference system for predicting demographics.
To deploy Twitter Demographer, we only used those countries that returned a large number of tweets with ‘health’ mentions – Finland (460 tweets), Germany (2621 tweets), Italy (241 tweets), Netherlands (709 tweets), and Turkey (586 tweets). The overall results show that the majority of health-associated tweets come from males, and from the users in the age category of 40 years and older. More detailed results for each of the selected languages are shown in figure 5.6-5.10.

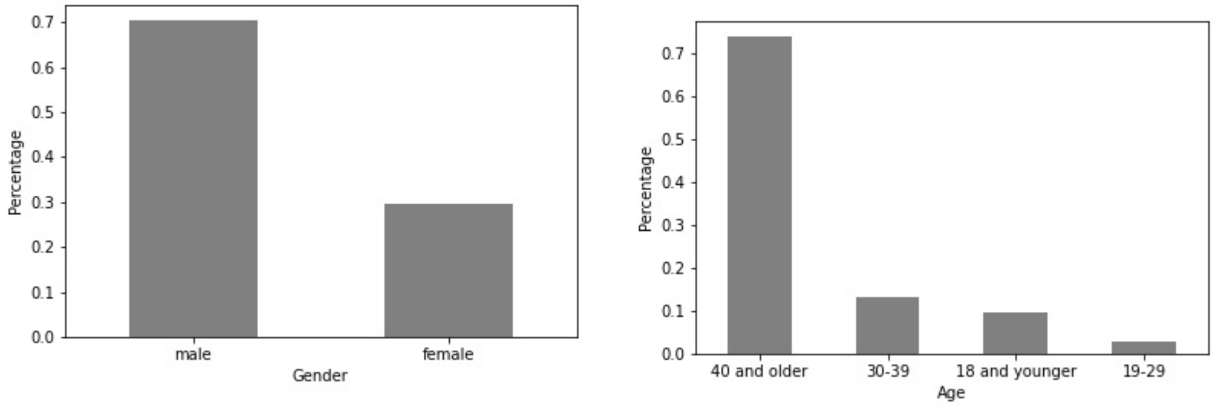

Figure 5.6 Demographic Distribution for tweets that mention ‘health’ in ‘climate change’ tweets in Finland.

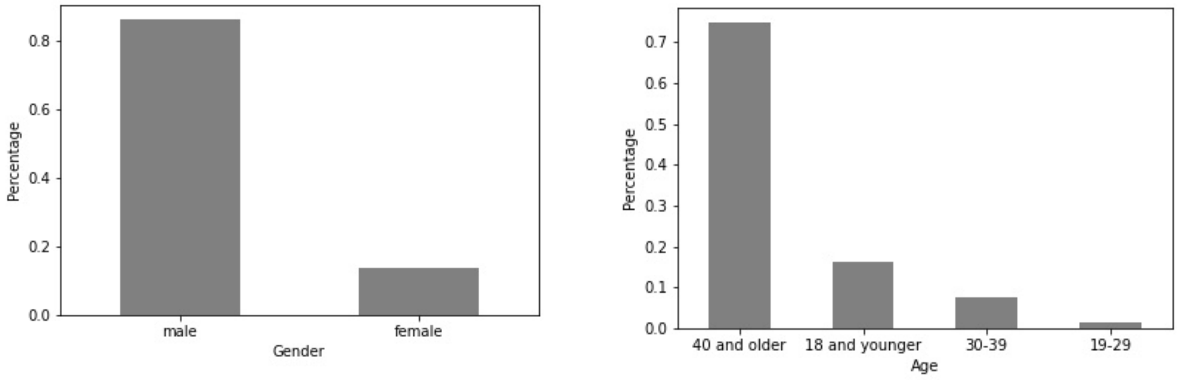

Figure 5.7 Demographic Distribution for tweets that mention ‘health’ in ‘climate change’ tweets in Germany.

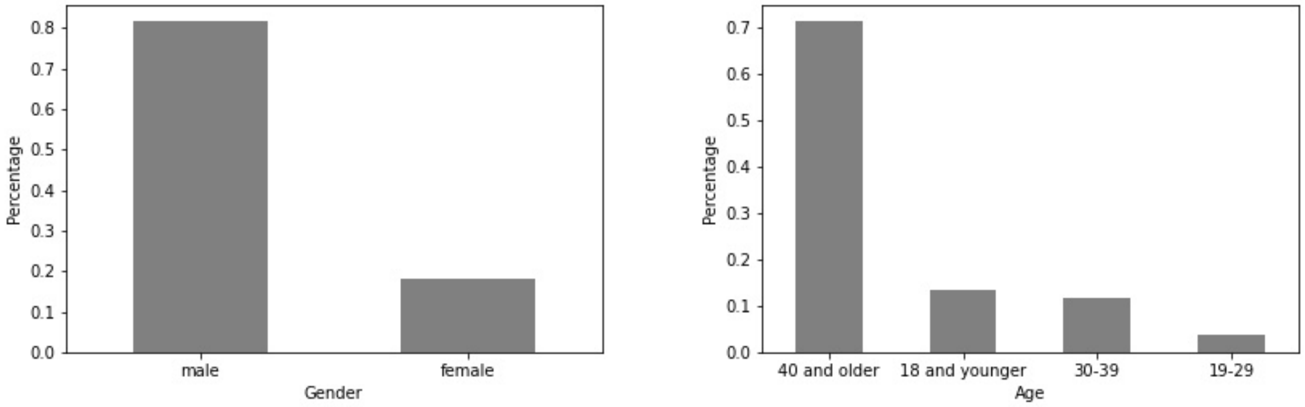

Figure 5.8 Demographic Distribution for tweets that mention 'health' in 'climate change' tweets in Italy.

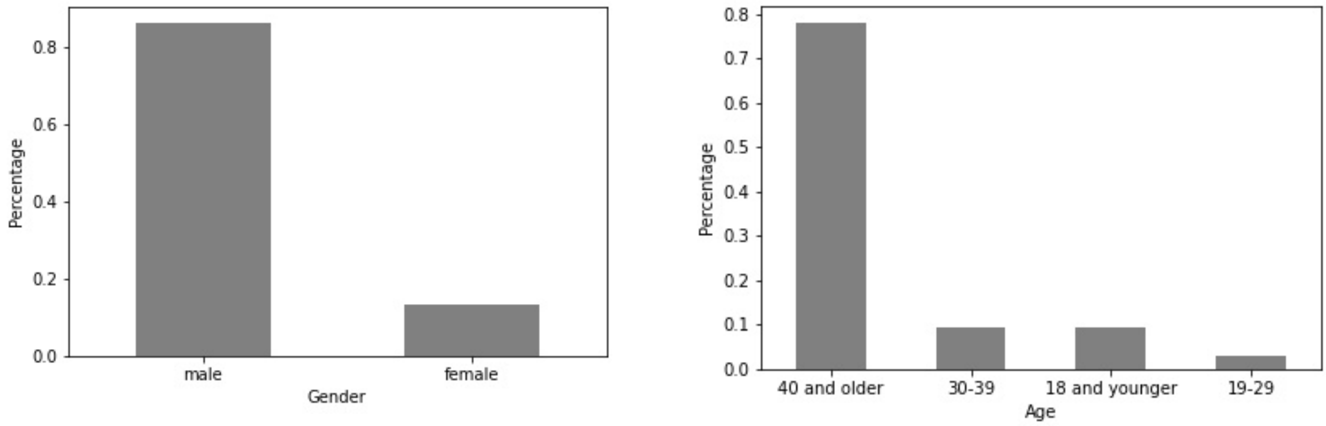

Figure 5.9 Demographic Distribution for tweets that mention 'health' in 'climate change' tweets in Netherlands.

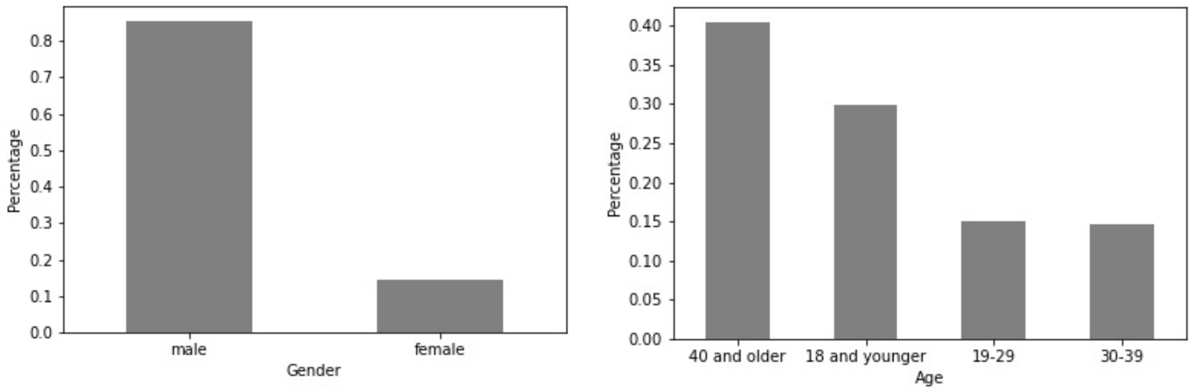

Figure 5.10 Demographic Distribution for tweets that mention 'health' in 'climate change' tweets in Turkey.

Predicted demographics shows that there is a smaller share of younger people or young adult writing about health when speaking about climate change; and that is consistent across several countries. However, these results only

indicate basic descriptive, and contain certain amount of selection bias. For instance, young adults might more likely to choose writing about climate change in English to be able to appeal to a broader Twitter audience. Additionally, Twitter API only allows us to pull tweets from currently existing and publicly open twitter accounts. It is reasonable to imagine that younger Twitter audience is more aware and more skilled in privacy issues, and prefers to express their opinions in the private social media accounts.

Further Work

The constructed indicator shows that public does actively talk about climate change and sometimes mentions health issues and health concerns in such conversations. To improve this indicator and to further explore the relations between climate change and health problems in the public rhetoric across various countries, we aim to develop several steps.

1. We plan to deploy a twitter geolocation model that will help us to more precisely identify the location of tweets. That way we would also be able to include tweets written in worldwide languages (English, French, Spanish, and Portuguese) but are written by users in European countries.
2. We plan to work more closely with the linguistics specifics of the analyzed languages (adjusting for various forms of nouns and adjectives, grammatical cases, and verb conjugations). That will allow us to improve querying of tweets and string search across the queried tweets corpora.
3. One of potential downsides of social media data is noise information produced by users-bots, promoted tweets, and duplicated tweets. One of the ways to address this issue is to additionally work with the queried tweets corpora. We will aim to exclude user-bots (for instance, using the Botometer project[iv]) and decrease meaningless tweet repetitions.
4. We plan to expand the set of health-related key words that we search for in the climate change tweets. That will help us to cover a wider dictionary of health terms that can be related to co-benefits angle.
5. Searching through key words potentially can underestimate the size of the health-related conversations, mainly because users can tweet about health issues without directly mentioning certain key words. Hence, to better estimate and understand the size of public rhetoric about health-related topics, we will deploy topic models. The novel set of topic models – contextualized topic models[v] use a pre-trained representations of language to support topic modeling.[191,192] We plan to adapt Zero-Shot Cross-Lingual Topic Model that helps to work with the multi-lingual component. The model uses one language tweets to identify topics and then with transfer learning predicts topics for each tweet in another language. That will allow us to predict topic prevalence in multiple languages without the direct translation of the text corpora.

Finally, to understand the sentiment behind health-related tweets and to explore to what extend public attributes health issues to the climate change or just simply speaks about climate change and health issues unrelated to each other, we will analyze the sentiment and causal meaning of the tweets. More specifically, we will use the constructed indicator to explore the sentiment of climate change social media discussions and will apply models of causality, examining causal attribution/causal reasoning in the analyzed text.

[iv] Official Webpage of the Botometer Project: https://botometer.osome.iu.edu (Last access on February 28, 2022).
[v] Source code is available here: https://github.com/MilaNLProc/contextualized-topic-models (Last access on February 28, 2022)

Indicator 5.3: Political engagement (European Parliament)

Methods

In order to produce the measure of engagement regarding climate change and health in debates of the European Union parliament, we used the publicly available transcripts of the debates. Our approach to produce the indicators is based on identifying the presence of key search terms related to health and climate change within each speech and identifying instances of intersection where terms relating to each field appeared close together, within a window of 25 words. We provide a full list of terms in table 5.5.

Table 5.5 A comprehensive list of terms on health and climate change

Health terms	Climate change terms
malaria	climate change
diarrhoea	changing climate
infection	climate emergency
disease	climate action
diseases	climate crisis
sars	climate decay
measles	global warming
pneumonia	green house
epidemic	temperature
epidemics	extreme weather
pandemic	global environmental
pandemics	change
epidemiology	climate variability
healthcare	greenhouse
health	greenhouse-gas
mortality	low carbon
morbidity	ghge
nutrition	ghges
illness	renewable energy
illnesses	carbon emission
ncd	carbon emissions
ncds	carbon dioxide
air pollution	carbon-dioxide
nutrition	co2 emission
malnutrition	co2 emissions
malnourishment	climate pollutant
mental disorder	climate pollutants
mental disorders	decarbonization
stunting	decarbonisation

	carbon neutral<br>carbon-neutral<br>carbon neutrality<br>climate neutrality<br>net-zero<br>net zero

Geographic coverage of Europe

For this indicator, we include the EU member states plus the United Kingdom.

Data

We downloaded the transcripts of the EU parliament debates for 2014-2022.[193] The data for 2019-22 was downloaded in XML format and data for 2014-19 was downloaded in JSON format, and then converted to CSV. There were a total of 413 debates containing 253,839 speeches in this dataset. Additionally, they were enhanced with metadata of the various speakers, taken from the database of MEPs available on the same website. Since a lot of the speakers for 2014-18 do not have data in the MEP database, however, much of the speeches do not have this meta-data. We pre-processed and prepared these documents for the application of natural language processing by converting the reports to plain text format as well as translating non-English documents to English texts. We used google_trans_new package,[194] a python API for automatic translation. The package is free to use for academic purposes and uses state-of-the-art neural machine translation developed at google. Further preprocessing involved the removal of stopwords and regularising (lowercasing) and was performed in Python with the NLTK package.

Engagement with Health, Climate Change, and the Intersection of Health and Climate Change in 2021

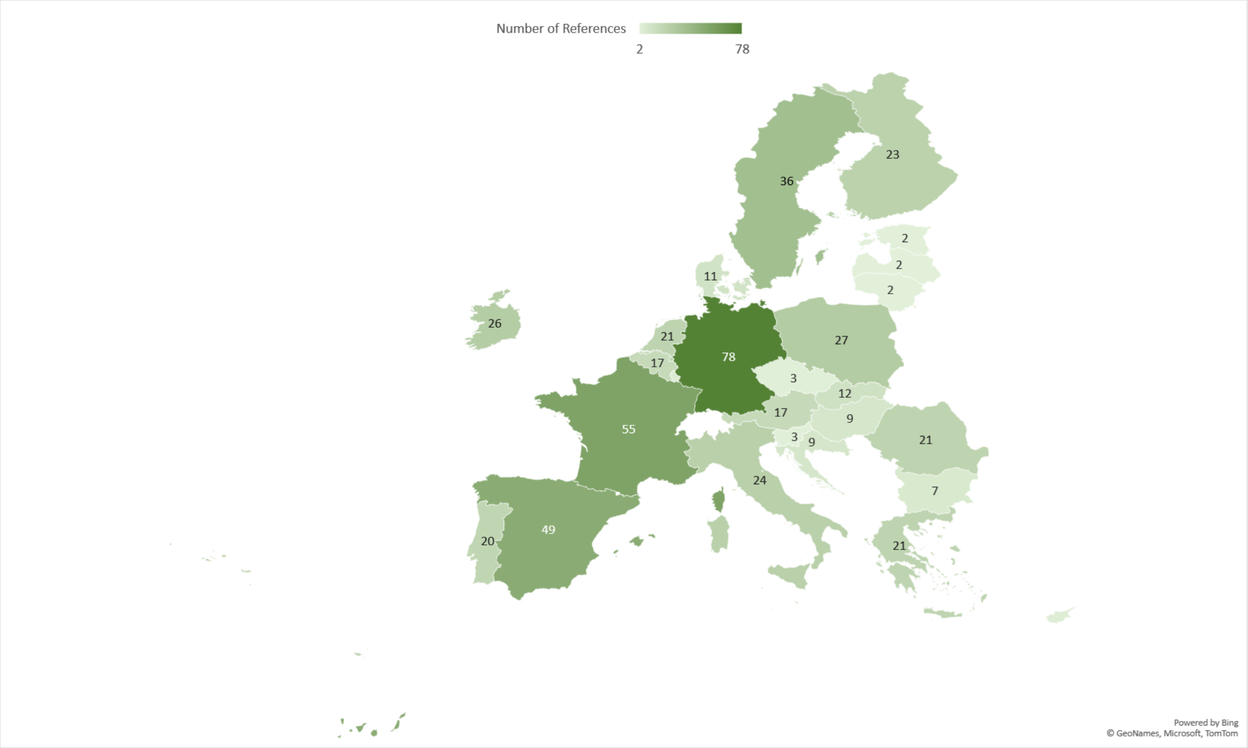

Figure 5.11 Total number of references to climate change in the European Parliament debates by country.

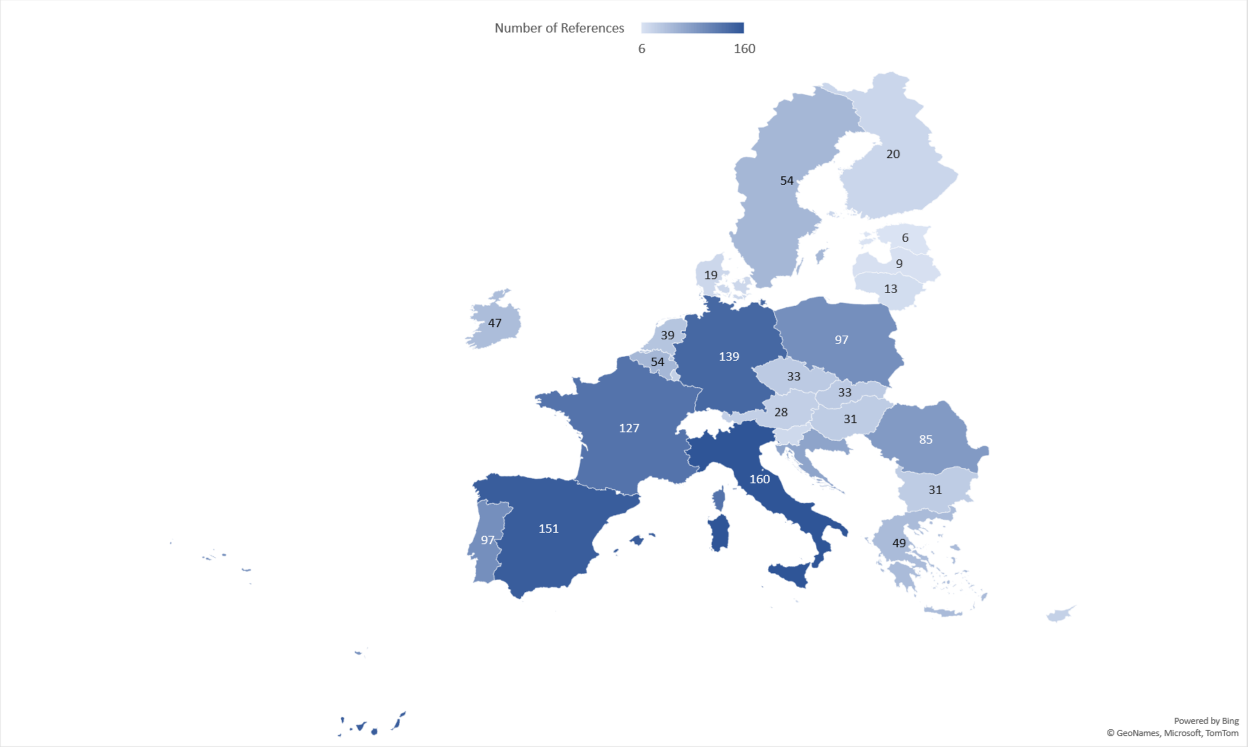

Figure 5.12 Total number of references to health in the European Parliament debates by country.

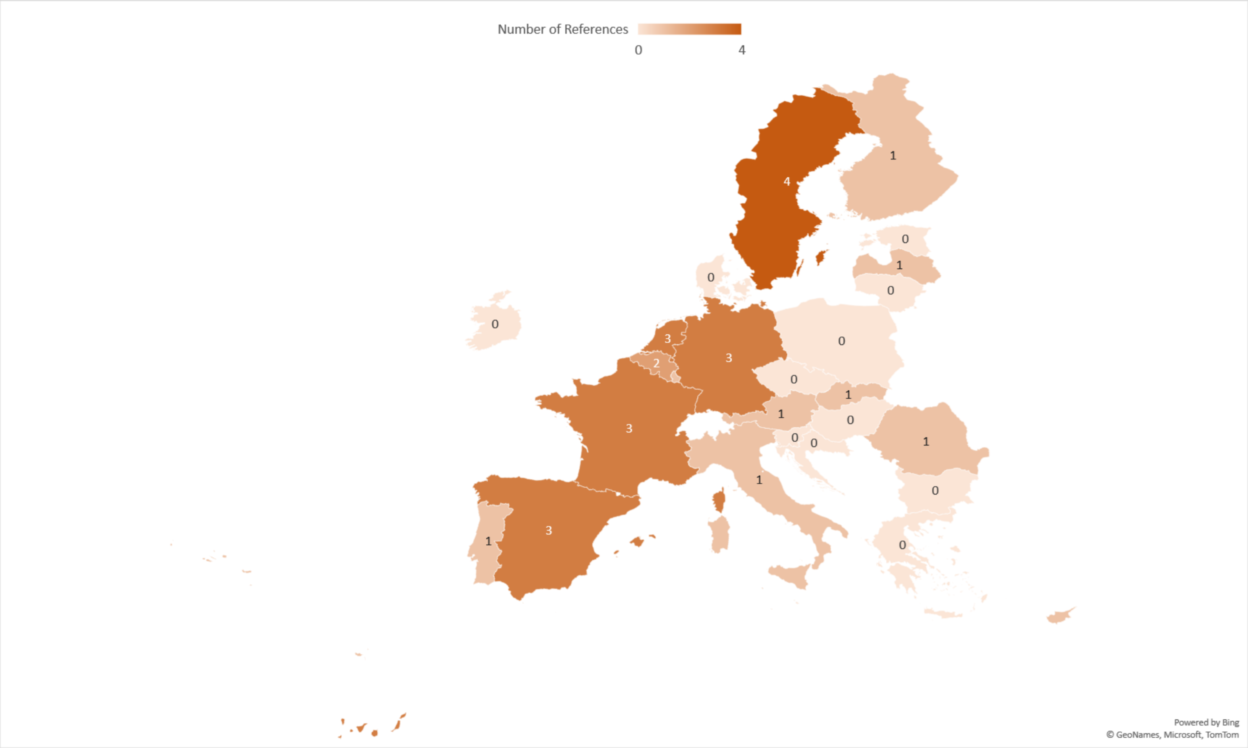

Figure 5.13 Total number of references to the intersection of health and climate change in the European Parliament debates by country.

Additional Analysis on Health and Climate Change

We present some additional findings and figures in this section.

Figure 5.14 shows the number of references to health, climate change, and the intersection of health and climate change for the EU parliamentary debates over time. This data is not normalised by number of sessions for each year, and instead provides a general overview of the years. The figure shows that despite the variability in discussing health and climate change individually, there is still a very low engagement with the impact of climate change on health, and its implications.

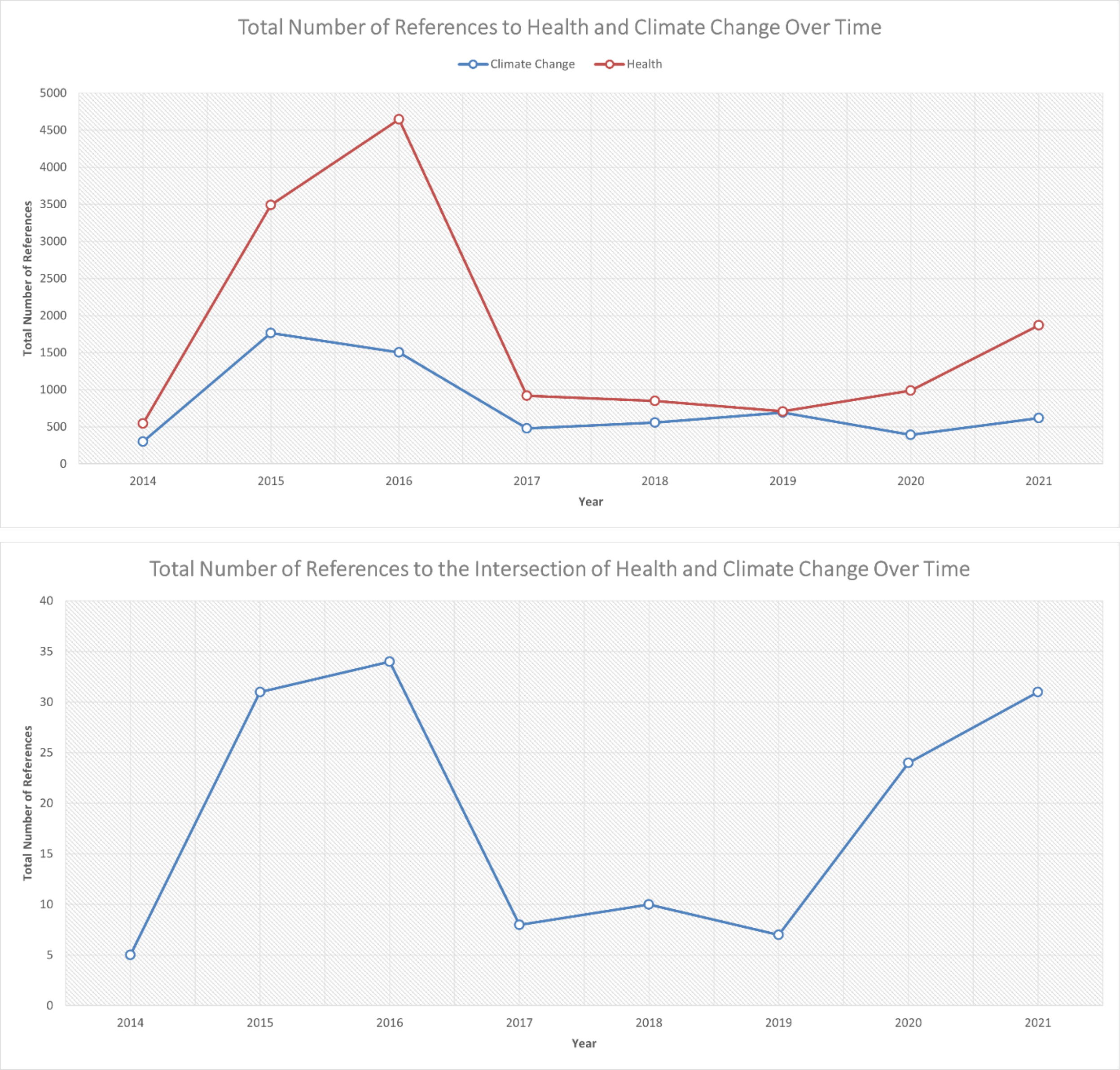

Figure 5.14 Total number of references to health, climate change, and the intersection of health and climate change over time, 2014-2021

Figure 5.15 presents the frequency of terms related to health, climate change, and the intersection of health and climate change for each political group within the European Parliament. The figure shows relatively low engagement with the impacts of climate change on health, despite a high investment in health from at least two of the participating political groups (EPP and S&D). The health implications of climate change remain largely untouched.

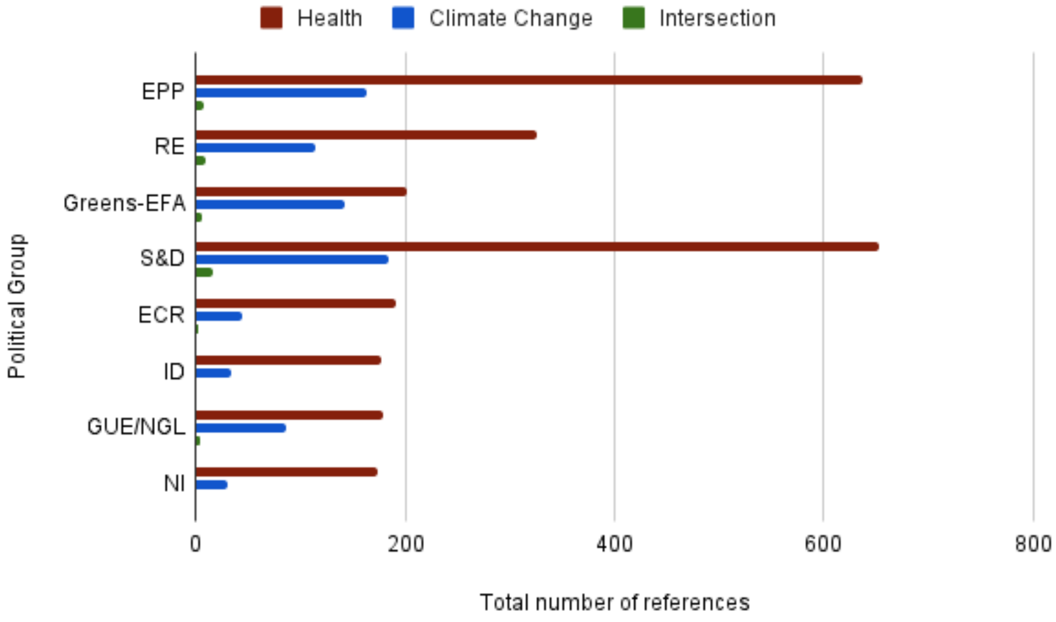

Figure 5.15 Total number of references to health, climate change, and the intersection of health and climate change by political group, 2014-2021.

Figure 5.16 shows the total number of references to health, climate change and the intersection of health and climate change broken down by each participating country. The bar chart shows that despite the high engagement in health terms at the debates, the connection to climate change remains unexplored for most countries, with only Germany, France and Sweden making a small connection.

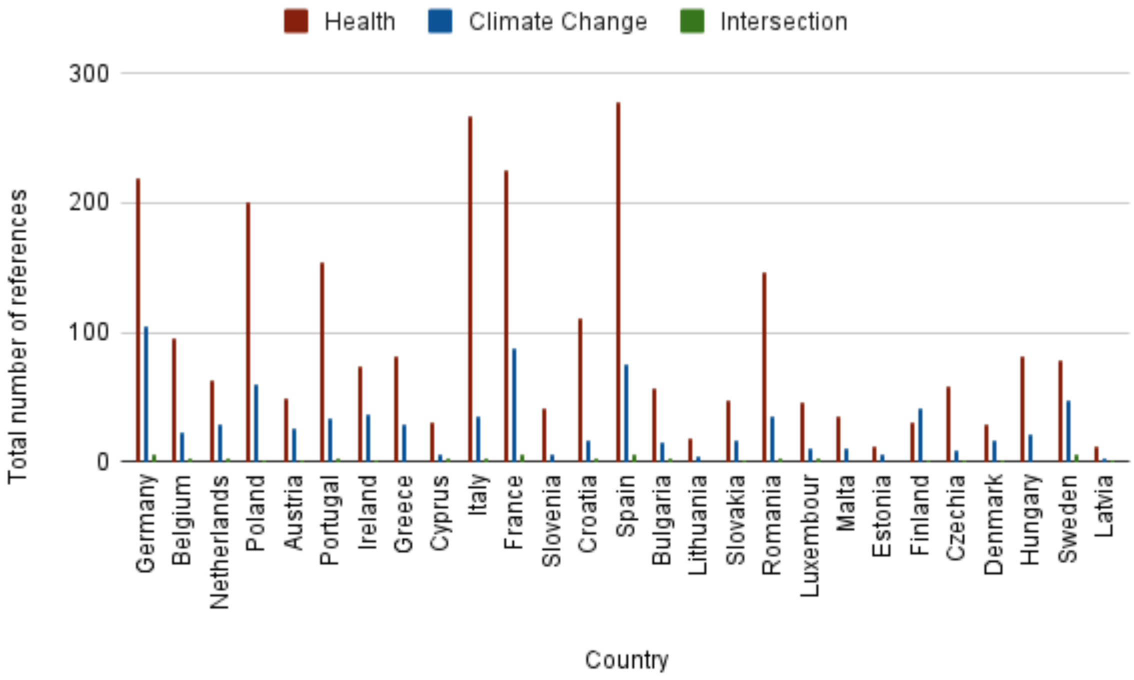

Figure 5.16 Total number of references to health, climate change, and the intersection of health and climate change by country, 2014-2021.

Further Analysis on Health and Climate Change by Country and Political Party

The figures in this section provide a comprehensive overview of each of the 28 participating EEA countries and the level of engagement of their political parties, showing the total number of references to health and climate change (and their intersection).

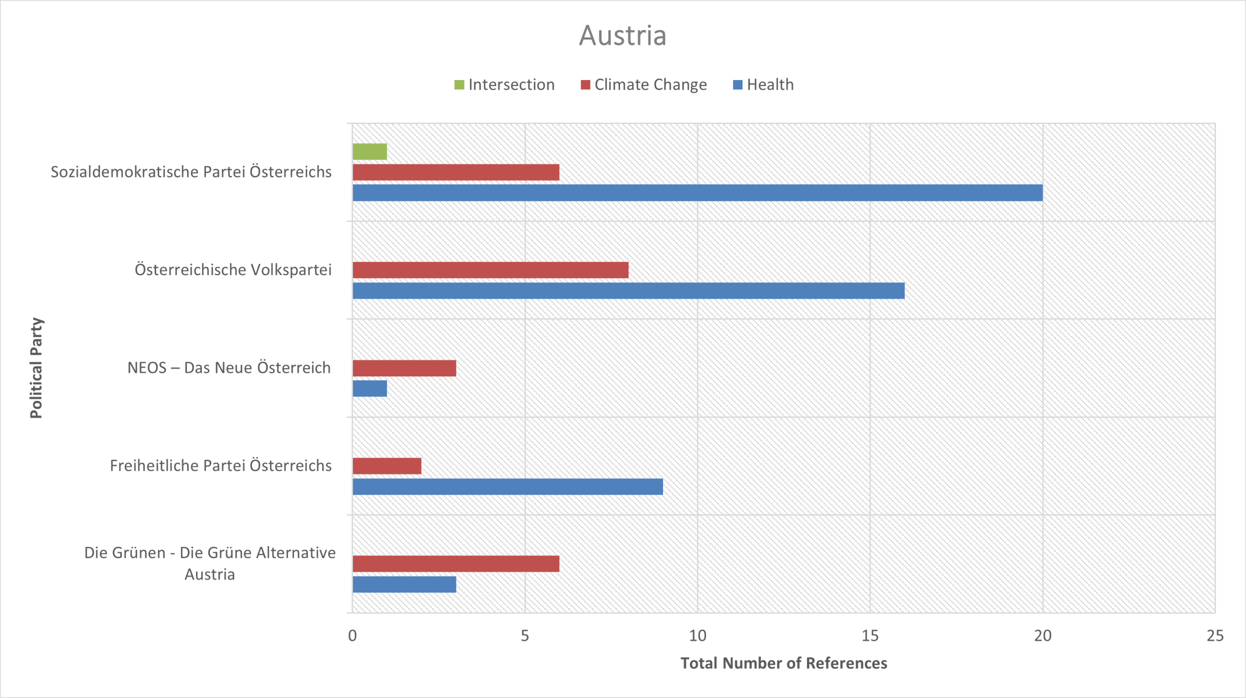

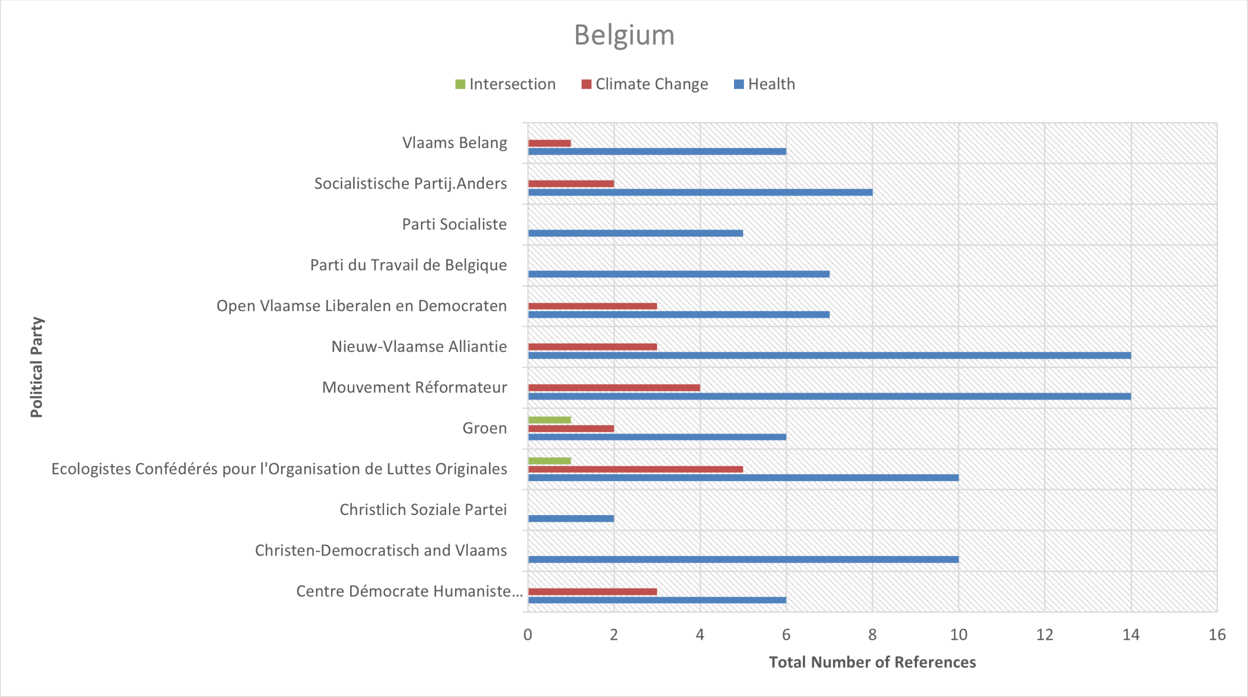

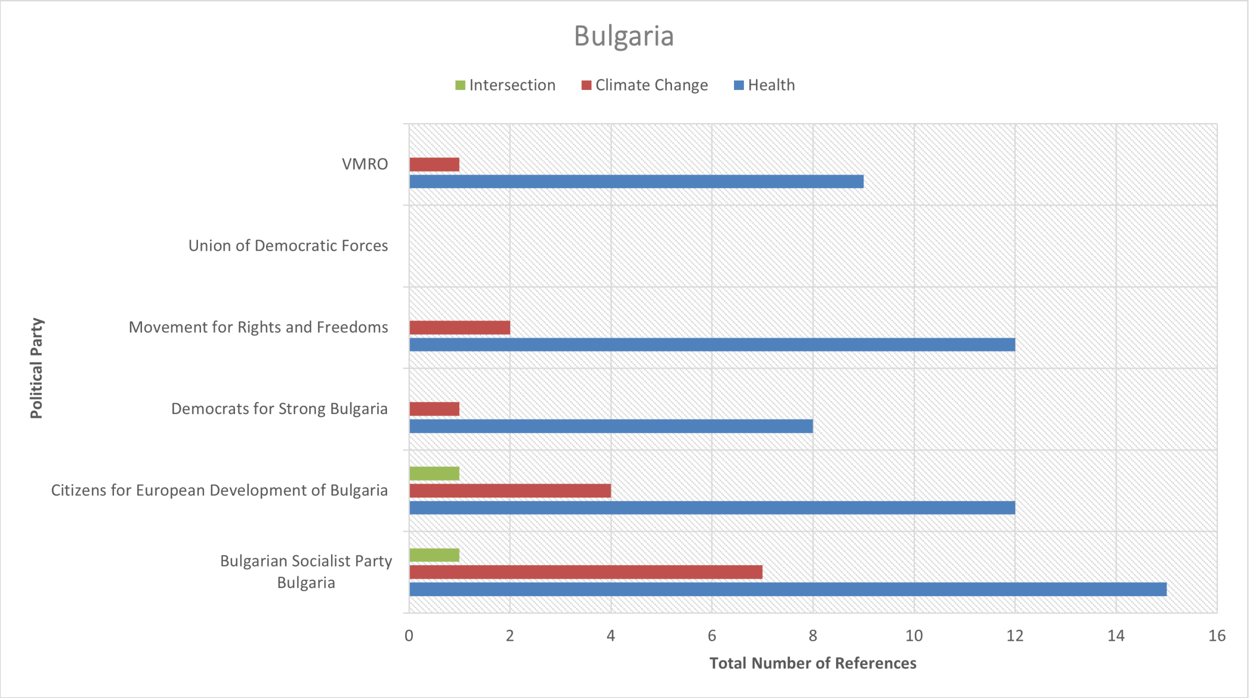
Bulgaria
Intersection
Climate Change
Health
VMRO
Union of Democratic Forces
Movement for Rights and Freedoms
Democrats for Strong Bulgaria
Citizens for European Development of Bulgaria
Bulgarian Socialist Party
Bulgaria
Political Party
0
2
4
6
8
10
12
14
16
Total Number of References

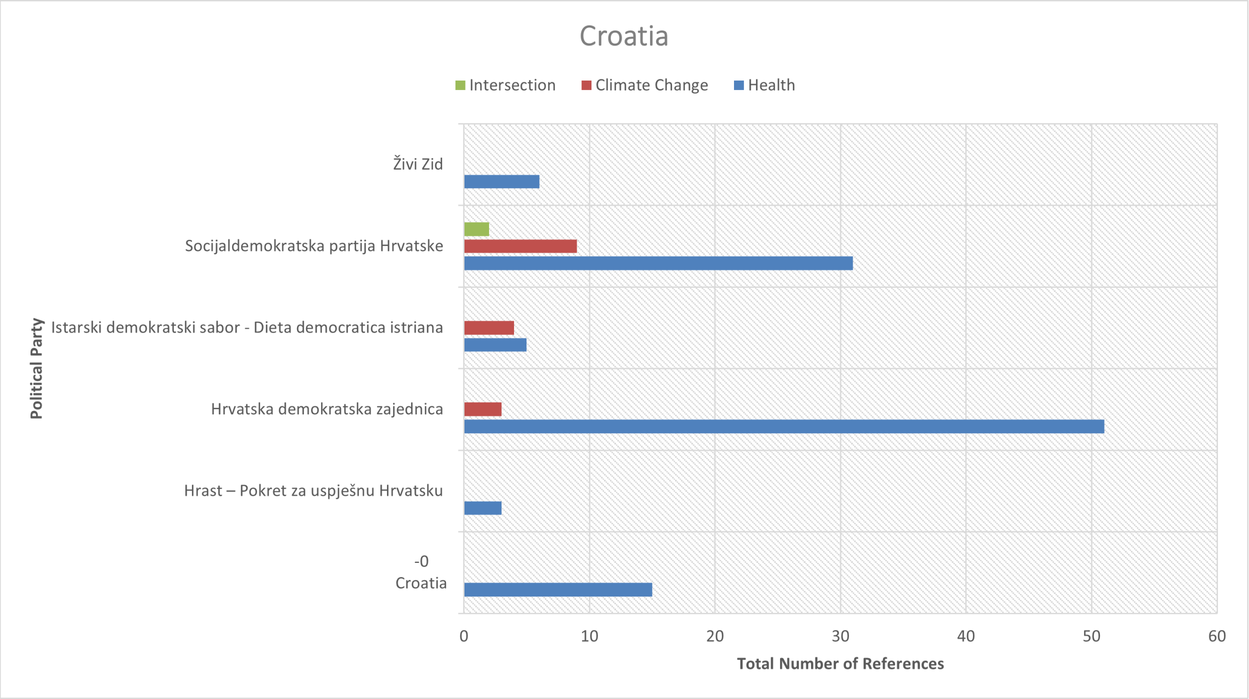
Croatia
Intersection
Climate Change
Health
Živi Zid
Socijaldemokratska partija Hrvatske
Istarski demokratski sabor - Dieta democratica istriana
Hrvatska demokratska zajednica
Hrast – Pokret za uspješnu Hrvatsku
-0
Croatia
Political Party
0
10
20
30
40
50
60
Total Number of References

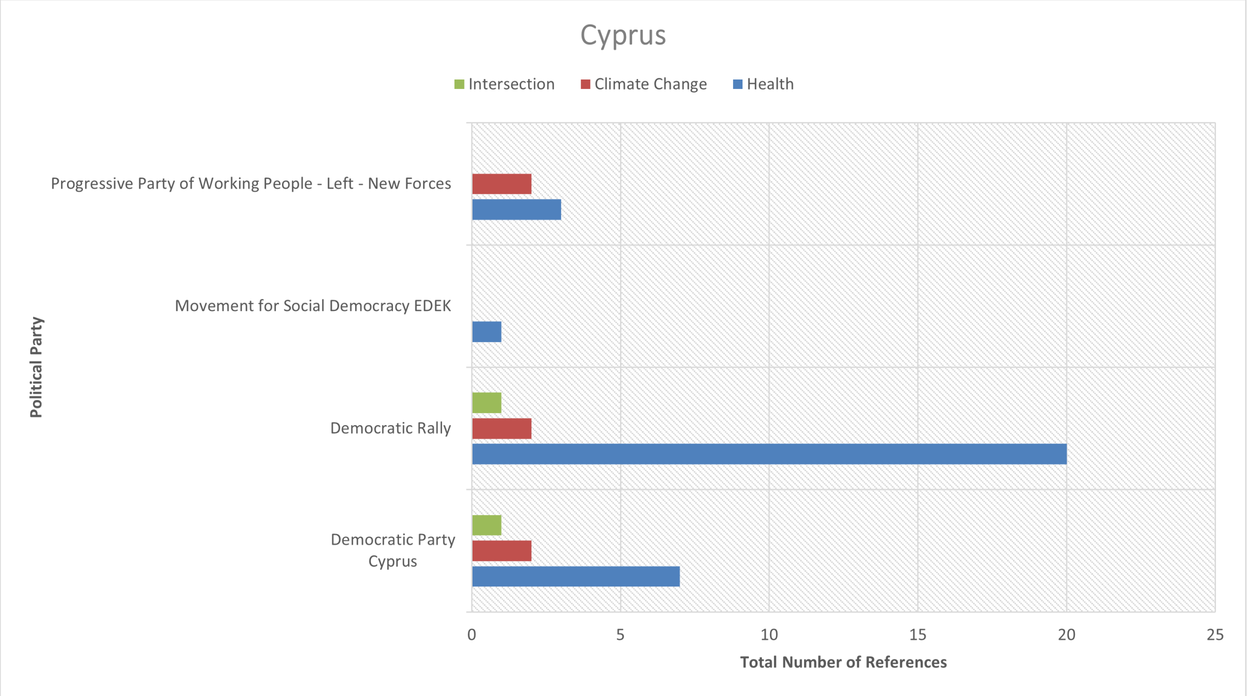
Cyprus
Intersection
Climate Change
Health
Political Party
Progressive Party of Working People - Left - New Forces
Movement for Social Democracy EDEK
Democratic Rally
Democratic Party
Cyprus
0
5
10
15
20
25
Total Number of References

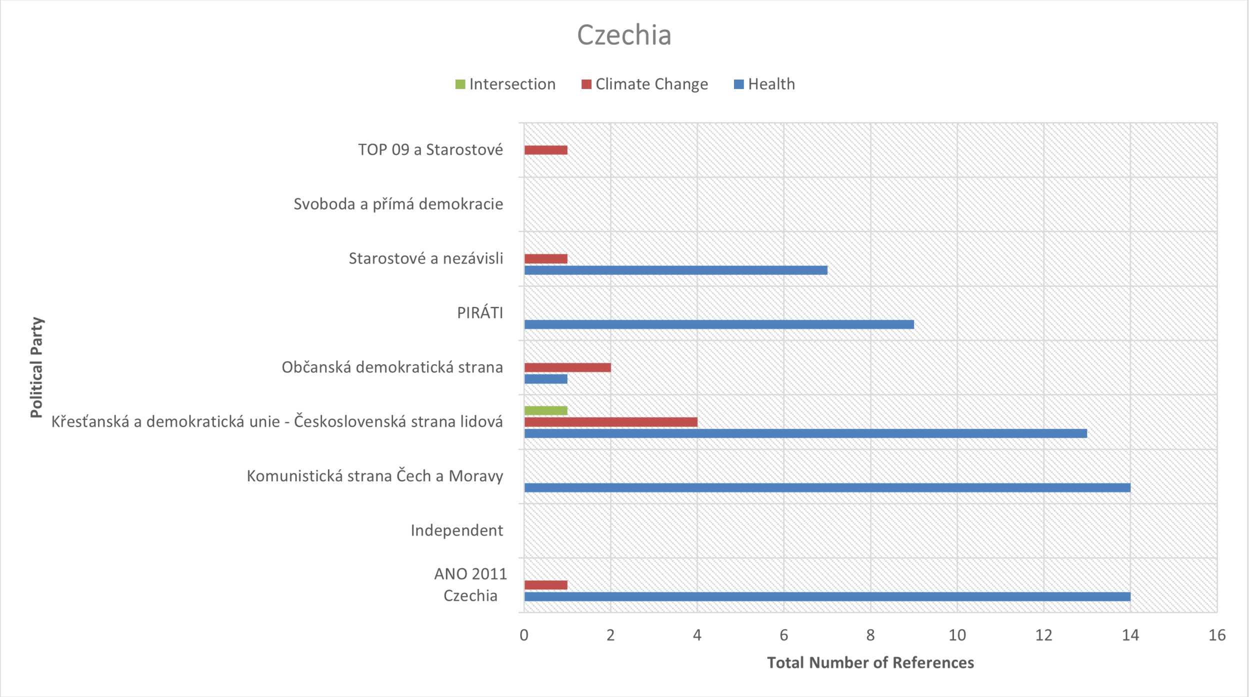
Czechia
Intersection
Climate Change
Health
Political Party
TOP 09 a Starostové
Svoboda a přímá demokracie
Starostové a nezávisli
PIRÁTI
Občanská demokratická strana
Křesťanská a demokratická unie - Československá strana lidová
Komunistická strana Čech a Moravy
Independent
ANO 2011
Czechia
0
2
4
6
8
10
12
14
16
Total Number of References

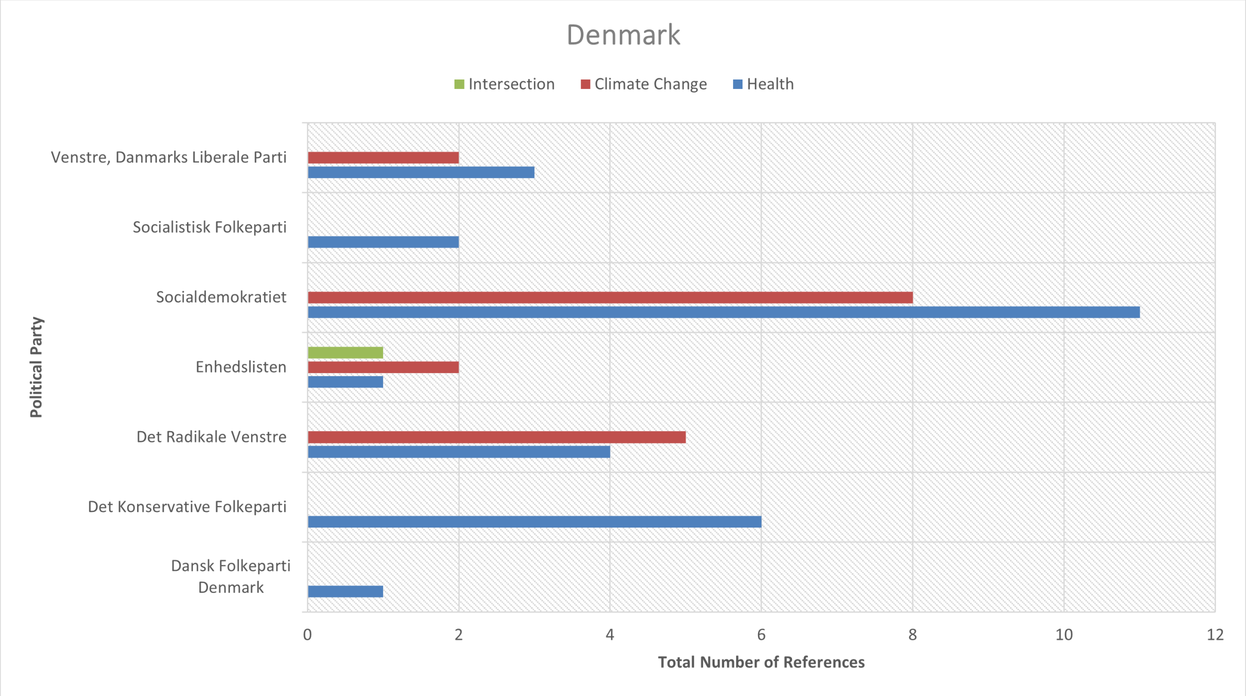
Denmark
Intersection
Climate Change
Health
Political Party
Venstre, Danmarks Liberale Parti
Socialistisk Folkeparti
Socialdemokratiet
Enhedslisten
Det Radikale Venstre
Det Konservative Folkeparti
Dansk Folkeparti
Denmark
0
2
4
6
8
10
12
Total Number of References

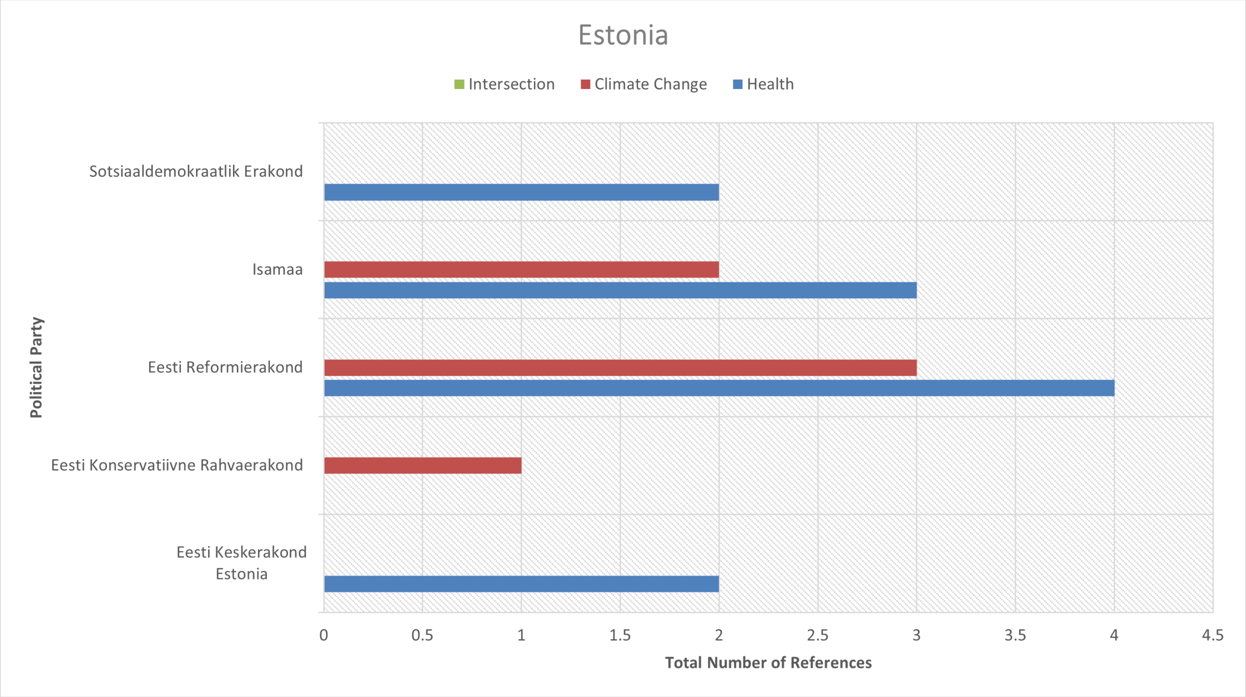
Estonia
Intersection
Climate Change
Health
Political Party
Sotsiaaldemokraatlik Erakond
Isamaa
Eesti Reformierakond
Eesti Konservatiivne Rahvaerakond
Eesti Keskerakond
Estonia
0
0.5
1
1.5
2
2.5
3
3.5
4
4.5
Total Number of References

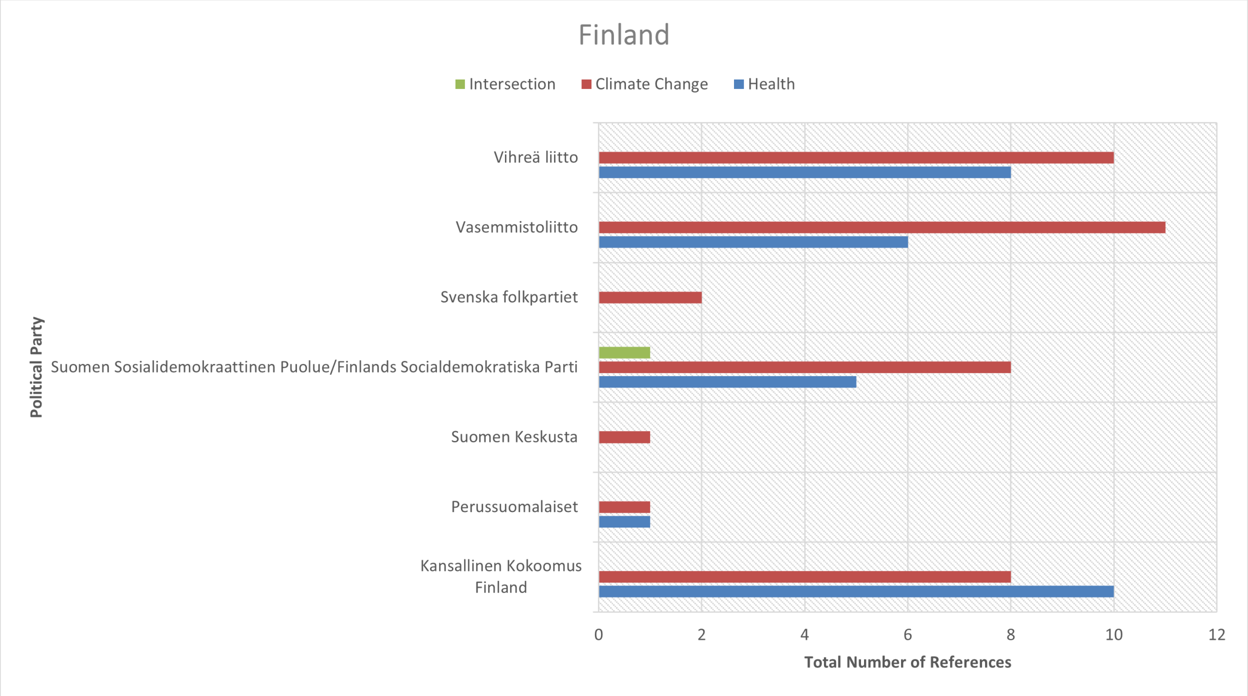
Finland
Intersection
Climate Change
Health
Political Party
Vihreä liitto
Vasemmistoliitto
Svenska folkpartiet
Suomen Sosialidemokraattinen Puolue/Finlands Socialdemokratiska Parti
Suomen Keskusta
Perussuomalaiset
Kansallinen Kokoomus Finland
0
2
4
6
8
10
12
Total Number of References

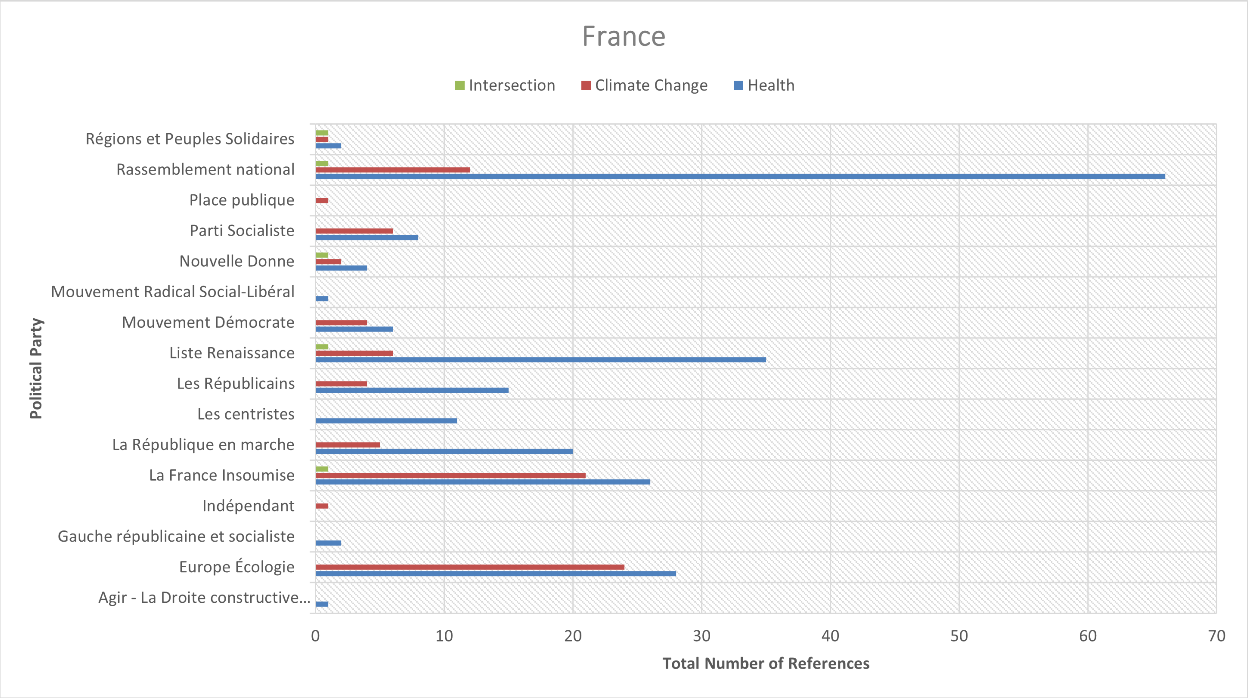
France
Intersection
Climate Change
Health
Political Party
Régions et Peuples Solidaires
Rassemblement national
Place publique
Parti Socialiste
Nouvelle Donne
Mouvement Radical Social-Libéral
Mouvement Démocrate
Liste Renaissance
Les Républicains
Les centristes
La République en marche
La France Insoumise
Indépendant
Gauche républicaine et socialiste
Europe Écologie
Agir - La Droite constructive...
0
10
20
30
40
50
60
70
Total Number of References

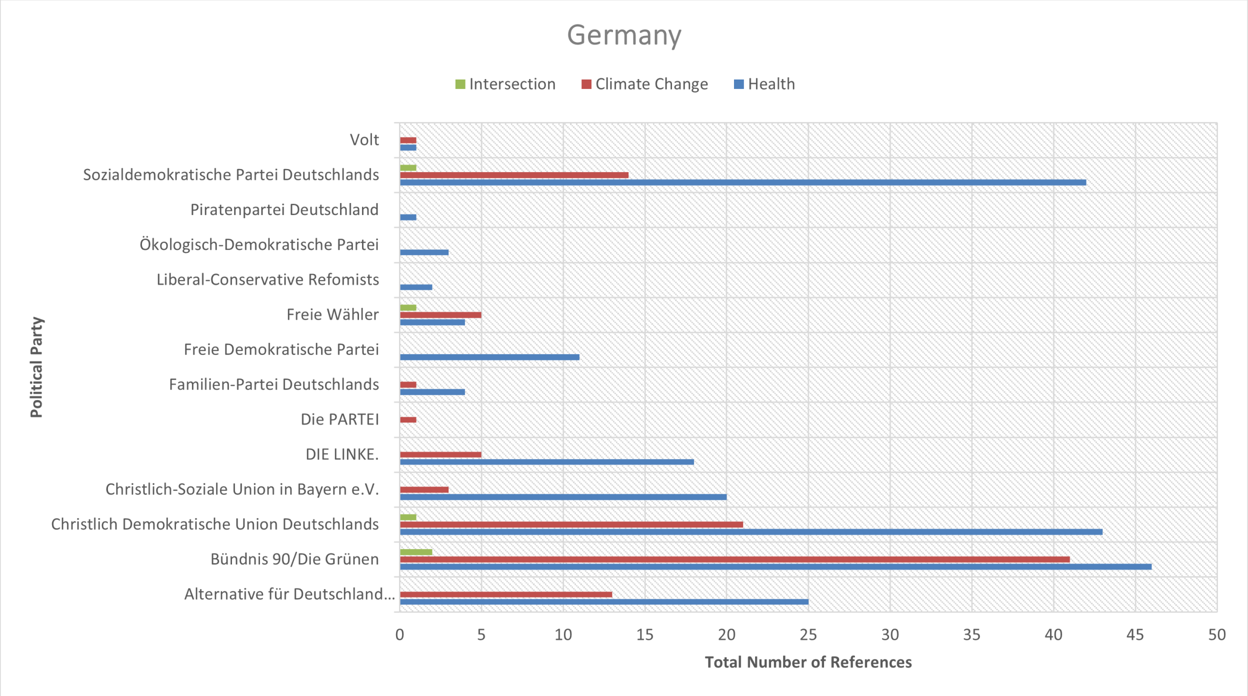
Germany
Intersection
Climate Change
Health
Political Party
Volt
Sozialdemokratische Partei Deutschlands
Piratenpartei Deutschland
Ökologisch-Demokratische Partei
Liberal-Conservative Refomists
Freie Wähler
Freie Demokratische Partei
Familien-Partei Deutschlands
Die PARTEI
DIE LINKE.
Christlich-Soziale Union in Bayern e.V.
Christlich Demokratische Union Deutschlands
Bündnis 90/Die Grünen
Alternative für Deutschland...
0
5
10
15
20
25
30
35
40
45
50
Total Number of References

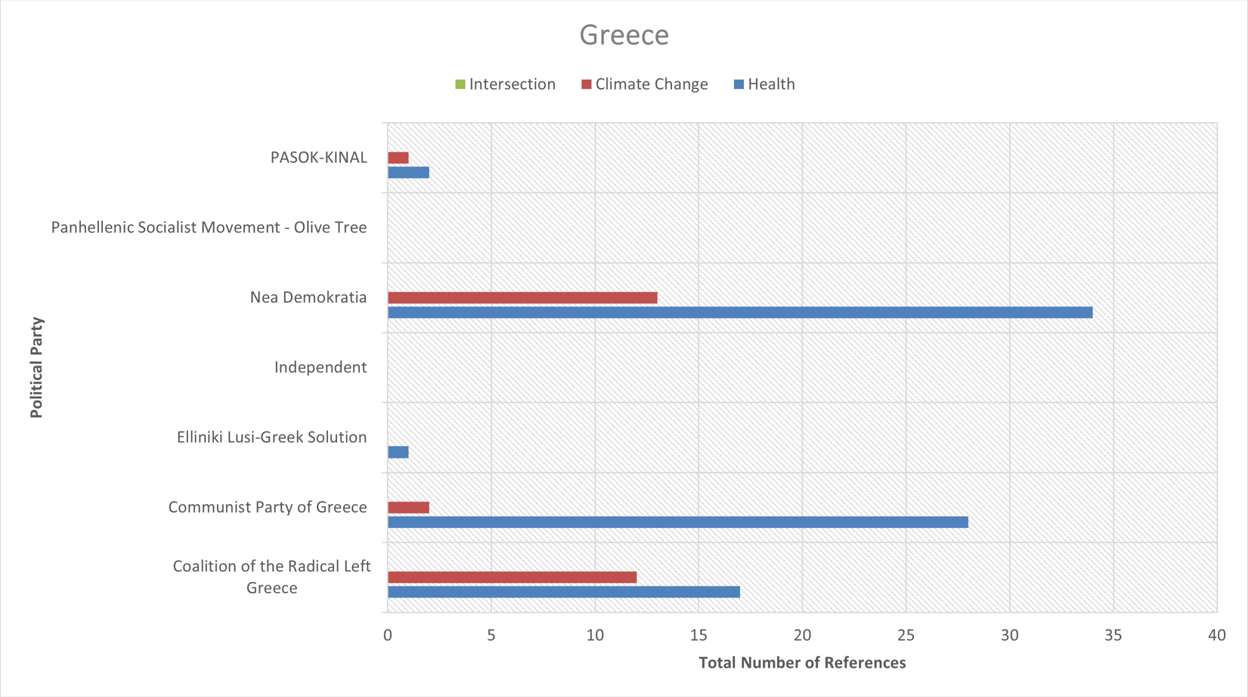
Greece
Intersection
Climate Change
Health
Political Party
PASOK-KINAL
Panhellenic Socialist Movement - Olive Tree
Nea Demokratia
Independent
Elliniki Lusi-Greek Solution
Communist Party of Greece
Coalition of the Radical Left Greece
0
5
10
15
20
25
30
35
40
Total Number of References

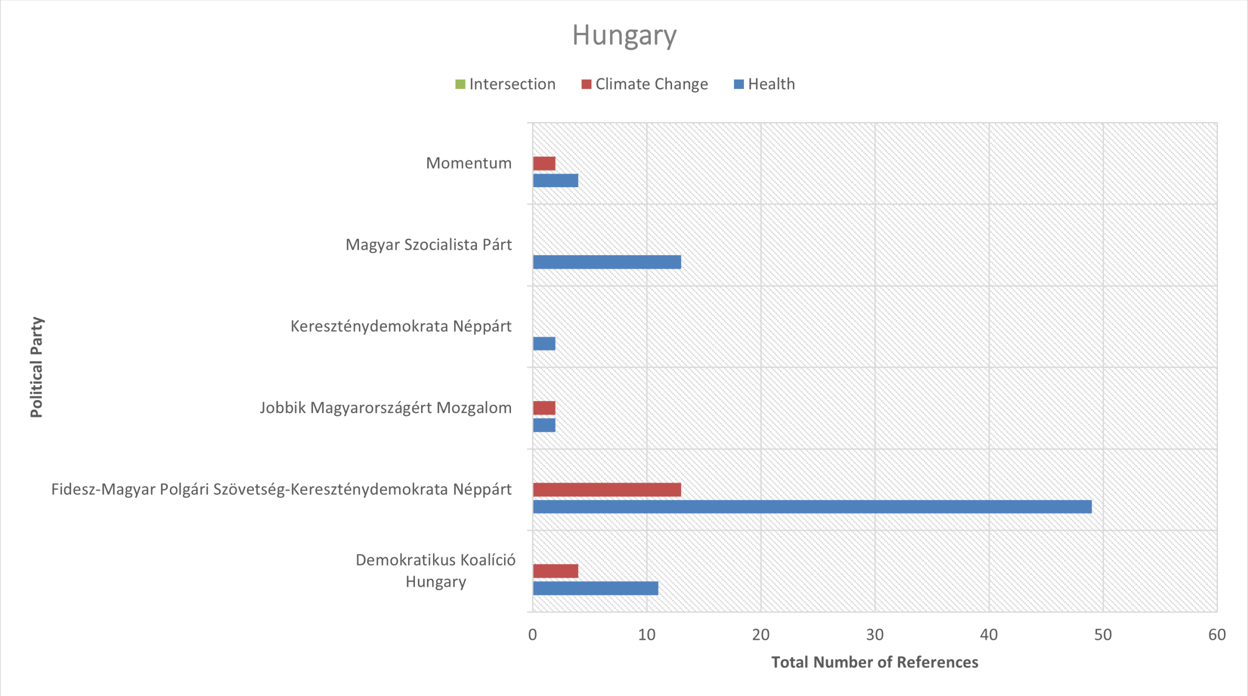
Hungary
Intersection
Climate Change
Health
Political Party
Momentum
Magyar Szocialista Párt
Kereszténydemokrata Néppárt
Jobbik Magyarországért Mozgalom
Fidesz-Magyar Polgári Szövetség-Kereszténydemokrata Néppárt
Demokratikus Koalíció Hungary
0
10
20
30
40
50
60
Total Number of References

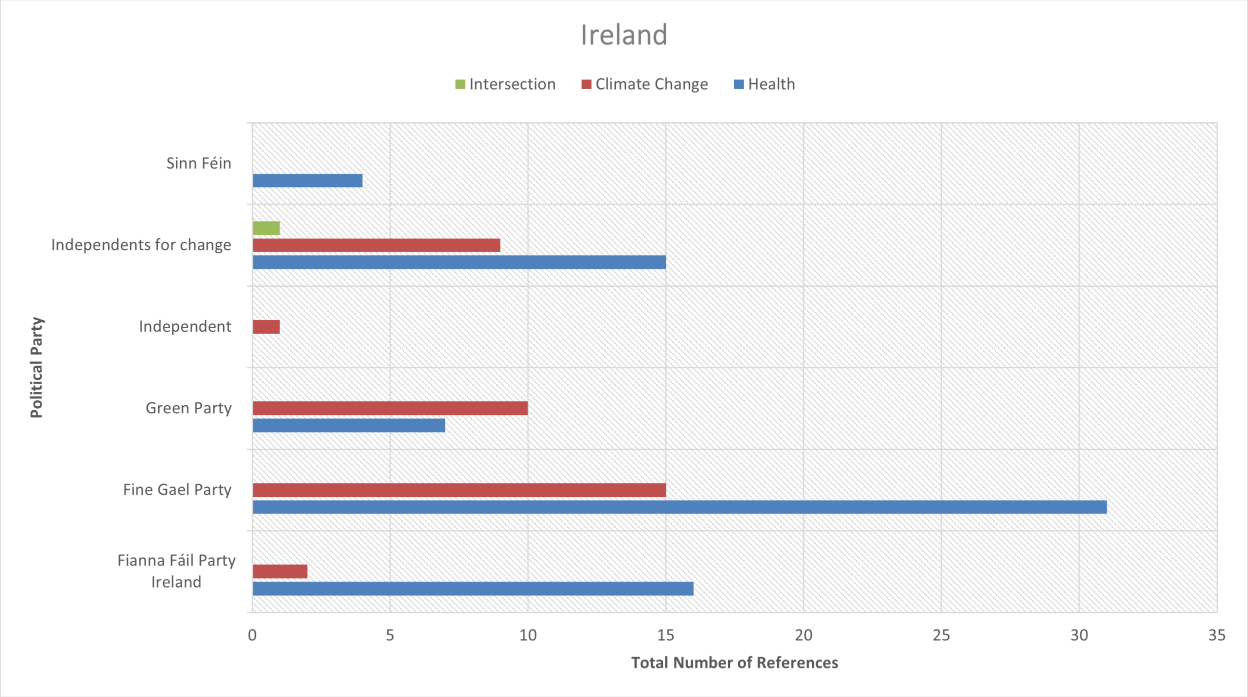
Ireland
Intersection
Climate Change
Health
Political Party
Sinn Féin
Independents for change
Independent
Green Party
Fine Gael Party
Fianna Fáil Party Ireland
0
5
10
15
20
25
30
35
Total Number of References

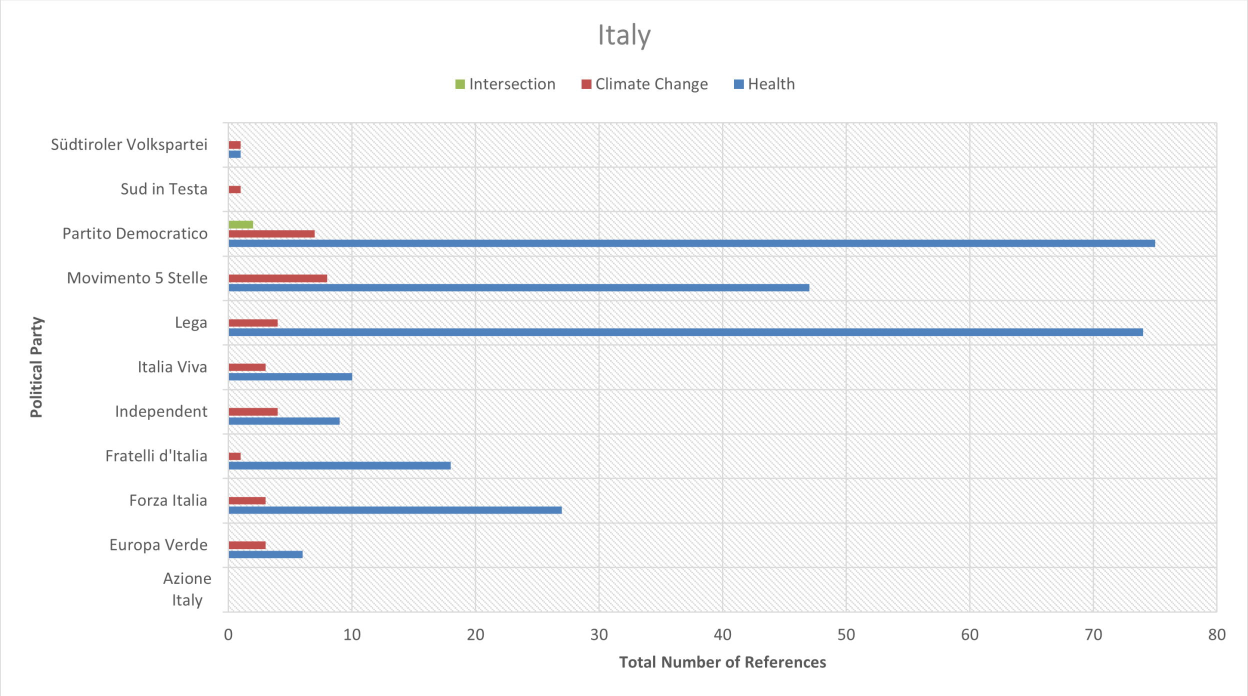
Italy
Intersection
Climate Change
Health
Südtiroler Volkspartei
Sud in Testa
Partito Democratico
Movimento 5 Stelle
Lega
Italia Viva
Independent
Fratelli d'Italia
Forza Italia
Europa Verde
Azione
Italy
Political Party
0
10
20
30
40
50
60
70
80
Total Number of References

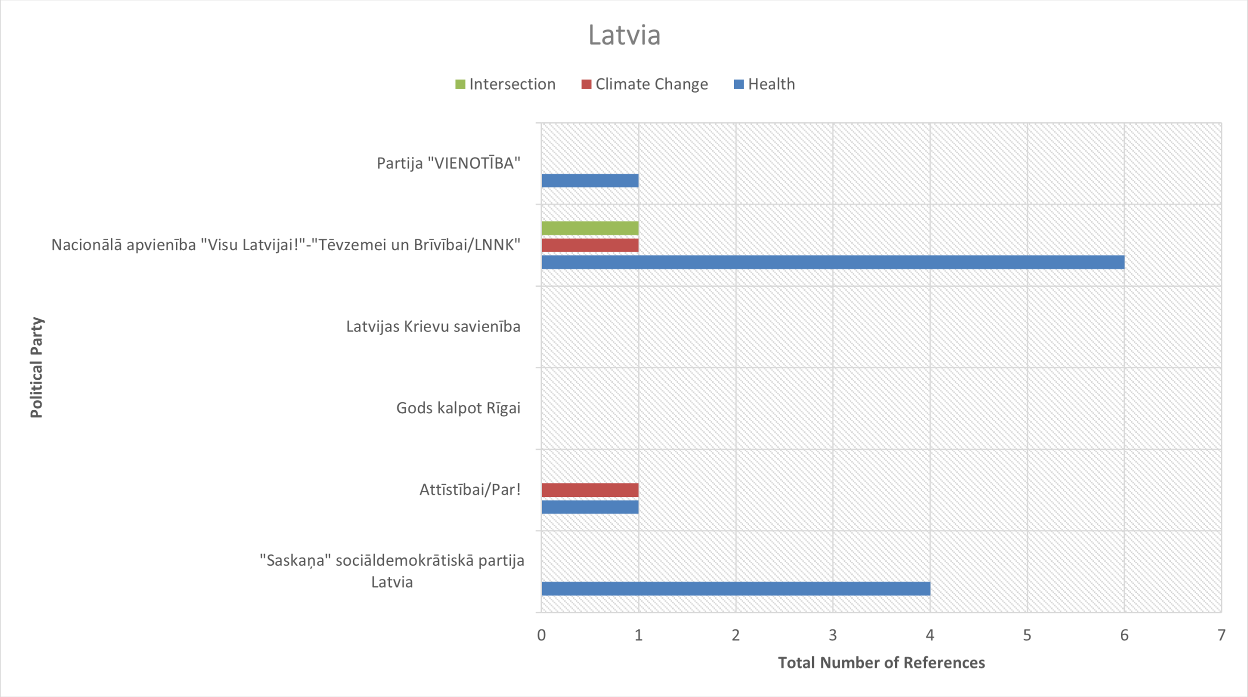
Latvia
Intersection
Climate Change
Health
Partija "VIENOTĪBA"
Nacionālā apvienība "Visu Latvijai!"-"Tēvzemei un Brīvībai/LNNK"
Latvijas Krievu savienība
Gods kalpot Rīgai
Attīstībai/Par!
"Saskaņa" sociāldemokrātiskā partija
Latvia
Political Party
0
1
2
3
4
5
6
7
Total Number of References

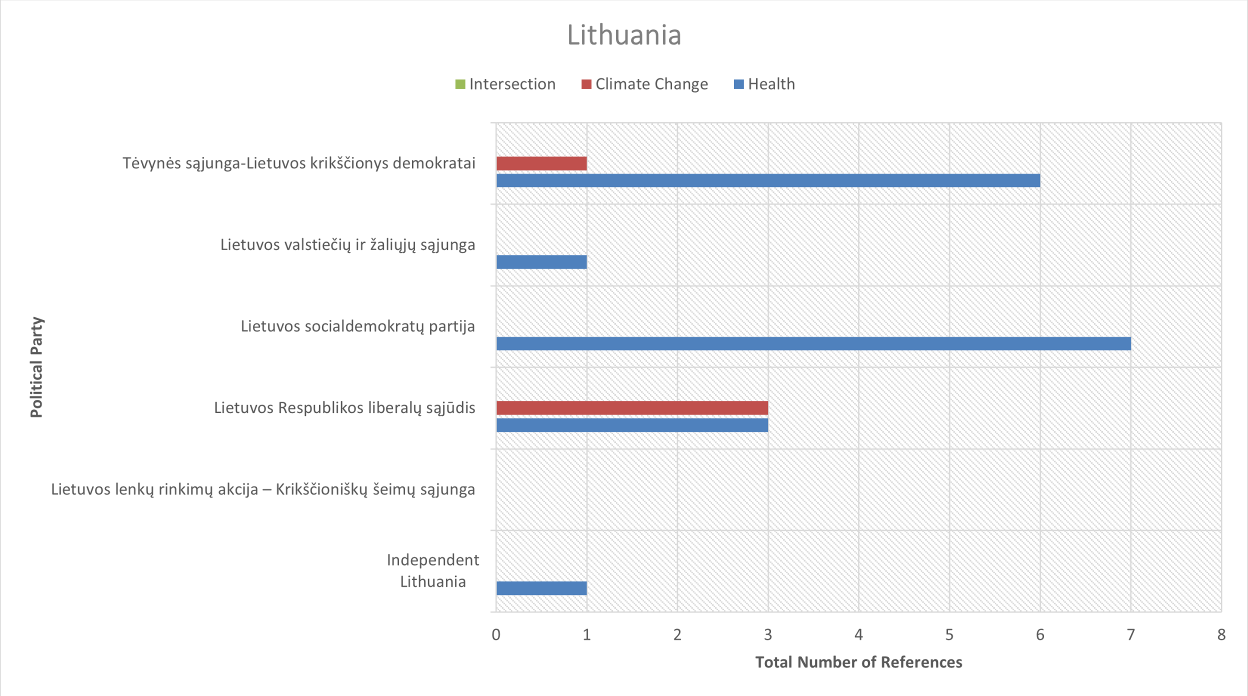
Lithuania
Intersection
Climate Change
Health
Political Party
Tėvynės sąjunga-Lietuvos krikščionys demokratai
Lietuvos valstiečių ir žaliųjų sąjunga
Lietuvos socialdemokratų partija
Lietuvos Respublikos liberalų sąjūdis
Lietuvos lenkų rinkimų akcija – Krikščioniškų šeimų sąjunga
Independent Lithuania
0
1
2
3
4
5
6
7
8
Total Number of References

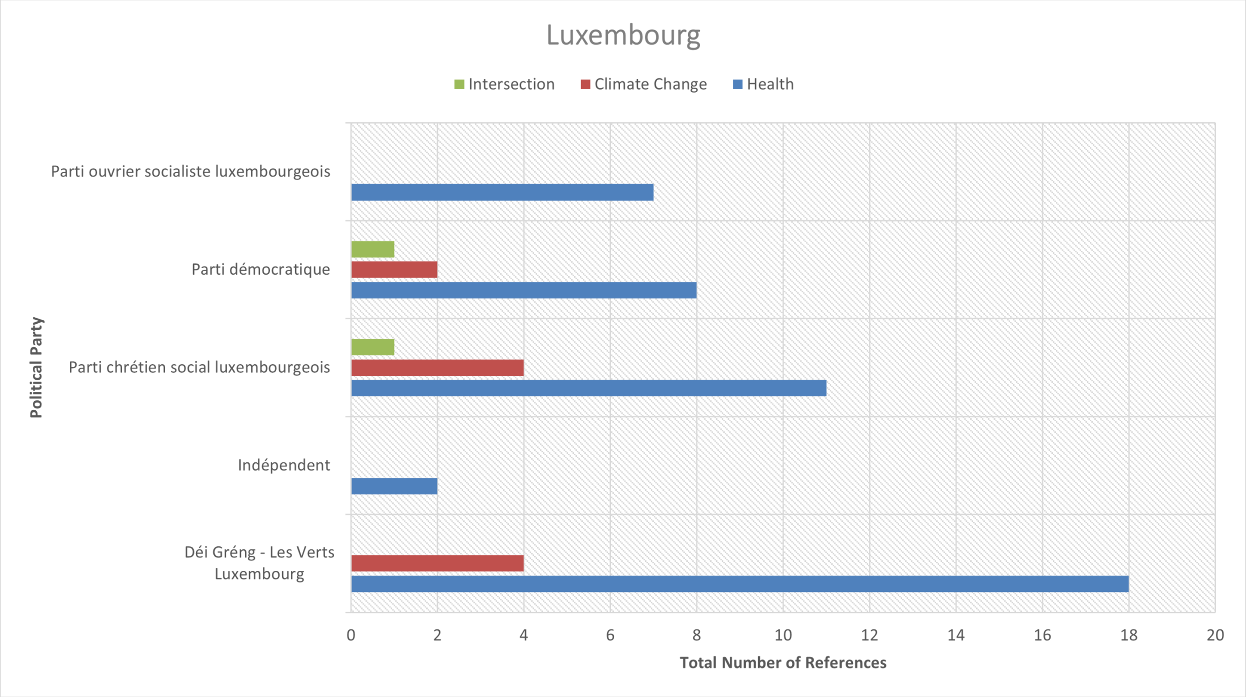
Luxembourg
Intersection
Climate Change
Health
Political Party
Parti ouvrier socialiste luxembourgeois
Parti démocratique
Parti chrétien social luxembourgeois
Indépendent
Déi Gréng - Les Verts Luxembourg
0
2
4
6
8
10
12
14
16
18
20
Total Number of References

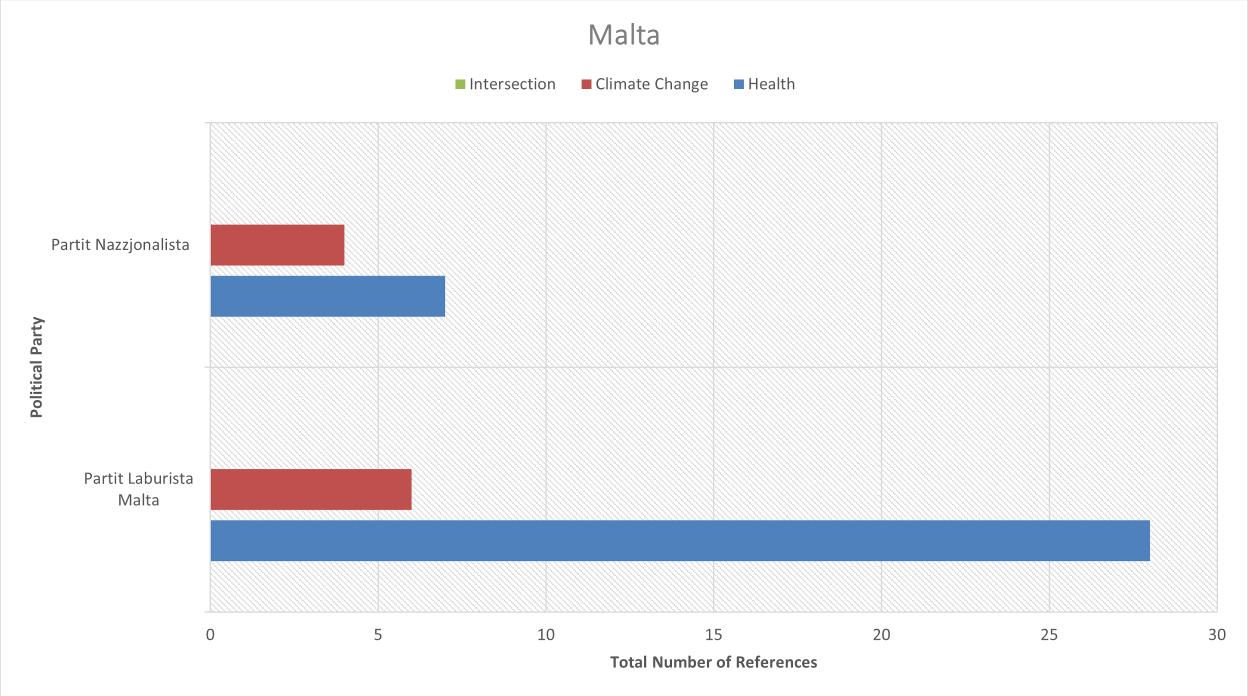
Malta
Intersection
Climate Change
Health
Political Party
Partit Nazzjonalista
Partit Laburista Malta
0
5
10
15
20
25
30
Total Number of References

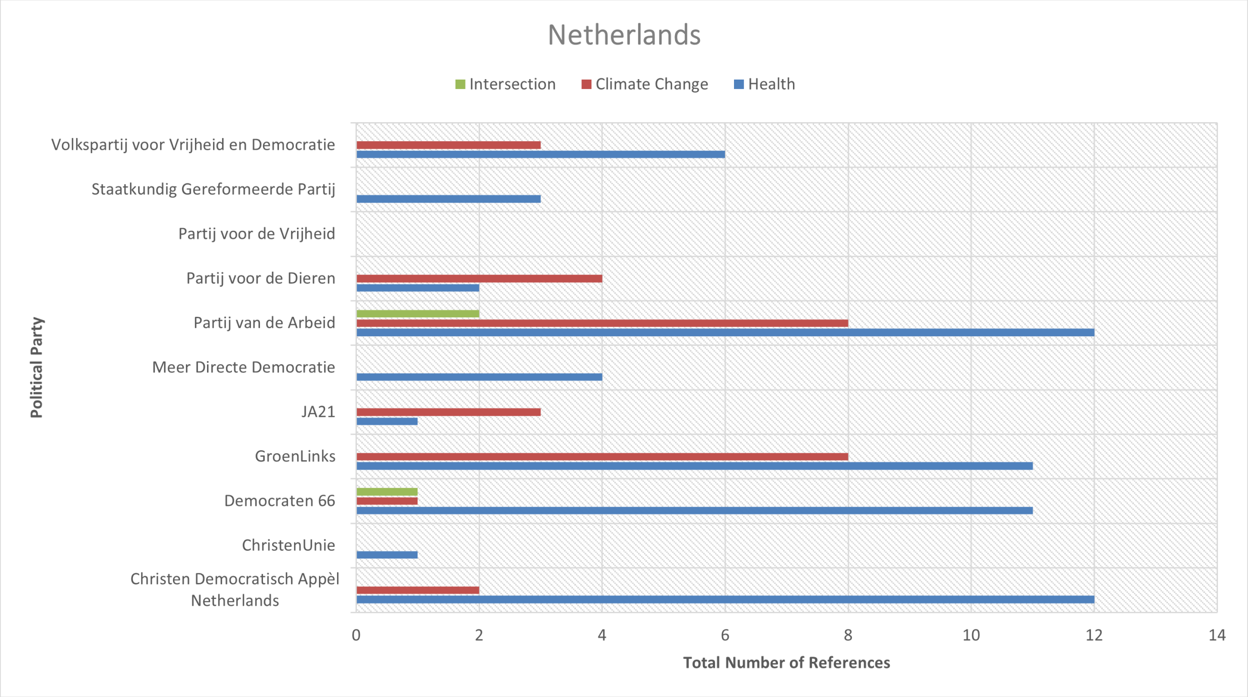
Netherlands
Intersection
Climate Change
Health
Political Party
Volkspartij voor Vrijheid en Democratie
Staatkundig Gereformeerde Partij
Partij voor de Vrijheid
Partij voor de Dieren
Partij van de Arbeid
Meer Directe Democratie
JA21
GroenLinks
Democraten 66
ChristenUnie
Christen Democratisch Appèl Netherlands
0
2
4
6
8
10
12
14
Total Number of References

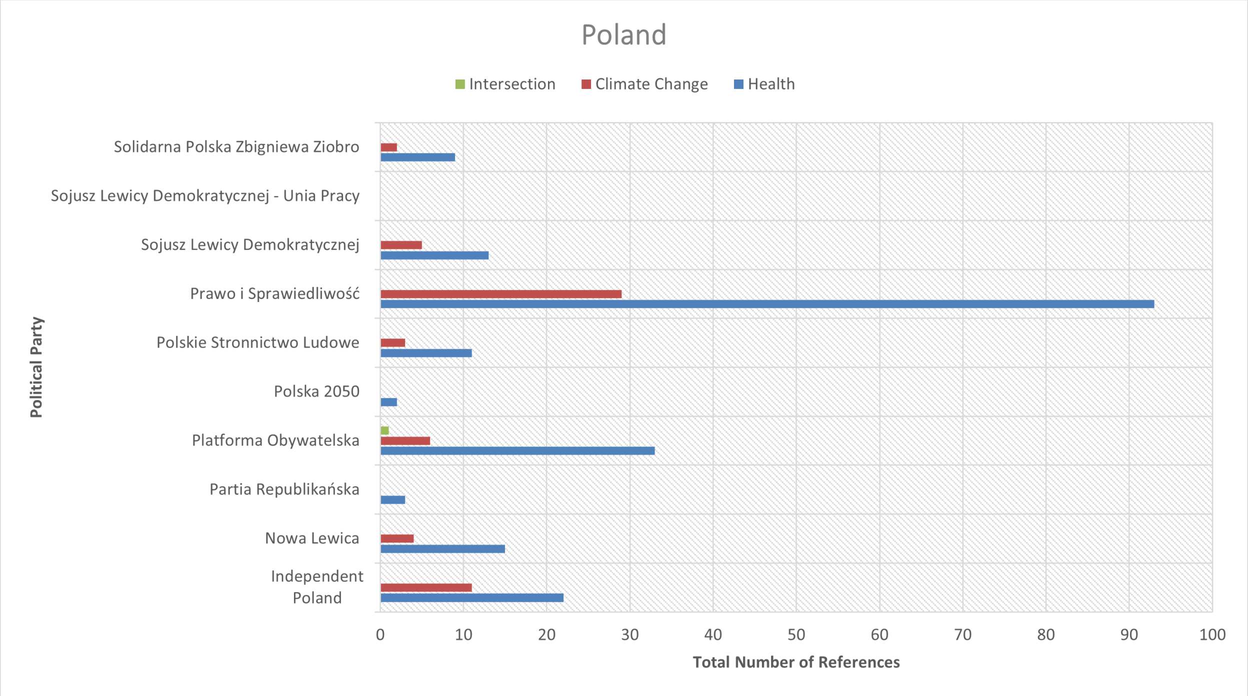
Poland
Intersection
Climate Change
Health
Political Party
Solidarna Polska Zbigniewa Ziobro
Sojusz Lewicy Demokratycznej - Unia Pracy
Sojusz Lewicy Demokratycznej
Prawo i Sprawiedliwość
Polskie Stronnictwo Ludowe
Polska 2050
Platforma Obywatelska
Partia Republikańska
Nowa Lewica
Independent Poland
0
10
20
30
40
50
60
70
80
90
100
Total Number of References

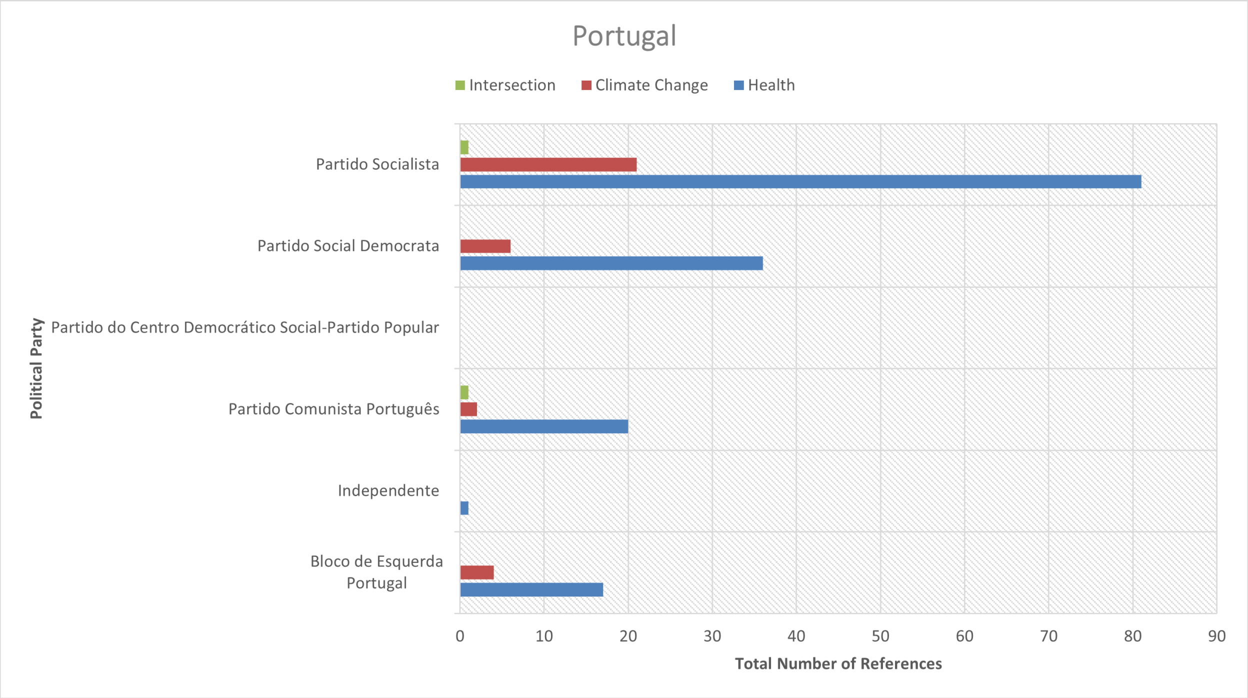
Portugal
Intersection
Climate Change
Health
Political Party
Partido Socialista
Partido Social Democrata
Partido do Centro Democrático Social-Partido Popular
Partido Comunista Português
Independente
Bloco de Esquerda Portugal
0
10
20
30
40
50
60
70
80
90
Total Number of References

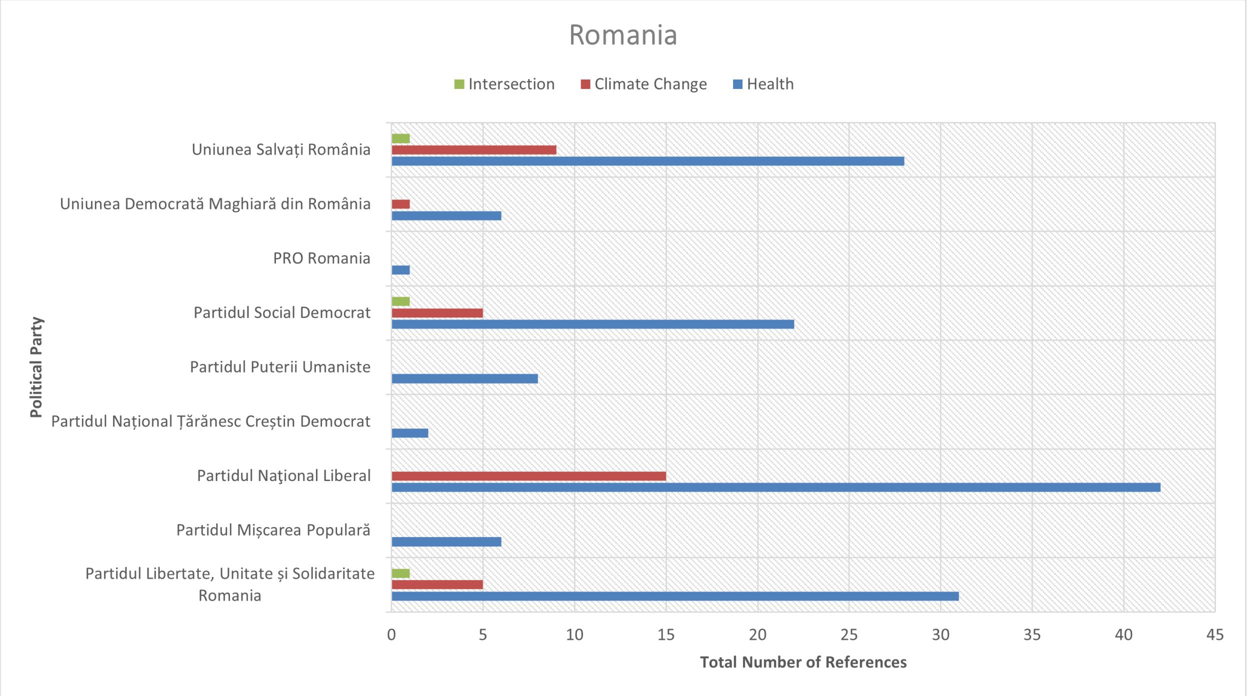
Romania
Intersection
Climate Change
Health
Political Party
Uniunea Salvați România
Uniunea Democrată Maghiară din România
PRO Romania
Partidul Social Democrat
Partidul Puterii Umaniste
Partidul Național Țărănesc Creștin Democrat
Partidul Naţional Liberal
Partidul Mișcarea Populară
Partidul Libertate, Unitate și Solidaritate Romania
0
5
10
15
20
25
30
35
40
45
Total Number of References

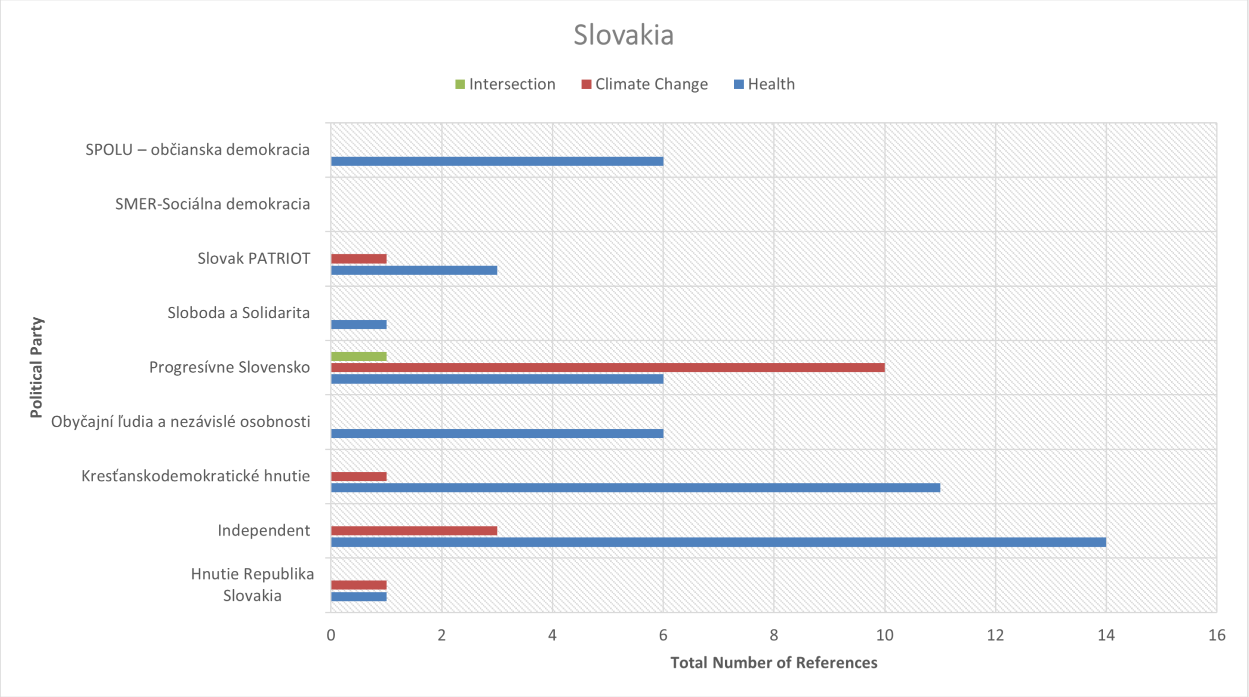
Slovakia
Intersection
Climate Change
Health
Political Party
SPOLU – občianska demokracia
SMER-Sociálna demokracia
Slovak PATRIOT
Sloboda a Solidarita
Progresívne Slovensko
Obyčajní ľudia a nezávislé osobnosti
Kresťanskodemokratické hnutie
Independent
Hnutie Republika Slovakia
0
2
4
6
8
10
12
14
16
Total Number of References

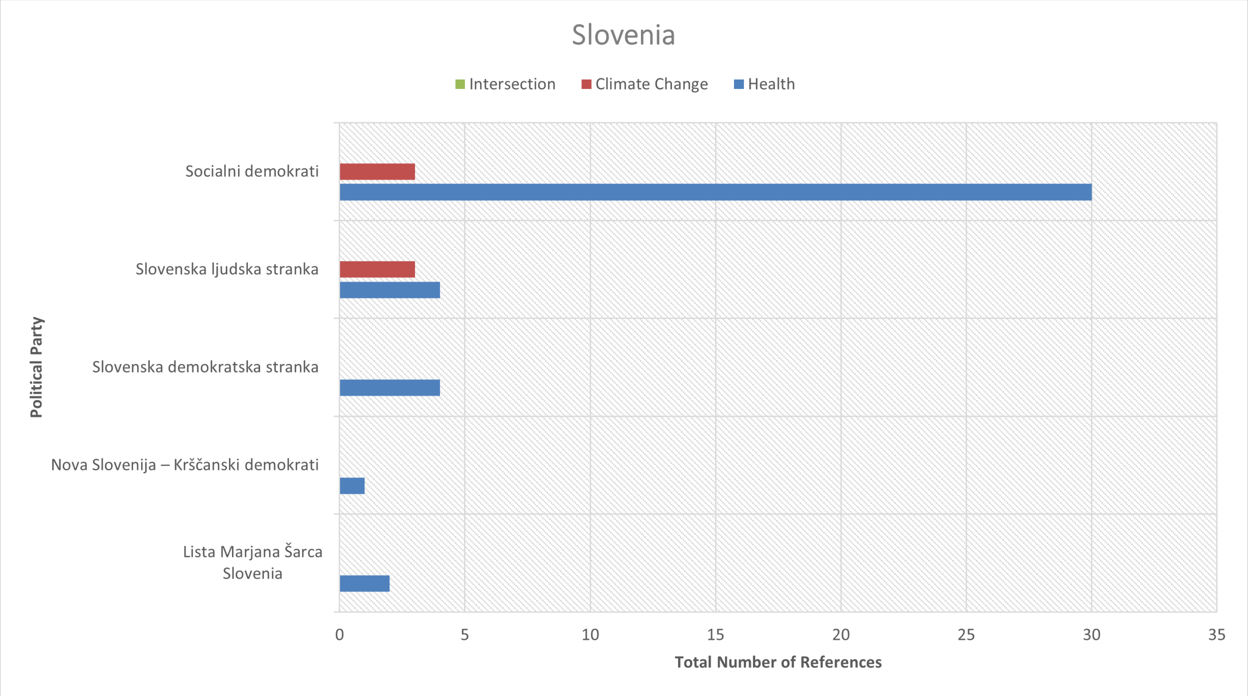

Slovenia
Intersection
Climate Change
Health
Socialni demokrati
Slovenska ljudska stranka
Slovenska demokratska stranka
Nova Slovenija – Krščanski demokrati
Lista Marjana Šarca Slovenia
Political Party
0
5
10
15
20
25
30
35
Total Number of References

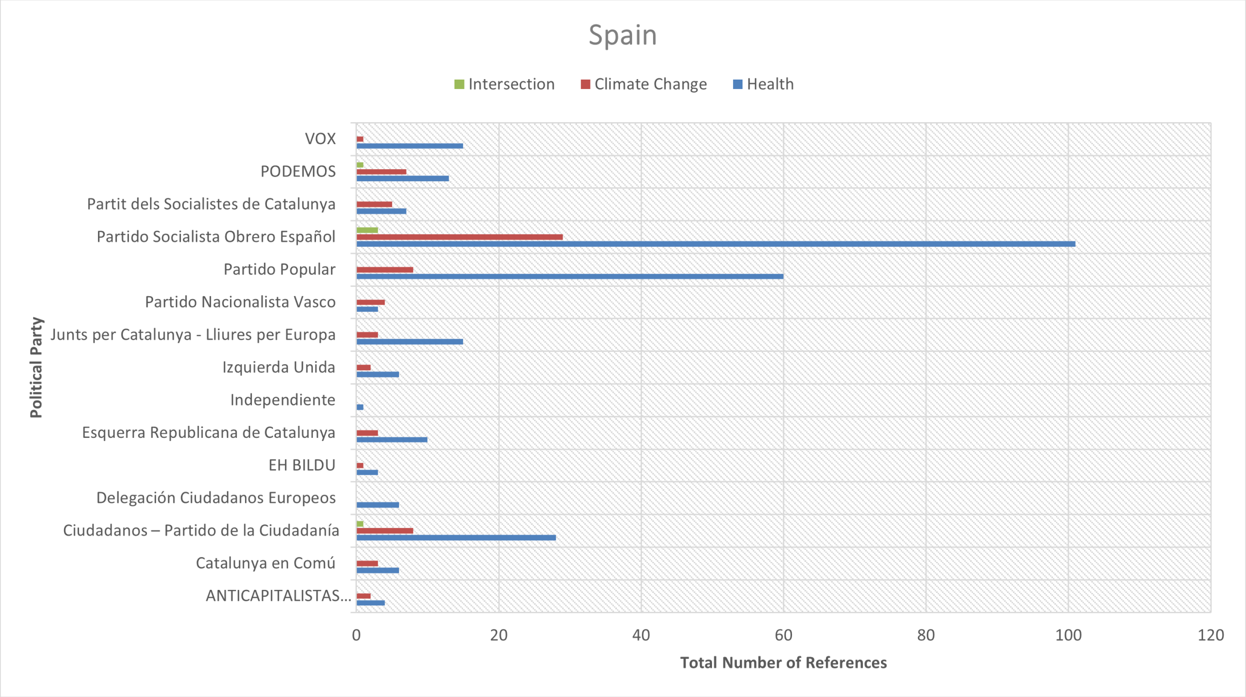

Spain
Intersection
Climate Change
Health
VOX
PODEMOS
Partit dels Socialistes de Catalunya
Partido Socialista Obrero Español
Partido Popular
Partido Nacionalista Vasco
Junts per Catalunya - Lliures per Europa
Izquierda Unida
Independiente
Esquerra Republicana de Catalunya
EH BILDU
Delegación Ciudadanos Europeos
Ciudadanos – Partido de la Ciudadanía
Catalunya en Comú
ANTICAPITALISTAS...
Political Party
0
20
40
60
80
100
120
Total Number of References

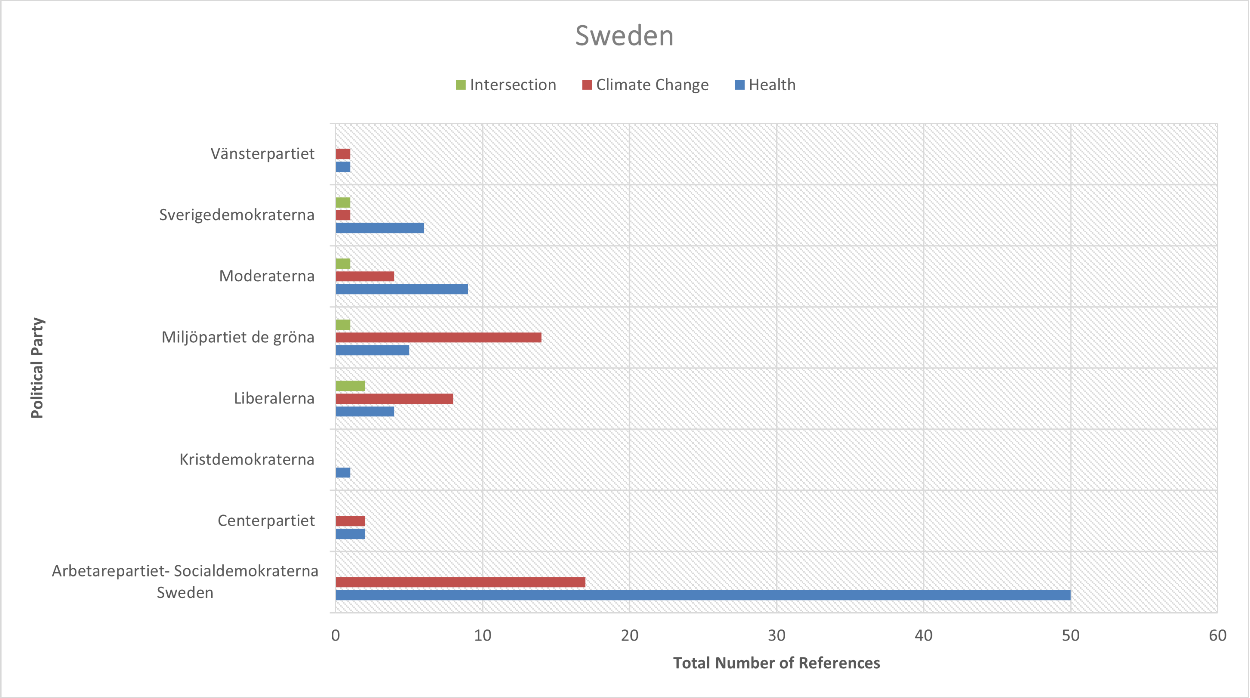
Sweden
Intersection
Climate Change
Health
Vänsterpartiet
Sverigedemokraterna
Moderaterna
Miljöpartiet de gröna
Liberalerna
Kristdemokraterna
Centerpartiet
Arbetarepartiet- Socialdemokraterna Sweden
Political Party
0
10
20
30
40
50
60
Total Number of References

Additional Figures

The figures in this section break down references to health, climate change, and the intersection of health and climate change between 2014 and 2021, by EEA country.

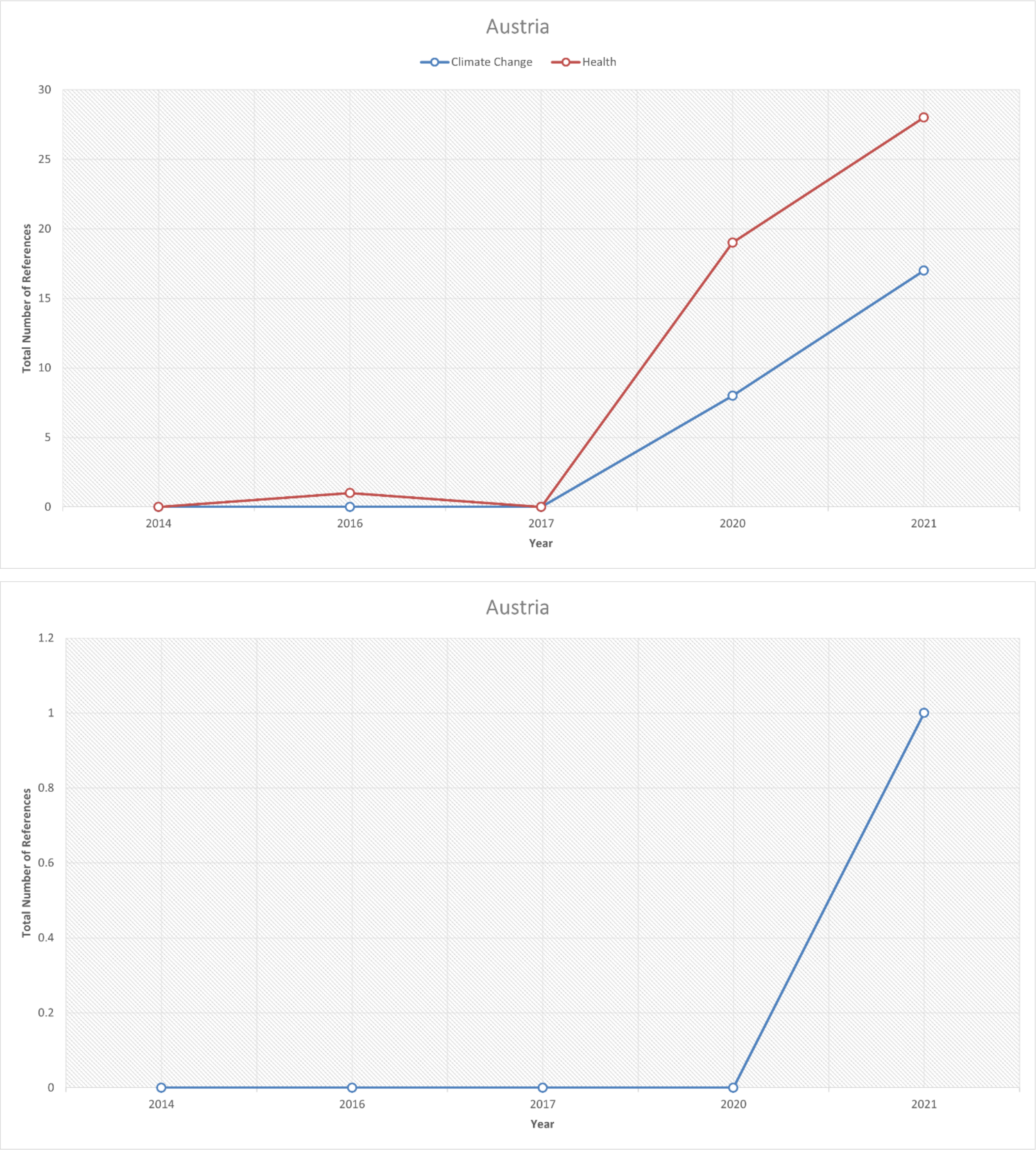

Figure 5.17 Total number of references to the intersection of health and climate change.

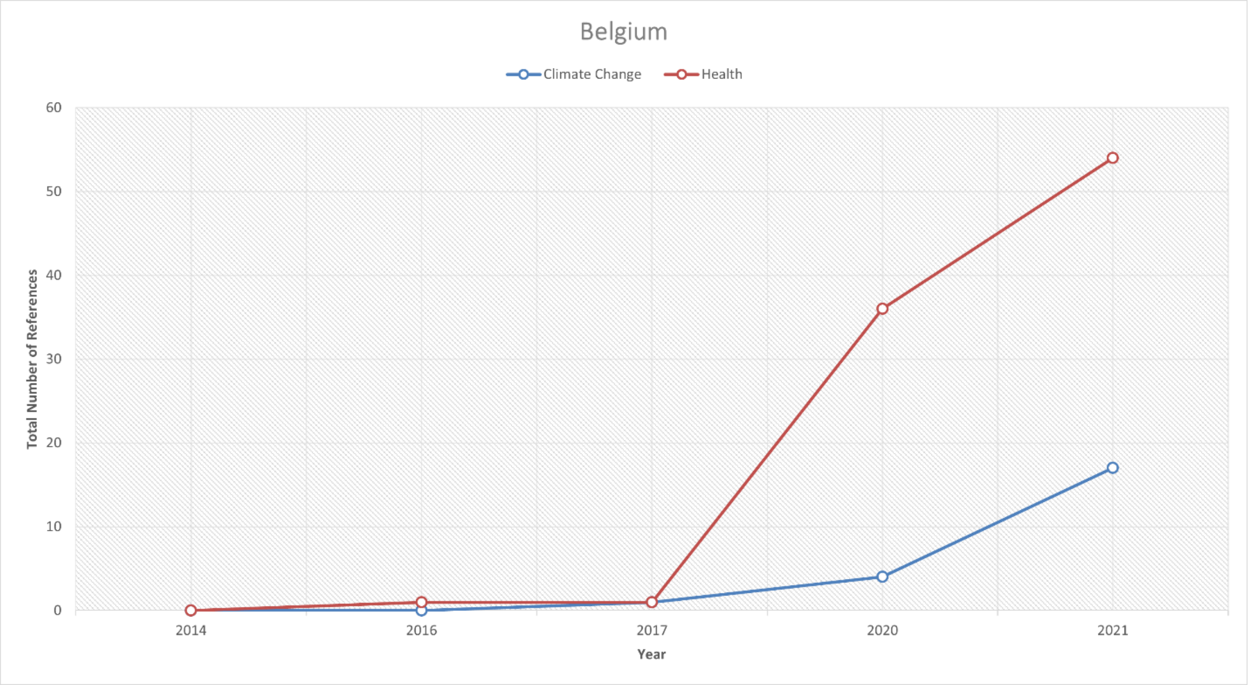

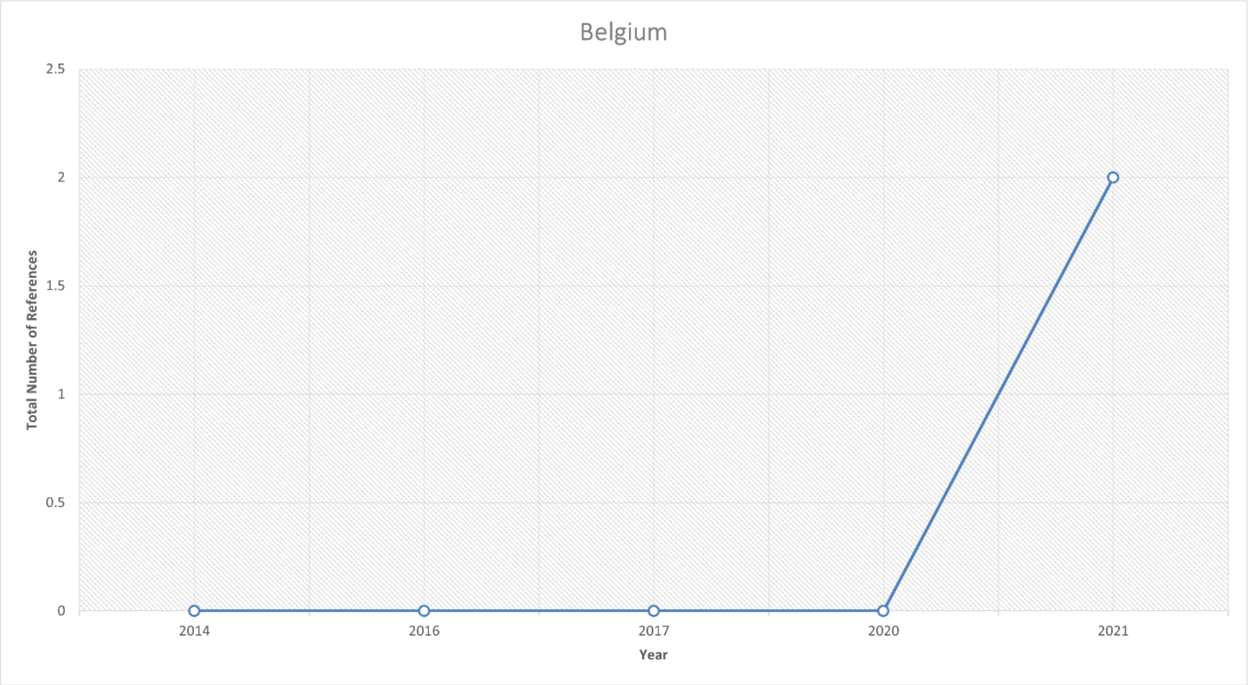

Figure 5.18 Total number of references to the intersection of health and climate change.

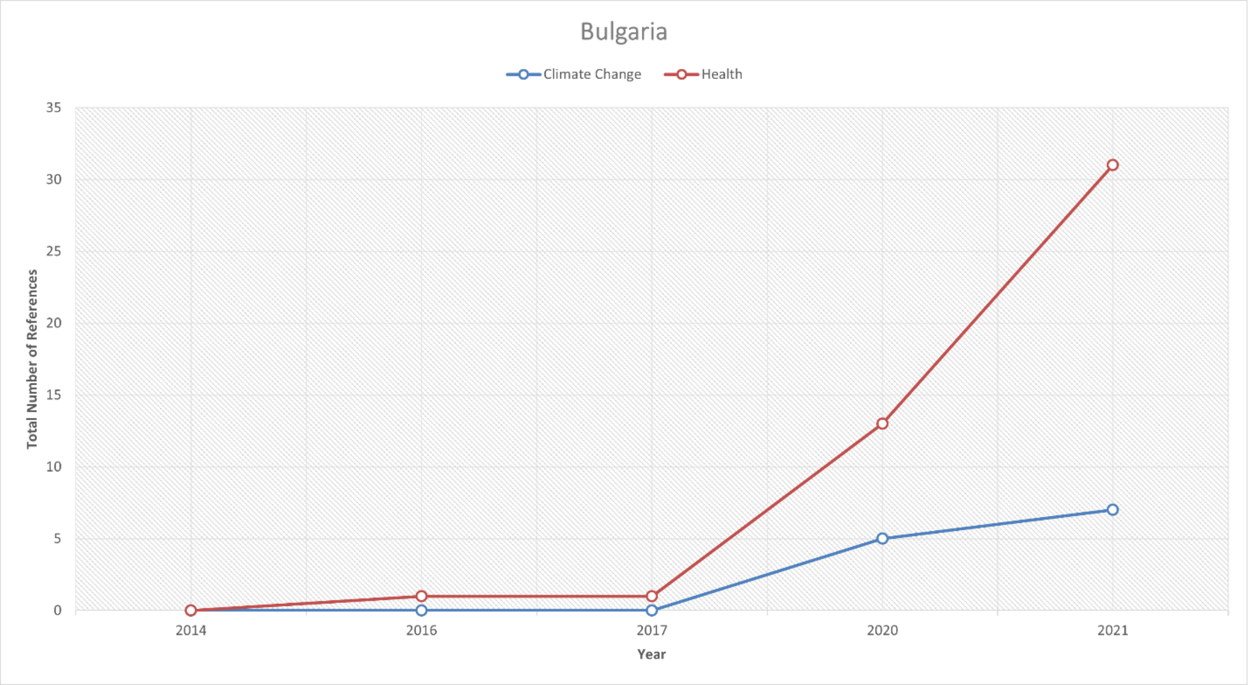

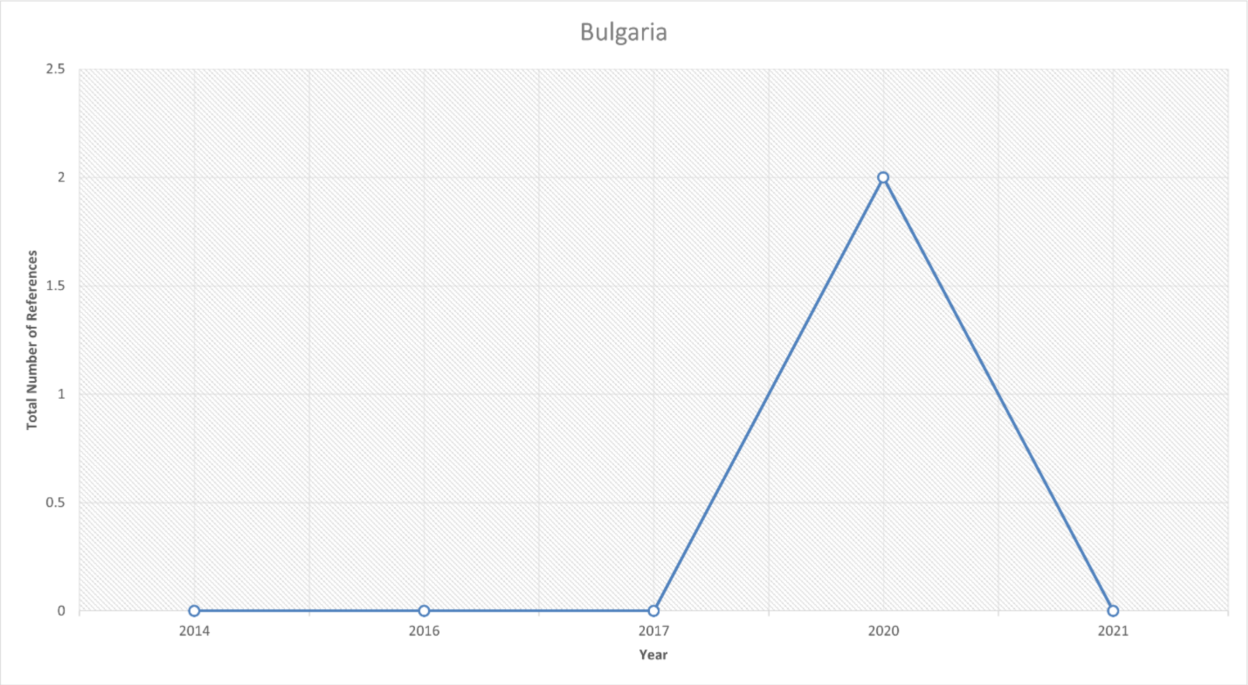

Figure 5.19 Total number of references to the intersection of health and climate change.

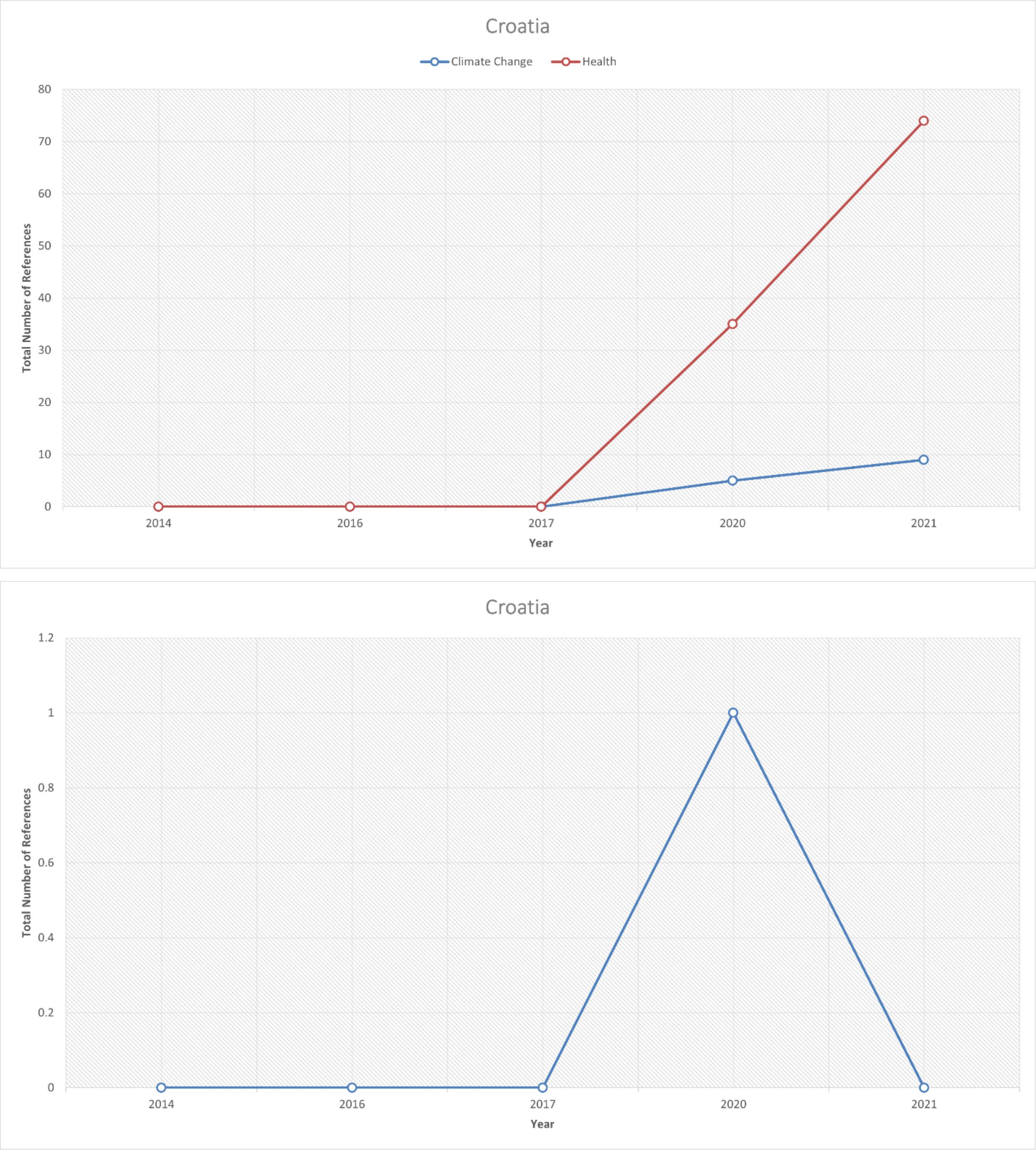

Figure 5.20 Total number of references to the intersection of health and climate change.

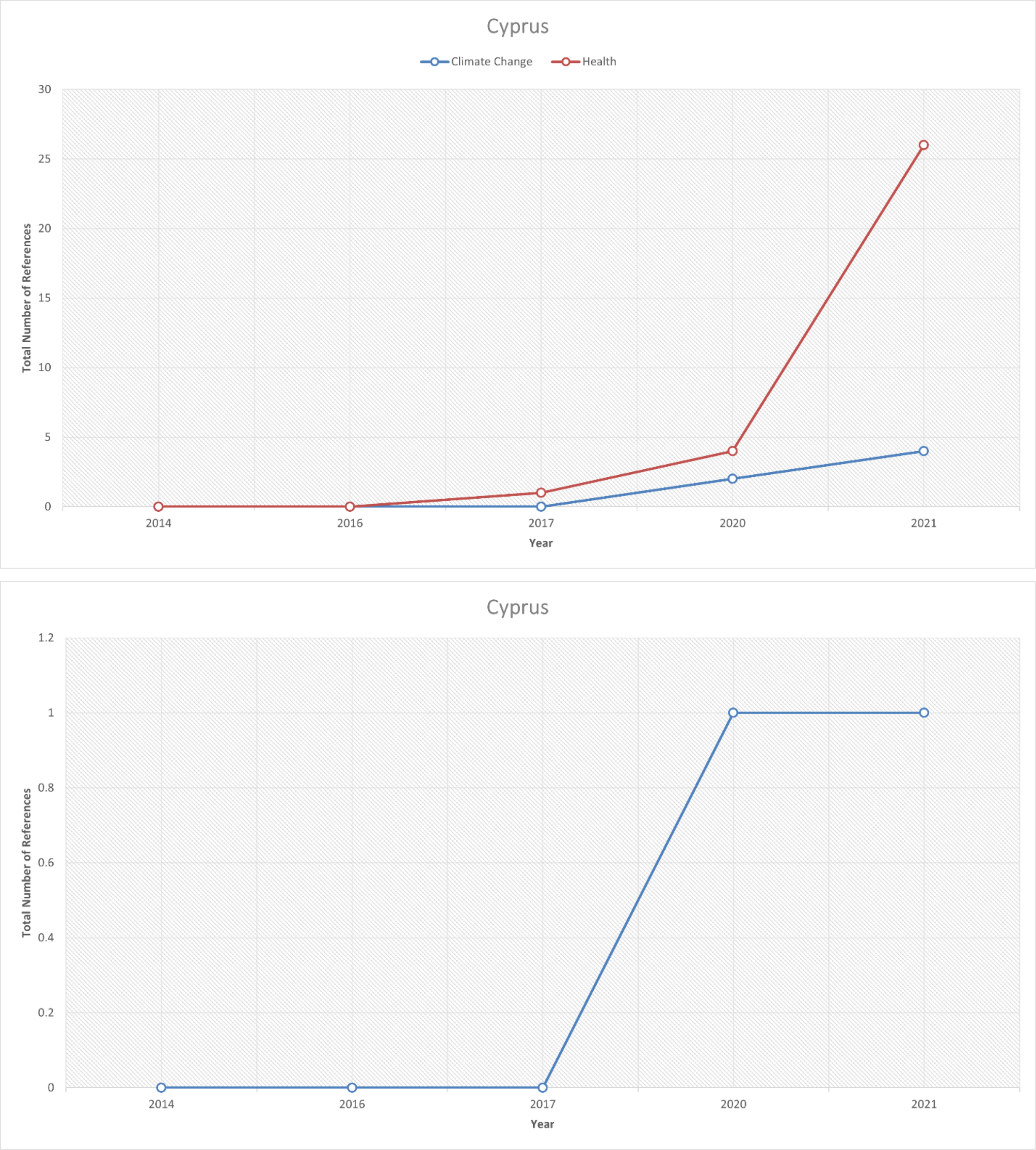

Figure 5.21 Total number of references to the intersection of health and climate change.

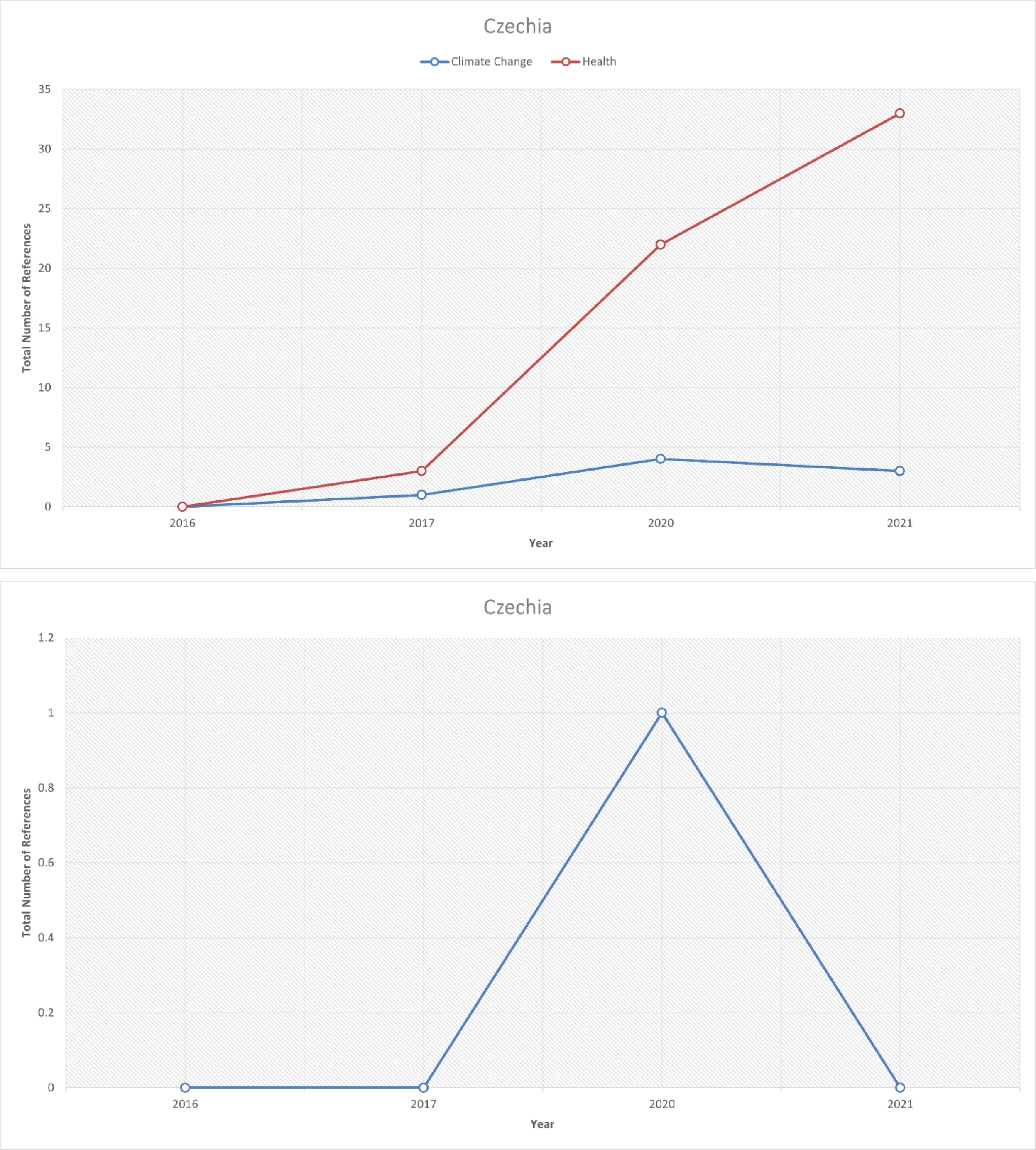

Figure 5.22 Total number of references to the intersection of health and climate change.

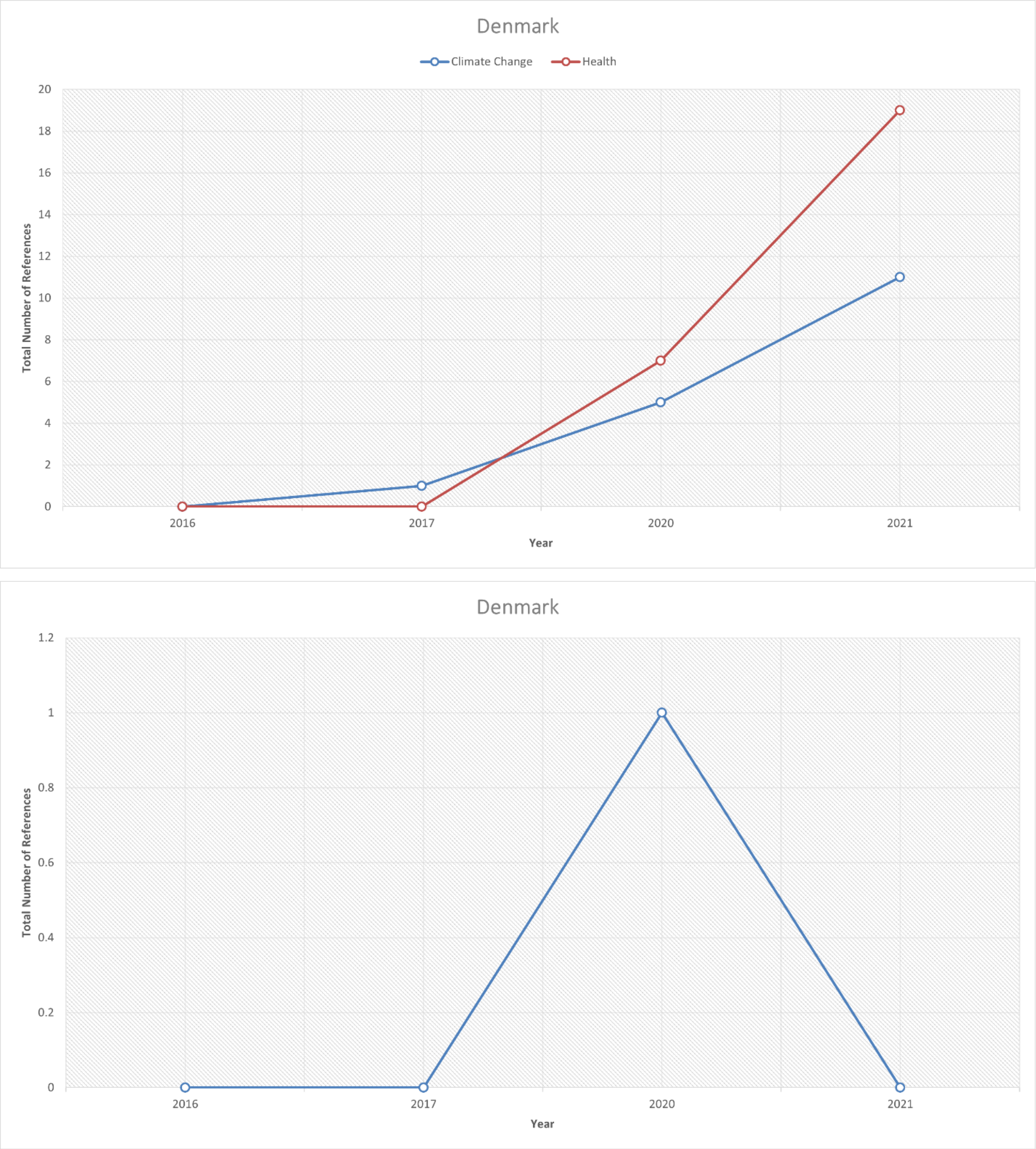

Figure 5.23 Total number of references to the intersection of health and climate change.

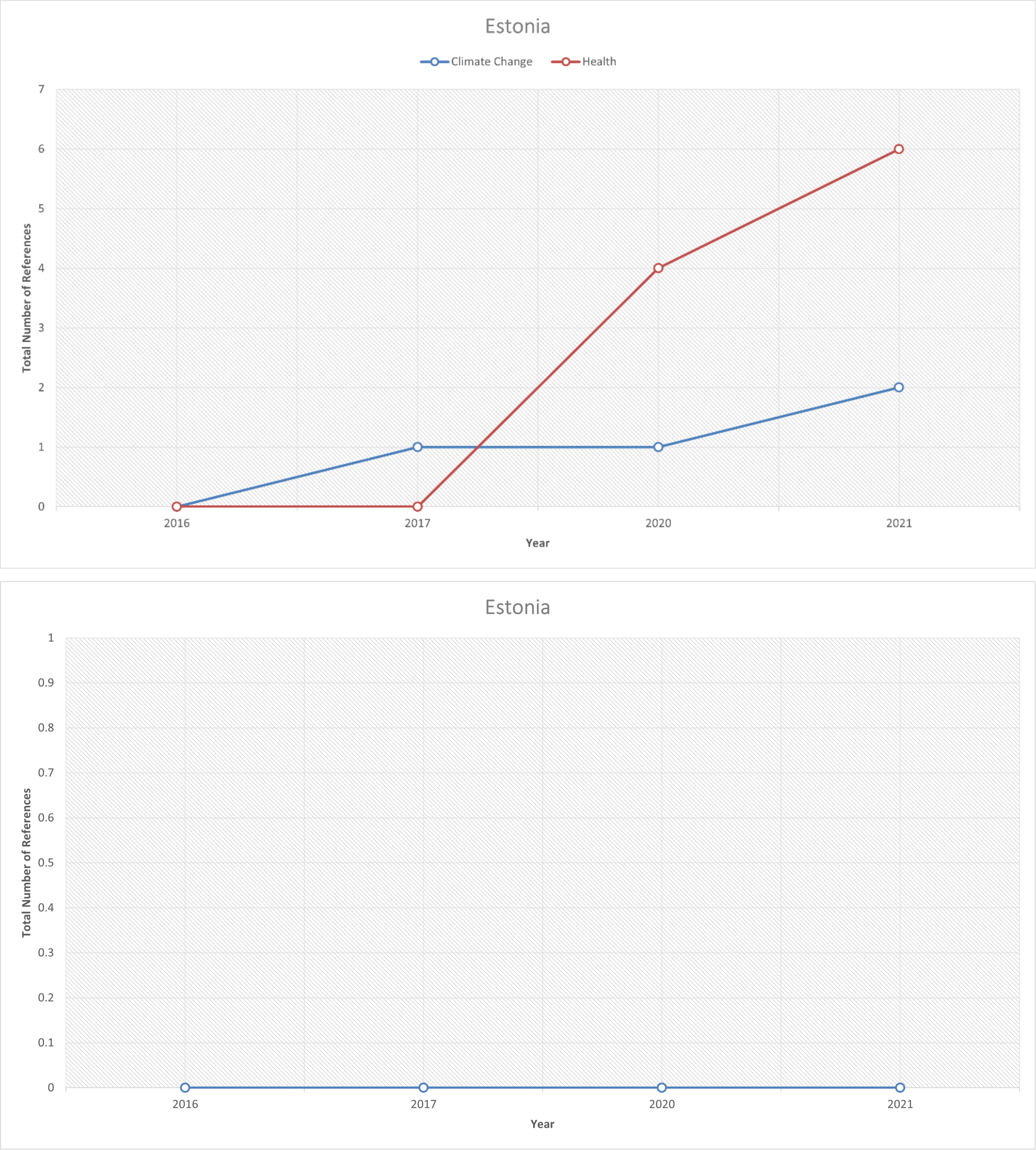

Figure 5.24 Total number of references to the intersection of health and climate change.

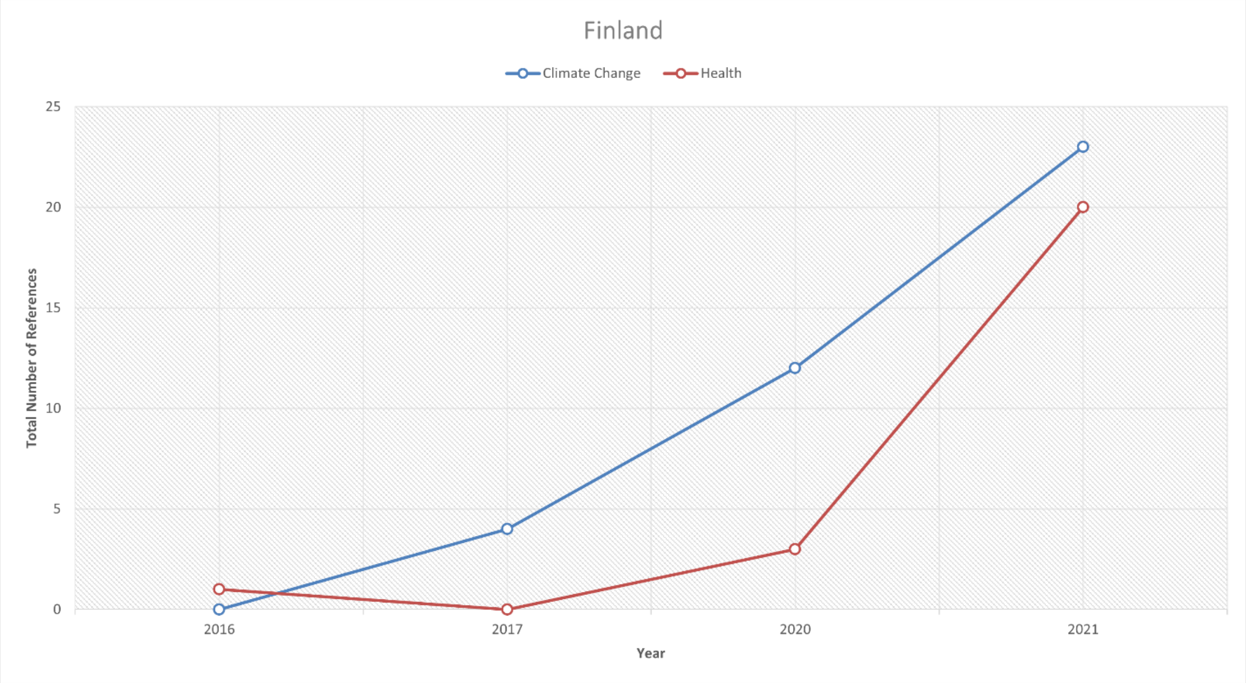

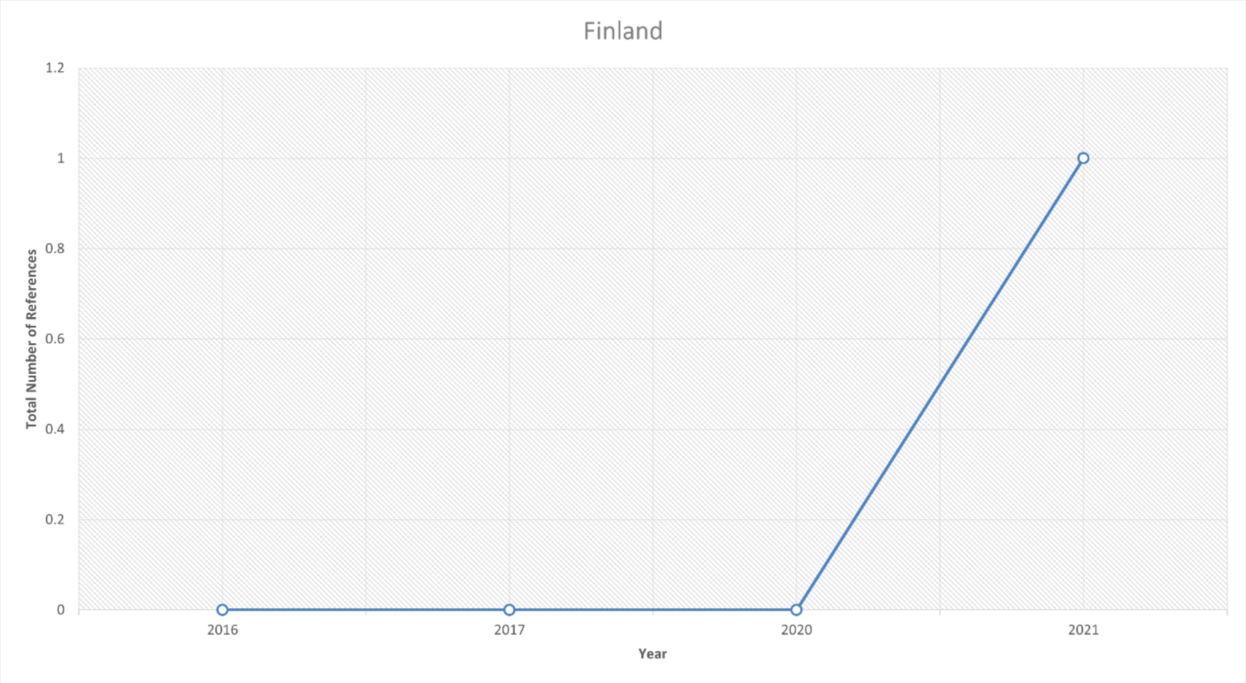

Figure 5.26 Total number of references to the intersection of health and climate change.

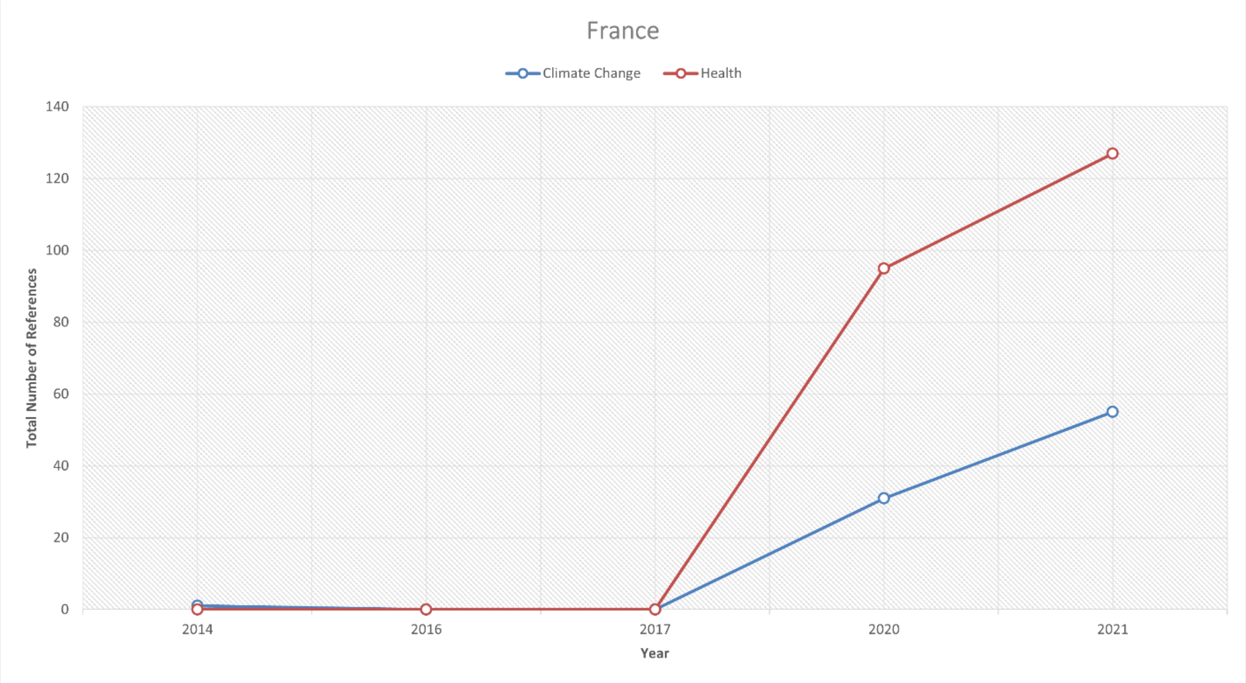

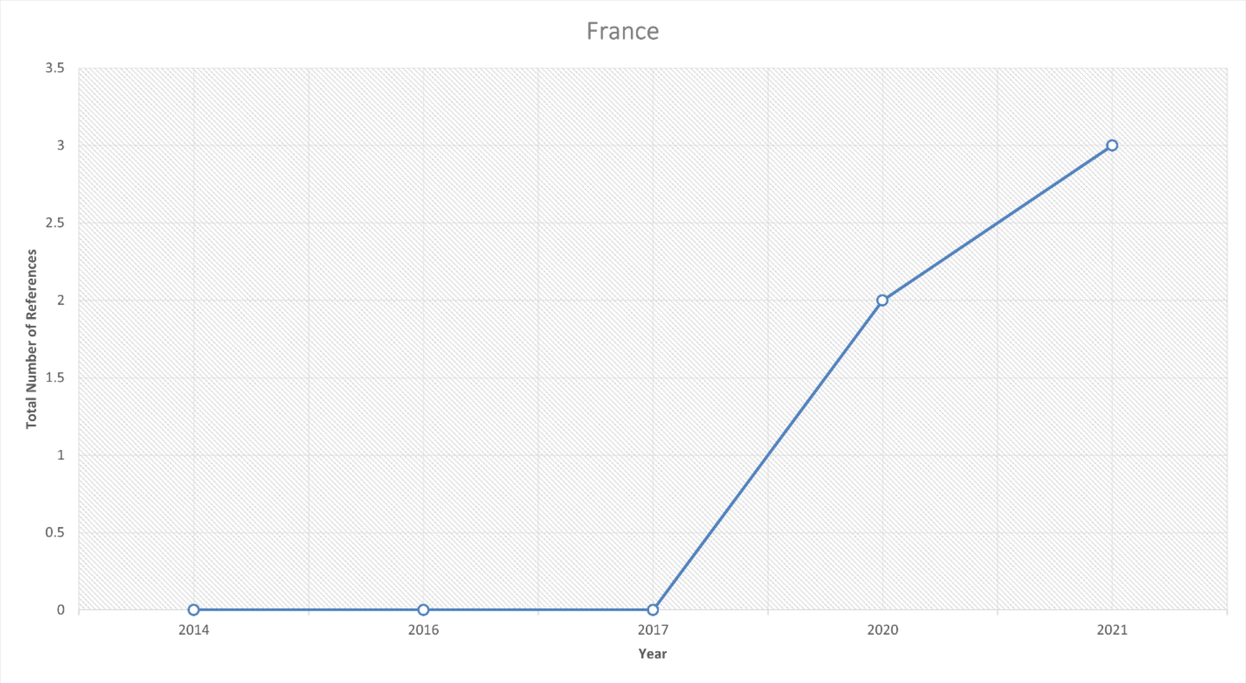

Figure 5.27 Total number of references to the intersection of health and climate change.

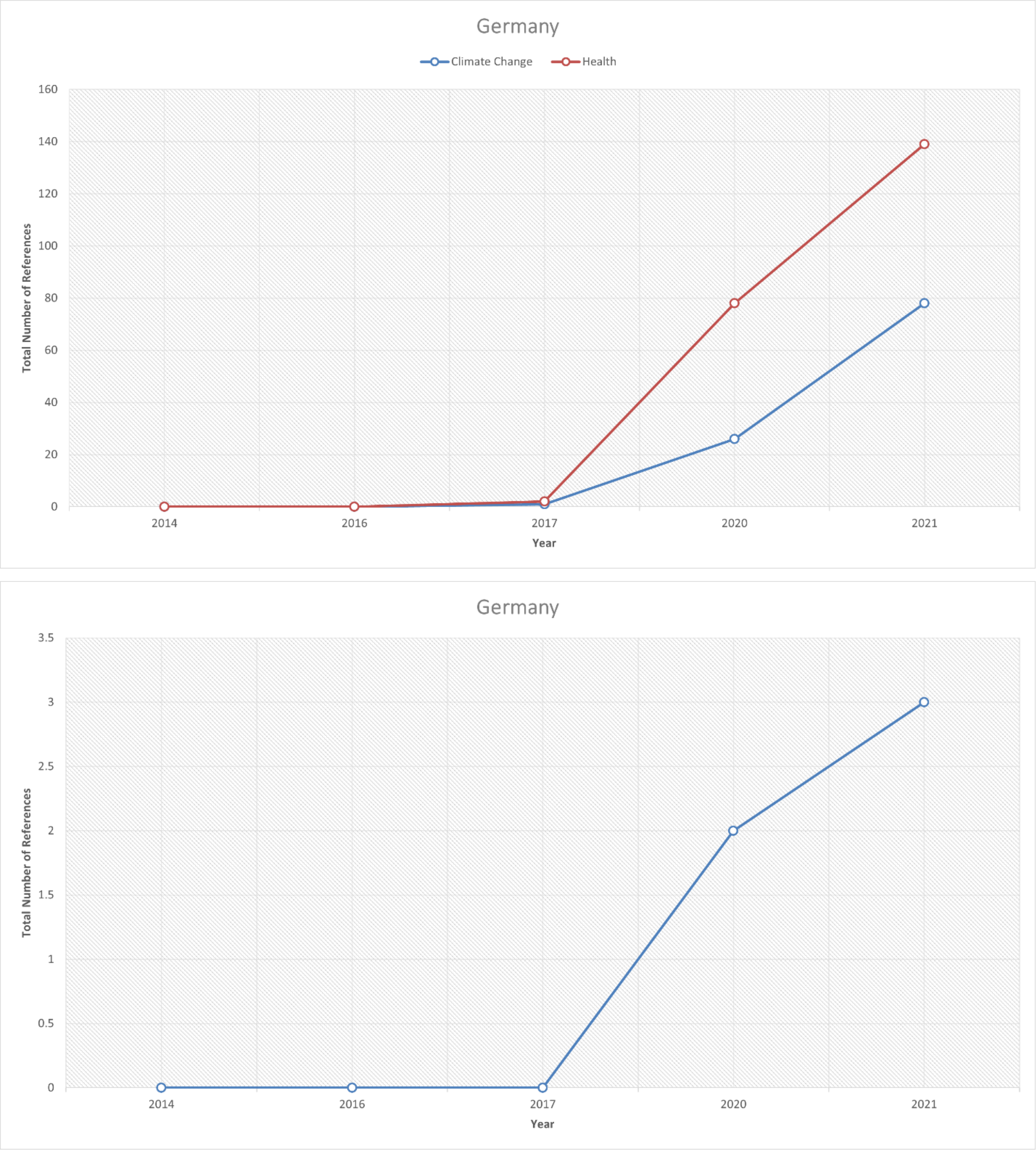

Figure 5.28 Total number of references to the intersection of health and climate change.

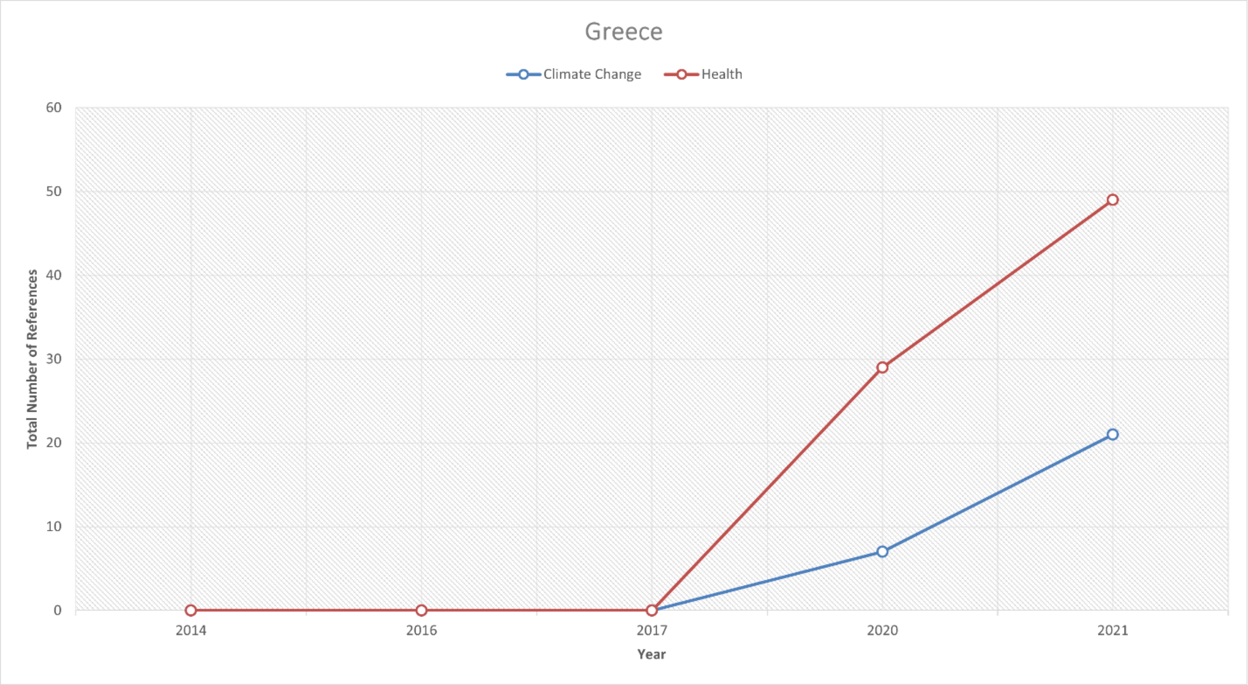

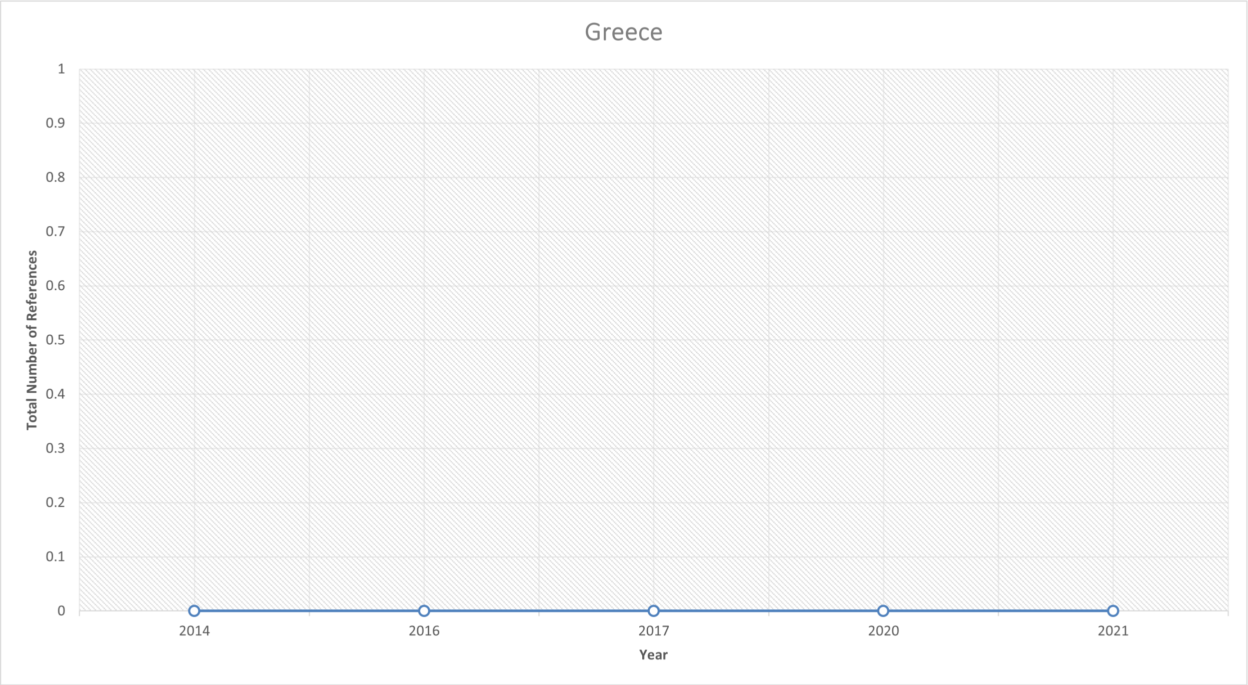

Figure 5.29 Total number of references to the intersection of health and climate change.

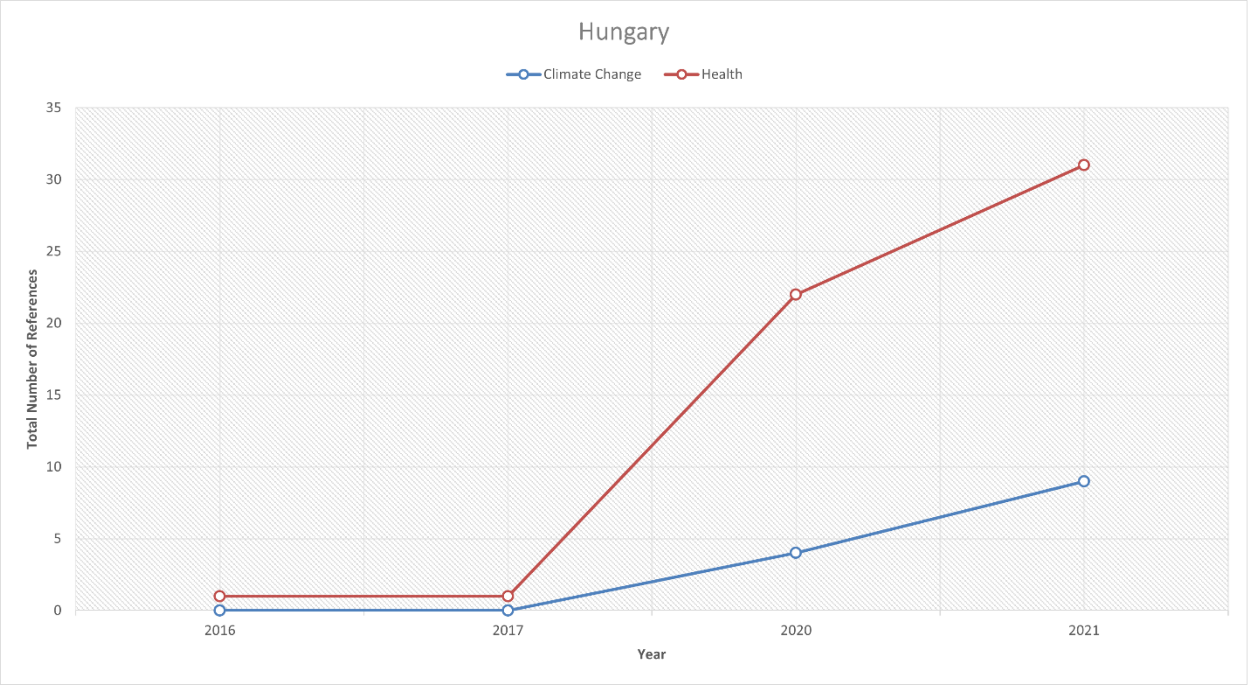

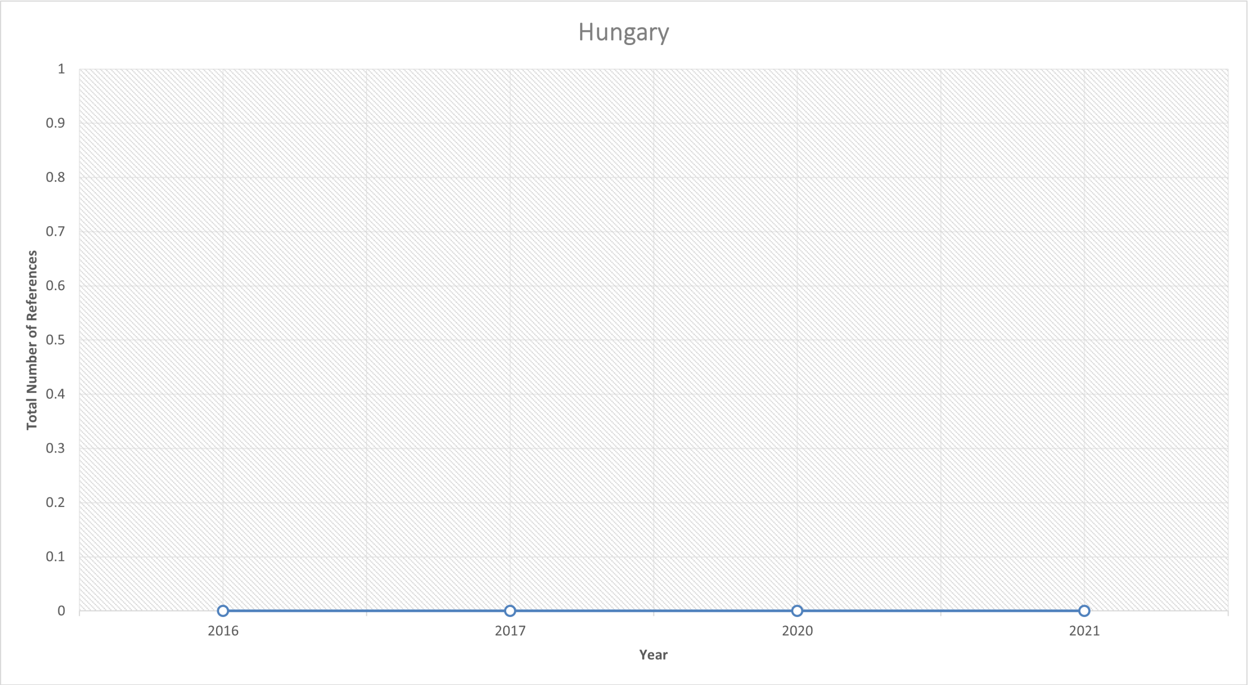

Figure 5.30 Total number of references to the intersection of health and climate change.

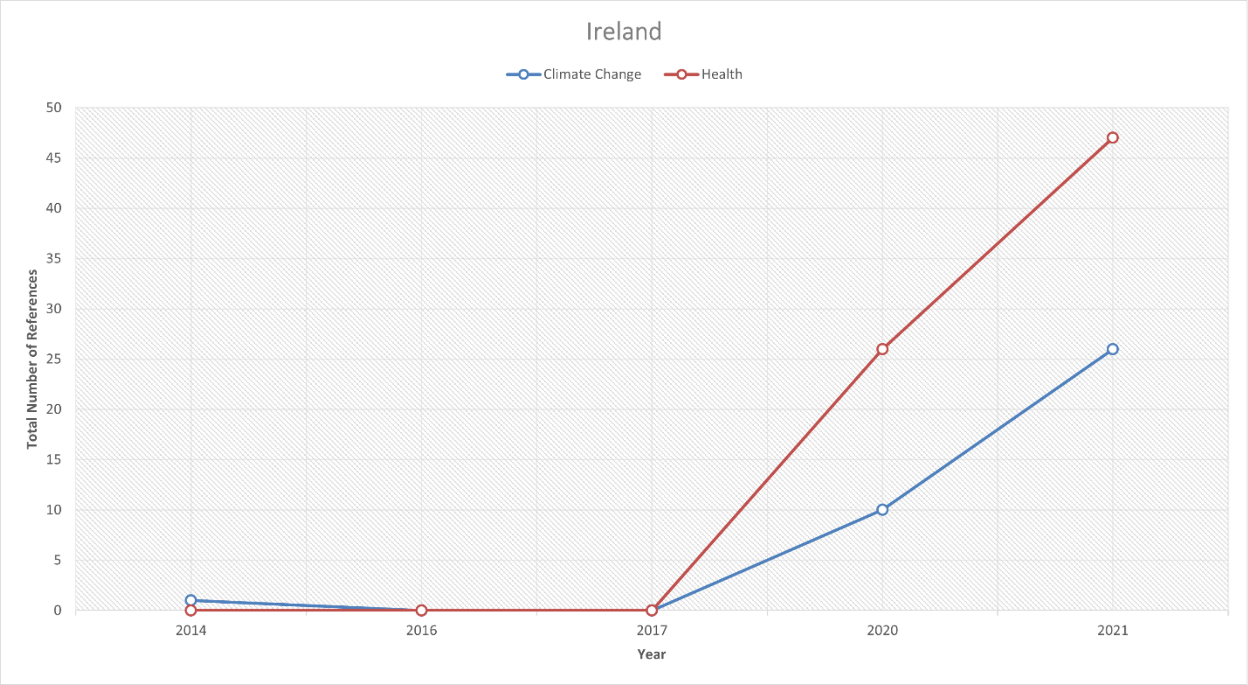

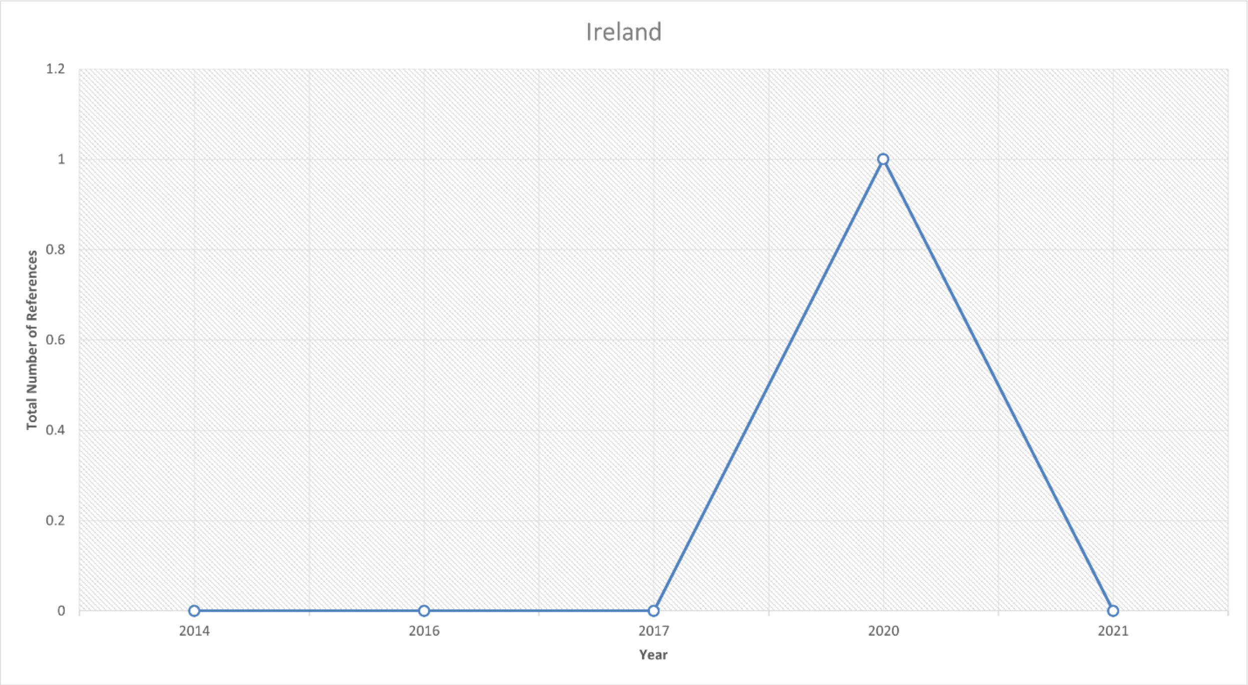

Figure 5.31 Total number of references to the intersection of health and climate change.

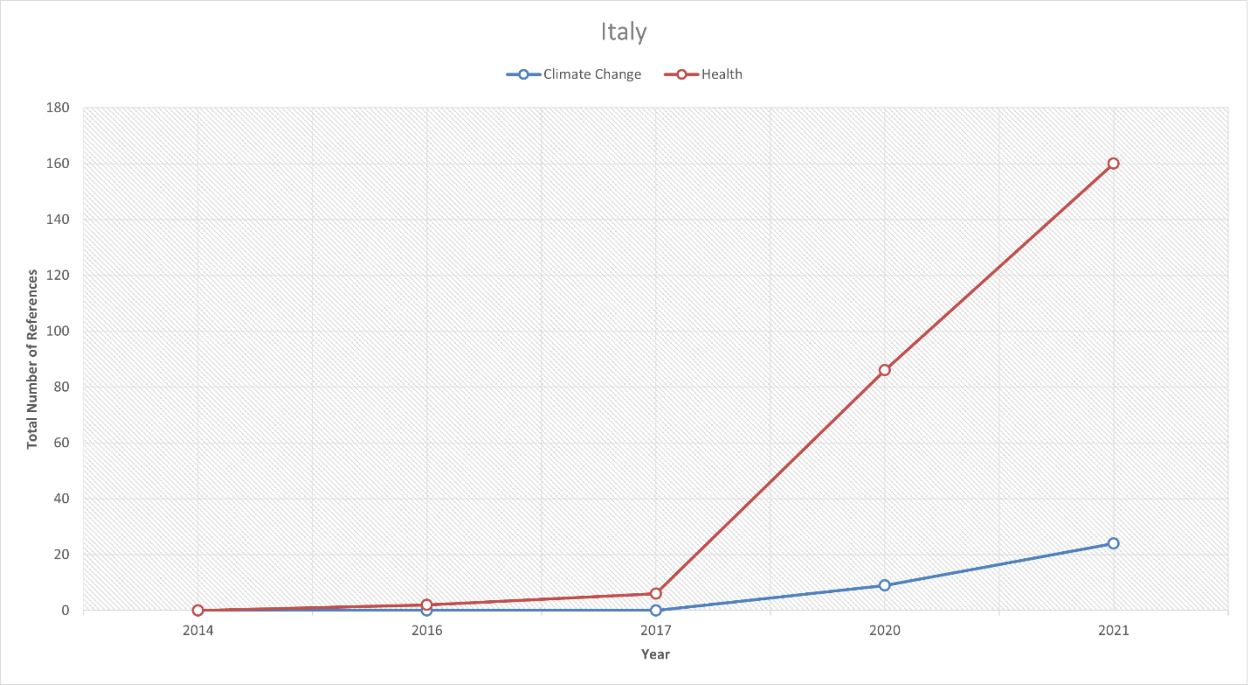

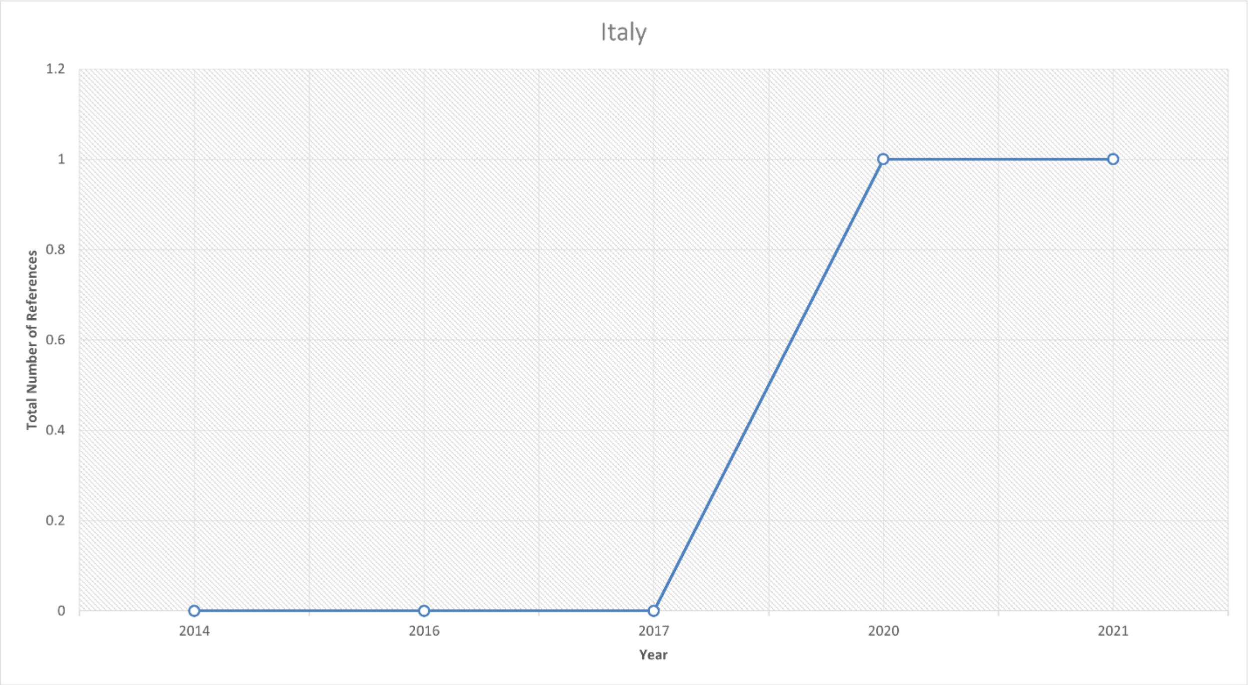

Figure 5.32 Total number of references to the intersection of health and climate change.

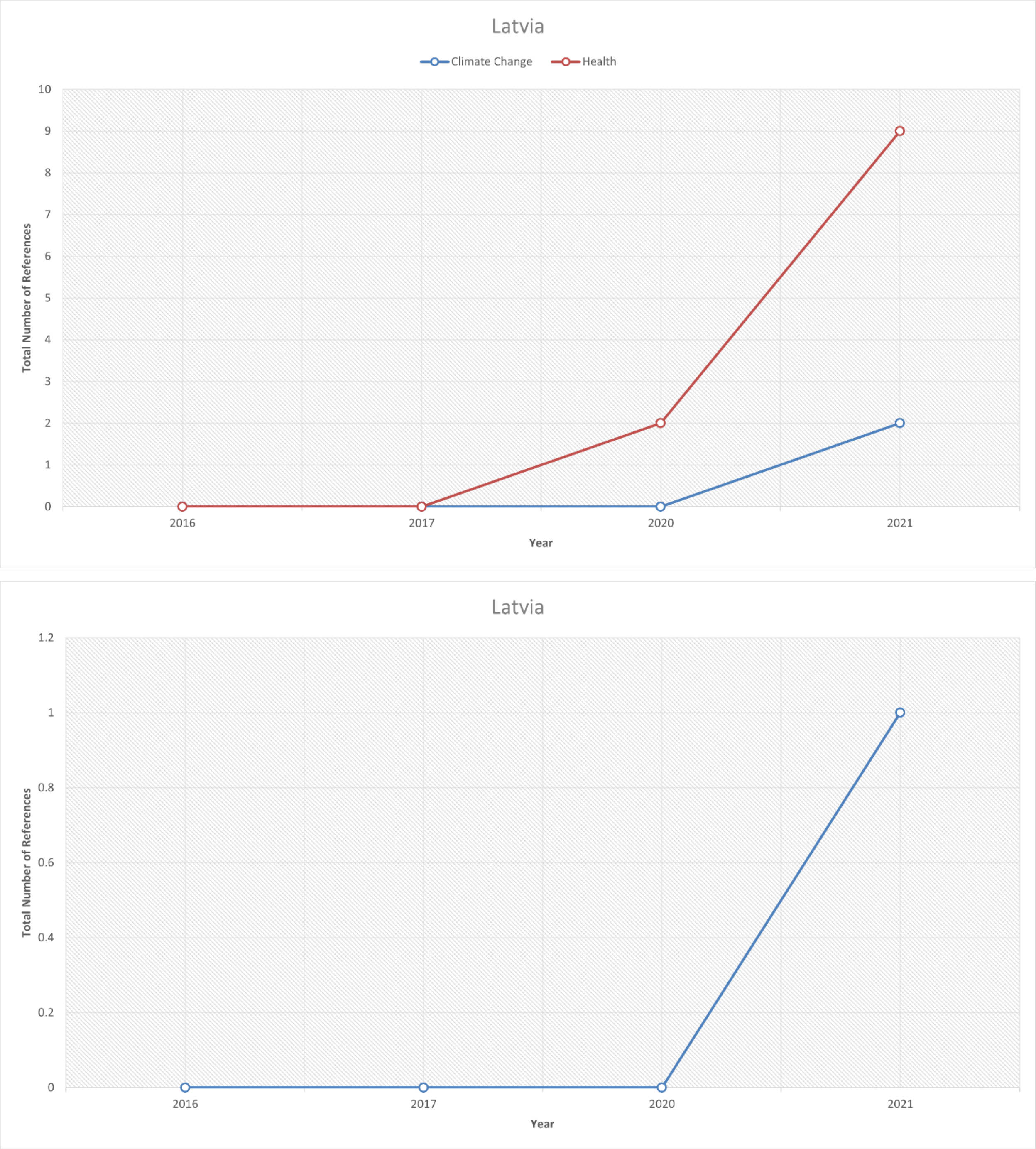

Figure 5.33 Total number of references to the intersection of health and climate change.

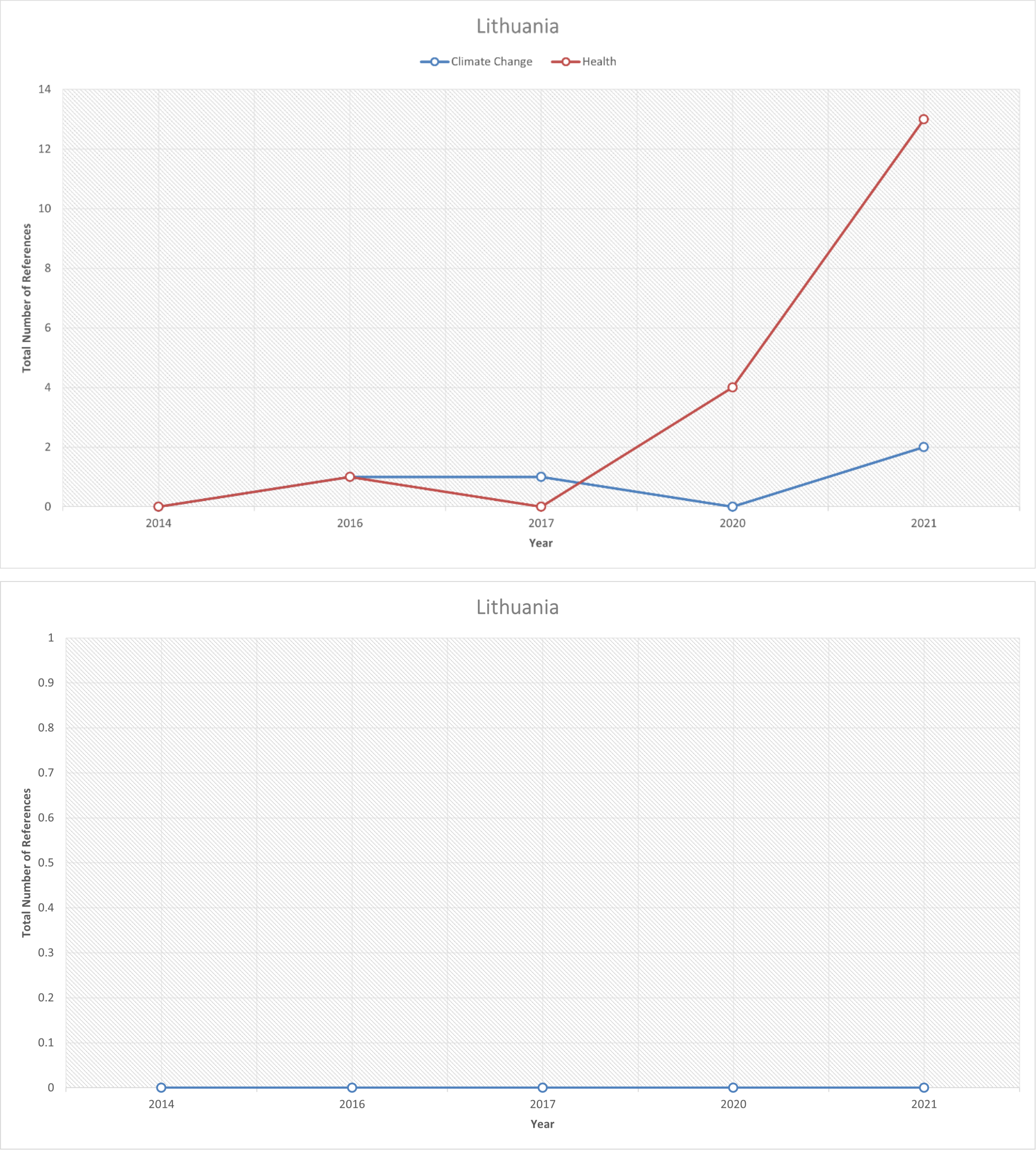

Figure 5.34 Total number of references to the intersection of health and climate change.

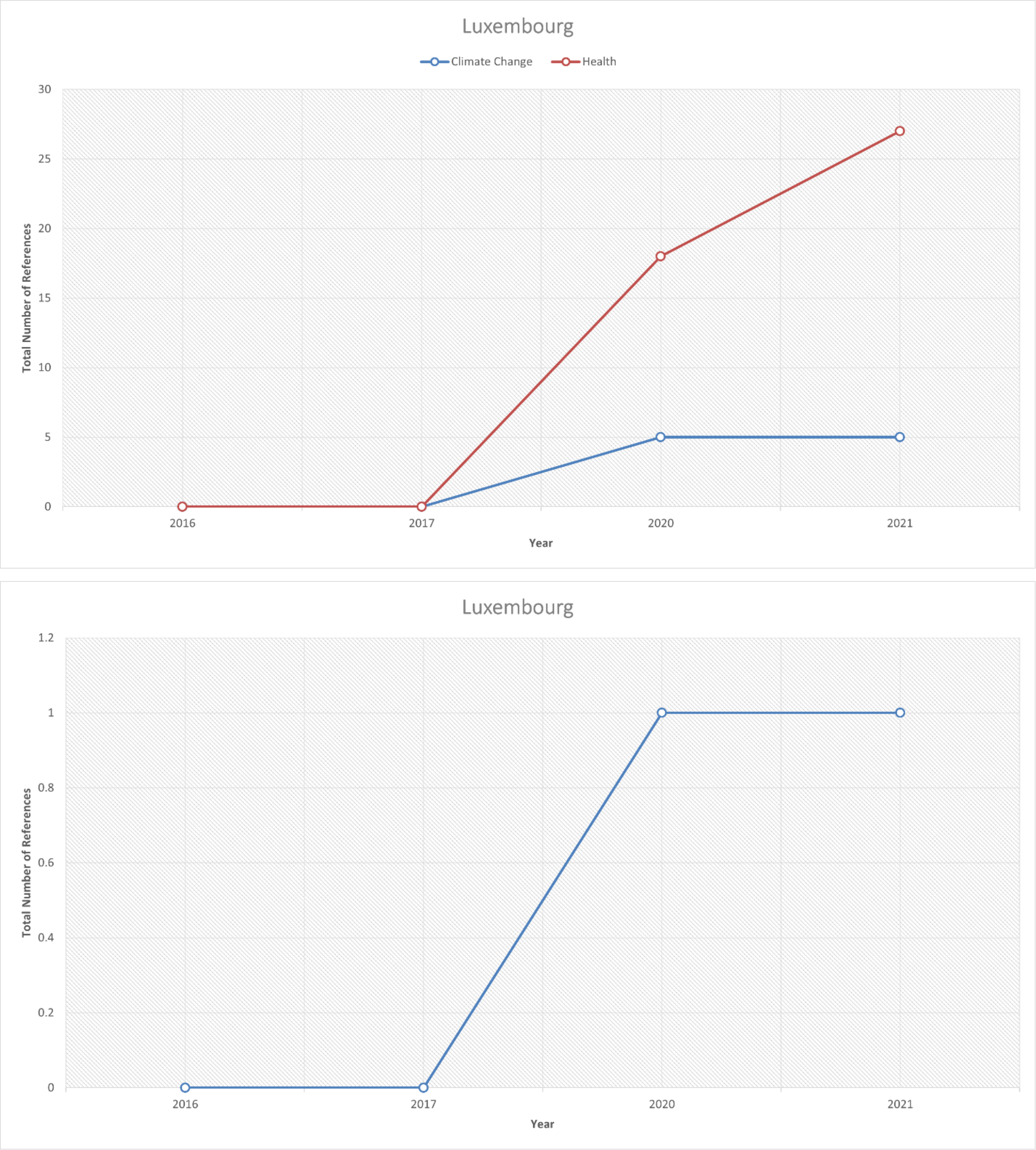

Figure 5.35 Total number of references to the intersection of health and climate change.

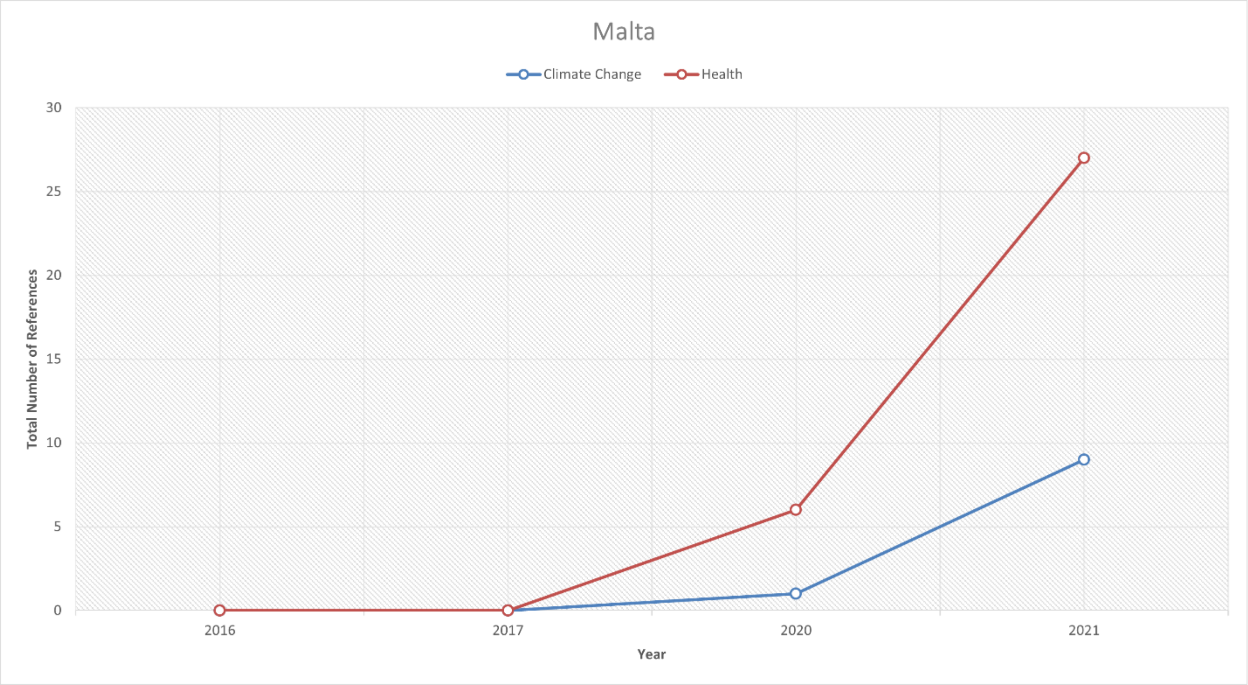

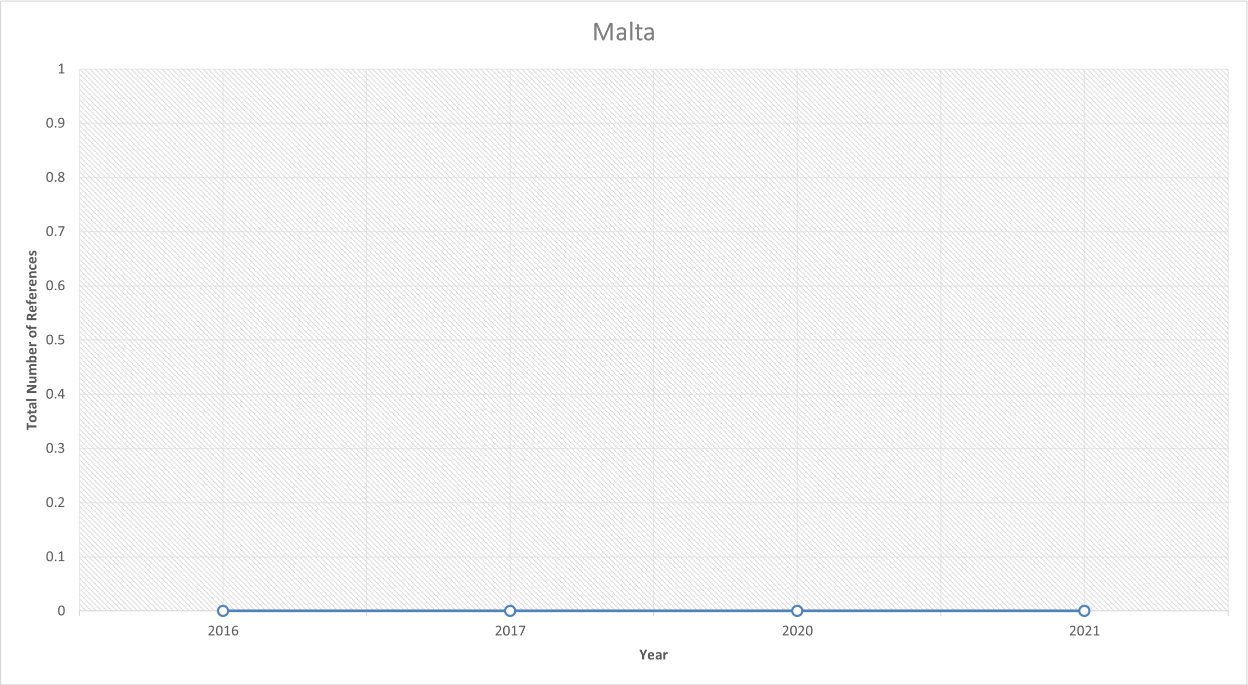

Figure 5.36 Total number of references to the intersection of health and climate change.

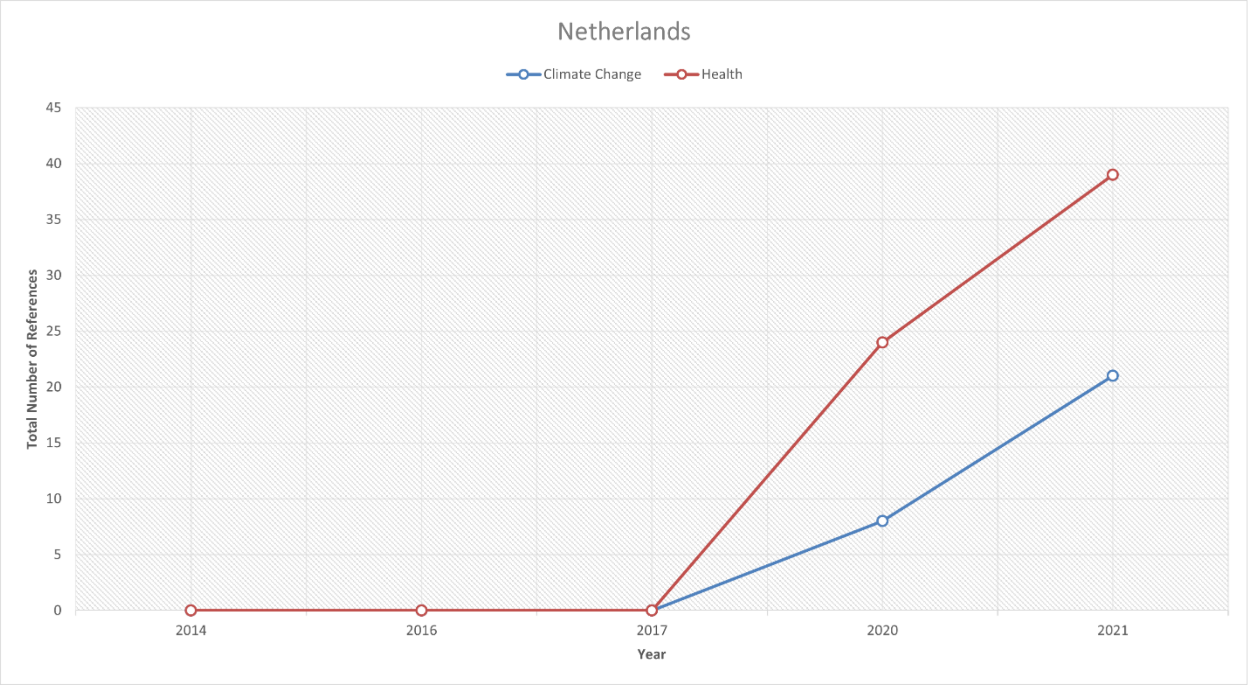

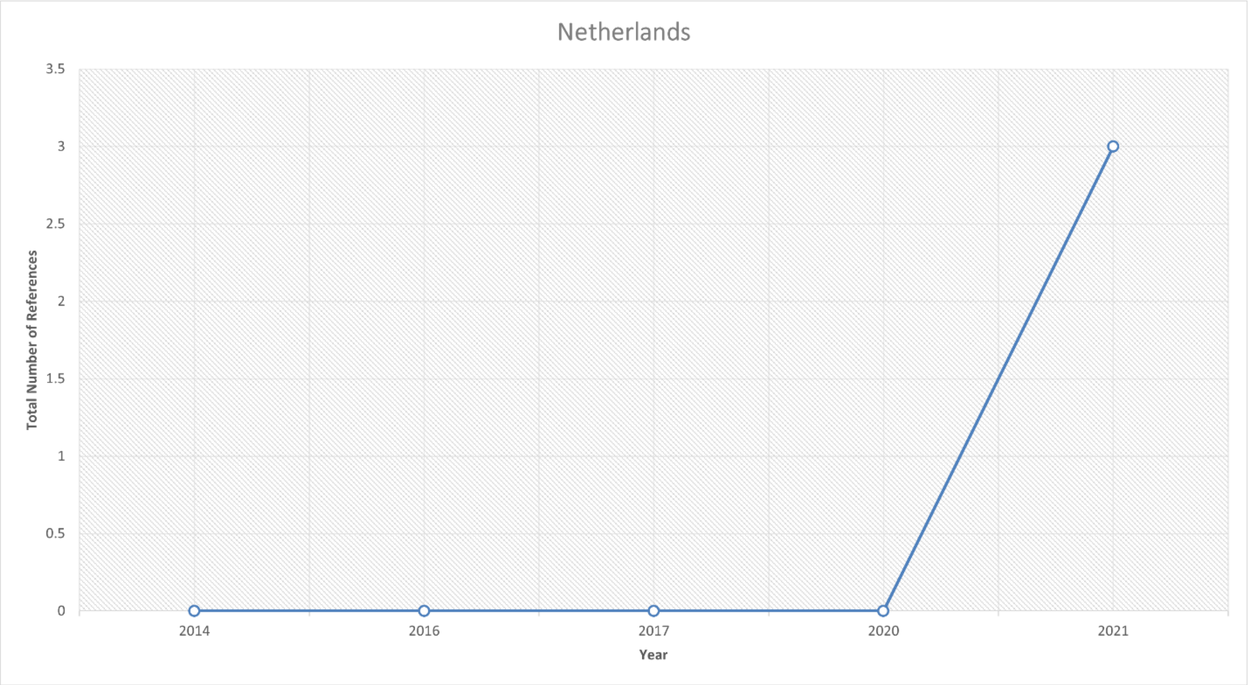

Figure 5.37 Total number of references to the intersection of health and climate change.

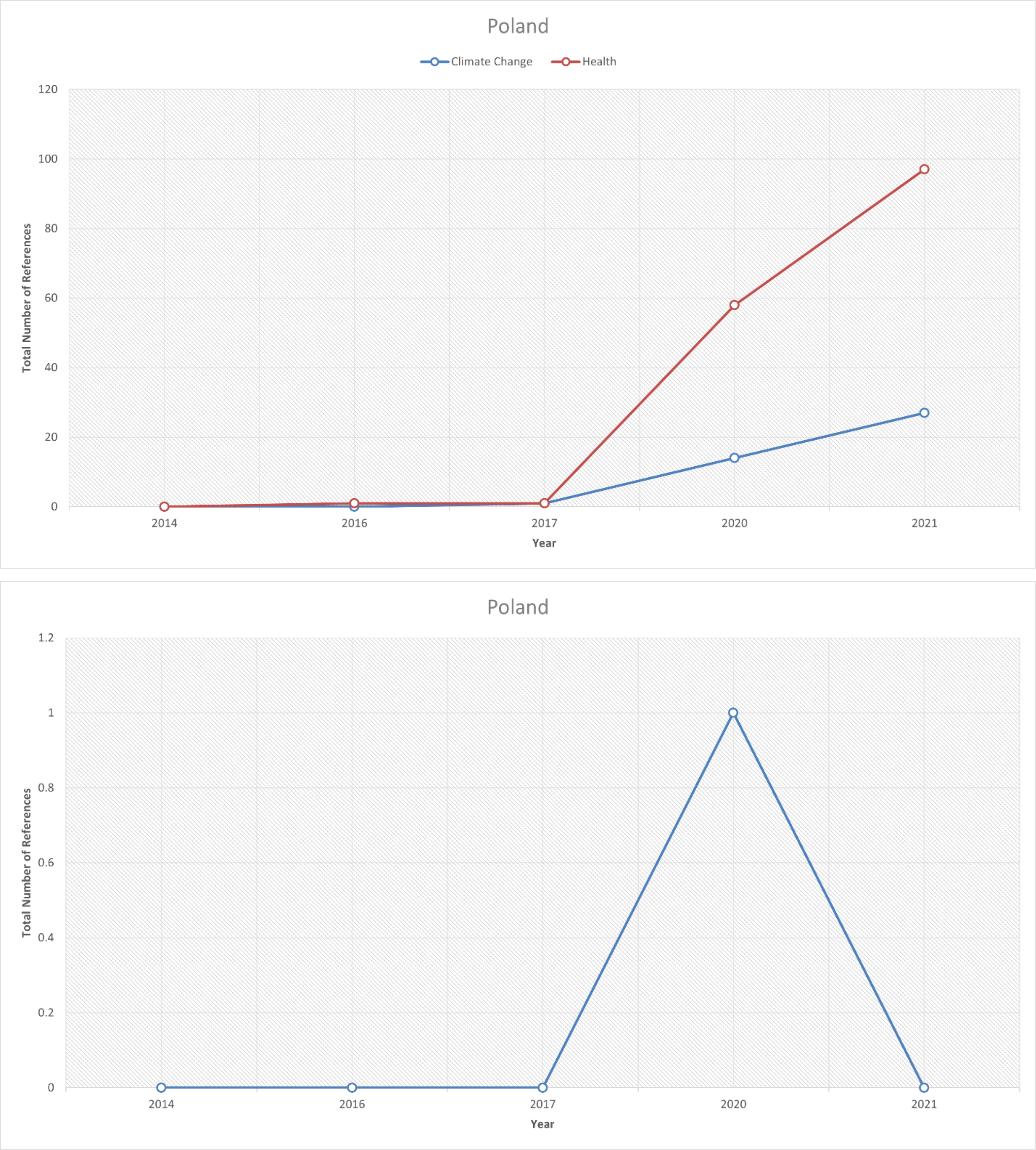

Figure 5.38 Total number of references to the intersection of health and climate change.

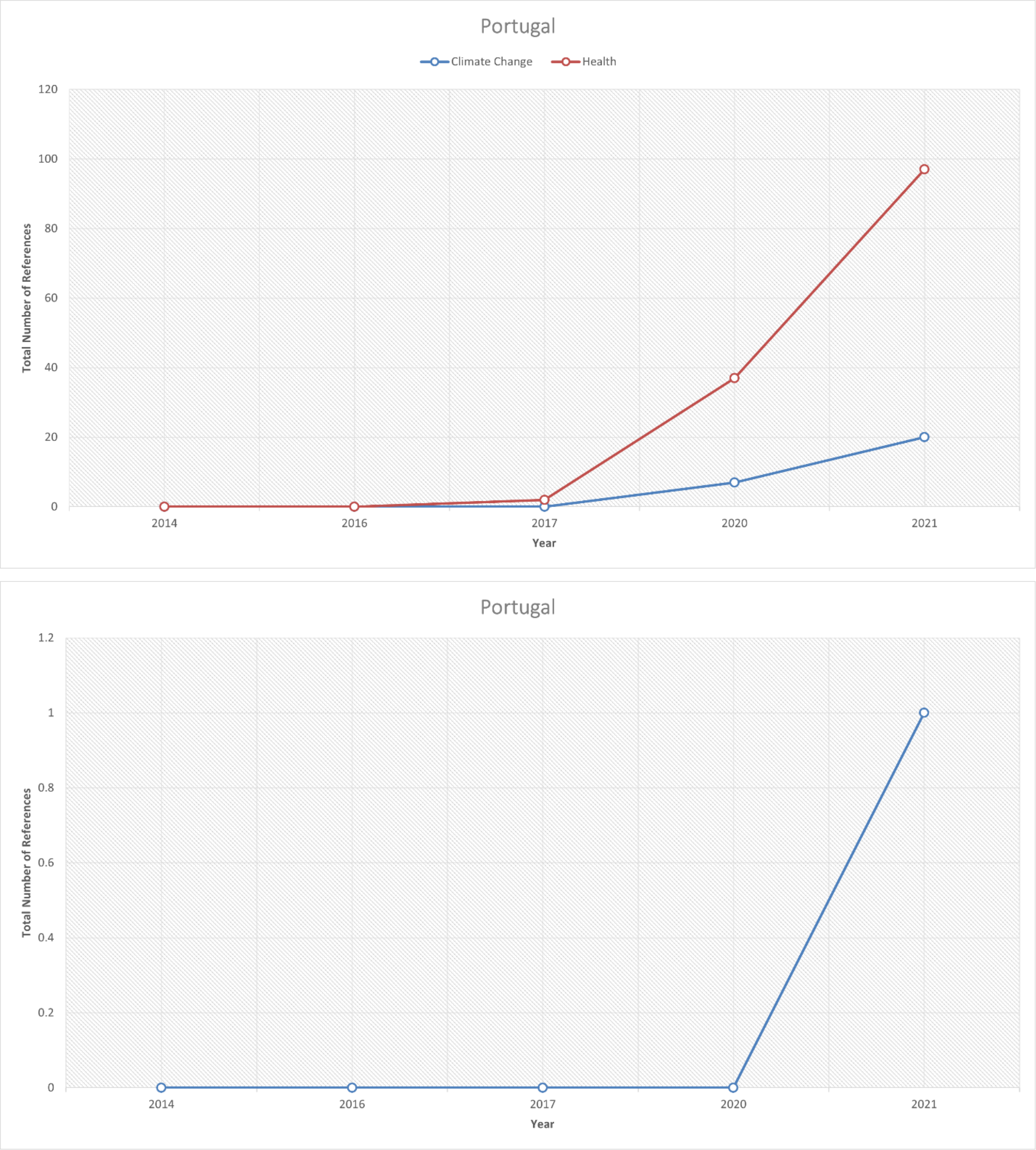

Figure 5.39 Total number of references to the intersection of health and climate change.

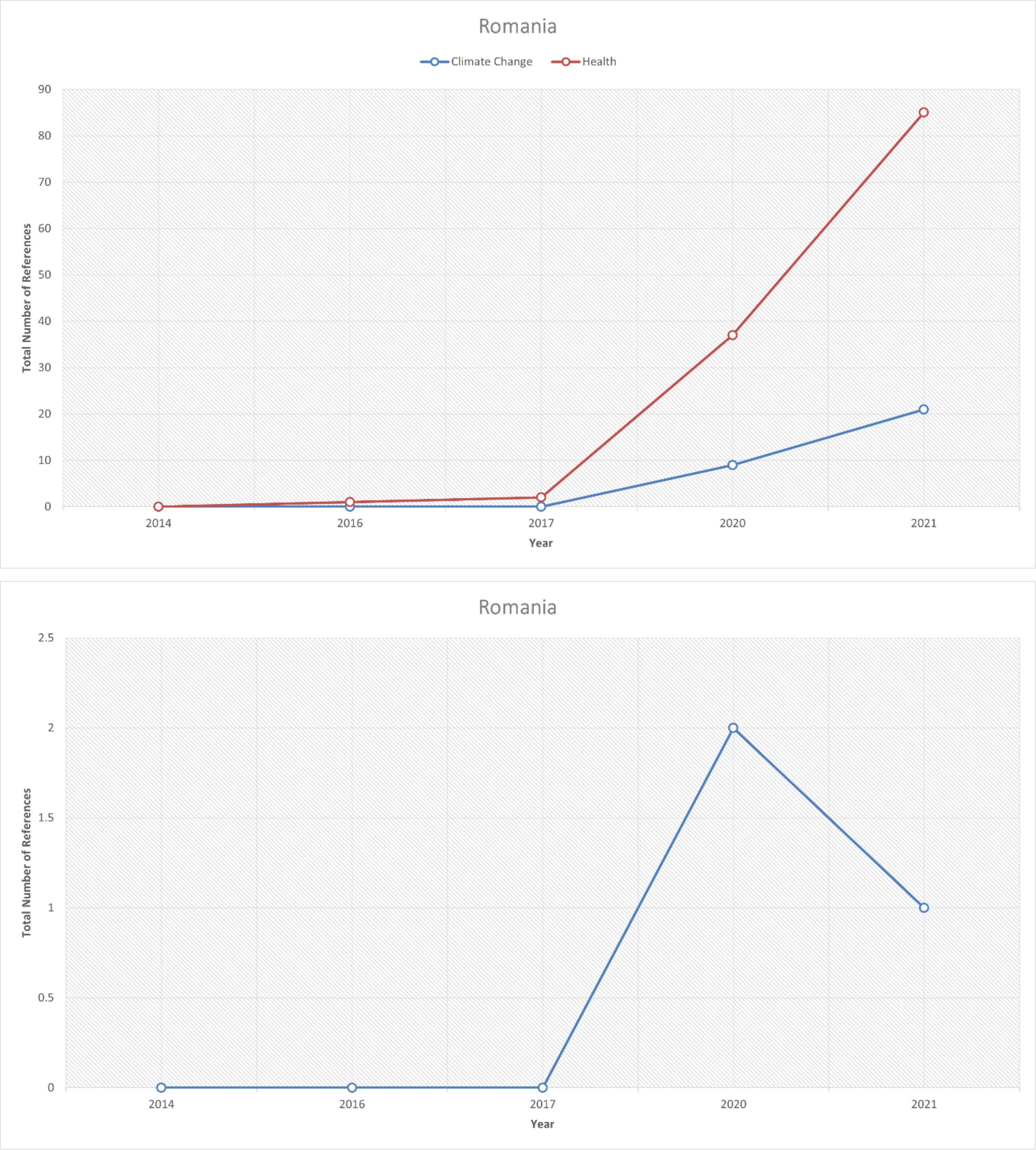

Figure 5.40 Total number of references to the intersection of health and climate change.

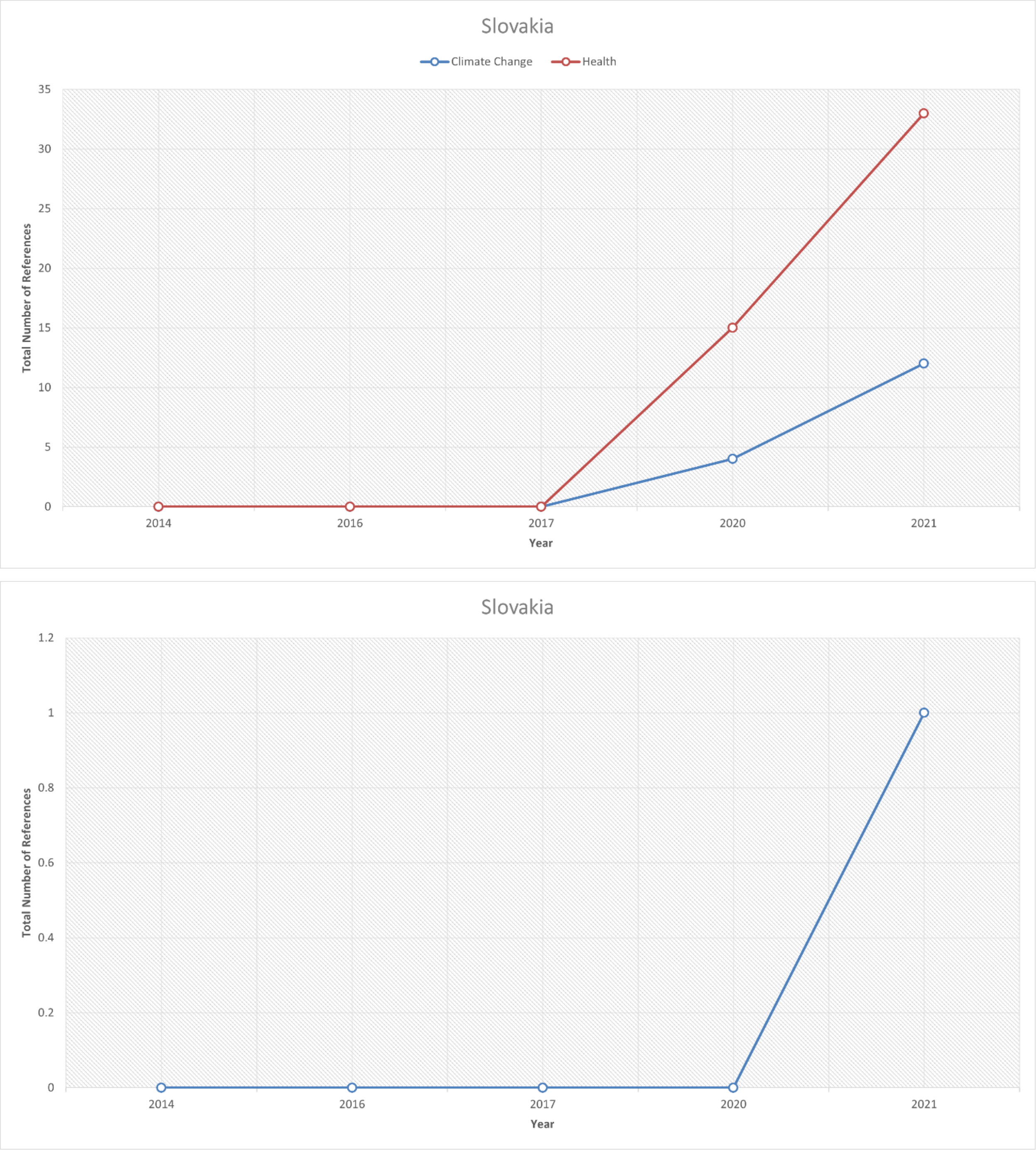

Figure 5.41 Total number of references to the intersection of health and climate change.

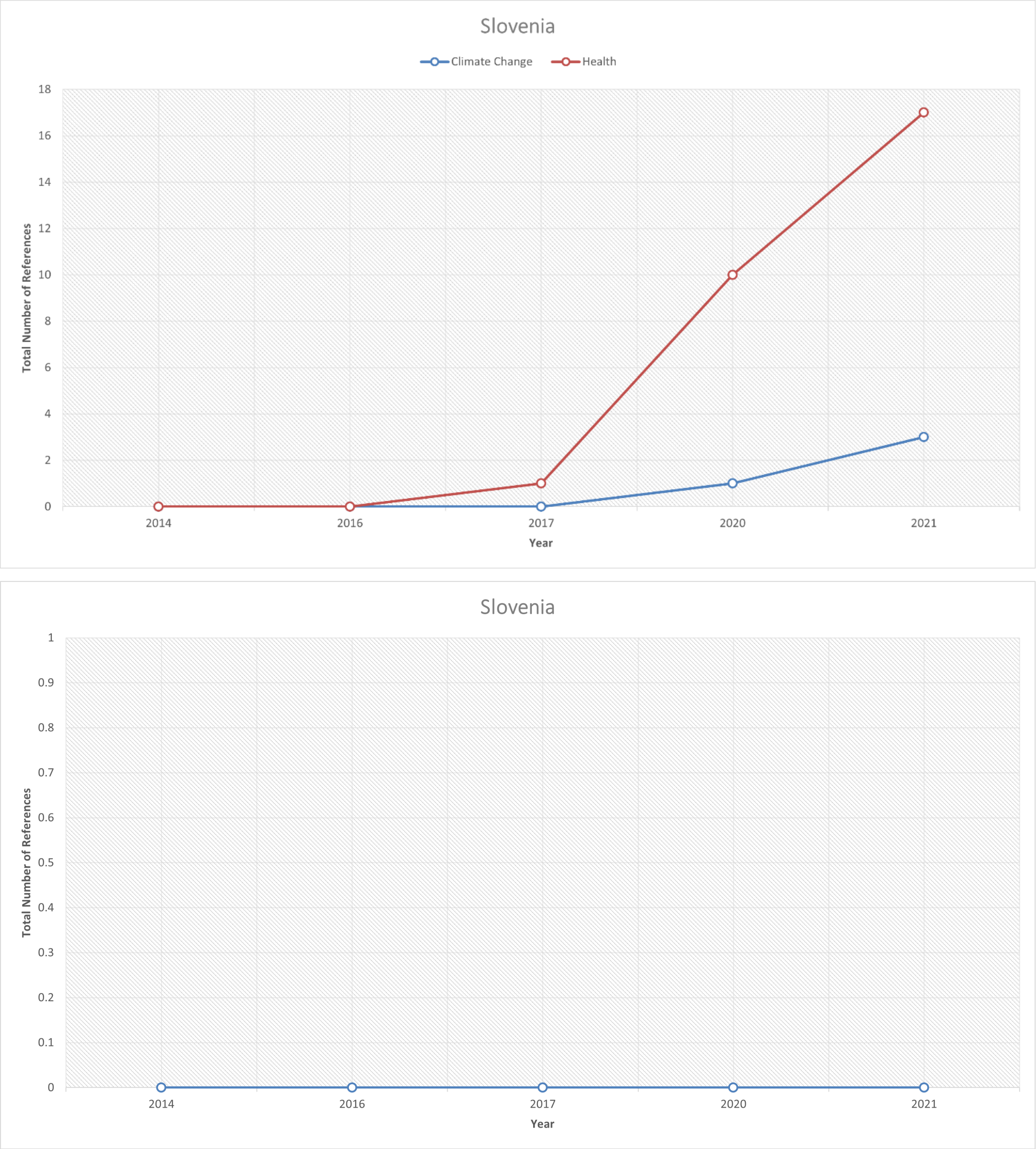

Figure 5.42 Total number of references to the intersection of health and climate change.

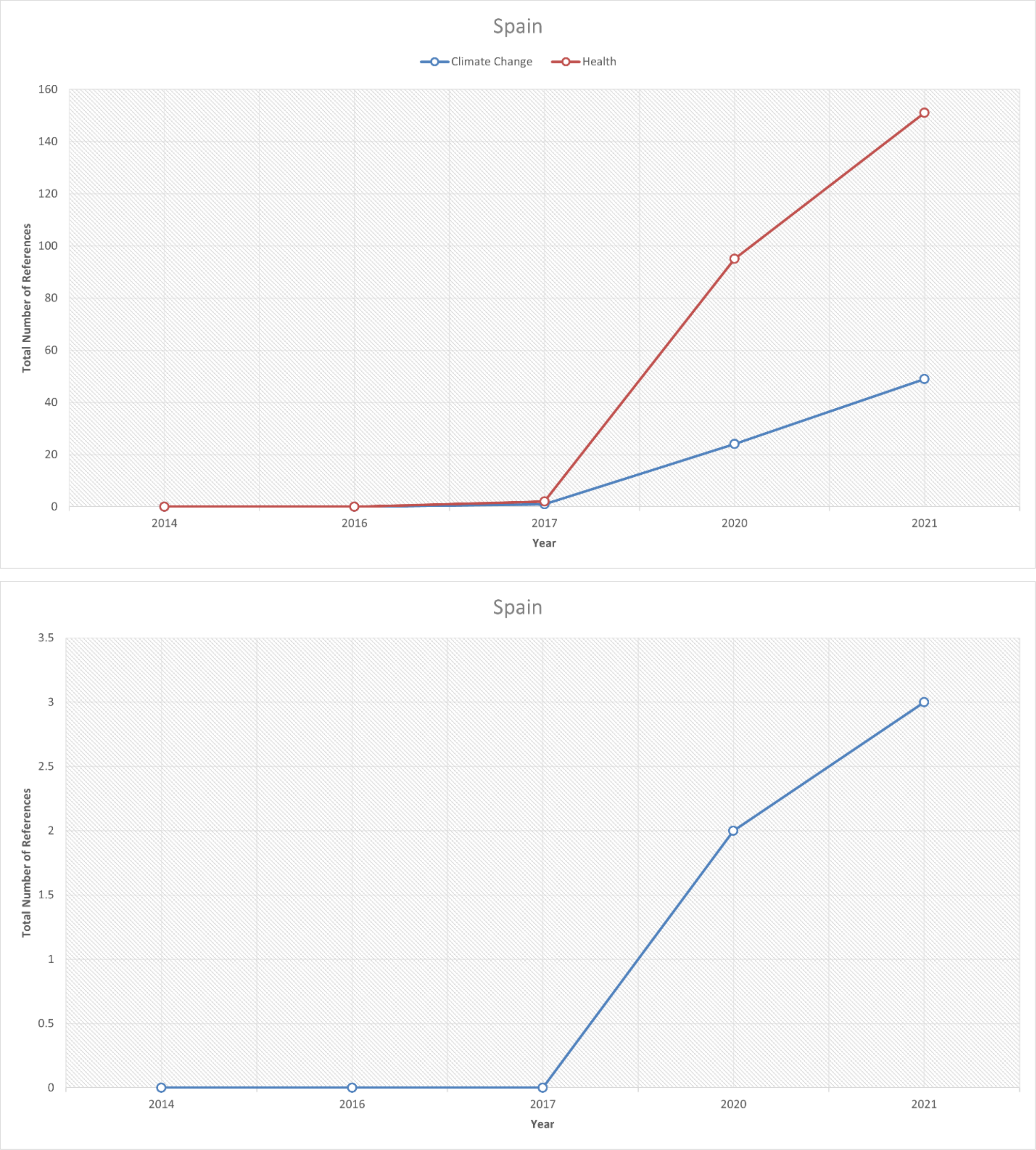

Figure 5.43 Total number of references to the intersection of health and climate change.

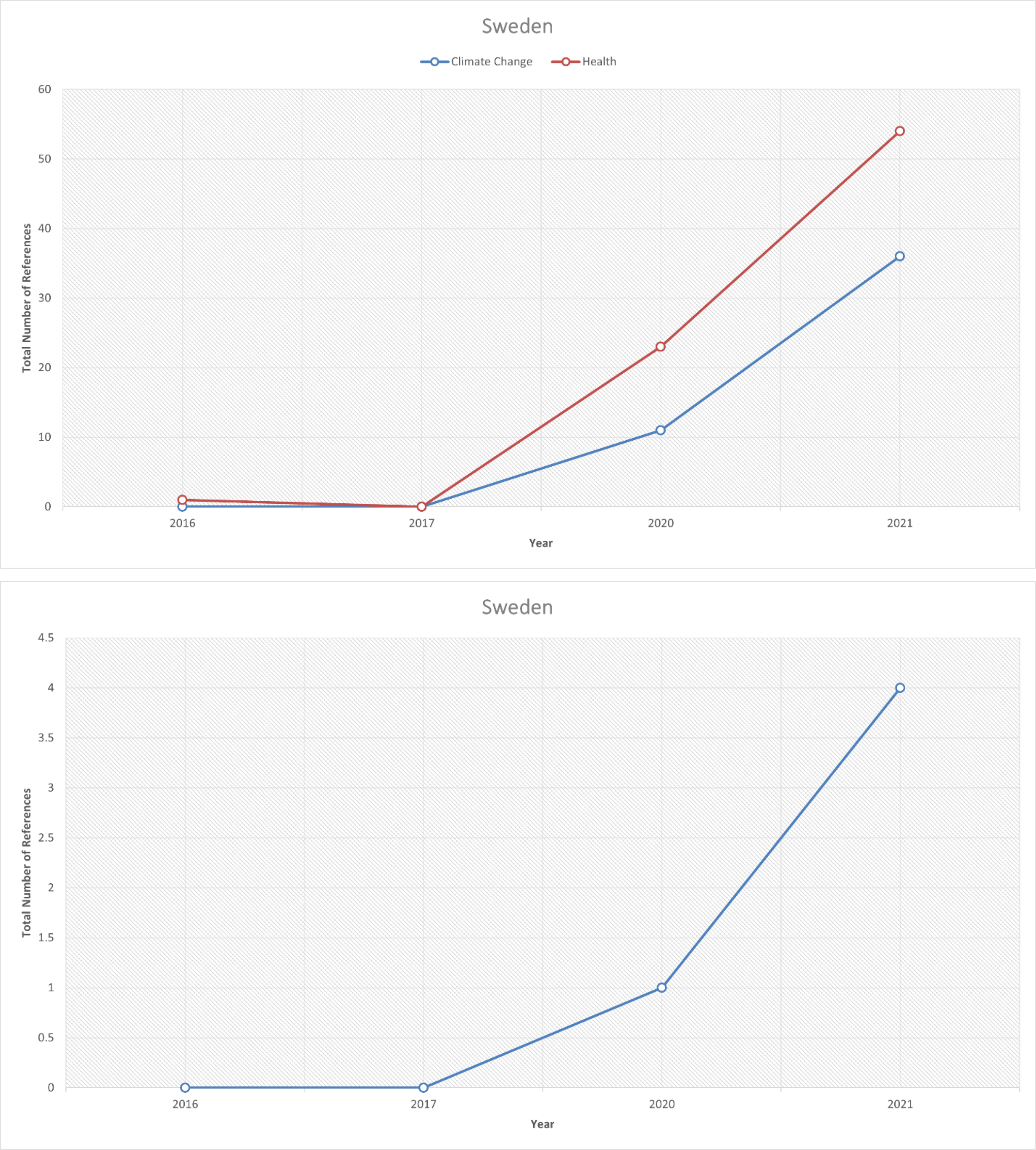

Figure 5.44 Total number of references to the intersection of health and climate change.

Indicator 5.4: Corporate engagement

Method

In this report, we measure the engagement with climate change and health in companies' UN Global Compact Communication on Progress (GCCOP) reports. We use the publicly available COP reports to produce indicators based on identifying references to key search terms on health and climate change. We furthermore examine the awareness of gendered impacts of climate change and health, and that of covid.

In order to produce these indicators, we focus on whether any of the climate change related terms appeared immediately before or after any public health terms in the COP reports. This was based on a search of the 25 words before and after a reference to a public health related term. To further produce indicators involving COVID-19 and gender, we used additional search terms related to gender and COVID-19 to identify which of the intersection references also engaged with these issues.

We provide a full list of terms in table 5.6.

Table 5.6 A comprehensive list of terms.

Health terms	Climate change terms	Covid terms	Gender terms
malaria	climate change	covid	gender
diarrhoea	changing climate	covid-19	male
infection	climate emergency	covid19	female
disease	climate action	covid 19	man
diseases	climate crisis	corona	men
sars	climate decay	coronavirus	woman
measles	global warming	sars-cov-2	women
pneumonia	green house		sex
epidemic	temperature		
epidemics	extreme weather		
pandemic	global environmental		
pandemics	change		
epidemiology	climate variability		
healthcare	greenhouse		
health	greenhouse-gas		
mortality	low carbon		
morbidity	ghge		
nutrition	ghges		
illness	renewable energy		
illnesses	carbon emission		
ncd	carbon emissions		
ncds	carbon dioxide		
air pollution	carbon-dioxide		
nutrition	co2 emission		
malnutrition	co2 emissions		
malnourishment	climate pollutant		

mental disorder	climate pollutants
mental disorders	decarbonization
stunting	decarbonisation
	carbon neutral
	carbon-neutral
	carbon neutrality
	climate neutrality
	net-zero
	net zero

Geographic coverage of Europe

For this indicator we included European Environment Agency (EEA) member and cooperating countries plus the United Kingdom.

Data

To produce this indicator, we draw on the publicly available UN GCCOP reports. A total of 57,915 reports were downloaded from GCCOP. The reports are available for companies based in 129 countries.

GCCOP reports are submitted in different languages. For the development of our indicator, we focused on three different groups of reports: the convertible reports available in English, the available reports from the European Environment Agency (EEA) region, and the available reports from the globe. A number of files were corrupt or could not be converted into plain text format for analysis. The distribution of available English-language reports over time is presented in table 5.7.

Table 5.7 Distribution of Available English-language reports over time.

Year	Number of reports English	Number of reports EEA	Number of reports Global
2011	1052	1059	2036
2012	1422	1658	2991
2013	1597	1688	3207
2014	1721	1738	3162
2015	1812	1921	3454
2016	1974	1926	3554
2017	2006	2003	3711
2018	2035	2075	3741
2019	2240	2298	4041
2020	2062	2062	3542
2021	3104	3206	5720
Total	21025	21634	39159

There are only single GCCOP report submissions before 2011, thus we limit the sample of COP reports to the period 2011-2021. We pre-processed and prepared these documents for the application of natural language processing by converting the reports to plain text format as well as translating non-English documents to English texts. We used the open-source pretrained neural machine translation model Opus-MT[195] under the Huggingface[196] pipeline to implement the translation task. This step is very time-consuming and may need days to complete. We conducted all analysis in English. Further text processing involves removing punctuation and numbers (except 1 and 9 which are included in COVID terms); removing stopwords; regularising (lowercasing). All pre-processing and analysis was carried out in Python using the NLTK package.

Additional Analysis on Health and Climate Change

We present some additional findings and figures in this section.

Figure 5.45 represents the proportion of companies that reference climate change, health and the intersection of climate change and health in the GCCOP reports. It shows the proportion of companies that mention terms associated with health and climate change, regardless of how many times they are mentioned. Since 2019, we observe an overall increase in the mention of health and climate change, though the intersection of health and climate change remains low relative to both.

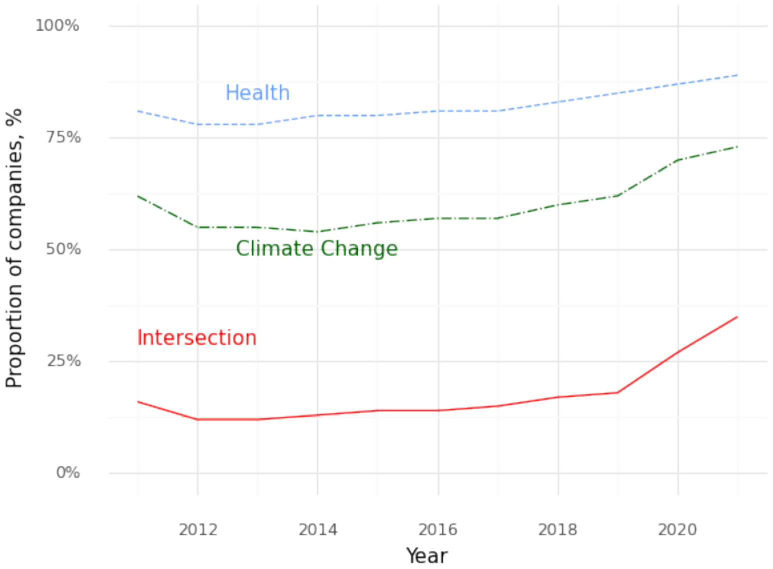

Figure 5.45 Proportion of companies that reference climate change, health and the intersection of climate change and health, 2011-2021

Figure 5.46 presents the total number of references to climate change, health, and the intersection of climate change and health across for the GCCOP reports. Despite the increase in the proportion of companies engaging with the climate change-health linkages, the overall number of references remains fairly low and consistent, relative to the individual references to health and climate change. The results show a sharp increase starting in 2020, especially in health, though the intersection remains relatively low.

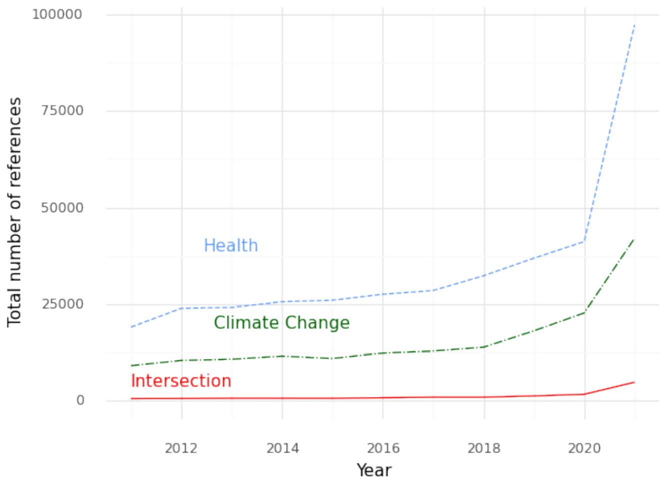

Figure 5.46 Total references to climate change, health and the intersection of climate change and health, 2011-2021

Figure 5.47 shows the total references with the intersection of climate change and health to better show any trends occurring in engagement. The figure shows that since 2018, and continuing into 2021, there has been a sharp rise in the number of references.

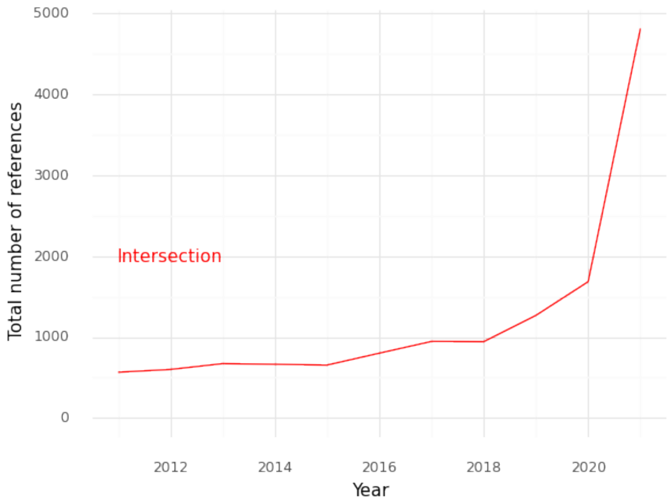

Figure 5.47 Total references to the intersection of climate change and health, 2011-2021.

Figure 5.48 shows the average number of references to climate change, health, and the intersection in GCCOP reports. This represents the total number of references normalised by the number of companies. The figure again demonstrates the relatively low level of engagement with the health impacts of climate change in GCCOP reports, compared to the separate references to health and climate change.

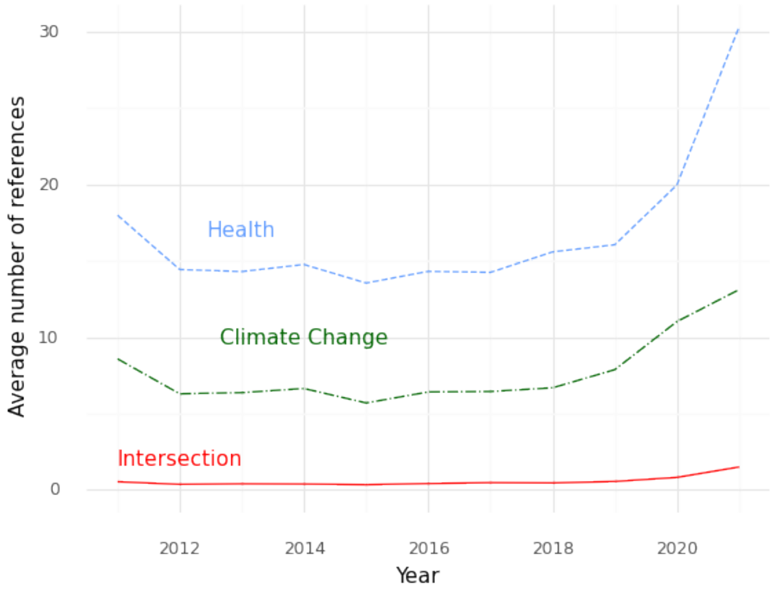

Figure 5.48 Average references to climate change, health and the intersection of climate change and health in GCCOP reports, 2011-2021.

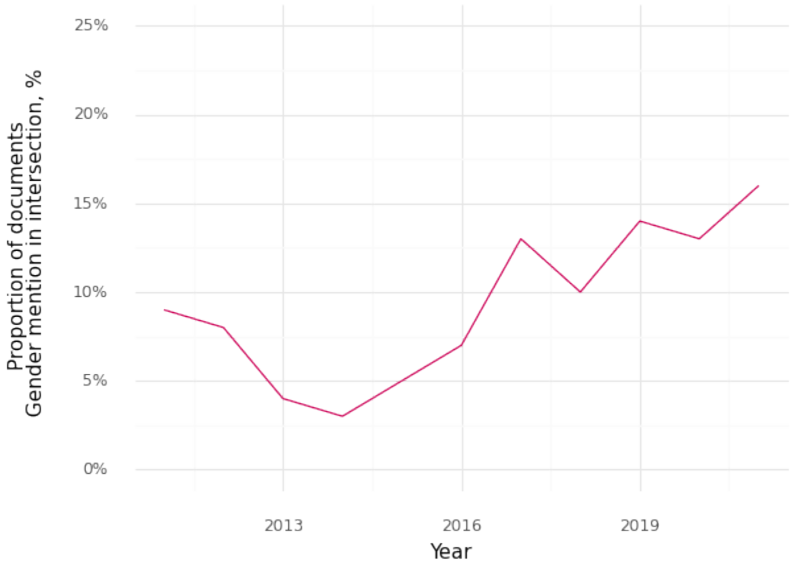

Figure 5.49 Proportion of references to the intersection of health and gender that include a reference to gender, 2011-2021

Additional Analysis on the Gendered Impacts of Health and Climate Change

Figure 5.49 presents annual references to the gender dimensions of climate change and health in UN Global Compact COP reports between 2011 and 2020. The figure shows a steady increase in engagement between 2014 and 2018, with another sharp rise in 2018, continuing in 2021.

Health and Climate Change by Sector

Figure 5.50 breaks down engagement by sector, showing references to health, climate change, and the intersection of health and climate change for companies grouped into sectors. This figure shows the proportion of companies for each sector that reference these terms, regardless of the number of times they are referenced. The highest level

of engagement with the impact of climate change on health comes from the Pharmaceuticals and Biotechnology sector. Despite a high engagement with both health and climate change from the Food & Beverage sector, there seems to be little focus on the intersection thereof.

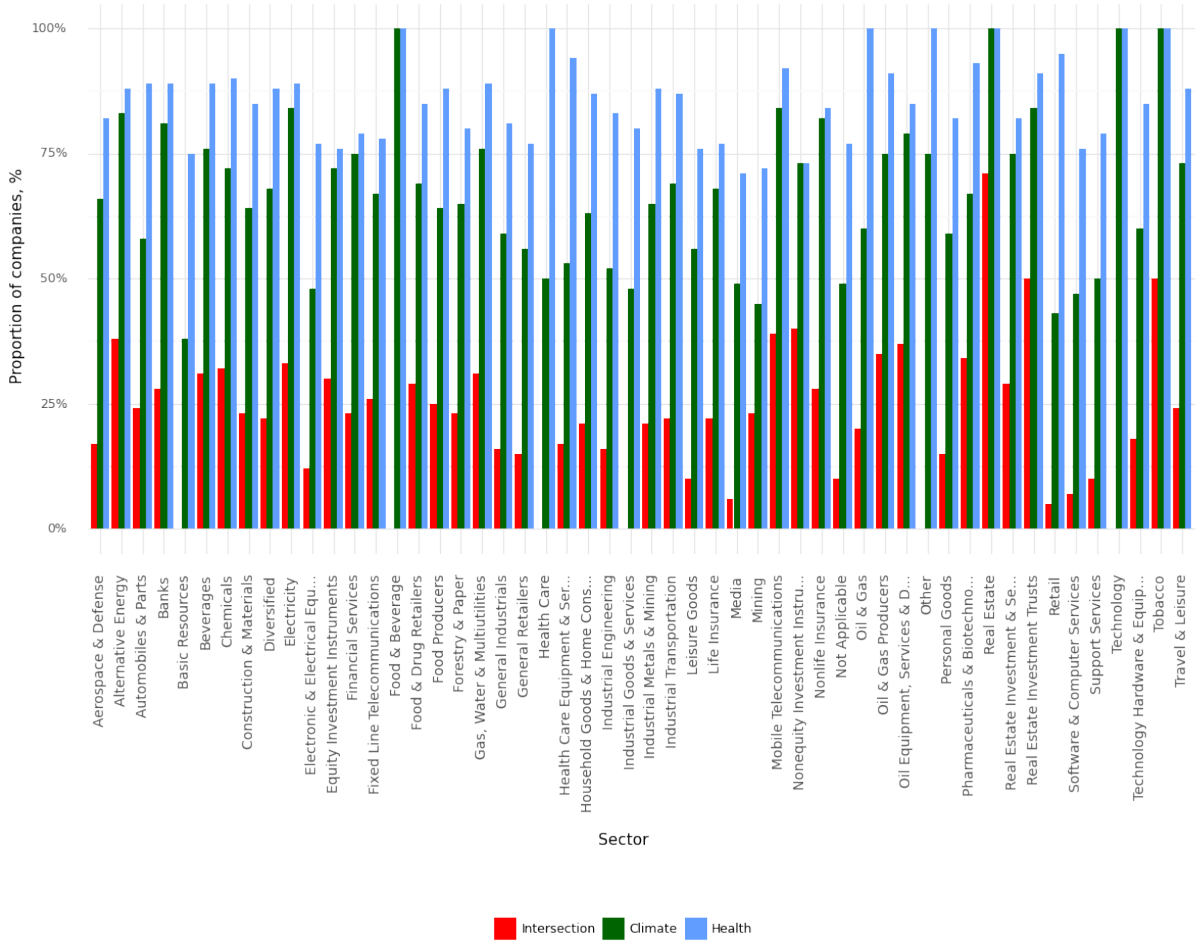

Figure 5.50 Proportion of companies by sector that mention health, climate change, and the intersection of health and climate change since 2011.

In order to provide a stronger focus on companies directly related to the health sector, we filter the reports by “Health Care Equipment and Services”. Figure 5.51 takes a look at companies that belong to this sector only, and shows the proportion of companies in that sector that mention health, climate change, and the intersection of health and climate change.

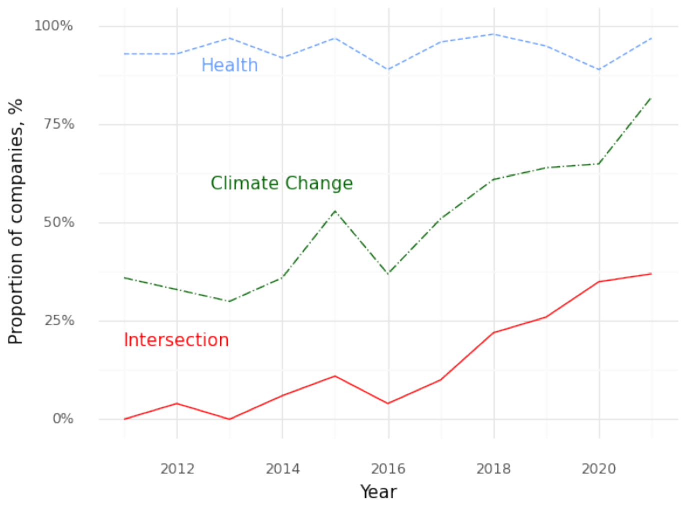

Figure 5.51 Proportion of companies within the health sector that mention health, climate change, and the intersection of health and climate change, 2011-2021

Figure 5.52 shows the total references to health, climate change, and the intersection of health and climate change for the health sector. The overall number of references remains fairly low, and the intersection negligible, although there is a small but consistent rise in the last year.

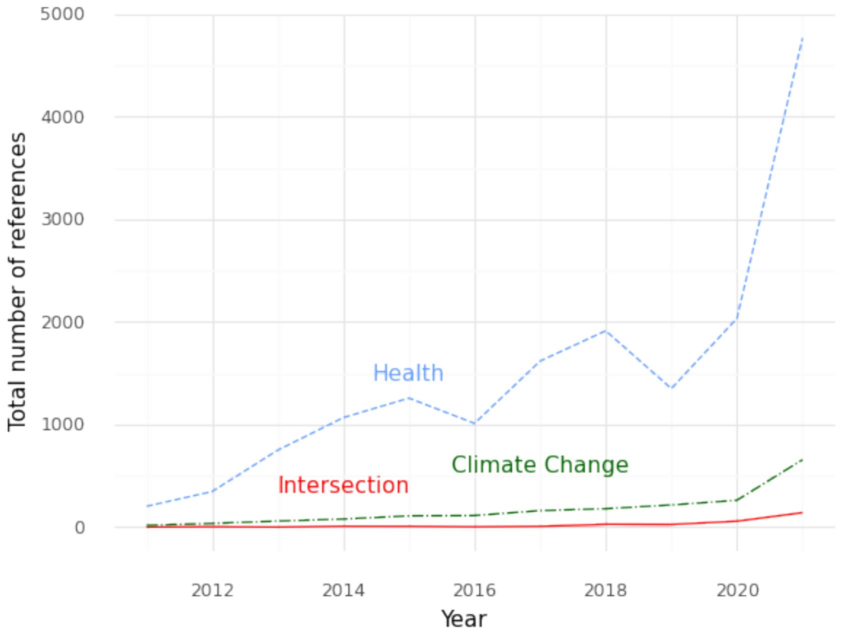

Figure 5.52 Total references to climate change, health and the intersection of climate change and health, for the health sector 2011-2021

Figure 5.53 shows the average number of references to climate change, health, and the intersection in GCCOP. reports, again filtered for the health sector. The figure again demonstrates the relatively low level of engagement with the health impacts of climate change in GCCOP reports, with only a slight increase in 2020.

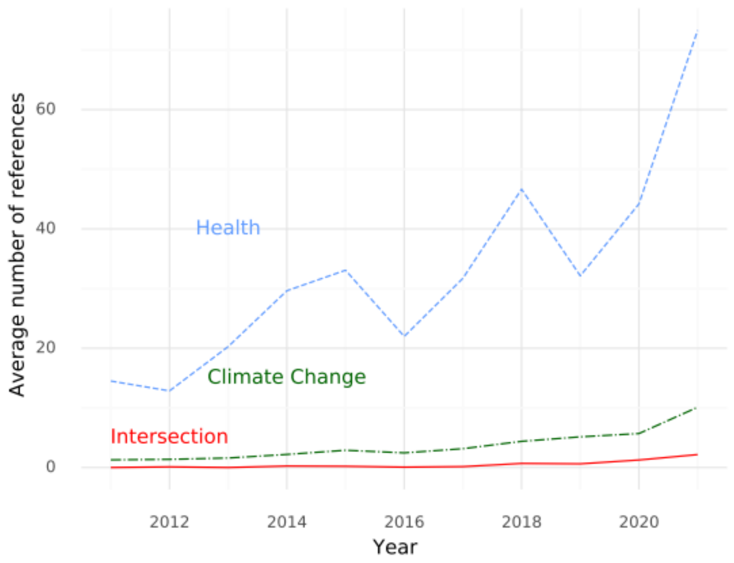

Figure 5.53 Average references to climate change, health and the intersection of climate change and health in GCCOP reports for the health sector, 2011-2021.

Additional information to Figure 10 in the main text

In Figure 10 of the main manuscript, an overview figure of a selected set of standardised climate-related health impact indicators and climate change response indicators of the 2022 European *Lancet* Countdown report is provided. Table 6 and Table 7 provide details on the standardisation process for the generation of a heatmap. In table 6, **unit** refers to the unit utilised in the original indicator (as reported within this report), and **baseline value** refers to the mean value of the original indicator over the **baseline period**. In table 7, **worst case value** refers to the worst value reported over the time-period measured of the original indicator, and **target value** refers to the desired value for each indicator. In both tables, **yearly score** refers to the annual original value (in the original unit) reported for each indicator. These methods are based on the methods of the China *Lancet* Countdown (Cai *et al.* 2021).[197]

Table 6 Description of the standardisation of impact indicators used in figure 10

Indicator	Unit	Baseline period	Baseline value	Time coverage	Standardization
Vulnerability to heat exposure	Annual heat vulnerability index (1-100)	1990-1994	38.29	1990-2019	$yearly\ score = \frac{year\ value}{baseline\ value}$
Exposure of vulnerable populations to change in heatwaves	Annual person-days of heatwave exposure of people >65 years	2000-2004	653393801	2000-2020	
Physical activity and heat stress	Annual number of hours in which heat exposure poses a risk of heat stress during physical exercise (high intensity)	1990-1994	163.56	1990-2020	
Heat-attributable mortality	Annual death per million per decade	2000-2004	44.86	2000-2020	
Wildfire smoke	Annual average population-weighted wildfire-$PM_{2.5}$ exposure	2003-2007	409.27	2003-2020	
Drought	Annual % land area under extreme drought	1951-1960	9.53	1951-2020	
Climate suitability for West Nile virus	Annual WNV outbreak risk probability	1951-1960	0.0116	1951-2020	
Climate suitability for dengue	Annual R0	1951-1960	0.0931	1951-2020	
Climate suitability for chikungunya	Annual R0	1951-1960	0.0838	1951-2020	
Climate suitability for Zika	Annual R0	1951-1960	0.1460	1951-2020	
Climate suitability for malaria	Annual mean number of months suitable for malaria transmission	1951-1960	0.39	1951-2020	
Economic losses due to climate-related extreme events	Annual economic losses due to climate-related extreme event in euros (billions)	2010	35.6	2010-2021	

Table 7 Description of the standardisation of impact indicators used in figure 10

Indicator	Original data	Time coverage	Worst case year	Worst case value	Target value	Target meaning	Standardisation
Reduction in premature mortality attributable to ambient fine particles	Annual total deaths attributable to ambient fine particles ($PM_{2.5}$)	2005-2020	2005	293452	0	No deaths from premature mortality attributable to air pollution	yearly score $= \frac{yearly\ value - worst\ case\ value}{target\ value - worst\ case\ value}$
Reduction of carbon intensity	Annual CO_2 / total energy supply (tCO_2 per TJ)	1990-2020	1990	58.14	0	Carbon neutrality	
Coal phase-out	Annual share (%) of coal in total energy supply	1991-2020	1991	25.68%	0	Total coal phase-out	
Zero-carbon emission energy	Annual share (%) of zero-carbon electricity in total electricity generation	1991-2020	2007	42.68%	100	100% zero-carbon electricity	
Reduction in carbon emission food consumption	Annual $GtCO_2$-equivalent of life-cycle emissions food consumption	2010-2019	2010	1.87	0	100% zero-carbon food consumption	
Net carbon price	Annual average carbon price (euros / tonne)	2010-2019	2014	-17.76	0	Zero fossil fuel subsidies	

References

1. Countries — European Environment Agency. Accessed March 19, 2022. https://www.eea.europa.eu/countries-and-regions
2. WHO Europe | Countries. Accessed March 19, 2022. https://www.euro.who.int/en/countries
3. Country profiles European Union . Accessed March 19, 2022. https://european-union.europa.eu/principles-countries-history/country-profiles_en
4. Negotiations status. Accessed March 19, 2022. https://ec.europa.eu/neighbourhood-enlargement/enlargement-policy/negotiations-status_en
5. The EFTA States | European Free Trade Association. Accessed March 19, 2022. https://www.efta.int/about-efta/the-efta-states
6. Global Burden of Disease Study 2019 (GBD 2019) Population Estimates 1950-2019 | GHDx. Accessed March 18, 2022. https://ghdx.healthdata.org/record/ihme-data/gbd-2019-population-estimates-1950-2019
7. Global Burden of Disease Collaborative Network. Global Burden of Disease Study 2019 (GBD 2019) Results. Seattle, United States: Institute for Health Metrics and Evaluation (IHME). Published 2020. Accessed March 18, 2022. https://ghdx.healthdata.org/gbd-results-tool.
8. World Bank. United Nations Population Division's World Urbanization Prospects: 2018 Revision. Accessed March 18, 2022. https://data.worldbank.org/indicator/SP.URB.TOTL
9. Chambers J. Global and cross-country analysis of exposure of vulnerable populations to heatwaves from 1980 to 2018. *Clim Change*. 2020;163(1):539-558. doi:10.1007/S10584-020-02884-2/FIGURES/8
10. Jay O. *Extreme Heat Policy*.; 2021.
11. Beggs PJ, Zhang Y, McGushin A, et al. The 2021 report of the MJA–Lancet Countdown on health and climate change: Australia increasingly out on a limb. *Med J Aust*. 2021;215(9):390-392.e22. doi:10.5694/MJA2.51302
12. Barthelme S. CRAN - Package imager. Accessed March 18, 2022. https://cran.r-project.org/web/packages/imager/index.html
13. ERA5-Land hourly data from 1950 to present. Accessed March 8, 2022. https://cds.climate.copernicus.eu/cdsapp#!/dataset/reanalysis-era5-land?tab=overview
14. May RM, Arms SC, Marsh P. MetPy: A Python Package for Meteorological Data. Published 2022. Accessed March 18, 2022. doi:doi:10.5065/D6WW7G29
15. GEOSTAT - GISCO - Eurostat. Accessed December 3, 2021. https://ec.europa.eu/eurostat/web/gisco/geodata/reference-data/population-distribution-demography/geostat
16. Hernangómez D. giscoR: Download Map Data from GISCO API - Eurostat. doi:10.5281/ZENODO.4317947
17. GISCO - Eurostat. Published 2022. Accessed March 18, 2022. https://ec.europa.eu/eurostat/web/gisco
18. Research informs Sports Medicine Australia's new heat policy | Mirage News. Accessed March 18, 2022. https://www.miragenews.com/research-informs-sports-medicine-australias-new-518619/
19. Library WO, Gasparrini A. Modeling exposure–lag–response associations with distributed lag non-linear models. *Stat Med*. 2014;33(5):881-899. doi:10.1002/SIM.5963

20. Ballester J, Lloyd S, Quijal-Zamorano M. Effect of temporal data resolution in time-series epidemiological studies of temperature attributable mortality. Published online 2022.
21. Sera F, Armstrong B, Blangiardo M, Gasparrini A. An extended mixed-effects framework for meta-analysis. *Stat Med*. 2019;38(29):5429-5444. doi:10.1002/SIM.8362
22. Gasparrini A, Leone M. Attributable risk from distributed lag models. *BMC Med Res Methodol*. 2014;14(1):1-8. doi:10.1186/1471-2288-14-55/FIGURES/3
23. Vicedo-Cabrera AM, Sera F, Gasparrini A. Hands-on Tutorial on a Modeling Framework for Projections of Climate Change Impacts on Health. *Epidemiology*. 2019;30(3):321-329. doi:10.1097/EDE.0000000000000982
24. Weekly death statistics - Eurostat. Accessed March 14, 2022. https://ec.europa.eu/eurostat/statistics-explained/index.php?title=Weekly_death_statistics&stable
25. Eurostat. Regional demographic statistics. Accessed March 18, 2022. https://ec.europa.eu/eurostat/data/database?node_code=demo_r_pjangrp3
26. Martínez-Solanas È, Quijal-Zamorano M, Achebak H, et al. Projections of temperature-attributable mortality in Europe: a time series analysis of 147 contiguous regions in 16 countries. *Lancet Planet Heal*. 2021;5(7):e446-e454. doi:10.1016/S2542-5196(21)00150-9/ATTACHMENT/CF8FBC2A-0B57-4F32-99B4-3395CAE5A955/MMC1.PDF
27. ISGlobal. EARLY-ADAPT. Accessed March 18, 2022. https://early-adapt.eu/
28. Robine JM, Cheung SLK, Le Roy S, et al. Death toll exceeded 70,000 in Europe during the summer of 2003. *Comptes Rendus - Biol*. 2008;331(2):171-178. doi:10.1016/j.crvi.2007.12.001
29. Achebak H, Devolder D, Ballester J. Trends in temperature-related age-specific and sex-specific mortality from cardiovascular diseases in Spain: a national time-series analysis. *Lancet Planet Heal*. 2019;3(7):e297-e306. doi:10.1016/S2542-5196(19)30090-7/ATTACHMENT/9FF2613A-A391-4E95-B6C6-0B1E83DC2A39/MMC1.PDF
30. Achebak H, Devolder D, Ingole V, Ballester J. Reversal of the seasonality of temperature-attributable mortality from respiratory diseases in Spain. *Nat Commun 2020 111*. 2020;11(1):1-9. doi:10.1038/s41467-020-16273-x
31. Orellano P, Quaranta N, Reynoso J, Balbi B, Vasquez J. Effect of outdoor air pollution on asthma exacerbations in children and adults: Systematic review and multilevel meta-analysis. *PLoS One*. 2017;12(3). doi:10.1371/JOURNAL.PONE.0174050
32. Hänninen R, Sofiev M, Uppstu A, Kouznetsov R. Daily surface concentration of fire related PM2.5 for 2003-2021, modelled by SILAM CTM when using the MODIS satellite data for the fire radiative power [Dataset]. Finnish Meteorological Institute. Published 2022. https://doi.org/10.23728/FMI-B2SHARE.A006840CCE9340E8BF11E562BB8D396E
33. Sofiev M, Vankevich R, Lotjonen M, et al. An operational system for the assimilation of the satellite information on wild-land fires for the needs of air quality modelling and forecasting. *Atmos Chem Phys*. 2009;9(18):6833-6847. doi:10.5194/ACP-9-6833-2009
34. Soares J, Sofiev M, Hakkarainen J. Uncertainties of wild-land fires emission in AQMEII phase 2 case study. *Atmos Environ*. 2015;115:361-370. doi:10.1016/J.ATMOSENV.2015.01.068
35. Kollanus V, Prank M, Gens A, et al. Mortality due to Vegetation Fire–Originated PM2.5 Exposure in

Europe—Assessment for the Years 2005 and 2008. *Environ Health Perspect*. 2017;125(1):30. doi:10.1289/EHP194

36. Zapata-Diomedi B, Barendregt JJ, Veerman JL. Population attributable fraction: names, types and issues with incorrect interpretation of relative risks. *Br J Sports Med*. 2018;52(4):212-213. doi:10.1136/BJSPORTS-2015-095531

37. Khomenko S, Cirach M, Pereira-Barboza E, et al. Premature mortality due to air pollution in European cities: a health impact assessment. *Lancet Planet Heal*. 2021;5(3):e121-e134. doi:10.1016/S2542-5196(20)30272-2/ATTACHMENT/EA6D2A19-0992-4ED1-AC46-9FF42EA6DB7B/MMC1.PDF

38. Aguilera R, Corringham T, Gershunov A, Benmarhnia T. Wildfire smoke impacts respiratory health more than fine particles from other sources: observational evidence from Southern California. *Nat Commun 2021 121*. 2021;12(1):1-8. doi:10.1038/s41467-021-21708-0

39. Chen G, Guo Y, Yue X, et al. Mortality risk attributable to wildfire-related PM2·5 pollution: a global time series study in 749 locations. *Lancet Planet Heal*. 2021;5(9):e579-e587. doi:10.1016/S2542-5196(21)00200-X/ATTACHMENT/E841C89B-3E49-4AB5-AFB7-E93EA966CFB8/MMC1.PDF

40. Maier SW, Russell-Smith J, Edwards AC, Yates C. Sensitivity of the MODIS fire detection algorithm (MOD14) in the savanna region of the Northern Territory, Australia. *ISPRS J Photogramm Remote Sens*. 2013;76:11-16. doi:10.1016/J.ISPRSJPRS.2012.11.005

41. Orellano P, Reynoso J, Quaranta N, Bardach A, Ciapponi A. Short-term exposure to particulate matter (PM10 and PM2.5), nitrogen dioxide (NO2), and ozone (O3) and all-cause and cause-specific mortality: Systematic review and meta-analysis. *Environ Int*. 2020;142:105876. doi:10.1016/J.ENVINT.2020.105876

42. Vicente-Serrano SM, Beguería S, López-Moreno JI. A Multiscalar Drought Index Sensitive to Global Warming: The Standardized Precipitation Evapotranspiration Index. *J Clim*. 2010;23(7):1696-1718. doi:10.1175/2009JCLI2909.1

43. Solaraju-Murali B, Gonzalez-Reviriego N, Caron LP, et al. Multi-annual prediction of drought and heat stress to support decision making in the wheat sector. *npj Clim Atmos Sci 2021 41*. 2021;4(1):1-9. doi:10.1038/s41612-021-00189-4

44. Hargreaves GH. Defining and Using Reference Evapotranspiration. *J Irrig Drain Eng*. 1994;120(6):1132-1139. doi:10.1061/(ASCE)0733-9437(1994)120:6(1132)

45. Vicente-Serrano SM, Beguería S. Comment on 'Candidate distributions for climatological drought indices (SPI and SPEI)' by James H. Stagge et al. *Int J Climatol*. 2016;36(4):2120-2131. doi:10.1002/JOC.4474

46. Stagge JH, Tallaksen LM, Gudmundsson L, Van Loon AF, Stahl K. Candidate Distributions for Climatological Drought Indices (SPI and SPEI). *Int J Climatol*. 2015;35(13):4027-4040. doi:10.1002/JOC.4267

47. Beguería S, Vicente-Serrano SM, Reig F, Latorre B. Standardized precipitation evapotranspiration index (SPEI) revisited: parameter fitting, evapotranspiration models, tools, datasets and drought monitoring. *Int J Climatol*. 2014;34(10):3001-3023. doi:10.1002/JOC.3887

48. Baker-Austin C, Trinanes JA, Taylor NGH, Hartnell R, Siitonen A, Martinez-Urtaza J. Emerging Vibrio risk at high latitudes in response to ocean warming. *Nat Clim Chang*. 2013;3(1):73-77.

doi:10.1038/nclimate1628
49. Trinanes J, Atlantic) ;, Martinez-Urtaza J, Trinanes J. Future scenarios of risk of Vibrio infections in a warming planet: a global mapping study. *Lancet Planet Heal*. 2021;5(7):e426-e435. doi:10.1016/S2542-5196(21)00169-8
50. Newton A, Kendall M, Vugia DJ, Henao OL, Mahon BE. Increasing Rates of Vibriosis in the United States, 1996–2010: Review of Surveillance Data From 2 Systems. *Clin Infect Dis*. 2012;54(0 5):S391. doi:10.1093/CID/CIS243
51. Ralston EP, Kite-Powell H, Beet A. An estimate of the cost of acute food and water borne health effects from marine pathogens and toxins in the United States. *J Water Health*. 2011;9(4):680. doi:10.2166/WH.2011.157
52. Kriticos DJ, Jarošik V, Ota N. Extending the suite of bioclim variables: a proposed registry system and case study using principal components analysis. *Methods Ecol Evol*. 2014;5(9):956-960. doi:10.1111/2041-210X.12244
53. European Centre for Disease Prevention and Control. Accessed March 18, 2022. https://www.ecdc.europa.eu/en
54. Introduction to Boosted Trees — xgboost 1.6.0-dev documentation. Accessed March 18, 2022. https://xgboost.readthedocs.io/en/latest/tutorials/model.html
55. Chen T, Guestrin C. XGBoost: A Scalable Tree Boosting System. *Proc 22nd ACM SIGKDD Int Conf Knowl Discov Data Min*. doi:10.1145/2939672
56. Notes on the N-Person Game — II: The Value of an N-Person Game. *Notes N-Person Game — II Value an N-Person Game*. Published online March 29, 2021. doi:10.7249/RM0670
57. Lundberg SM, Allen PG, Lee S-I. A Unified Approach to Interpreting Model Predictions. doi:10.5555/3295222
58. Lundberg SM, Erion G, Chen H, et al. From local explanations to global understanding with explainable AI for trees. *Nat Mach Intell 2020 21*. 2020;2(1):56-67. doi:10.1038/s42256-019-0138-9
59. Molnar C. Interpretable Machine Learning. Accessed March 18, 2022. https://christophm.github.io/interpretable-ml-book/
60. Hersbach H, Bell B, Berrisford P, et al. The ERA5 global reanalysis. *Q J R Meteorol Soc*. 2020;146(730):1999-2049. doi:10.1002/QJ.3803
61. Stanaway JD, Shepard DS, Undurraga EA, et al. The global burden of dengue: an analysis from the Global Burden of Disease Study 2013. *Lancet Infect Dis*. 2016;16(6):712-723. doi:10.1016/S1473-3099(16)00026-8
62. Hales S, De Wet N, Maindonald J, Woodward A. Potential effect of population and climate changes on global distribution of dengue fever: an empirical model. *Lancet*. 2002;360(9336):830-834. doi:10.1016/S0140-6736(02)09964-6
63. Rocklöv J, Tozan Y. Climate change and the rising infectiousness of dengue. *Emerg Top Life Sci*. 2019;3(2):133. doi:10.1042/ETLS20180123
64. Rocklöv J, Quam MB, Sudre B, et al. Assessing Seasonal Risks for the Introduction and Mosquito-borne Spread of Zika Virus in Europe. *eBioMedicine*. 2016;9:250-256. doi:10.1016/J.EBIOM.2016.06.009

65. Rocklöv J, Tozan Y, Ramadona A, et al. Using Big Data to Monitor the Introduction and Spread of Chikungunya, Europe. *Emerg Infect Dis*. 2019;25(6):1041-1049. doi:10.3201/EID2506.180138
66. Liu-Helmersson J, Brännström Å, Sewe MO, Semenza JC, Rocklöv J. Estimating past, present, and future trends in the global distribution and abundance of the arbovirus vector Aedes aegypti under climate change scenarios. *Front Public Heal*. 2019;7(JUN):148. doi:10.3389/FPUBH.2019.00148/BIBTEX
67. DiSera L, Sjödin H, Rocklöv J, et al. The Mosquito, the Virus, the Climate: An Unforeseen Réunion in 2018. *GeoHealth*. 2020;4(8). doi:10.1029/2020GH000253
68. Colón-González FJ, Sewe MO, Tompkins AM, et al. Projecting the risk of mosquito-borne diseases in a warmer and more populated world: a multi-model, multi-scenario intercomparison modelling study. *Lancet Planet Heal*. 2021;5(7):e404-e414. doi:10.1016/S2542-5196(21)00132-7/ATTACHMENT/F6794FC7-A9E6-410F-B0FB-86D5C90BA907/MMC1.PDF
69. Chambers J. Hybrid gridded demographic data for the world, 1950-2020. Published online April 27, 2020. doi:10.5281/ZENODO.3768003
70. Liu-Helmersson J, Stenlund H, Wilder-Smith A, Rocklöv J. Vectorial Capacity of Aedes aegypti: Effects of Temperature and Implications for Global Dengue Epidemic Potential. *PLoS One*. 2014;9(3). doi:10.1371/JOURNAL.PONE.0089783
71. Liu-Helmersson J, Quam M, Wilder-Smith A, et al. Climate Change and Aedes Vectors: 21st Century Projections for Dengue Transmission in Europe. *EBioMedicine*. 2016;7:267-277. doi:10.1016/J.EBIOM.2016.03.046
72. Simini F, González MC, Maritan A, Barabási AL. A universal model for mobility and migration patterns. *Nat 2012 4847392*. 2012;484(7392):96-100. doi:10.1038/nature10856
73. The World Tourism Organization (UNWTO). Accessed March 18, 2022. https://www.unwto.org/
74. Rocklöv J, Lohr W, Hjertqvist M, Wilder-Smith A. Attack rates of dengue fever in Swedish travellers. *http://dx.doi.org/103109/003655482014887222*. 2014;46(6):412-417. doi:10.3109/00365548.2014.887222
75. Stefanouli M, Polyzos S. Gravity vs radiation model: two approaches on commuting in Greece. *Transp Res Procedia*. 2017;24:65-72. doi:10.1016/J.TRPRO.2017.05.069
76. Patz JA, Graczyk TK, Geller N, Vittor AY. Effects of environmental change on emerging parasitic diseases. *Int J Parasitol*. 2000;30(12-13):1395-1405. doi:10.1016/S0020-7519(00)00141-7
77. Boualam MA, Pradines B, Drancourt M, Barbieri R. Malaria in Europe: A Historical Perspective. *Front Med*. 2021;8:876. doi:10.3389/FMED.2021.691095/BIBTEX
78. Fischer L, Gültekin N, Kaelin MB, Fehr J, Schlagenhauf P. Rising temperature and its impact on receptivity to malaria transmission in Europe: A systematic review. *Travel Med Infect Dis*. 2020;36:101815. doi:10.1016/J.TMAID.2020.101815
79. Zhao X, Smith DL, Tatem AJ. Exploring the spatiotemporal drivers of malaria elimination in Europe. *Malar J*. 2016;15(1):1-13. doi:10.1186/S12936-016-1175-Z/TABLES/5
80. Grover-Kopec EK, Blumenthal MB, Ceccato P, Dinku T, Omumbo JA, Connor SJ. Web-based climate information resources for malaria control in Africa. *Malar J*. 2006;5(1):1-9. doi:10.1186/1475-2875-5-38/FIGURES/5

81. Lyon B, Dinku T, Raman A, Thomson MC. Temperature suitability for malaria climbing the Ethiopian Highlands. *Environ Res Lett*. 2017;12(6):064015. doi:10.1088/1748-9326/AA64E6
82. Benali A, Nunes JP, Freitas FB, et al. Satellite-derived estimation of environmental suitability for malaria vector development in Portugal. *Remote Sens Environ*. 2014;145:116-130. doi:10.1016/J.RSE.2014.01.014
83. Romanello M, McGushin A, Di Napoli C, et al. The 2021 report of the Lancet Countdown on health and climate change: code red for a healthy future. *Lancet*. 2021;398(10311):1619-1662. doi:10.1016/S0140-6736(21)01787-6/ATTACHMENT/737E7C31-E370-4004-818C-8ED9EE8AF7B8/MMC5.PDF
84. Watts N, Amann M, Arnell N, et al. The 2020 report of The Lancet Countdown on health and climate change: responding to converging crises. *Lancet*. 2021;397(10269):129-170. doi:10.1016/S0140-6736(20)32290-X
85. Watts N, Amann M, Arnell N, et al. The 2019 report of The Lancet Countdown on health and climate change: ensuring that the health of a child born today is not defined by a changing climate. *Lancet*. 2019;394(10211):1836-1878. doi:10.1016/S0140-6736(19)32596-6
86. Watts N, Amann M, Arnell N, et al. The 2018 report of the Lancet Countdown on health and climate change: shaping the health of nations for centuries to come. *Lancet (London, England)*. 2018;392(10163):2479-2514. doi:10.1016/S0140-6736(18)32594-7
87. Arends JE, Oosterheert JJ, Kraaij-Dirkzwager MM, et al. Two Cases of Plasmodium falciparum Malaria in the Netherlands without Recent Travel to a Malaria-Endemic Country. *Am J Trop Med Hyg*. 2013;89(3):527-530. doi:10.4269/AJTMH.13-0213
88. Krüger A, Rech A, Su XZ, Tannich E. Two cases of autochthonous Plasmodium falciparum malaria in Germany with evidence for local transmission by indigenous Anopheles plumbeus. *Trop Med Int Heal*. 2001;6(12):983-985. doi:10.1046/J.1365-3156.2001.00816.X
89. Muñoz-Sabater J, Dutra E, Agustí-Panareda A, et al. ERA5-Land: A state-of-the-art global reanalysis dataset for land applications. *Earth Syst Sci Data*. 2021;13(9):4349-4383. doi:10.5194/ESSD-13-4349-2021
90. Alduchov O. Improved Magnus Form Approximation of Saturation Vapor Pressure. *J Appl Meteorol Climatol*. 1996;33(4). Accessed March 18, 2022. https://journals.ametsoc.org/view/journals/apme/35/4/1520-0450_1996_035_0601_imfaos_2_0_co_2.xml
91. Davies JM, Berman D, Beggs PJ, et al. Global Climate Change and Pollen Aeroallergens: A Southern Hemisphere Perspective. *Immunol Allergy Clin North Am*. 2021;41(1):1-16. doi:10.1016/J.IAC.2020.09.002
92. Sofiev M, Bergmann KC. Allergenic pollen: A review of the production, release, distribution and health impacts. *Allergenic Pollen A Rev Prod Release, Distrib Heal Impacts*. Published online May 1, 2012:1-247. doi:10.1007/978-94-007-4881-1
93. Pfaar O, Bastl K, Berger U, et al. Defining pollen exposure times for clinical trials of allergen immunotherapy for pollen-induced rhinoconjunctivitis - an EAACI position paper. *Allergy*. 2017;72(5):713-722. doi:10.1111/ALL.13092
94. Sofiev M, Vira J, Kouznetsov R, Prank M, Soares J, Genikhovich E. Construction of the SILAM

Eulerian atmospheric dispersion model based on the advection algorithm of Michael Galperin. *Geosci Model Dev*. 2015;8(11):3497-3522. doi:10.5194/GMD-8-3497-2015
95. Sofiev M, Siljamo P, Ranta H, et al. A numerical model of birch pollen emission and dispersion in the atmosphere. Description of the emission module. *Int J Biometeorol*. 2013;57(1):45-58. doi:10.1007/S00484-012-0532-Z/FIGURES/4
96. Palamarchuk J, Sofiev M. The clinically relevant pollen season characteristics (season start/duration/end) for Alder, Birch, Olives computed over Europe based on SILAM model retrospective analysis. Finnish Meteorological Institute. Published 2022. http://doi.org/10.23728/fmi-b2share.d5e2bf8ac4e149bc9776b5514937d5d2
97. Linkosalo T, Ranta H, Oksanen A, et al. A double-threshold temperature sum model for predicting the flowering duration and relative intensity of Betula pendula and B. pubescens. *Agric For Meteorol*. 2010;150(12):1579-1584. doi:10.1016/J.AGRFORMET.2010.08.007
98. Sofiev M, Siljamo P, Ranta H, Rantio-Lehtimäki A. Towards numerical forecasting of long-range air transport of birch pollen: Theoretical considerations and a feasibility study. *Int J Biometeorol*. 2006;50(6):392-402. doi:10.1007/S00484-006-0027-X/FIGURES/5
99. Sofiev M, Berger U, Prank M, et al. MACC regional multi-model ensemble simulations of birch pollen dispersion in Europe. *Atmos Chem Phys*. 2015;15(14):8115-8130. doi:10.5194/ACP-15-8115-2015
100. Sofiev M, Ritenberga O, Albertini R, et al. Multi-model ensemble simulations of olive pollen distribution in Europe in 2014: Current status and outlook. *Atmos Chem Phys*. 2017;17(20):12341-12360. doi:10.5194/ACP-17-12341-2017
101. Sofiev M. On impact of transport conditions on variability of the seasonal pollen index. *Aerobiologia (Bologna)*. 2017;33(1):167. doi:10.1007/S10453-016-9459-X
102. Faroux S, Kaptué Tchuenté AT, Roujean J-L, Masson V, Martin E, Le Moigne P. ECOCLIMAP-II/Europe: a twofold database of ecosystems and surface parameters at 1 km resolution based on satellite information for use in land surface, meteorological and climate models. *Geosci Model Dev*. 2013;6:563-582. doi:10.5194/gmd-6-563-2013
103. Köble R, Seufert G. Novel maps for forest tree species in Europe, in: European Symp. on the Physico-Chemical Behaviour of Air Pollutants: " A Changing Atmosphere ". Presented at the European Symp. on the Physico-Chemical Behaviour of Air Pollutants: " A Changing Atmosphere ", Torino.
104. Earth Resources Observation And Science (EROS) Center. Global Land Cover Characterization (GLCC). Published 2017. Accessed March 18, 2022. https://doi.org/10.5066/F7GB230D
105. Prank M, Chapman DS, Bullock JM, et al. An operational model for forecasting ragweed pollen release and dispersion in Europe. *Agric For Meteorol*. 2013;182-183:43-53. doi:10.1016/J.AGRFORMET.2013.08.003
106. Ritenberga O, Sofiev M, Siljamo P, et al. A statistical model for predicting the inter-annual variability of birch pollen abundance in Northern and North-Eastern Europe. *Sci Total Environ*. 2018;615:228-239. doi:10.1016/J.SCITOTENV.2017.09.061
107. Sofiev M. On possibilities of assimilation of near-real-time pollen data by atmospheric composition models. *Aerobiologia (Bologna)*. 2019;35(3):523-531. doi:10.1007/S10453-019-09583-1
108. Ranta H, Kubin E, Siljamo P, et al. Long distance pollen transport cause problems for determining the

timing of birch pollen season in Fennoscandia by using phenological observations. *http://dx.doi.org/101080/00173130600984740*. 2007;45(4):297-304. doi:10.1080/00173130600984740

109. Veriankaitė L, Siljamo P, Sofiev M, Šaulienė I, Kukkonen J. Modelling analysis of source regions of long-range transported birch pollen that influences allergenic seasons in Lithuania. *Aerobiologia (Bologna)*. 2010;26(1):47-62. doi:10.1007/S10453-009-9142-6/FIGURES/8
110. Caffarra A, Zottele F, Gleeson E, Donnelly A. Spatial heterogeneity in the timing of birch budburst in response to future climate warming in Ireland. *Int J Biometeorol*. 2014;58(4):509-519. doi:10.1007/S00484-013-0720-5/FIGURES/7
111. Volkova O, Severova E, Nosova M. Six years of observation of airborne and deposited pollen in central European Russia: first results. *http://dx.doi.org/101080/0017313420161157206*. 2016;55(4):311-318. doi:10.1080/00173134.2016.1157206
112. Ziello C, Sparks TH, Estrella N, et al. Changes to Airborne Pollen Counts across Europe. Añel JA, ed. *PLoS One*. 2012;7(4):e34076. doi:10.1371/journal.pone.0034076
113. Ziska LH, Makra L, Harry SK, et al. Temperature-related changes in airborne allergenic pollen abundance and seasonality across the northern hemisphere: a retrospective data analysis. *Lancet Planet Heal*. 2019;3(3):e124-e131. doi:10.1016/S2542-5196(19)30015-4/ATTACHMENT/EB72CDF5-ED53-415E-B232-96A29D979133/MMC1.PDF
114. World Health Organization. 2021 WHO health and climate change global survey report. Published online 2021:1-79. Accessed March 18, 2022. https://www.who.int/health-topics/climate-change#tab=tab_1
115. Didan K. MOD13Q1 MODIS/Terra Vegetation Indices 16-Day L3 Global 250m SIN Grid V006. NASA EOSDIS Land Processes DAAC. *USGS*. 2015;5:2002-2015. doi:10.5067/MODIS
116. Didan K, Barreto Munoz A, Solano R, Huete A. *MODIS Vegetation Index User's Guide (MOD13 Series).*; 2015.
117. USGS. LP DAAC - MOD44W.
118. Population on 1 January by age group, sex and NUTS 3 region[demo_r_pjangrp3] Last update: 11-03-2021. Accessed March 23, 2022. https://appsso.eurostat.ec.europa.eu/nui/show.do?dataset=demo_r_pjangrp3&lang=en
119. Gascon M, Triguero-Mas M, Martínez D, et al. Residential green spaces and mortality: A systematic review. *Environ Int*. 2016;86:60-67. doi:10.1016/J.ENVINT.2015.10.013
120. Tree Cover Density — Copernicus Land Monitoring Service. Accessed March 18, 2022. https://land.copernicus.eu/pan-european/high-resolution-layers/forests/tree-cover-density
121. Urban Atlas 2018 — Copernicus Land Monitoring Service. Accessed March 18, 2022. https://land.copernicus.eu/local/urban-atlas/urban-atlas-2018
122. Street Tree Layer (STL) 2018 — Copernicus Land Monitoring Service. Accessed March 18, 2022. https://land.copernicus.eu/local/urban-atlas/street-tree-layer-stl-2018
123. International Energy Agency. CO2 Emissions from Fuel Combustion (2021 edition). Accessed March 18, 2022. http://www.iea.org/t&c/termsandconditions/.
124. International Environment Agency. World Extended Energy Balances (2021 edition). Accessed March 18, 2022. https://www.oecd-ilibrary.org/energy/data/iea-world-energy-statistics-and-balances/extended-

world-energy-balances-edition-2021_cac5fa90-en
125. Modal split of passenger transport. Accessed March 18, 2022. http://appsso.eurostat.ec.europa.eu/nui/show.do?dataset=tran_hv_psmod
126. Amann M, Bertok I, Borken-Kleefeld J, et al. Cost-effective control of air quality and greenhouse gases in Europe: Modeling and policy applications. *Environ Model Softw*. 2011;26(12):1489-1501. doi:10.1016/J.ENVSOFT.2011.07.012
127. Simpson D, Benedictow A, Berge H, et al. The EMEP MSC-W chemical transport model: technical description. *Atmos Chem Phys*. 2012;12(16):7825-7865. doi:10.5194/ACP-12-7825-2012
128. Menut L, Bessagnet B, Khvorostyanov D, et al. CHIMERE 2013: a model for regional atmospheric composition modelling. *Geosci Model Dev*. 2013;6(4):981-1028. doi:10.5194/GMD-6-981-2013
129. Kiesewetter G, Borken-Kleefeld J, Schöpp W, et al. Modelling street level PM10 concentrations across Europe: Source apportionment and possible futures. *Atmos Chem Phys*. 2015;15(3):1539-1553. doi:10.5194/ACP-15-1539-2015
130. Chen J, Hoek G. Long-term exposure to PM and all-cause and cause-specific mortality: A systematic review and meta-analysis. *Environ Int*. 2020;143:105974. doi:10.1016/J.ENVINT.2020.105974
131. Energy balances - Energy - Eurostat. Accessed March 18, 2022. https://ec.europa.eu/eurostat/web/energy/data/energy-balances
132. Food and Agriculture Organization of the United Nations. FAOSTAT Statistical Database. Published 2022. Accessed March 18, 2022. https://www.fao.org/faostat/en/#home
133. IFASTAT. Accessed March 18, 2022. https://www.fertilizer.org/Public/Market_Intelligence/IFASTAT/Public/IFA_Stat/IFASTAT.aspx?hkey=16e71efa-7bd3-421e-8344-3a191a46078f
134. World Urbanization Prospects 2018 - Population Division - United Nations. Accessed March 14, 2022. https://population.un.org/wup/Download/
135. Tilman D, Clark M. Global diets link environmental sustainability and human health. *Nat 2014 5157528*. 2014;515(7528):518-522. doi:10.1038/nature13959
136. Springmann M, Godfray HCJ, Rayner M, Scarborough P. Analysis and valuation of the health and climate change cobenefits of dietary change. *Proc Natl Acad Sci U S A*. 2016;113(15):4146-4151. doi:10.1073/PNAS.1523119113/SUPPL_FILE/PNAS.1523119113.SAPP.PDF
137. Poore J, Nemecek T. Reducing food's environmental impacts through producers and consumers. *Science (80-)*. 2018;360(6392):987-992. doi:10.1126/science.aaq0216
138. Food and Agriculture Organization of the United Nations. Food balance sheets: a handbook. Accessed March 18, 2022. https://www.fao.org/3/x9892e/X9892e.htm
139. Gustavsson J, Cederberg C, Sonesson U, Van Otterdijk R, Meybeck A, Rome F. Global food losses and food waste: extent, causes and prevention. *Food Agric Organ*. Published online 2011:1-37. Accessed March 18, 2022. http://www.fao.org/news/story/en/item/74192/icode/
140. Miller V, Singh GM, Onopa J, et al. Global Dietary Database 2017: data availability and gaps on 54 major foods, beverages and nutrients among 5.6 million children and adults from 1220 surveys worldwide. *BMJ Glob Heal*. 2021;6(2):e003585. doi:10.1136/BMJGH-2020-003585
141. Del Gobbo LC, Khatibzadeh S, Imamura F, et al. Assessing global dietary habits: a comparison of

national estimates from the FAO and the Global Dietary Database. *Am J Clin Nutr*. 2015;101(5):1038-1046. doi:10.3945/AJCN.114.087403
142. Micha R, Khatibzadeh S, Shi P, Andrews KG, Engell RE, Mozaffarian D. Global, regional and national consumption of major food groups in 1990 and 2010: a systematic analysis including 266 country-specific nutrition surveys worldwide. *BMJ Open*. 2015;5(9):e008705. doi:10.1136/BMJOPEN-2015-008705
143. Freedman LS, Commins JM, Moler JE, et al. Pooled Results From 5 Validation Studies of Dietary Self-Report Instruments Using Recovery Biomarkers for Energy and Protein Intake. *Am J Epidemiol*. 2014;180(2):172-188. doi:10.1093/AJE/KWU116
144. Rennie KL, Coward A, Jebb SA. Estimating under-reporting of energy intake in dietary surveys using an individualised method. *Br J Nutr*. 2007;97(6):1169-1176. doi:10.1017/S0007114507433086
145. Di Cesare M, Bentham J, Stevens GA, et al. Trends in adult body-mass index in 200 countries from 1975 to 2014: A pooled analysis of 1698 population-based measurement studies with 19.2 million participants. *Lancet*. 2016;387(10026):1377-1396. doi:10.1016/S0140-6736(16)30054-X/ATTACHMENT/50E15529-B93E-4149-B990-695FF383D537/MMC1.PDF
146. Murray CJL, Ezzati M, Lopez AD, Rodgers A, Vander Hoorn S. Comparative quantification of health risks: Conceptual framework and methodological issues. *Popul Health Metr*. 2003;1(1):1-20. doi:10.1186/1478-7954-1-1/FIGURES/4
147. S.S. L, T. V, A.D. F, et al. A comparative risk assessment of burden of disease and injury attributable to 67 risk factors and risk factor clusters in 21 regions, 1990-2010: A systematic analysis for the Global Burden of Disease Study 2010. *Lancet*. 2012;380(9859):2224-2260. doi:http://dx.doi.org/10.1016/S0140-6736%2812%2961766-8
148. Forouzanfar MH, Alexander L, Bachman VF, et al. Global, regional, and national comparative risk assessment of 79 behavioural, environmental and occupational, and metabolic risks or clusters of risks in 188 countries, 1990-2013: A systematic analysis for the Global Burden of Disease Study 2013. *Lancet*. 2015;386(10010):2287-2323. doi:10.1016/S0140-6736(15)00128-2/ATTACHMENT/8ECECF19-44E8-4A5A-843A-B063BA3F32E2/MMC1.PDF
149. Murray CJL, Ezzati M, Flaxman AD, et al. GBD 2010: Design, definitions, and metrics. *Lancet*. 2012;380(9859):2063-2066. doi:10.1016/S0140-6736(12)61899-6/ATTACHMENT/40CF69A5-C53F-4177-85F3-F2177FA6EC29/MMC1.PDF
150. Abbafati C, Machado DB, Cislaghi B, et al. Global age-sex-specific fertility, mortality, healthy life expectancy (HALE), and population estimates in 204 countries and territories, 1950–2019: a comprehensive demographic analysis for the Global Burden of Disease Study 2019. *Lancet*. 2020;396(10258):1160-1203. doi:10.1016/S0140-6736(20)30977-6/ATTACHMENT/4E76F1CB-0626-4875-9CF1-96DD065CD148/MMC2.PDF
151. Afshin A, Micha R, Khatibzadeh S, Mozaffarian D. Consumption of nuts and legumes and risk of incident ischemic heart disease, stroke, and diabetes: a systematic review and meta-analysis. *Am J Clin Nutr*. 2014;100(1):278-288. doi:10.3945/AJCN.113.076901
152. Aune D, Keum NN, Giovannucci E, et al. Nut consumption and risk of cardiovascular disease, total cancer, all-cause and cause-specific mortality: A systematic review and dose-response meta-analysis of

prospective studies. *BMC Med*. 2016;14(1):1-14. doi:10.1186/S12916-016-0730-3/TABLES/1

153. Aune D, Giovannucci E, Boffetta P, et al. Fruit and vegetable intake and the risk of cardiovascular disease, total cancer and all-cause mortality—a systematic review and dose-response meta-analysis of prospective studies. *Int J Epidemiol*. 2017;46(3):1029-1056. doi:10.1093/IJE/DYW319

154. Bechthold A, Boeing H, Schwedhelm C, et al. Food groups and risk of coronary heart disease, stroke and heart failure: A systematic review and dose-response meta-analysis of prospective studies. *https://doi.org/101080/1040839820171392288*. 2017;59(7):1071-1090. doi:10.1080/10408398.2017.1392288

155. Schwingshackl L, Hoffmann G, Lampousi AM, et al. Food groups and risk of type 2 diabetes mellitus: a systematic review and meta-analysis of prospective studies. *Eur J Epidemiol*. 2017;32(5):363-375. doi:10.1007/S10654-017-0246-Y/TABLES/1

156. Schwingshackl L, Schwedhelm C, Hoffmann G, et al. Food groups and risk of colorectal cancer. *Int J Cancer*. 2018;142(9):1748-1758. doi:10.1002/IJC.31198

157. Di Angelantonio E, Bhupathiraju SN, Wormser D, et al. Body-mass index and all-cause mortality: individual-participant-data meta-analysis of 239 prospective studies in four continents. *Lancet*. 2016;388(10046):776-786. doi:10.1016/S0140-6736(16)30175-1/ATTACHMENT/E2A32D35-AEAE-445A-8FEB-FE6D9C03DA38/MMC1.PDF

158. Singh GM, Danaei G, Farzadfar F, et al. The Age-Specific Quantitative Effects of Metabolic Risk Factors on Cardiovascular Diseases and Diabetes: A Pooled Analysis. *PLoS One*. 2013;8(7):e65174. doi:10.1371/JOURNAL.PONE.0065174

159. Micha R, Shulkin ML, Peñalvo JL, et al. Etiologic effects and optimal intakes of foods and nutrients for risk of cardiovascular diseases and diabetes: Systematic reviews and meta-analyses from the Nutrition and Chronic Diseases Expert Group (NutriCoDE). *PLoS One*. 2017;12(4):e0175149. doi:10.1371/JOURNAL.PONE.0175149

160. Afshin A, Sur PJ, Fay KA, et al. Health effects of dietary risks in 195 countries, 1990–2017: a systematic analysis for the Global Burden of Disease Study 2017. *Lancet*. 2019;393(10184):1958-1972. doi:10.1016/S0140-6736(19)30041-8/ATTACHMENT/89650CE3-EC1A-450B-AD88-3B2DB16AD72D/MMC1.PDF

161. Schwingshackl L, Knüppel S, Schwedhelm C, et al. Perspective: NutriGrade: A Scoring System to Assess and Judge the Meta-Evidence of Randomized Controlled Trials and Cohort Studies in Nutrition Research. *Adv Nutr*. 2016;7(6):994-1004. doi:10.3945/AN.116.013052

162. World Cancer Research Fund/American Institute for Cancer Research. Diet, Nutrition, Physical Activity and Cancer: A Global Perspective. Accessed March 19, 2022. https://www.wcrf.org/diet-and-cancer/

163. Aune D, Norat T, Romundstad P, Vatten LJ. Dairy products and the risk of type 2 diabetes: a systematic review and dose-response meta-analysis of cohort studies. *Am J Clin Nutr*. 2013;98(4):1066-1083. doi:10.3945/AJCN.113.059030

164. Aune D, Lau R, Chan DSM, et al. Dairy products and colorectal cancer risk: a systematic review and meta-analysis of cohort studies. *Ann Oncol*. 2012;23(1):37-45. doi:10.1093/ANNONC/MDR269

165. Mohan D, Mente A, Dehghan M, et al. Associations of Fish Consumption With Risk of Cardiovascular Disease and Mortality Among Individuals With or Without Vascular Disease From 58 Countries. *JAMA*

Intern Med. 2021;181(5):631-649. doi:10.1001/JAMAINTERNMED.2021.0036

166. Satija A, Yu E, Willett WC, Hu FB. Understanding Nutritional Epidemiology and Its Role in Policy. *Adv Nutr*. 2015;6(1):5-18. doi:10.3945/AN.114.007492

167. Zheng J, Huang T, Yu Y, Hu X, Yang B, Li D. Fish consumption and CHD mortality: an updated meta-analysis of seventeen cohort studies. *Public Health Nutr*. 2012;15(4):725-737. doi:10.1017/S1368980011002254

168. Aune D, Keum N, Giovannucci E, et al. Whole grain consumption and risk of cardiovascular disease, cancer, and all cause and cause specific mortality: systematic review and dose-response meta-analysis of prospective studies. *BMJ*. 2016;353. doi:10.1136/BMJ.I2716

169. MacMahon S, Baigent C, Duffy S, et al. Body-mass index and cause-specific mortality in 900 000 adults: Collaborative analyses of 57 prospective studies. *Lancet*. 2009;373(9669):1083-1096. doi:10.1016/S0140-6736(09)60318-4/ATTACHMENT/43FF481A-61DA-4EDE-9FC5-F7D72B899318/MMC1.PDF

170. Schwingshackl L, Hoffmann G, Iqbal K, Schwedhelm C, Boeing H. Food groups and intermediate disease markers: a systematic review and network meta-analysis of randomized trials. *Am J Clin Nutr*. 2018;108(3):576-586. doi:10.1093/AJCN/NQY151

171. Xun P, Qin B, Song Y, et al. Fish consumption and risk of stroke and its subtypes: accumulative evidence from a meta-analysis of prospective cohort studies. *Eur J Clin Nutr 2012 6611*. 2012;66(11):1199-1207. doi:10.1038/ejcn.2012.133

172. Jayedi A, Shab-Bidar S, Eimeri S, Djafarian K. Fish consumption and risk of all-cause and cardiovascular mortality: a dose–response meta-analysis of prospective observational studies. *Public Health Nutr*. 2018;21(7):1297-1306. doi:10.1017/S1368980017003834

173. Zhao LG, Sun JW, Yang Y, Ma X, Wang YY, Xiang YB. Fish consumption and all-cause mortality: a meta-analysis of cohort studies. *Eur J Clin Nutr 2016 702*. 2015;70(2):155-161. doi:10.1038/ejcn.2015.72

174. Guasch-Ferré M, Satija A, Blondin SA, et al. Meta-Analysis of Randomized Controlled Trials of Red Meat Consumption in Comparison with Various Comparison Diets on Cardiovascular Risk Factors. *Circulation*. 2019;139(15):1828-1845. doi:10.1161/CIRCULATIONAHA.118.035225

175. Schwingshackl L, Knüppel S, Michels N, et al. Intake of 12 food groups and disability-adjusted life years from coronary heart disease, stroke, type 2 diabetes, and colorectal cancer in 16 European countries. *Eur J Epidemiol*. 2019;34(8):765-775. doi:10.1007/S10654-019-00523-4/TABLES/3

176. Dasgupta S, van Maanen N, Gosling SN, Piontek F, Otto C, Schleussner CF. Effects of climate change on combined labour productivity and supply: an empirical, multi-model study. *Lancet Planet Heal*. 2021;5(7):e455-e465. doi:10.1016/S2542-5196(21)00170-4/ATTACHMENT/F9ABD22F-AA15-40B1-A694-ACA36E0FA68F/MMC1.PDF

177. Springmann M, Wiebe K, Mason-D'Croz D, Sulser TB, Rayner M, Scarborough P. Health and nutritional aspects of sustainable diet strategies and their association with environmental impacts: a global modelling analysis with country-level detail. *Lancet Planet Heal*. 2018;2(10):e451-e461. doi:10.1016/S2542-5196(18)30206-7

178. Springmann M, Clark M, Mason-D'Croz D, et al. Options for keeping the food system within

environmental limits. *Nat 2018 5627728*. 2018;562(7728):519-525. doi:10.1038/s41586-018-0594-0

179. Mortality Risk Valuation in Environment, Health and Transport Policies - OECD. Accessed March 19, 2022. https://www.oecd.org/environment/mortalityriskvaluationinenvironmenthealthandtransportpolicies.htm

180. Lindhjem H, Navrud S, Braathen NA, Biausque V. Valuing Mortality Risk Reductions from Environmental, Transport, and Health Policies: A Global Meta-Analysis of Stated Preference Studies. *Risk Anal*. 2011;31(9):1381-1407. doi:10.1111/J.1539-6924.2011.01694.X

181. Berrang-Ford L, Sietsma AJ, Callaghan M, et al. Systematic mapping of global research on climate and health: a machine learning review. *Lancet Planet Heal*. 2021;5(8):e514-e525. doi:10.1016/S2542-5196(21)00179-0/ATTACHMENT/6A529F7F-DF6B-4C1B-B5AB-258A524E6205/MMC2.XLSX

182. Chang C-C, Lin C-J. LIBSVM: A Library for Support Vector Machines. Accessed March 19, 2022. www.csie.ntu.edu.tw/

183. Halterman A. Mordecai: Full Text Geoparsing and Event Geocoding. *J Open Source Softw*. 2017;2(9):91. doi:10.21105/JOSS.00091

184. Akerlof K, Debono R, Berry P, et al. Public Perceptions of Climate Change as a Human Health Risk: Surveys of the United States, Canada and Malta. *Int J Environ Res Public Health*. 2010;7(6):2559. doi:10.3390/IJERPH7062559

185. Maibach EW, Kreslake JM, Roser-Renouf C, Rosenthal S, Feinberg G, Leiserowitz AA. Do americans understand that global warming is harmful to human health? Evidence from a national survey. *Ann Glob Heal*. 2015;81(3):396-409. doi:10.1016/J.AOGH.2015.08.010/METRICS/

186. Cody EM, Reagan AJ, Mitchell L, Dodds PS, Danforth CM. Climate Change Sentiment on Twitter: An Unsolicited Public Opinion Poll. *PLoS One*. 2015;10(8):e0136092. doi:10.1371/JOURNAL.PONE.0136092

187. Sanford M, Painter J, Yasseri T, Lorimer J. Controversy around climate change reports: a case study of Twitter responses to the 2019 IPCC report on land. *Clim Change*. 2021;167(3-4):1-25. doi:10.1007/S10584-021-03182-1/TABLES/5

188. Rahimi A, Baldwin T, Cohn T. Continuous Representation of Location for Geolocation and Lexical Dialectology using Mixture Density Networks. *EMNLP 2017 - Conf Empir Methods Nat Lang Process Proc*. Published online August 14, 2017:167-176. doi:10.48550/arxiv.1708.04358

189. Thomas P, Hennig L. Twitter geolocation prediction using neural networks. *Lect Notes Comput Sci (including Subser Lect Notes Artif Intell Lect Notes Bioinformatics)*. 2018;10713 LNAI:248-255. doi:10.1007/978-3-319-73706-5_21/TABLES/3

190. Mahajan R, Mansotra V. Predicting Geolocation of Tweets: Using Combination of CNN and BiLSTM. *Data Sci Eng*. 2021;6(4):402-410. doi:10.1007/S41019-021-00165-1/FIGURES/5

191. Bianchi F, Terragni S, Hovy D. Pre-training is a Hot Topic: Contextualized Document Embeddings Improve Topic Coherence. *ACL-IJCNLP 2021 - 59th Annu Meet Assoc Comput Linguist 11th Int Jt Conf Nat Lang Process Proc Conf*. 2021;2:759-766. doi:10.18653/V1/2021.ACL-SHORT.96

192. Bianchi F, Terragni S, Hovy D, Nozza D, Fersini E. Cross-lingual Contextualized Topic Models with Zero-shot Learning. *EACL 2021 - 16th Conf Eur Chapter Assoc Comput Linguist Proc Conf*. Published online 2021:1676-1683. doi:10.18653/V1/2021.EACL-MAIN.143

193. European Parliament. Accessed March 19, 2022. https://www.europarl.europa.eu/portal/en
194. google-trans-new · PyPI. Accessed March 19, 2022. https://pypi.org/project/google-trans-new/
195. GitHub - Helsinki-NLP/Opus-MT: Open neural machine translation models and web services. Accessed March 19, 2022. https://github.com/Helsinki-NLP/Opus-MT
196. Wolf T, Debut L, Sanh V, et al. Transformers: State-of-the-Art Natural Language Processing. Published online November 29, 2020:38-45. doi:10.18653/V1/2020.EMNLP-DEMOS.6
197. Cai W, Zhang C, Zhang S, et al. The 2021 China report of the Lancet Countdown on health and climate change: seizing the window of opportunity. *Lancet Public Heal*. 2021;6(12):e932-e947. doi:10.1016/S2468-2667(21)00209-7
